# Comparative transcriptome analysis of the effects of friction and exogenous gibberellin on germination in *Abrus cantoniensis*

**DOI:** 10.1080/15592324.2022.2149113

**Published:** 2022-11-30

**Authors:** Zhu Yanxia, Jiang Jianping, Huang Yanfen, Dong Qingsong, Wei Kunhua

**Affiliations:** a Guangxi Key Laboratory of Medicinal Resources Protection and Genetic Improvement, Guangxi Botanical Garden of Medicinal Plants, Nanning, China; b Guangxi Key Laboratory of High-quality Formation and Utilization of Dao-di Herbs, Guangxi Botanical Garden of Medicinal Plants, Nanning, China

**Keywords:** *Abrus cantoniensis*, transcriptome analysis, seed germination, candidate gene

## Abstract

The seeds of *Abrus cantoniensis* (*A. cantonensis*) have dormancy characteristics with very low germination under natural conditions. In general, its seed dormancy could be broken by friction or soaking with exogenous gibberellins (GA_3_). To date, the molecular mechanism underlying the effects of GA_3_ and friction on its seed germination is unclear. In this study, we tested the effects of different treatments, including soaking in sterile water (G1), friction (G2), soaking in GA_3_ (G3), combined treatment of friction, and GA_3_ (G4)) on seed germination. Then, we have investigated the seed transcriptome profiles corresponding to the different treatments by RNA sequencing. The results showed that seed germination was significantly increased by combined treatment with friction and GA_3_. RNA-Seq analysis generated 84.80 gigabases (Gb) of sequences. 82,996 out of 121,776 unigenes were annotated. Comparative transcriptome analysis observed that 1,130, 1,097, and 708 unigenes were deferentially expressed in G1 vs. G2, G1 vs. G3, and G1 vs. G4 groups, respectively. Additionally, 20 putatively candidate genes related to seed germination, including *CYP78A5, Bg*7s, *GA-20-ox, rd22, MYB*4, *LEA, CHS*, and *STH*-2, and other potential candidates with abundant expression were identified. Our findings provide first insights into gene expression profiles and physiological response for friction combined with GA_3_ on *A. cantoniensis* seed germination.

## 1. Introduction

*Abrus cantoniensis* (*A. cantonensis*), commonly named “Jigucao” in Chinese, is a kind of traditional medicinal plant that mainly distributed in Guangdong and Guangxi in China. Its dried plant is the main raw materials of traditional Chinese medicines such as “Jigucao capsule”, “Jigucao Hepatitis Granule”, “Jieshitong tablet”, and “Gandele capsule”, which has effects on hepatoprotective, antioxidation, anti-inflammation, and anti-proliferation^1^, ^2^.

The seeds of *A. cantoniensis* have a typical dormancy characteristic with harder seed coat and lower water permeability. Notably, about 98% of the seeds of *A. cantoniensis* are hard seeds.^3^ It is reported that *A. cantoniensis* seed dormancy can be relieved by soaking in sulfuric acid, scalding with boiling water, and friction.^4–6^ Particularly, accumulative evidence indicated that gibberellin (GA_3_) plays a critical role in breaking seed dormancy, and a wealth of information is available on the molecular mechanism of GA_3_ on seed germination.^7–10^

With the rapid development of high-throughput sequencing technology, RNA-seq has been widely used to study the gene expression profiles, unraveled a large number of response elements, and illustrated the molecular mechanisms in plants.^11–13^ To date, the molecular mechanism of friction and GA_3_ treatment on the seed germination of *A. cantoniensis* remains unclear.

In this study, we aimed to determine the effects of different treatment (including friction soaking in GA_3_ and friction combined with GA_3_) on seed germination and performed seed transcriptomic sequencing to investigate the gene expression profiles and therefore to unravel the candidate genes related to seed germination and elucidate the physiological and molecular mechanisms underlying the seed germination. Taken together, our transcriptome data may be used for further research on *A. cantoniensis*.

## 2. Materials and methods

### 2.1 Plant materials

**Figure 1.**
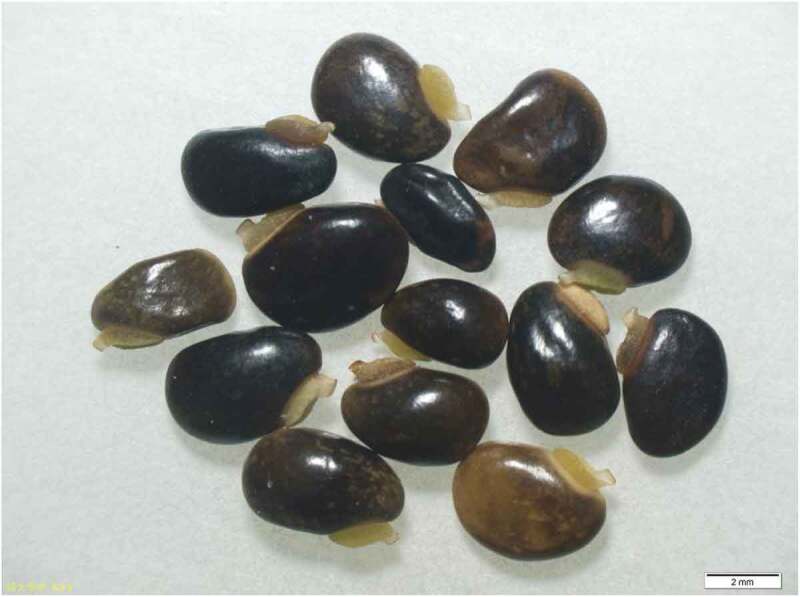
The seed morphology of *A. cantonensis.*

*A. Cantonensis* seeds were collected in Lingshan County, Qinzhou City, Guangxi Province (109°13′E, 22°24′N), in November 2020. The plant was formally identified by Professor Dong Qingsong from Guangxi Botanical Garden of Medicinal Plants. The seed morphology is shown in Figure 1. The weight of 100 seeds was 1.687 ± 0.035 g.

The seeds were placed on two layers of filter paper soaked with sterile water and saturated with sterile incubation in a controlled environment (25°C, photoperiod of 8 h light/16 h dark),100 seeds per petri dish. Then, samples were divided into four experimental groups: G1 (seeds are soaked in sterile water for 24 h and then placed in the petri dish), G2 (seeds are rubbed in a rice milling machine for 90 s and then placed in the petri dish), G3 (seeds are soaked in 100 mg/L gibberellin solution for 24 h and then placed in the petri dish), and G4 (seeds are rubbed in a rice milling machine for 90 s and then immersed in 100 mg/L gibberellin solution for 24 h and then placed in the petri dish), and 4 replicates are set for each treatment. After sowing, the number of germinated seeds was counted every day until the end of germination on the 10th day, that is, the number of germinated seeds will no longer increase, then the germination was summarized, and statistical analysis is conducted as described by Deng et al.,^14^

$$\begin{array}{rl} & Germination\;(\%)=100\%\times \frac{Number\;of\;germinated\;seeds}{number\;of\;tested\;seeds} \end{array}$$

Five days later, germinated seeds from each of four groups with three biological replicates were collected to conduct RNA sequencing. Additionally, germinated seeds were fixed in FAA (50% ethanol:glacial acetic acid:38% formaldehyde = 90:5:5) nd then stored at room temperature. The samples were dehydrated with 50% alcohol, clarified with turpentine oil (Guangxi Cenxi rosin factory, 500 mL/bottle), then embedded in paraffin, subsequently, cut into slices with a thickness of 8 μm, and finally stained with safranin and solid green. The images were taken using an Olympus bx-51 imaging system.

### 2.2 RNA extraction, library construction, and transcriptome sequencing

Approximately 20 frozen seed samples for each treatment were used for RNA extraction. Total RNA was isolated using the RNAeasy Plant Mini Kit (Qiagen, Hilden) and then treated with DNase I (RNase-free) following the manufacturer’s instructions. The concentration and integrity of RNA were determined using a NanoDrop 2000 spectrophotometer (Thermo Scientific, USA) and an Agilent Bioanalyzer 2100 system (Agilent Technologies, California, USA). High-quality RNA samples were used to construct libraries by applying to NEBNext®Ultra™ RNALibrary Prep Kit for Illumina®(NEB, USA) in accordance with the manufacturer’s protocols and then sequenced by the Illumina Hiseq 2000 platform (Illumina, San Diego, CA, USA).

### 2.3 De novo assembly and functional annotation

Raw data were processed with the FastQC program. Clean reads were obtained by removing adapter sequences, poly-N sequences, and low-quality reads. High-quality reads were then assembled using Trinity software package^15^ with min_kmer_cov set to 2 by default and all other parameters set default. Then, nonredundant unigenes were generated from assembly sequences. Functional annotation of unigenes was performed based on the following databases: NR (NCBI nonredundant protein sequences), Pfam (Protein family), KOG/COG/eggNOG (Clusters of Orthologous Groups of proteins), Swiss-Prot (A manually annotated and reviewed protein sequence database), KEGG (Kyoto Encyclopedia of Genes and Genomes), and GO (Gene Ontology) database.

### 2.4 Differential gene expression and gene functional enrichment analyses

Gene expression levels were estimated by RSEM^16^ by mapping to the assembled transcriptome. Differential expression analysis was performed using the DESeq R package (1.10.1) within FDR< 0.01, |log2 (fold change)| > 2. In addition, GO enrichment and KEGG pathway analysis of DEGs was conducted using the topGO R packages and KOBAS software,^17^ respectively.

### 2.5 Real-time quantitative RT-PCR (qRT-PCR) analysis

Ten DEGs were selected to verify the expression level using qRT-PCR with the same samples of RNA-seq. The genes and primers are listed in Table S1. Total RNAs were extracted as mentioned before. cDNAs were synthesized using the PrimeScript RT reagent kit with gDNA eraser (TaKaRa Biotechnology, Dalian, China). qRT-PCR was performed using SYBR Premix Ex Taq (TliRNaseH Plus; TaKaRa Biotechnology,Dalian, China). The reaction conditions are as follows: pre-denaturing for 30 s at 95°C, followed by 40 cycles of 5 s at 95°C and 40 s at 60°C. The relative expression levels of the selected genes were normalized with GAPDH serving as housekeeping gene and calculated using the 2^−ΔΔCt^ method.^18^

## 3. Results

### 3.1 Effects of friction and soaking in GA_3_ on seed germination and germination morphology

**Figure 2.**
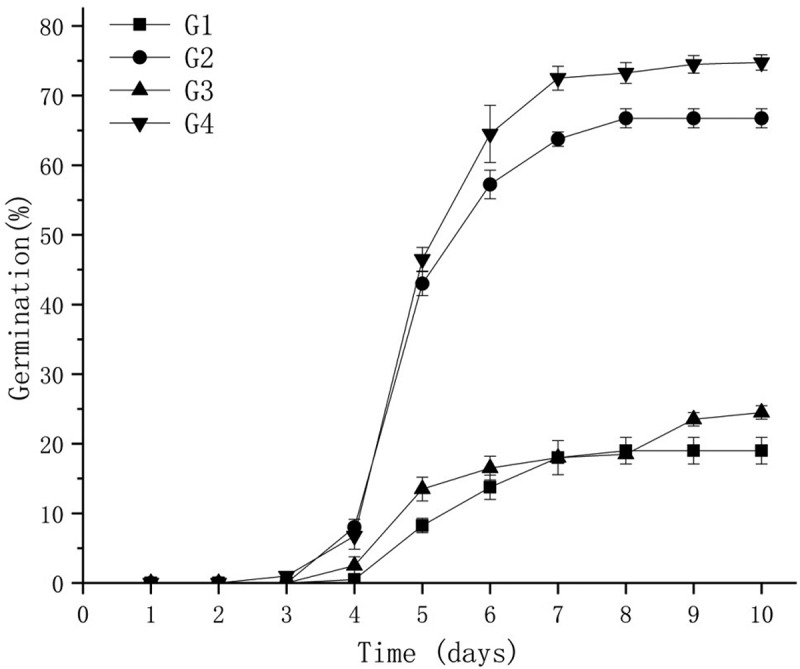
The change trend of *A. cantonensi*s seed germination with the time.

The results showed that combined treatment by friction and GA_3_ significantly increased the seed germination of *A.cantonensis*, and the statistics of seed germination were 19%, 67%, 25%, and 75% for G1-, G2-, G3-, and G4-treated seeds, respectively (Figure 2). The seeds began to germinate on the fourth day, and the germination increased rapidly on the fifth day, then increased further with the extension of time, and finally reached a stable state on the 10th day.

**Figure 3.**
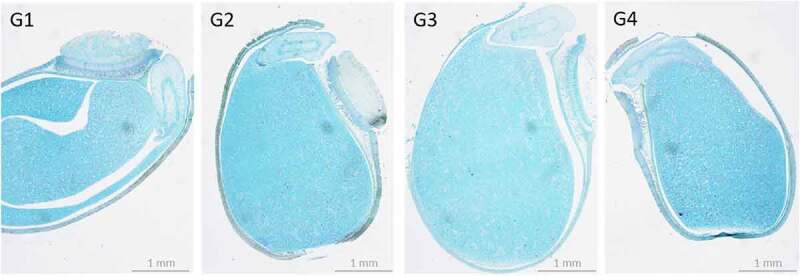
The morphology during the early stage of germination of *A. cantonensi*s seeds.

Microscopic observation showed that there was no difference in seed morphology after germination among the four treatments. The cotyledons are complete, and the radicle and hypocotyl pass through the seed coat and grow perpendicular to the longitudinal section of the seed (Figure 3).

### 3.2 Transcriptome sequencing and de novo assembly

In total, 84.80 gigabases (Gb) clean reads were generated (Table 1). The clean reads ranged from 21,037,318 to 26,049,578 for twelve libraries. The GC% and Q30 of each library were higher than 44.16% and 92.6%, respectively. As illustrated in Table 2, 677,319 transcripts were obtained. 59.78% (404,925) transcript was with length > 2kb. A total of 121,776 unigenes were assembled with a mean size of 800 bp and N50 of 1,645 bp (Table 2).

**Table 1.** Summary statistics of seed transcriptome data of *A. cantoniensis.*

Group	Sample ID	Read Number	Base Number	Clean Reads	Mapped Reads	Mapped Ratio	GC Content	Q30(%)
G1	G11	23,862,371	7,137,423,868	23,862,371	19,163,117	80.31%	44.35%	93.31%
	G12	21,253,104	6,359,159,448	21,253,104	16,991,239	79.95%	44.38%	93.30%
	G13	26,049,578	7,792,120,184	26,049,578	20,988,403	80.57%	44.35%	93.45%
G2	G21	24,646,407	7,374,493,780	24,646,407	19,617,826	79.60%	44.22%	93.37%
	G22	24,820,613	7,418,965,518	24,820,613	19,927,998	80.29%	44.33%	93.71%
	G23	23,205,599	6,931,206,660	23,205,599	18,379,563	79.20%	44.29%	93.11%
G3	G31	24,759,756	7,402,149,094	24,759,756	19,926,494	80.48%	44.61%	93.24%
	G32	23,676,296	7,077,990,596	23,676,296	18,676,239	78.88%	44.45%	92.81%
	G33	25,792,911	7,711,922,748	25,792,911	20,654,207	80.08%	44.22%	93.33%
G4	G41	22,408,385	6,700,912,516	22,408,385	18,097,048	80.76%	44.47%	92.86%
	G42	21,037,318	6,290,794,776	21,037,318	17,086,152	81.22%	44.16%	92.73%
	G43	22,121,879	6,608,013,784	22,121,879	17,827,611	80.59%	44.53%	92.6%

**Table 2.** Statistics of unigene results.

Length Range	Transcript	Unigene
200–300	53,332 (7.87%)	47,375 (38.90%)
300–500	41,001 (6.05%)	29,950 (24.59%)
500–1000	56,060 (8.28%)	21,771 (17.88%)
1000–2000	122,001 (18.01%)	11,204 (9.20%)
>2000	404,925 (59.78%)	11,476 (9.42%)
Total number	677,319	121,776
Total length	1,882,832,990	97,524,323
N50 length	3,930	1,645
Mean length	2779.83	800.85

### 3.3 Functional annotation and classification of *A. cantoniensis* unigenes

All unigenes were searched against the NR, eggNOG, KEGG, SwissProt, Pfam, GO, and KOG/COG databases. Totally, 82,996 (68.15%) of 121,776 unigenes were annotated in eight databases. Among them, 80,438 (96.92%) out of 82,996 unigenes were annotated in NR database; 75,604 (91.09%) were obtained from eggNOG database followed by 61,134 (73.66%) in GO database. 46,120 (55.57%), 40,572 (48.88%), 35,271 (42.50%), 25,990 (31.31%), and 22,706 (27.36%) unigenes were annotated in Pfam, KOG, Swissprot, KEGG, and COG, respectively (Table 3).

**Table 3.** Functional annotations of unigenes.

Database	COG	GO	KEGG	KOG	Pfam	Swissprot	eggNOG	NR	All
Annotated_number	22,706	61,134	25,990	40,572	46,120	35,271	75,604	80,438	82,996
Pecentage	27.36%	73.66%	31.31%	48.88%	55.57%	42.50%	91.09%	96.92%	100%

**Figure 4.**
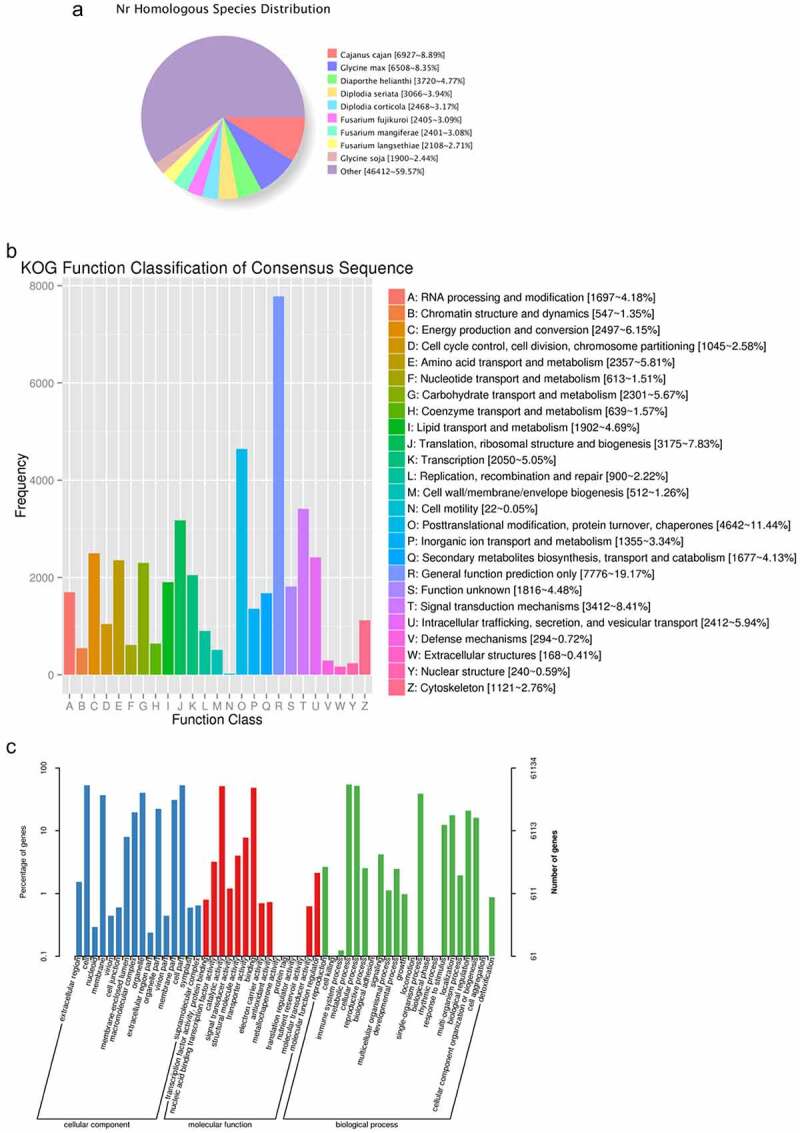
Functional annotations of the unigenes of the *A. cantoniensis* seed transcriptome. (a) The species identified by a homology search against the NR databases. (b) KOG function annotation. (c) GO function annotation.

Aligning to the NR database, *A. cantoniensis* transcripts were highly similar to those in *Cajanus cajan* (8.89%), Glycine max (8.35%), and *Diaporthe helianthi* (4.77%) (Figure 4(a)).

Through the KOG function classification, 40,572 unigenes were classified into 26 functional categories, the top three of which are as follows: General function prediction only (7,776 unigenes), posttranslational modification, protein turnover, chaperones (4,642 unigenes), and signal transduction mechanisms (3,412 unigenes) (Figure 4(b)).

Annotating to the GO database, 61,134 unigenes were annotated into 53 GO terms. Among them, “cell” (53.4%), “catalytic activity” (51.56%), and “metabolic process” (54.85%) were the main terms among the cellular component, molecular function, and biological process, respectively (Figure 4(c)).

### 3.4 DEGs identification and enrichment analysis

**Figure 5.**
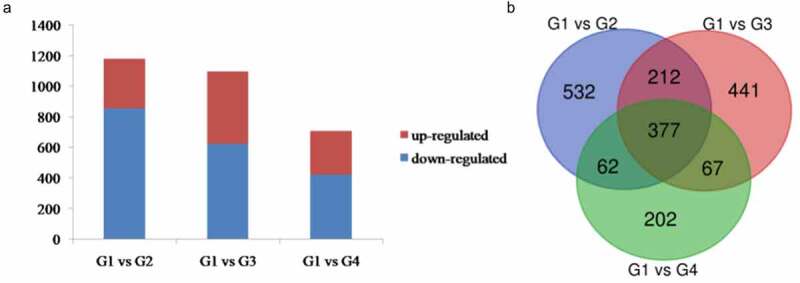
Statistics of DEGs among the three groups. (a) Statistical analysis of up-/downregulated unigenes in the three groups. (b) Venn diagram of all DEGs.

The results of DEGs across the three comparison pairs (G1 vs G2, G1 vs G3, G1 vs G4) lead in 1130, 1097, and 708 DEGs, respectively (Table S2). Among them, 857 and 326, 623 and 474, and 422 and 286 genes were downregulated and upregulated in G1 vs G2, G1 vs G3, and G1 vs G4, respectively (Figure 5(a)). Totally, 377 DEGs were common among the three groups (Figure 5(b)).

GO annotation was conducted to classify the DEGs among the three groups. It was shown that 43, 44, and 40 GO terms were significantly enriched, respectively. Few GO terms, such as metabolic process, response to stimulus, and developmental process, were involved in the seed germination of *A. cantoniensis* (Table S3).

KEGG pathway analysis was performed by KOBAS database. Totally, 9 out of 32 pathways were enriched as common pathways, including fatty acid biosynthesis, flavonoid biosynthesis, nitrogen metabolism, starch and sucrose metabolism, tyrosine metabolism, phenylpropanoid biosynthesis, diterpenoid biosynthesis, pentose and glucuronate interconversions, and isoquinoline alkaloid biosynthesis (Table S4).

### 3.5 Candidate genes related to seed germination

**Figure 6.**
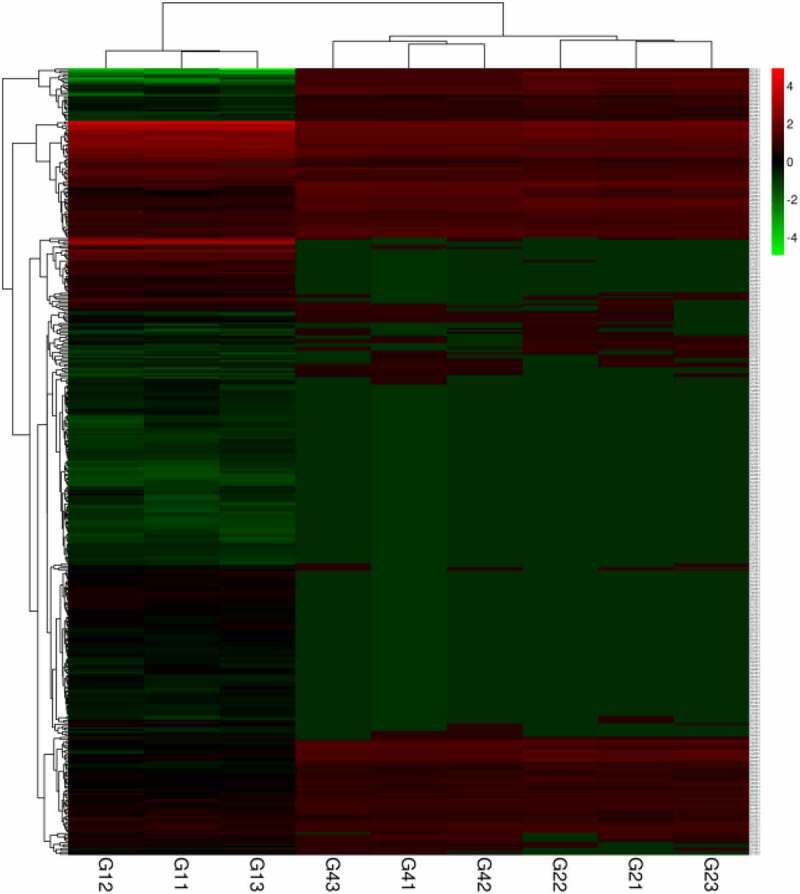
Heatmap analysis for common genes of G1 vs G2 and G1 vs G4.

According to germination tests, we mainly focused on the DEGs of G1 vs G2 and G1 vs G4 and identified 418 common genes. Heatmaps of expression patterns were obtained among the two groups (Figure 6).

On the basis of previous publications, in line with thresholds of FPKM more than 50, we selected 20 candidate genes related to seed germination (Table 4).

**Table 4.** List of candidate genes in *A. cantoniensis.*

Cluster_ID	Mean FPKM (G1)	Mean FPKM G2)	Mean FPKM (G3)	Gene Description	Hit Species
c90756.graph_c2	144.10	1088.24	465.82	*Subunit of beta-conglycinin, partial*	*Glycine max*
c37861.graph_c0	101.22	379.49	274.66	*Seed maturation protein PM35*	*Glycine max*
c82654.graph_c0	41.80	604.37	197.74	*Embryonic abundant-like protein*	*Medicago truncatula*
c71104.graph_c0	27.16	76.22	60.51	*Hypothetical protein PHAVU_009G158100g*	*Phaseolus vulgaris*
c91933.graph_c1	24.18	74.55	44.63	*Hypothetical protein GLYMA_20G183700*	*Glycine ma*x
c81168.graph_c0	23.88	51.58	46.46	*Hypothetical protein glysoja_013685*	*Glycine soja*
c81367.graph_c0	9.21	54.10	30.04	*PREDICTED: gibberellin 20-oxidase 1 isoform X1*	*Glycine max*
c89530.graph_c0	5.50	52.55	11.76	*PREDICTED: late embryogenesis abundant protein 2-like*	*Glycine max*
c80941.graph_c0	5.46	69.72	61.97	*Hypothetical protein PHAVU_002G305200g*	*Phaseolus vulgaris*
c93554.graph_c0	1.47	51.98	3.87	*sSucrose binding protein homolog S-64*	*Glycine max*
c80387.graph_c0	0.21	135.13	4.41	*Dehydration-responsive protein RD22*	*Glycine soja*
c73316.graph_c0	68.39	23.58	213.77	*PREDICTED: myb-related protein Myb4-like*	*Glycine max*
c76615.graph_c2	53.67	25.41	125.83	*PREDICTED: chalcone synthase 1*	*Lupinus angustifolius*
c80388.graph_c0	25.87	11.04	61.14	*Anthocyanidin reductase ((2S)-flavan-3-ol-forming) isoform X1*	*Cajanus cajan*
c37737.graph_c0	23.44	8.15	63.61	*Pathogenesis-related protein STH-2-like*	*Cajanus cajan*
c37849.graph_c0	10.61	0.74	73.73	*Hypothetical protein PHAVU_007G222900g*	*Phaseolus vulgaris*
c95266.graph_c0	8.31	59.80	324.89	*Basic 7S globulin precursor*	*Glycine max*
c68684.graph_c0	30.41	12.94	62.94	*Hypothetical protein GLYMA_11G070200*	*Glycine max*
c80521.graph_c0	25.90	59.27	67.19	*Cytochrome P450 78A5-like*	*Cajanus cajan*
c95706.graph_c0	56.59	0.00	0.00	*Hypothetical protein ANO11243_042010*	Fungal sp. No. 11243

### 3.6 qRT-PCR Validation

**Figure 7.**
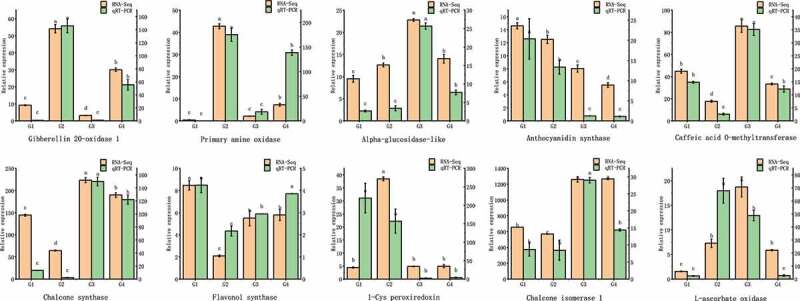
Verification of the expression levels determined by qRT-PCR and RNA-seq. Groups with different letters represent significant difference (*p* < .05).

Furthermore, 10 DEGs were randomly selected to perform qRT-PCR to validate the transcriptome data. As shown in Figure 7, eight out of ten DEGs displayed similar expression patterns compared with RNA-seq data, which indicated that our transcriptome analysis was reliable and accurate.

## 4. Discussion

As the traditional Chinese medicinal plant, a better understanding of the genetic and biological mechanisms underlying the seed germination of *A. cantoniensis* is especially significant, yet it is poorly stated. In this study, we investigated the gene expression profiles of *A. cantoniensis* under different treatments (friction, GA_3_, combined treatment of friction, and GA_3_) using RNA-seq. Totally, 121,776 unigenes were assembled and 82,996 unigenes were annotated based on the eight databases (NT, NR, Swiss-Port, PFAM, KOG, GO, COG, and KEGG). Subsequently, the DEGs were significantly enriched in nine common pathways, especially in starch and sucrose metabolism and phenylpropanoid biosynthesis pathway. It was observed that phenylpropanoid biosynthesis played an important role in seed germination in plants byRNA_seq and metabolomic analysis.^19^ Starch functioned as the main carbohydrate storage and was responsible for providing energy for seed development and germination.^20^

Of note, twenty candidate genes related to seed germination were identified through integrating analysis of the KEGG pathways, results of germination tests, and FPKM values of DEGs. According to our previous study and the current results, we found that stimulating by exogenous GA_3_ has slight effects on seed germination of *A. cantoniensis*. Hence, we paid more attention on the DEGs from G1 vs G2 and G1 vs G4 to selected candidate genes. Therefore, 20 candidates were revealed. Four clusters of 20 candidates were classified in detail. Eleven genes were upregulated in G2 and G4, and six and two out of eight genes were downregulated in G2 and upregulated in G4, respectively. One gene was only expressed in G1 (Table 4).

Among them, *β*-conglycinin is the major seed storage protein, which consists of three subunits:*α*’ (76 kDa), *α* (72 kDa), and *β* (52 kDa).^21^ Song *et al*. observed that the α-subunit of β-conglycinin deficiency had an impact on seed maturation of soybean.^22^ The α’ subunit has been reported to show a higher level in rice seed, and accumulation of *β*-conglycinin contributed to seed development for proving more nutrition.^23^

GA20 oxidase was involved in diterpenoid biosynthesis, and it is a multifunctional enzyme that catalyzes the formation of active GAs, which contributed to the regulation of GA biosynthesis.^24^ Numerous studies have demonstrated that active GAs played an essential role in plant growth, seed germination, and development.^25,26^ In the current study, GA-20-ox was significantly upregulated in G2 and G4, otherwise downregulated in G1.

*LEA* proteins are reported to a large group of hydrophilic proteins, which were involved in drought tolerance and abiotic stress in plants, fungi, and bacteria.^27,28^ As the name suggests, *LEA* protein was known to be correlated with embryo development of seeds at the late stage.^29^

A previous study has observed that dehydration-responsive gene, *rd22*, had a profound effect on response for drought stress, and it was mediated by abscisic acid (ABA).^30^ In this study, it has the highest expression pattern in G2 compared with other groups, which was consistent with Wei *et a*l.^31^

*MYB* proteins are characterized as a ubiquitous transcription factor in plants and played a key role in mediating the development and metabolism in plants.^32,33^ Recently, it has been reported that Myb4 was demonstrated to play dual roles in flavonoid biosynthesis.^33^ In our study, MYB4 was downregulated in the G2 group, whereas upregulated in the G4 group, which deduced that GA3 might modulate MYB4 and corresponding target genes to regulate seed germination.^34^

*CHS* is one of the members of the plants-specific type III polyketide synthase (PKS), which is the essential enzyme for biosynthesis of flavonoids.^35,36^ It is well known that flavonoids play a vital role in flower pigmentation, pathogen defense, auxin transport, and pollen fertility.^37,38^

Pathogenesis-related protein (also named as *STH-2*) belongs to a *PRP* family and characterized as the key indicator of acquired resistance.^39^ It was higher expression in G4 and lower expression in G2 compared with G1, respectively. This was coordinated with Li *et al*. that STH-2 might inhibit the seed germination.^40^

*Bg7S* was originally found from soybean that bound to insulin and insulin-like growth factor.^41^ It belongs to a family of storage proteins. Accumulative evidences confirmed that it played multifunctional roles, such as protein kinase activity, stress response, and antibacterial activity.^42^ In our study, it had the highest expression upon the G4 group. The results speculated that *Bg7S* is a promising candidate that was responsible for GA_3_ and friction treatment to promote seed germination

*CYP78A5* belonging to the subfamily of cytochrome P450 enzymes was known to be influencing the seed size in several plants.^43–45^ Adamski *et al*. have demonstrated that seeds with deficiency of *CYP78A5* led to smaller seed size and seedlings.^46^ Previous studies in wheat suggested that *CYP78A5* highly expressed in ovary and seed coat; meanwhile, modified expression of *CYP78A5* could increase the grain weight and grain yield via auxin accumulation.^47^ Currently, we found that treated with GA_3_ and friction, *CYP78A5* showed a higher expression level compared with the Control group. Hence, *CYP78A5* might act as a key candidate gene related to seed germination.

In addition, seed maturation protein *PM35*, embryonic abundant-like protein, cytochrome P450 *CYP73A100*-like, and sucrose binding protein homolog S-64 were key candidates for seed germination although limited studies were reported. Moreover, novel genes, which were with abundant expression, such as hypothetical protein PHAVU_009G158100g, and hypothetical protein GLYMA_20G183700, might be potential candidates.

## 5. Conclusions

The present study provided comprehensive gene expression profiles of seed transcriptome under GA_3_ and friction treatment of *A. cantoniensis*. 121,776 unigenes were obtained. Numerous DEGs and 20 candidate genes related to seed germination were identified. These findings provided a valuable database for further studies on functional analyses of candidate genes and elucidation of the molecular mechanism for *A. cantoniensis*.

## Supplementary Material

Supplemental Material
