# Supplementary material for: Comparative transcriptome analysis of the effects of friction and exogenous gibberellin on germination in Abrus cantoniensis
Source: Plant Signal Behav. 2022 Nov 30;17(1):2149113. doi: 10.1080/15592324.2022.2149113 (PMC9721420; doi:10.1080/15592324.2022.2149113)
Supplement: Supplemental Material [file KPSB_A_2149113_SM4039.zip › ╕╜▒φ/Table S1.pdf]

Table S1. DEGs idenfied among the three groups

Cluster_ID	FDR	log2FC	regulate	nr_annotation
c40224.graph_ c0	3.98E-12	-1.7628166	down	putative transmembrane alpha-helix domain-containing protein [Diaporthe ampelina]
c89071.graph_	1.15E-08	-1.4509202	down	60S ribosomal protein L2 [Eremothecium gossypii ATCC
c92264.graph_	1.98E-66	1.2011898	up	ABI-3 homolog [Psophocarpus tetragonolobus]
c82188.graph_ c0	1.34E-22	-2.2832303	down	pathogenesis associated protein Cap20, putative [Macrophomina phaseolina MS6]
c37669.graph_	7.13E-44	-3.3084692	down	versicolorin reductase [Aspergillus udagawae]
c87288.graph_	5.84E-31	-1.1290736	down	LRR receptor-like kinase plant [Medicago truncatula]
c77890.graph_	4.74E-07	-1.2267845	down	MLP-like protein 34 isoform X2 [Arachis duranensis]
c76695.graph_	4.78E-26	2.20556013	up	macrophage migration inhibitory factor homolog [Cajanus
c90547.graph_	1.27E-48	-1.777541	down	hypothetical protein GLYMA_19G093000 [Glycine max]
c82384.graph_	4.44E-303	2.81796876	up	seed maturation protein PM24 [Glycine max]
c54083.graph_	5.33E-05	-1.0458239	down	predicted protein [Phaeodactylum tricornutum CCAP
c85310.graph_	4.10E-12	-1.7136176	down	related to C6 finger domain protein [Fusarium fujikuroi]
c89943.graph_ c0	0.0001157	-1.0256987	down	UTP-glucose-1-phosphate uridylyltransferase [Fusarium oxysporum f. sp. lycopersici 4287]
c85490.graph_	8.77E-27	-1.0333776	down	kinesin-like protein KIN-5B [Cajanus cajan]
c94788.graph_ c0	3.39E-35	2.70836225	up	Retrovirus-related Pol polyprotein from transposon TNT 1-94 [Cajanus cajan]
c52452.graph_	1.24E-26	-2.652858	down	STE like transcription factor [Diaporthe helianthi]
c88231.graph_	0.0001006	-1.0465504	down	retrotransposon-related protein, partial [Trifolium pratense]
c82586.graph_ c0	4.67E-41	-1.3342378	down	Putative xyloglucan endotransglucosylase/hydrolase protein 23 [Glycine soja]
c83300.graph_ c1	8.59E-06	1.08523818	up	probable 2-oxoglutarate-dependent dioxygenase AOP1 [Cajanus cajan]
c38035.graph_	3.30E-21	-2.3757632	down	hypothetical protein DHEL01_v200180 [Diaporthe
c80846.graph_	8.22E-15	-1.1788162	down	Bis(5'-adenosyl)-triphosphatase [Glycine soja]
c76247.graph_	3.92E-37	-1.1171933	down	unknown [Glycine max]
c38123.graph_	9.90E-25	-2.5604371	down	hypothetical protein UCDDS831_g02644 [Diplodia
c95361.graph_ c0	5.19E-07	-1.3046597	down	putative polyketide synthase protein [Neofusicoccum parvum UCRNP2]
c87615.graph_	1.46E-09	-1.552886	down	hypothetical protein BK809_0004349 [Diplodia seriata]
c98390.graph_	3.84E-10	-1.5865235	down	hypothetical protein UCDDA912_g00683 [Diaporthe
c89073.graph_	7.99E-08	-1.254293	down	NAC domain-containing protein 42 [Glycine soja]
c94301.graph_	1.88E-15	1.58640008	up	predicted protein [Hordeum vulgare subsp. vulgare]
c91706.graph_	2.57E-06	-1.2345176	down	hypothetical protein FPOA_08124 [Fusarium poae]
c71104.graph_	2.44E-33	1.40430084	up	hypothetical protein PHAVU_009G158100g [Phaseolus
c76838.graph_ c0	1.11E-16	1.31091768	up	PREDICTED: glycine cleavage system H protein, mitochondrial [Glycine max]
c58605.graph_	2.10E-18	-2.2119428	down	hypothetical protein M426DRAFT_13512 [Hypoxylon sp.
c37683.graph_ c0	1.75E-10	-1.5931572	down	glutamine synthetase [Fusarium oxysporum f. sp. lycopersici MN25]
c83968.graph_	4.49E-19	-1.8072767	down	transaldolase [Fusarium langsethiae]
c77657.graph_	4.11E-10	-1.6115351	down	hypothetical protein BKCO1_1300023 [Diplodia corticola]
c40010.graph_	1.03E-41	-3.237823	down	hypothetical protein DHEL01_v202423 [Diaporthe
c68861.graph_	1.37E-21	-1.1521988	down	hypothetical protein PHAVU_008G051600g [Phaseolus
c53676.graph_ c0	4.29E-06	-1.1998574	down	hypothetical protein FPSE_02544 [Fusarium pseudograminearum CS3096]

c93918.graph_	1.60E-31	2.01974104	up	hypothetical protein PHAVU_009G255800g [Phaseolus
c79048.graph_	1.08E-194	1.76834875	up	chitinase homologue [Sesbania rostrata]
c91928.graph_	0.0001393	-1.0260395	down	citrate synthase, mitochondrial [Fusarium verticillioides
c88261.graph_ c0	2.19E-06	-1.234369	down	GPR1/FUN34/yaaH family-domain-containing protein [Absidia repens]
c83534.graph_	2.32E-109	1.10960374	up	transcription factor MYB12-like [Cajanus cajan]
c86837.graph_ c0	2.31E-19	-1.9079022	down	uncharacterized protein LOC106777345 isoform X2 [Vigna radiata var. radiata]
c82881.graph_	2.23E-07	-1.2644457	down	unnamed protein product [Fusarium venenatum]
c69304.graph_	7.39E-15	-1.5994505	down	geraniol 8-hydroxylase-like [Cajanus cajan]
c92712.graph_	1.82E-09	-1.3164708	down	probable malate dehydrogenase [Fusarium fujikuroi IMI
c76633.graph_	6.29E-13	-1.2245534	down	alpha-dioxygenase 1 [Cajanus cajan]
c54668.graph_ c0	1.76E-07	-1.2892813	down	putative mitochondrial aldehyde dehydrogenase [Emiliania huxleyi CCMP1516]
c95325.graph_	7.82E-263	6.47320489	up	MLP-like protein 28-like [Glycine max]
c85879.graph_	1.67E-06	-1.2526044	down	3-hydroxyisobutyrate dehydrogenase [Diplodia corticola]
c98462.graph_	3.76E-09	-1.5209112	down	tpr repeat protein oca3 protein [Diplodia corticola]
c94799.graph_ c0	2.49E-26	1.17749369	up	PREDICTED: aldehyde dehydrogenase family 3 member H1 [Glycine max]
c76573.graph_	2.57E-25	1.06558792	up	aquaporin TIP2;1 [Robinia pseudoacacia]
c85411.graph_	4.96E-15	-1.6787123	down	histone h2a [Diplodia corticola]
c58193.graph_	2.01E-09	-1.5369761	down	elongation factor EF-3 [Ectocarpus siliculosus]
c79471.graph_ c0	3.46E-79	2.71621334	up	uncharacterized protein LOC109797106 isoform X2 [Cajanus cajan]
c77869.graph_	2.63E-10	-1.6020743	down	putative dicarboxylic amino acid permease [Diplodia
c78881.graph_ c0	2.35E-07	1.32863225	up	hypothetical protein POPTR_012G000100v3 [Populus trichocarpa]
c97072.graph_	1.59E-10	-1.6293501	down	putative extracellular exo [Diplodia seriata]
c73662.graph_	8.67E-28	1.36970174	up	hypothetical protein PHAVU_008G092800g [Phaseolus
c85570.graph_ c0	4.61E-32	2.68286668	up	PREDICTED: FBD-associated F-box protein At5g22730-like [Glycine max]
c97022.graph_	1.05E-12	-1.811177	down	putative lipase 2 [Diplodia seriata]
c94542.graph_	0	2.46395221	up	late embryogenesis abundant protein 2-like [Cajanus cajan]
c82691.graph_	5.13E-51	2.61950761	up	seed maturation protein [Glycine tabacina]
c37729.graph_	9.25E-15	-1.5176099	down	PREDICTED: protein YLS9 [Glycine max]
c57918.graph_	3.13E-33	-2.904799	down	putative allantoate permease [Diplodia seriata]
c89195.graph_	1.23E-24	-2.4978167	down	nitrate reductase [Fusarium langsethiae]
c91933.graph_	0	1.76480059	up	hypothetical protein GLYMA_20G183700 [Glycine max]
c91323.graph_	8.52E-68	-3.952335	down	hypothetical protein DHEL01_v206890 [Diaporthe
c86551.graph_	3.50E-62	-1.3923584	down	ribonuclease H, partial [Trifolium pratense]
c85232.graph_ c0	2.80E-07	1.12122883	up	PREDICTED: putative GATA transcription factor 22 [Glycine max]
c89905.graph_	2.50E-129	4.21876093	up	germin family protein [Medicago truncatula]
c76156.graph_	1.11E-183	4.28239555	up	51 kDa seed maturation protein precursor [Glycine max]
c80161.graph_	6.03E-30	-2.7264751	down	acetate-CoA ligase [Diaporthe helianthi]
c86893.graph_	6.25E-201	5.24518844	up	PREDICTED: gibberellin 20 oxidase 2-like [Glycine max]
c73735.graph_	2.14E-29	-2.7743564	down	putative riboflavin transporter mch5 [Diaporthe ampelina]
c57661.graph_	4.73E-14	-1.3732895	down	NDR1/HIN1-like protein 10 [Cajanus cajan]
c95562.graph_	2.22E-15	-2.0135011	down	NADPH-dependent FMN reductase [Diaporthe helianthi]

c93496.graph_	6.70E-15	-1.6428984	down	GroES-like protein [Basidiobolus meristosporus CBS
c80433.graph_ c0	6.05E-06	1.12112012	up	omega-6 fatty acid desaturase, endoplasmic reticulum isozyme 1 isoform X2 [Cajanus cajan]
c38187.graph_	5.04E-11	-1.6667021	down	hypothetical protein BKCO1_2200012 [Diplodia corticola]
c92799.graph_	2.81E-12	-1.787148	down	related to kynureninase [Fusarium mangiferae]
c56489.graph_	4.66E-09	-1.4878878	down	gnat family [Diplodia corticola]
c82707.graph_ c0	1.39E-07	1.06850186	up	PREDICTED: uncharacterized protein LOC102666892 [Glycine max]
c54063.graph_ c0	7.28E-54	-3.4159387	down	putative glycosyl transferase family 2 protein [Neofusicoccum parvum UCRNP2]
c89817.graph_	0.0001108	1.0336968	up	ribosomal protein L19 [Ostreococcus lucimarinus
c76909.graph_ c0	7.55E-30	-2.793337	down	hypothetical protein CGGC5_8688 [Colletotrichum gloeosporioides Nara gc5]
c88288.graph_	6.47E-05	1.04133243	up	predicted protein [Hordeum vulgare subsp. vulgare]
c79385.graph_	5.90E-08	1.17241378	up	unknown [Lotus japonicus]
c76603.graph_	1.95E-31	1.29365639	up	Salicylate O-methyltransferase [Glycine soja]
c37789.graph_	1.66E-12	-1.7948484	down	plasma membrane ATPase-1 [Diaporthe helianthi]
c79549.graph_	3.05E-06	-1.2211478	down	calcium calmodulin-dependent protein [Diplodia corticola]
c57051.graph_	1.90E-24	-2.5449496	down	putative mfs multidrug transporter [Diaporthe ampelina]
c76181.graph_	6.14E-50	1.8081506	up	hypothetical protein TSUD_113300 [Trifolium
c95931.graph_	3.61E-19	-2.258356	down	putative 2og-fe oxygenase [Diaporthe ampelina]
c88403.graph_	1.25E-07	-1.3729898	down	hypothetical protein PHAVU_009G185200g [Phaseolus
c87299.graph_	0	4.2510979	up	uncharacterized protein LOC109792496 [Cajanus cajan]
c94377.graph_ c0	1.90E-08	1.31744729	up	PREDICTED: TMV resistance protein N-like isoform X1 [Glycine max]
c73872.graph_ c0	7.29E-11	-1.6525677	down	cell pattern formation-associated protein stuA [Diaporthe helianthi]
c56936.graph_	1.76E-12	-1.8012077	down	Bifunctional protein GAL10 [Diplodia seriata]
c55240.graph_	3.98E-234	4.83806321	up	hypothetical protein GLYMA_06G078700 [Glycine max]
c37760.graph_	7.10E-13	-1.8242195	down	putative secreted protein [Diaporthe ampelina]
c95493.graph_	2.15E-09	-1.017705	down	uncharacterized protein LOC100527699 [Glycine max]
c92006.graph_	3.70E-27	-2.1885312	down	short chain dehydrogenase [Diaporthe helianthi]
c89248.graph_ c1	6.21E-56	2.80547733	up	PREDICTED: polyphenol oxidase A1, chloroplastic-like [Glycine max]
c37916.graph_	3.09E-11	-1.5573337	down	PREDICTED: L-lactate dehydrogenase B-like [Glycine
c37515.graph_	4.63E-14	-1.9171387	down	hypothetical protein UCDDA912_g05724 [Diaporthe
c89352.graph_ c0	3.61E-69	1.09049959	up	PREDICTED: transcriptional corepressor LEUNIG-like isoform X1 [Glycine max]
c38016.graph_	1.15E-20	-2.3250523	down	putative apoptosis-inducing factor 1 [Diplodia seriata]
c96249.graph_	6.70E-14	-1.9094525	down	putative amidase [Hypoxylon sp. EC38]
c92759.graph_	6.37E-20	-1.8781725	down	60S ribosomal protein L3 [Fusarium verticillioides 7600]
c91922.graph_	2.48E-15	-1.9277638	down	hypothetical protein FPOA_08123 [Fusarium poae]
c88923.graph_	3.55E-12	-1.7295899	down	hypothetical protein FPOA_04072 [Fusarium poae]
c76403.graph_ c0	4.90E-20	-1.0429356	down	ATPase 10, plasma membrane-type isoform X2 [Arachis duranensis]
c91851.graph_ c2	2.10E-73	3.619452	up	PREDICTED: 51 kDa seed maturation protein isoform X1 [Glycine max]
c69338.graph_ c0	3.04E-10	-1.3004878	down	PREDICTED: ethylene-responsive transcription factor ERF014 [Vigna angularis]
c94788.graph_ c1	4.24E-10	1.58228926	up	Retrovirus-related Pol polyprotein from transposon TNT 1-94 [Cajanus cajan]

c92958.graph_	3.78E-06	-1.2212714	down	omega-6 fatty acid desaturase [Fusarium langsethiae]
c95312.graph_	1.73E-07	-1.3006481	down	Early nodulin-like protein 2 [Glycine soja]
c84185.graph_ c0	1.51E-14	-1.0791728	down	PREDICTED: leucine-rich repeat receptor-like serine/threonine/tyrosine-protein kinase SOBIR1 [Glycine
c47840.graph_	6.44E-36	-3.0379137	down	amino-acid permease inda1 [Diaporthe helianthi]
c83308.graph_	2.84E-15	-2.0061031	down	glutamine synthetase [Diaporthe helianthi]
c96318.graph_	4.87E-13	-1.8447294	down	cytochrome p450 [Diplodia corticola]
c86109.graph_	1.92E-38	-2.4582807	down	histone H3, partial [Colletotrichum tofieldiae]
c80031.graph_	5.49E-09	-1.4757856	down	probable glutamate decarboxylase [Fusarium proliferatum]
c97280.graph_	5.89E-08	-1.3593366	down	hypothetical protein DHEL01_v201055 [Diaporthe
c92242.graph_	4.30E-15	-1.5623212	down	tubulin alpha chain [Fusarium oxysporum f. sp. lycopersici
c89054.graph_	2.87E-07	-1.3329557	down	heat shock protein 70 [Aureococcus anophagefferens]
c54982.graph_ c0	1.36E-11	-1.7179912	down	RNA recognition domain-containing protein [Diaporthe helianthi]
c98237.graph_	4.62E-11	-1.6702525	down	hypothetical protein DHEL01_v200979 [Diaporthe
c81110.graph_ c1	9.91E-105	3.94031718	up	PREDICTED: uncharacterized protein LOC100813191 [Glycine max]
c71268.graph_	3.61E-24	-2.5312864	down	alpha-amylase 1 [Diaporthe helianthi]
c95613.graph_	1.07E-12	-1.8154408	down	Cytochrome P450 71D10-like protein [Glycine soja]
c81688.graph_ c0	1.05E-07	1.14992242	up	PREDICTED: CBL-interacting serine/threonine-protein kinase 14 [Glycine max]
c53684.graph_ c0	2.02E-16	-2.0841341	down	putative rhamnogalacturonan acetylesterase protein [Neofusicoccum parvum UCRNP2]
c85609.graph_	1.40E-05	-1.1461767	down	putative aspartic endopeptidase pep2 [Diplodia seriata]
c95812.graph_	7.22E-30	-2.7950736	down	clock-controlled gene-15 [Diaporthe helianthi]
c54633.graph_	3.06E-23	-2.4848431	down	heavy metal tolerance protein [Diaporthe helianthi]
c97394.graph_	1.04E-10	-1.6596472	down	Nitrate transporter [Diplodia seriata]
c82578.graph_ c1	1.77E-48	-1.4120717	down	PREDICTED: uncharacterized protein LOC109327790 [Lupinus angustifolius]
c95940.graph_	4.71E-10	-1.600032	down	putative oxalate protein [Neofusicoccum parvum
c96769.graph_	8.07E-13	-1.820056	down	rhamnogalacturonate lyase a [Diplodia corticola]
c83709.graph_ c0	2.28E-93	2.10022771	up	major intrinsic protein (MIP) family transporter [Medicago truncatula]
c91217.graph_	9.96E-06	-1.1641419	down	hypothetical protein FPOA_01318 [Fusarium poae]
c70046.graph_	2.65E-08	-1.0021466	down	uncharacterized protein LOC100527144 [Glycine max]
c89248.graph_ c3	2.48E-28	2.43139114	up	PREDICTED: polyphenol oxidase A1, chloroplastic-like [Vigna angularis]
c93925.graph_	6.83E-104	2.30234	up	PREDICTED: probable protein phosphatase 2C 65
c81563.graph_	9.38E-07	-1.2857271	down	related to neutral amino acid permease [Fusarium
c54203.graph_	1.96E-24	-2.5453412	down	hypothetical protein DHEL01_v212164 [Diaporthe
c38490.graph_	1.01E-15	-2.0382384	down	mfs sugar transporter [Diplodia corticola]
c80334.graph_	3.60E-11	-1.6938488	down	hypothetical protein FAVG1_09175 [Fusarium
c90790.graph_	4.34E-10	1.52483247	up	cysteine-rich receptor-kinase-like protein [Medicago
c38190.graph_	1.34E-12	-1.8026962	down	phosphorus acquisition-controlling protein [Diaporthe
c81574.graph_ c0	1.01E-11	1.34099557	up	PREDICTED: protein IN2-1 homolog B-like [Lupinus angustifolius]
c38524.graph_ c0	1.33E-11	-1.7187661	down	putative methyltransferase [Aspergillus novofumigatus IBT 16806]
c83464.graph_ c0	2.06E-12	1.03575505	up	PREDICTED: ACT domain-containing protein ACR3-like [Glycine max]

c76820.graph_	8.89E-05	-1.0491524	down	hypothetical protein DHEL01_v203734 [Diaporthe
c81394.graph_	1.64E-07	1.16566467	up	PREDICTED: protein DETOXIFICATION 55-like
c84003.graph_	3.57E-09	1.51268092	up	Retrotransposable element Tf2 [Cajanus cajan]
c95532.graph_	7.17E-114	-4.8605337	down	putative multicopper oxidase [Diaporthe ampelina]
c39619.graph_c1	1.77E-13	-1.8724683	down	putative aspartate aminotransferase protein [Phaeoacremonium minimum UCRPA7]
c83039.graph_	1.41E-05	-1.1296991	down	l-xylulose reductase [Diplodia corticola]
c82730.graph_c0	5.57E-08	-1.3610599	down	ubiquitin-conjugating enzyme e2-16 kda, partial [Colletotrichum incanum]
c95233.graph_c0	4.60E-05	-1.0725819	down	hypothetical protein VIGAN_08199700, partial [Vigna angularis var. angularis]
c95519.graph_	1.06E-61	-3.741997	down	Sugar/inositol transporter [Macrophomina phaseolina
c82953.graph_c1	6.64E-10	-1.5909814	down	transmembrane alpha-helix domain-containing protein [Diplodia corticola]
c68962.graph_	1.11E-18	-2.157973	down	sugar transporter [Diplodia corticola]
c88639.graph_c0	7.85E-12	-1.7025037	down	hypothetical protein FPSE_00124 [Fusarium pseudograminearum CS3096]
c96102.graph_c0	4.04E-11	-1.6746391	down	putative isochorismatase hydrolase protein [Neofusicoccum parvum UCRNP2]
c82042.graph_	2.65E-76	-3.7025447	down	putative zinc finger containing protein [Diplodia seriata]
c78119.graph_	5.73E-151	5.34164962	up	seed maturation protein PM31 [Glycine max]
c86837.graph_	9.56E-06	-1.1748839	down	PQ-loop repeat [Trema orientalis]
c83905.graph_	7.43E-17	2.05254908	up	oil body-associated protein 2C-like [Cajanus cajan]
c96030.graph_	1.51E-28	-2.7358707	down	hypothetical protein DHEL01_v209067 [Diaporthe
c61923.graph_c0	3.19E-08	-1.3891838	down	cAMP-independent regulatory protein pac2 [Diaporthe helianthi]
c92268.graph_c0	2.76E-05	-1.1189771	down	AaceriAEL038Cp [Saccharomycetaceae sp. 'Ashbya aceri']
c95987.graph_	7.18E-43	-3.2771488	down	nitrite reductase [Diaporthe helianthi]
c39766.graph_	1.43E-09	-1.5397085	down	hypothetical protein UCDDS831_g08547 [Diplodia
c94793.graph_c0	3.76E-22	1.19467588	up	PREDICTED: subtilisin-like protease Glyma18g48580 isoform X1 [Lupinus angustifolius]
c95103.graph_	9.20E-12	-1.1693485	down	Transposon Ty3-I Gag-Pol polyprotein [Cajanus cajan]
c82226.graph_	4.83E-22	-2.4207806	down	hypothetical protein DHEL01_v209978 [Diaporthe
c64960.graph_	1.75E-08	-1.4535147	down	aggrecan core protein [Diplodia corticola]
c88648.graph_	5.09E-16	-1.9512192	down	putative cmgc mapk protein kinase [Diaporthe ampelina]
c90985.graph_	5.57E-08	-1.3927359	down	uncharacterized LOC100527249 precursor [Glycine max]
c83090.graph_	3.20E-29	2.5887742	up	hypothetical protein TanjilG_27505 [Lupinus
c54965.graph_c0	6.02E-17	1.0351955	up	PREDICTED: uncharacterized protein LOC100804606 [Glycine max]
c93555.graph_	4.70E-32	-1.1173801	down	hypothetical protein PHAVU_002G290800g [Phaseolus
c92014.graph_	3.54E-36	-2.5259126	down	pentose phosphate metabolism-2 [Diaporthe helianthi]
c90468.graph_	7.86E-09	-1.4291751	down	probable PYC2 Pyruvate carboxylase 2 [Fusarium
c87690.graph_c0	2.76E-13	-1.8611723	down	related to cutinase negative acting protein [Fusarium proliferatum]
c55439.graph_	2.44E-16	-2.0794748	down	hypothetical protein PFICI_00156 [Pestalotiopsis fici
c96449.graph_	7.89E-14	-1.8994179	down	hypothetical protein DHEL01_v209474 [Diaporthe
c75231.graph_c0	4.67E-132	2.77541912	up	PREDICTED: 17.9 kDa class II heat shock protein-like [Lupinus angustifolius]
c88279.graph_	1.02E-07	-1.365288	down	Ribosomal protein L5 [Macrophomina phaseolina MS6]
c39866.graph_	6.05E-10	-1.5669415	down	hypothetical protein DHEL01_v209106 [Diaporthe

c88052.graph_	3.83E-05	1.05223269	up	cysteine-rich receptor-kinase-like protein [Medicago
c88447.graph_	4.42E-192	2.05814753	up	seed lipoxygenase [Trifolium pratense]
c69739.graph_	1.27E-37	-3.0963125	down	putative tannase subunit [Diaporthe ampelina]
c90612.graph_ c0	1.27E-10	1.40121548	up	PREDICTED: probable rhamnogalacturonate lyase B isoform X1 [Glycine max]
c68455.graph_	1.81E-12	-1.7921721	down	hypothetical protein UCDDS831_g00989 [Diplodia
c88310.graph_	2.60E-08	-1.4333307	down	putative 60s ribosomal protein l6 [Diaporthe ampelina]
c85390.graph_	4.10E-125	4.47758811	up	Gly m Bd 28K allergen, partial [Glycine max]
c95785.graph_	8.04E-05	-1.0578767	down	uncharacterized protein LOC109817634 [Cajanus cajan]
c38011.graph_	3.08E-67	-3.9460437	down	atpase aaa+ type core protein [Diplodia corticola]
c95727.graph_	1.30E-14	-1.9580435	down	hypothetical protein DHEL01_v205104 [Diaporthe
c38051.graph_	1.63E-08	-1.465094	down	hypothetical protein BK809_0005152 [Diplodia seriata]
c80941.graph_	4.04E-214	3.56791922	up	hypothetical protein PHAVU_002G305200g [Phaseolus
c87806.graph_	2.91E-43	1.33097152	up	PREDICTED: agmatine deiminase [Glycine max]
c37306.graph_	6.59E-12	-1.743937	down	malate dehydrogenase [Diaporthe helianthi]
c95492.graph_	1.31E-22	-2.315368	down	PREDICTED: alpha carbonic anhydrase 7-like [Glycine
c57632.graph_	2.71E-13	-1.8584504	down	calcium P-type ATPase-2 [Diaporthe helianthi]
c79385.graph_	3.30E-07	1.05556536	up	unknown [Lotus japonicus]
c68809.graph_ c0	7.82E-18	-2.1763376	down	hypothetical protein MYCTH_2061639 [Thermothelomyces thermophila ATCC 42464]
c82180.graph_	4.64E-39	2.29026507	up	oleosin [Medicago truncatula]
c92036.graph_	4.73E-08	1.19845947	up	uncharacterized protein LOC109788558 [Cajanus cajan]
c93381.graph_	1.87E-22	1.37838946	up	hypothetical protein GLYMA_07G151900 [Glycine max]
c88447.graph_	1.10E-51	1.28763899	up	seed linoleate 9S-lipoxygenase [Medicago truncatula]
c95713.graph_	8.76E-38	-3.1024136	down	multicopper oxidase [Diplodia corticola]
c86808.graph_	1.34E-66	-3.168679	down	aldehyde dehydrogenase [Fusarium verticillioides 7600]
c80366.graph_	1.29E-29	2.54942186	up	uncharacterized protein LOC109818840 [Cajanus cajan]
c61452.graph_	1.61E-26	-2.6463259	down	hypothetical protein DHEL01_v208629 [Diaporthe
c37795.graph_	2.41E-07	-1.3245601	down	hypothetical protein PHAVU_006G103800g [Phaseolus
c89142.graph_	9.91E-05	1.04748043	up	hypothetical protein glysoja_039404 [Glycine soja]
c71965.graph_ c0	2.22E-10	-1.6293751	down	Proteinase inhibitor I13 potato inhibitor I [Macrophomina phaseolina MS6]
c40188.graph_ c0	4.27E-12	-1.7609331	down	hypothetical protein VFPPC_08845 [Pochonia chlamydosporia 170]
c82986.graph_ c0	2.37E-06	1.20728043	up	Bifunctional monodehydroascorbate reductase and carbonic anhydrase nectarin-3 [Glycine soja]
c54456.graph_	1.83E-15	1.29860445	up	extracellular ribonuclease LE-like [Cajanus cajan]
c58306.graph_	6.24E-17	-2.1185141	down	NADPH dehydrogenase [Diaporthe helianthi]
c92388.graph_	3.77E-12	-1.3838175	down	S-Adenosyl homocysteine hydrolase [Ectocarpus
c82538.graph_	1.11E-15	-2.0348211	down	Hexose transporter HXT13 [Valsa mali]
c81623.graph_ c1	1.82E-05	-1.0007904	down	PREDICTED: UDP-glucose 6-dehydrogenase 1-like [Glycine max]
c37830.graph_	6.44E-20	-1.0913647	down	hypothetical protein PHAVU_002G300200g [Phaseolus
c61524.graph_	8.55E-09	-1.4512014	down	hypothetical protein BKCO1_8100018 [Diplodia corticola]
c94822.graph_	1.52E-06	1.0982514	up	hypothetical protein LR48_Vigan406s006600 [Vigna
c75932.graph_	1.56E-09	-1.1650981	down	uncharacterized protein LOC100780763 [Glycine max]
c73491.graph_	1.86E-95	3.13247966	up	Em protein [Robinia pseudoacacia]
c91516.graph_	7.09E-06	-1.1805941	down	hypothetical protein FPOA_01235 [Fusarium poae]

c86642.graph_	7.60E-42	-3.1364665	down	beta-1,3-glucanosyltransferase [Diaporthe helianthi]
c90042.graph_	3.81E-08	-1.2420738	down	unnamed protein product [Fusarium sp. FIESC_5 CS3069]
c73344.graph_ c0	0.0001378	1.01323193	up	late embryogenesis abundant (LEA)-like protein [Medicago truncatula]
c39775.graph_	2.25E-09	-1.5107626	down	hypothetical protein DHEL01_v207076 [Diaporthe [illegible]
c82890.graph_	0.0001425	-1.0247773	down	50S ribosomal protein L26e [Fusarium verticillioides [illegible]
c71205.graph_	5.07E-05	-1.0678268	down	putative Ddr48p [Diplodia seriata]
c37899.graph_ c0	0.0001374	-1.0059909	down	F-type H+-transporting ATPase subunit alpha [Fistulifera solaris]
c76688.graph_	2.13E-15	1.19830129	up	hypothetical protein PHAVU_006G075700g [Phaseolus [illegible]
c84429.graph_	2.35E-30	1.72757268	up	cation/calcium exchanger 1-like [Cajanus cajan]
c39701.graph_	8.91E-15	-1.9721936	down	putative fatty acid oxygenase [Diplodia seriata]
c37761.graph_	1.40E-16	-2.0440971	down	putative amino acid permease [Diplodia seriata]
c86908.graph_	2.64E-11	-1.4422032	down	hypothetical protein glysoja_048907, partial [Glycine soja]
c89724.graph_	1.73E-80	3.30590052	up	hypothetical protein glysoja_013880 [Glycine soja]
c79298.graph_	3.31E-51	-3.5028375	down	hypothetical protein DHEL01_v206493 [Diaporthe [illegible]
c95560.graph_ c0	0.0001003	-1.0367866	down	hypothetical protein EMIHUDRAFT_435784 [Emiliania huxleyi CCMP1516]
c94464.graph_	1.56E-15	1.80281678	up	hypothetical protein TanjilG_29652 [Lupinus [illegible]
c92843.graph_	1.27E-37	1.21837732	up	hypothetical protein GLYMA_10G146100 [Glycine max]
c37763.graph_ c0	5.09E-25	-2.5751216	down	hypothetical protein MYCTH_2110682 [Thermothelomyces thermophila ATCC 42464]
c38130.graph_	6.80E-06	-1.0476049	down	C2H2-type zinc finger domain-containing protein [Glycine [illegible]
c80836.graph_	4.04E-11	-1.6954941	down	putative dioxygenase [Colletotrichum sublineola]
c90242.graph_	7.11E-115	3.83551915	up	Enzymatic polyprotein [Cajanus cajan]
c88350.graph_ c0	5.36E-16	-1.7016399	down	tyrosine 3-monooxygenase tryptophan 5-monooxygenase activation protein [Fusarium langsethiae]
c68022.graph_	6.31E-10	-1.5647458	down	hypothetical protein DHEL01_v203398 [Diaporthe [illegible]
c68717.graph_	2.05E-24	-2.5296217	down	putative hexose transporter protein [Diplodia seriata]
c94255.graph_	7.54E-194	1.30119287	up	3-ketoacyl-CoA thiolase 2, peroxisomal [Cajanus cajan]
c77215.graph_ c1	1.20E-09	-1.5364368	down	cAMP-dependent protein kinase regulatory subunit [Diaporthe helianthi]
c90756.graph_	0	2.80610332	up	alpha' subunit of beta-conglycinin, partial [Glycine [illegible]
c84470.graph_	6.11E-05	-1.0703928	down	atpase aaa+ type core protein [Diplodia corticola]
c95514.graph_	1.95E-22	-1.7122198	down	flavonol synthase [Glycine max]
c90345.graph_	3.74E-11	1.6968244	up	uncharacterized protein LOC109814814 [Cajanus cajan]
c86583.graph_	7.92E-09	-1.1283327	down	uncharacterized protein At1g66480-like [Cajanus cajan]
c86637.graph_	7.58E-06	-1.1821648	down	unnamed protein product [Fusarium graminearum]
c83632.graph_ c0	1.66E-08	-1.1664951	down	PREDICTED: probable L-type lectin-domain containing receptor kinase S.5 [Glycine max]
c40212.graph_ c0	1.11E-10	-1.6366828	down	glucose-methanol-choline oxidoreductase [Arthroderma otae CBS 113480]
c37861.graph_	5.04E-92	1.84011369	up	seed maturation protein PM35 [Glycine max]
c95766.graph_	1.18E-14	-1.9609515	down	glycoside hydrolase family 61 protein [Diplodia corticola]
c89854.graph_ c1	0.0002152	1.00060565	up	PREDICTED: uncharacterized protein LOC105852030 [Cicer arietinum]
c91740.graph_	3.99E-06	-1.2101694	down	succinate dehydrogenase [Fusarium langsethiae]
c89912.graph_ c0	9.32E-20	1.13885729	up	DEAD-box ATP-dependent RNA helicase 52C [Vigna radiata var. radiata]
c82044.graph_	9.24E-20	1.0372083	up	unknown [Lotus japonicus]

c95213.graph_c0	9.18E-09	1.09616406	up	Retrovirus-related Pol polyprotein from transposon TNT 1-94, partial [Cajanus cajan]
c90925.graph_c0	2.32E-13	-1.8258059	down	NADPH-ferrihemoprotein reductase [Fusarium verticillioides 7600]
c38465.graph_c0	2.79E-27	-2.679782	down	putative glycoside hydrolase family 16 protein [Diaporthe ampelina]
c78289.graph_	1.13E-20	-2.3455187	down	Lactose permease [Valsa mali]
c89488.graph_c1	5.21E-25	1.02085256	up	soyasapogenol B glucuronide galactosyltransferase [Vigna radiata var. radiata]
c84211.graph_	0.0001939	-1.0016251	down	related to allantoate permease [Fusarium mangiferae]
c78735.graph_	5.30E-32	-1.4152233	down	mitotic cyclin [Sesbania rostrata]
c67703.graph_	2.44E-99	-4.3707892	down	lactose permease protein [Diplodia corticola]
c37850.graph_	6.00E-68	-1.3527421	down	glucan endo-1,3-beta-glucosidase, basic isoform [Cajanus
c94485.graph_	1.09E-17	1.60401454	up	hypothetical protein GLYMA_15G254300 [Glycine max]
c80361.graph_c0	2.91E-26	-2.2321861	down	putative glutamine synthetase protein [Neofusicoccum parvum UCRNP2]
c89685.graph_	4.39E-17	1.22349462	up	putative protein phosphatase 2C 8 [Cajanus cajan]
c74990.graph_	2.72E-19	-2.2654433	down	MFS general substrate transporter [Aspergillus
c87225.graph_c0	1.37E-08	-1.4706517	down	hypothetical protein FPSE_11617 [Fusarium pseudograminearum CS3096]
c38822.graph_	6.12E-09	-1.466661	down	transcriptional activator hac1 [Diaporthe helianthi]
c89582.graph_	1.36E-11	-1.1687073	down	uncharacterized protein LOC109793947 [Cajanus cajan]
c88270.graph_c0	2.58E-36	-2.8672781	down	putative glycosyl hydrolase family 7 protein [Neofusicoccum parvum UCRNP2]
c94768.graph_	4.81E-16	-1.5081421	down	unnamed protein product [Fusarium venenatum]
c82859.graph_c0	1.54E-20	-2.1688505	down	putative aspergillopepsin-2 protein [Neofusicoccum parvum UCRNP2]
c80800.graph_	2.82E-35	-1.0292653	down	hypothetical protein GLYMA_15G072400 [Glycine max]
c90758.graph_c0	4.91E-08	-1.4046338	down	hypothetical protein FOXG_02474 [Fusarium oxysporum f. sp. lycopersici 4287]
c82748.graph_	5.84E-15	1.4532469	up	uncharacterized protein LOC109811309 [Cajanus cajan]
c87838.graph_	9.77E-226	4.3105393	up	seed biotin-containing protein SBP65-like [Cajanus cajan]
c93468.graph_c0	7.39E-06	-1.1751417	down	related to synaptic vesicle transporter SV2 (major facilitator superfamily) [Fusarium proliferatum]
c97161.graph_	5.45E-10	-1.5718538	down	putative solute carrier family 35 member e3 [Diaporthe
c55680.graph_c0	4.56E-13	-1.8399514	down	putative glucose-6-phosphate 1-dehydrogenase [Diaporthe ampelina]
c78582.graph_	1.46E-109	-4.368184	down	putative btb poz-like protein [Diplodia seriata]
c96049.graph_	3.28E-11	-1.6953146	down	c6 zinc finger domain containing protein [Diplodia
c85642.graph_c0	3.26E-06	-1.2208743	down	hypothetical protein FPSE_03661 [Fusarium pseudograminearum CS3096]
c84497.graph_c0	1.13E-09	-1.0070112	down	Retrovirus-related Pol polyprotein from transposon TNT 1-94 [Cajanus cajan]
c95402.graph_	1.08E-22	-2.4550381	down	hypothetical protein PFICI_00156 [Pestalotiopsis fici
c55966.graph_	1.02E-24	-2.55834	down	MFS quinate transporter [Diaporthe helianthi]
c80538.graph_	2.69E-18	-1.3452056	down	uncharacterized protein LOC109804418 [Cajanus cajan]
c96381.graph_	1.92E-11	-1.7043869	down	NHL repeat-containing protein [Aspergillus steynii IBT
c95650.graph_	3.36E-21	-1.2763477	down	--
c69825.graph_	5.36E-16	-2.0057803	down	ammonium transporter [Diplodia corticola]
c94365.graph_	2.54E-22	1.79719915	up	sodium/hydrogen exchanger 4 [Cajanus cajan]

c82640.graph_ c1	2.93E-13	-1.7546034	down	eukaryotic translation initiation factor eIF-1 [Fusarium oxysporum f. sp. cubense tropical race 4 54006]
c78159.graph_ c0	1.48E-33	-2.9435032	down	high-affinity methionine permease [Colletotrichum orchidophilum]
c94980.graph_ c1	7.77E-12	-1.0596088	down	PREDICTED: uncharacterized protein LOC108336731 [Vigna angularis]
c37588.graph_	1.03E-08	-1.1408485	down	small ubiquitin-related modifier 1-like [Cajanus cajan]
c96411.graph_	2.94E-14	-1.9311012	down	putative ral amino-acid permease gap1 [Diaporthe
c79812.graph_ c0	1.93E-25	1.61730778	up	PREDICTED: uncharacterized protein LOC102665184 [Glycine max]
c55555.graph_	4.46E-14	-1.4462969	down	uncharacterized oxidoreductase At4g09670 [Arachis
c95623.graph_	2.27E-25	-2.5442388	down	putative pectinesterase A [Diplodia seriata]
c38126.graph_	3.86E-18	-2.1954836	down	hypothetical protein DHEL01_v208702 [Diaporthe
c92390.graph_	9.27E-34	1.9428679	up	Low-temperature-induced 65 kDa protein [Glycine soja]
c79394.graph_ c0	1.41E-17	-2.0239926	down	BAG family molecular chaperone regulator 5, mitochondrial [Glycine soja]
c95244.graph_	5.18E-09	-1.4790913	down	C2H2-type zinc finger protein [Glycine max]
c55806.graph_ c0	6.55E-08	-1.4075873	down	putative calcium calmodulin-dependent protein kinase [Diplodia seriata]
c81458.graph_	1.07E-17	-2.0885503	down	ubiquitin carboxyl-terminal hydrolase 2 [Diplodia
c65214.graph_	3.21E-11	-1.6847697	down	hypothetical protein DHEL01_v201142 [Diaporthe
c38729.graph_	4.91E-16	-2.0590644	down	hypothetical protein DHEL01_v200797 [Diaporthe
c53750.graph_ c0	2.15E-09	-1.5131135	down	putative short chain dehydrogenase reductase [Diaporthe ampelina]
c88217.graph_ c0	1.28E-06	-1.2745033	down	putative glutamate dehydrogenase (NADP+) [Fusarium fujikuroi]
c73316.graph_	1.06E-68	-1.4837271	down	PREDICTED: myb-related protein Myb4-like [Glycine
c80054.graph_	6.10E-64	-1.6579538	down	hypothetical protein GLYMA_18G220600 [Glycine max]
c55893.graph_	2.93E-21	-2.3780819	down	hypothetical protein VP1G_00949 [Valsa mali var. pyri]
c55847.graph_ c0	2.42E-20	2.31584015	up	uncharacterized GPI-anchored protein At3g06035-like [Cajanus cajan]
c82654.graph_	0	3.81248531	up	embryonic abundant-like protein [Medicago truncatula]
c84700.graph_	1.35E-11	-1.7154912	down	cyclosporin-resistant-1 [Diaporthe helianthi]
c84485.graph_	5.31E-22	-2.4098786	down	hypothetical protein DHEL01_v211129 [Diaporthe
c80545.graph_ c0	3.86E-06	1.2190514	up	PREDICTED: flavonol synthase/flavanone 3-hydroxylase-like [Glycine max]
c90279.graph_	1.53E-08	-1.4364483	down	hypothetical protein FNYG_14977 [Fusarium nygamai]
c84481.graph_	3.27E-27	-2.6412065	down	pectin lyase [Fusarium langsethiae]
c37705.graph_ c0	6.52E-08	-1.4058405	down	LOW QUALITY PROTEIN: GDSL esterase/lipase 1-like [Cajanus cajan]
c95684.graph_	2.19E-14	-1.9408277	down	kinase-like protein [Stagonospora sp. SRC1lsM3a]
c87934.graph_	8.25E-09	1.48941771	up	probable endopeptidase K [Fusarium fujikuroi IMI 58289]
c82135.graph_	6.46E-22	-2.3663409	down	hypothetical protein UCDDS831_g01262 [Diplodia
c88603.graph_	2.88E-63	1.66581096	up	Vacuolar cation/proton exchanger 3 [Glycine soja]
c95201.graph_ c0	4.07E-66	-1.4617844	down	retrotransposon protein, putative, Ty1-copia sub-class [Oryza sativa Japonica Group]
c94180.graph_	1.77E-08	1.42363691	up	predicted protein [Hordeum vulgare subsp. vulgare]
c90056.graph_	4.19E-37	1.25386358	up	uncharacterized protein LOC109797678 [Cajanus cajan]
c68228.graph_	5.16E-12	-1.7517367	down	putative serine threonine protein kinase [Diplodia seriata]
c37889.graph_	4.21E-07	-1.2748545	down	hypothetical protein PHAVU_003G218900g [Phaseolus

c59651.graph_c0	1.59E-22	-2.4466305	down	nucleoside-triphosphatase, RNA helicase [Pseudoloma neurophilia]
c89277.graph_	1.81E-05	-1.1365559	down	patatin-like protein 2 [Cajanus cajan]
c95136.graph_c0	7.57E-18	1.36457959	up	PREDICTED: microtubule-associated protein futsch isoform X6 [Glycine max]
c89007.graph_c0	8.54E-12	-1.6427518	down	probable ribosomal protein L9.e.c14 [Fusarium fujikuroi IMI 58289]
c95345.graph_c0	1.22E-12	-1.803865	down	hypothetical protein P174DRAFT_442171 [Aspergillus novofumigatus IBT 16806]
c95827.graph_c0	9.95E-88	-4.3994213	down	hypothetical protein MPH_13779 [Macrophomina phaseolina MS6]
c38636.graph_	4.70E-10	-1.5784013	down	putative cyclin-like protein [Diaporthe ampelina]
c80223.graph_	6.35E-05	-1.0721926	down	hypothetical protein FGSG_05999 [Fusarium graminearum [illegible]
c92078.graph_c3	0	4.17049997	up	group 3 late embryogenesis abundant protein [Phaseolus vulgaris]
c93803.graph_c0	2.77E-69	-2.8876425	down	putative alcohol oxidase protein [Neofusicoccum parvum UCRNP2]
c90517.graph_	4.24E-06	1.21351439	up	hypothetical protein GLYMA_06G085100 [Glycine max]
c88447.graph_	1.95E-147	1.69351799	up	probable linoleate 9S-lipoxygenase 5 [Cajanus cajan]
c83281.graph_c1	2.97E-08	-1.4367757	down	PREDICTED: glutamate synthase [NADH], amyloplastic-like isoform X4 [Glycine max]
c87124.graph_c0	9.13E-08	-1.0184639	down	putative SWI/SNF-related matrix-associated actin-dependent regulator of chromatin subfamily A member 3-like 1 [Cajanus cajan]
c92523.graph_	2.23E-66	1.15056225	up	hypothetical protein PHAVU_008G090600g [Phaseolus [illegible]
c84367.graph_	8.25E-08	1.22754528	up	uncharacterized protein LOC109813600 [Cajanus cajan]
c92705.graph_	8.48E-06	-1.1308835	down	linoleate 13S-lipoxygenase 3-1, chloroplastic-like [Cajanus [illegible]
c95191.graph_	5.33E-07	1.30382021	up	hypothetical protein TSUD_56070 [Trifolium [illegible]
c95843.graph_	4.93E-12	-1.7559398	down	putative mfs sugar transporter [Diplodia seriata]
c67762.graph_	7.21E-09	-1.4587314	down	general substrate transporter [Diplodia corticola]
c90415.graph_	2.15E-14	1.03722987	up	pathogen-related protein-like [Cajanus cajan]
c76654.graph_	1.56E-40	-1.1151839	down	beta-galactosidase 13-like [Vigna radiata var. radiata]
c95142.graph_c0	1.06E-06	1.26639934	up	Retrovirus-related Pol polyprotein from transposon TNT 1-94 [Cajanus cajan]
c87661.graph_	1.30E-07	-1.0118893	down	trihelix transcription factor PTL [Cajanus cajan]
c93680.graph_c0	4.13E-41	1.60469014	up	PREDICTED: probable N-acetyltransferase HLS1 [Vigna angularis]
c86501.graph_	1.53E-05	-1.1409122	down	hypothetical protein NA56DRAFT_683555 [Pezoloma [illegible]
c95235.graph_c0	9.23E-15	1.59313138	up	PREDICTED: uncharacterized protein LOC101495759 [Cicer arietinum]
c93268.graph_	6.33E-14	1.05273143	up	hypothetical protein glysoja_034424 [Glycine soja]
c90982.graph_c0	1.27E-63	2.94432654	up	uncharacterized protein LOC106756243 [Vigna radiata var. radiata]
c89059.graph_	9.23E-07	-1.120089	down	polyadenylate-binding protein 1-B [Achlya hypogyna]
c95571.graph_	3.44E-30	-2.807223	down	putative acid proteinase [Diaporthe ampelina]
c69835.graph_	1.17E-08	-1.0537157	down	hypothetical protein TanjilG_14592 [Lupinus [illegible]
c95207.graph_	1.78E-136	2.34568552	up	disease resistance protein RPM1-like [Arachis duranensis]
c81219.graph_	2.95E-12	-1.6788434	down	40S ribosomal protein S27 [Colletotrichum tofieldiae]
c87851.graph_c0	3.65E-08	-1.2790411	down	putative transcription factor bHLH041 isoform X1 [Cajanus cajan]
c82676.graph_	1.30E-09	-1.5263939	down	EF-hand [Aureobasidium namibiae CBS 147.97]
c69203.graph_	2.16E-20	-1.3905468	down	glutathione S-transferase U17-like [Cajanus cajan]

c89116.graph_ c0	5.05E-22	1.87611862	up	PREDICTED: putative 12-oxophytodienoate reductase 11 isoform X3 [Lupinus angustifolius]
c83486.graph_	2.43E-30	-1.1979749	down	hypothetical protein CFP56_05604 [Quercus suber]
c89941.graph_	2.45E-16	1.05910618	up	hypothetical protein GLYMA_09G279000 [Glycine max]
c73882.graph_	9.57E-05	-1.0023636	down	MYB transcription factor MYB84 [Glycine max]
c81937.graph_	5.63E-06	1.1975877	up	hypothetical protein glysoja_006307, partial [Glycine soja]
c82102.graph_	3.31E-06	1.21337003	up	beta-amyrin 11-oxidase [Glycyrrhiza uralensis]
c76778.graph_	3.23E-06	1.19957926	up	ALA-interacting subunit 3-like [Cajanus cajan]
c37692.graph_ c0	4.12E-23	-2.4771596	down	NAD-dependent epimerase/dehydratase, putative [Talaromyces marneffei ATCC 18224]
c78629.graph_	9.69E-10	-1.358955	down	putative WRKY transcription factor 51 [Cajanus cajan]
c58153.graph_	9.38E-12	-1.7382921	down	h+ nucleoside cotransporter [Diplodia corticola]
c96316.graph_	3.93E-12	-1.763959	down	putative endoglucanase d precursor [Diplodia seriata]
c77566.graph_	7.36E-35	-2.7667538	down	c2h2 transcription factor [Diplodia corticola]
c87025.graph_	5.90E-09	-1.497636	down	unnamed protein product [Fusarium venenatum]
c70289.graph_	1.93E-19	-2.2606345	down	hypothetical protein BK809_0007542 [Diplodia seriata]
c95653.graph_	3.82E-29	-2.7436698	down	hypothetical protein DHEL01_v207178 [Diaporthe
c82346.graph_	4.32E-17	1.16126499	up	germin-like protein subfamily 1 member 17 [Cajanus
c58153.graph_	5.88E-10	-1.5959712	down	putative h+ nucleoside cotransporter [Diplodia seriata]
c97279.graph_ c0	2.24E-07	-1.3188763	down	fungal specific transcription factor domain-containing protein [Diplodia corticola]
c85468.graph_ c0	3.88E-103	-1.2810101	down	PREDICTED: universal stress protein A-like protein [Lupinus angustifolius]
c89118.graph_	2.17E-15	1.01547998	up	hypothetical protein glysoja_033459 [Glycine soja]
c91060.graph_	0.0001881	-1.0075505	down	fructose-bisphosphate aldolase [Fusarium graminearum
c73296.graph_	2.16E-27	-2.0709461	down	putative lipid-transfer protein DIR1 [Cajanus cajan]
c77233.graph_	4.74E-181	1.91937682	up	double-headed trypsin inhibitor [Phaseolus lunatus]
c74422.graph_	1.62E-74	-3.7532265	down	polyketide synthase [Diplodia corticola]
c76694.graph_	4.73E-11	-1.4230327	down	PREDICTED: putative nuclease HARBI1 [Vigna
c90852.graph_	8.18E-08	-1.3251093	down	enolase [Fusarium langsethiae]
c91803.graph_	2.78E-74	1.8747411	up	Secologanin synthase [Glycine soja]
c94240.graph_	9.18E-10	-1.2922888	down	probable aconitase [Fusarium proliferatum]
c95819.graph_	5.12E-35	-3.0006537	down	sulfate permease [Diaporthe helianthi]
c96306.graph_	5.98E-12	-1.7601365	down	glycosyltransferase family 2 protein [Diplodia corticola]
c81209.graph_	1.17E-18	-1.0138242	down	protein EXORDIUM-like 2 [Arachis ipaensis]
c74367.graph_ c1	4.35E-09	-1.5069903	down	putative pre-mrna splicing factor protein [Neofusicoccum parvum UCRNP2]
c74291.graph_	2.20E-46	-3.3857358	down	2-keto-4-pentenoate hydratase [Diaporthe helianthi]
c86375.graph_ c0	9.04E-13	1.06237551	up	PREDICTED: putative gamma-glutamylcyclotransferase At3g02910 [Glycine max]
c58116.graph_	4.21E-07	-1.3174342	down	Phosphoesterase [Macrophomina phaseolina MS6]
c82285.graph_	3.44E-30	-1.9176961	down	PREDICTED: glutamate decarboxylase 1-like [Glycine
c91468.graph_	2.25E-45	1.31659294	up	hypothetical protein glysoja_002605 [Glycine soja]
c76051.graph_	1.31E-30	-2.8258172	down	H+/nucleoside cotransporter [Diaporthe helianthi]
c88937.graph_	1.40E-10	-1.6507677	down	hypothetical protein FPOA_10356 [Fusarium poae]
c88362.graph_	8.61E-15	-1.8672252	down	unnamed protein product [Fusarium sp. FIESC_5 CS3069]
c84114.graph_ c0	8.80E-21	-1.7209838	down	PREDICTED: 18.2 kDa class I heat shock protein-like [Nicotiana attenuata]
c85689.graph_	7.58E-304	3.72602068	up	metallothionein-II protein [Glycine max]

c96073.graph_	7.27E-13	-1.8311122	down	putative cdc50 family protein [Diaporthe ampelina]
c94191.graph_	4.22E-23	1.00685884	up	Para-aminobenzoate synthase [Glycine soja]
c81829.graph_ c1	1.54E-07	1.35721313	up	probable disease resistance protein At1g12280 [Vigna radiata var. radiata]
c82570.graph_	3.42E-12	-1.7815755	down	elongation factor 3 [Diplodia corticola]
c54876.graph_	0.0001357	-1.0040181	down	40S ribosomal protein S4 putative [Albugo laibachii Nc14]
c37734.graph_	4.68E-15	-1.9920527	down	putative proline-rich protein [Neofusicoccum parvum
c76925.graph_	1.20E-34	-2.9860556	down	hypothetical protein UCDDA912_g05228 [Diaporthe
c84515.graph_ c0	9.17E-07	-1.2884915	down	hypothetical protein FPSE_00523 [Fusarium pseudograminearum CS3096]
c76489.graph_	2.16E-06	-1.2431636	down	alcohol dehydrogenase [Cajanus cajan]
c92782.graph_	1.96E-09	1.44806095	up	hypothetical protein GLYMA_02G002200 [Glycine max]
c54296.graph_	4.89E-10	-1.5998125	down	Histone H1/H5 [Macrophomina phaseolina MS6]
c80282.graph_ c0	2.95E-12	-1.6522276	down	PREDICTED: uncharacterized protein LOC100803389 [Glycine max]
c96125.graph_	5.16E-14	-1.9127142	down	hypothetical protein ALT_4866 [Aspergillus lentulus]
c56179.graph_	8.63E-12	-1.749898	down	hypothetical protein BK809_0007407 [Diplodia seriata]
c86759.graph_	2.08E-20	-1.9098563	down	hypothetical protein CDD82_6266 [Ophiocordyceps
c95030.graph_	9.19E-175	2.63259448	up	PREDICTED: 1-Cys peroxiredoxin [Glycine max]
c82444.graph_	9.19E-16	-2.0394955	down	hypothetical protein DHEL01_v208490 [Diaporthe
c92020.graph_ c6	2.68E-09	-1.5030398	down	putative flavoprotein involved in k+ transport [Diaporthe ampelina]
c94656.graph_	9.04E-123	1.67088607	up	glycinin G1 precursor [Glycine max]
c89765.graph_ c0	2.85E-15	-1.5823159	down	glyceraldehyde-3-phosphate dehydrogenase [Fusarium verticillioides 7600]
c64715.graph_	5.76E-15	-1.9840477	down	hypothetical protein DHEL01_v202533 [Diaporthe
c82154.graph_ c0	2.65E-05	-1.1202184	down	V-type proton ATPase proteolipid subunit [Fusarium oxysporum f. sp. lycopersici 4287]
c68815.graph_ c0	7.89E-14	1.89959425	up	PREDICTED: uncharacterized protein LOC102660776 [Glycine max]
c36772.graph_	5.73E-12	-1.7494872	down	udp-glucose 4-epimerase [Diaporthe helianthi]
c67694.graph_ c0	2.13E-71	-4.058354	down	major facilitator superfamily domain-containing protein [Pseudomassariella vexata]
c67561.graph_	3.04E-14	-1.0621249	down	hypothetical protein glysoja_008433 [Glycine soja]
c84880.graph_	3.33E-30	2.70021599	up	subtilisin inhibitor 1-like protein [Trifolium pratense]
c90829.graph_	7.22E-191	4.65871903	up	Gag-pol polyprotein, putative [Theobroma cacao]
c67487.graph_ c0	4.73E-13	-1.8472584	down	hypothetical protein MPH_10939 [Macrophomina phaseolina MS6]
c84662.graph_	3.06E-13	-1.8533098	down	putative endoglucanase-4 precursor [Diplodia seriata]
c76819.graph_	5.30E-10	1.1869302	up	uncharacterized protein LOC112015919 [Quercus suber]
c70252.graph_	5.82E-05	1.02649055	up	hypothetical protein PHAVU_007G057800g [Phaseolus
c84644.graph_	0	3.93709902	up	PREDICTED: peroxygenase 1-like [Lupinus angustifolius]
c55590.graph_	7.58E-17	-2.1130767	down	putative MFS-type transporter PB1E7.08c [Valsa mali]
c91797.graph_	3.94E-38	-2.7111724	down	probable nitrate transport protein crnA [Fusarium
c80075.graph_	1.36E-13	-1.8813715	down	phthalate transporter [Diaporthe helianthi]
c89167.graph_ c1	3.42E-17	-2.0469744	down	phosphoenolpyruvate carboxykinase (ATP) [Diaporthe helianthi]
c75692.graph_	5.68E-06	1.19714118	up	PREDICTED: protein CROWDED NUCLEI 4-like
c76323.graph_ c0	1.39E-54	2.04324979	up	PREDICTED: 1-aminocyclopropane-1-carboxylate synthase 7-like [Glycine max]

c72960.graph_c0	2.67E-12	-1.0331313	down	nematode resistance protein-like HSPRO2 [Vigna radiata var. radiata]
c71678.graph_	1.25E-11	-1.1701615	down	Major allergen Pru av 1 [Glycine soja]
c70434.graph_c0	6.29E-25	-1.0754464	down	DNA damage-repair/toleration protein DRT100-like [Cajanus cajan]
c85087.graph_	6.19E-18	-1.5185526	down	PREDICTED: protein ECERIFERUM 1-like [Glycine
c79771.graph_c0	3.41E-06	-1.1545122	down	PREDICTED: solute carrier family 25 member 44-like isoform X2 [Glycine max]
c80224.graph_c0	2.98E-19	1.32152529	up	probable 2-oxoglutarate-dependent dioxygenase AOP1 [Cajanus cajan]
c76644.graph_	1.33E-68	-2.1775602	down	uncharacterized protein LOC109807675 [Cajanus cajan]
c57159.graph_	1.50E-11	-1.7122128	down	hypothetical protein DHEL01_v201049 [Diaporthe
c55844.graph_	1.21E-33	-2.9473462	down	hypothetical protein DHEL01_v207595 [Diaporthe
c80213.graph_	1.11E-65	-1.6657896	down	putative glutathione S-transferase parA, partial [Cajanus
c76112.graph_c0	1.62E-08	-1.052608	down	L-type lectin-domain containing receptor kinase VII.1-like [Cajanus cajan]
c37574.graph_	3.65E-11	-1.6854046	down	hypothetical protein UCDDS831_g03274 [Diplodia
c84862.graph_	1.58E-08	-1.4513028	down	hypothetical protein FPOA_00687 [Fusarium poae]
c95157.graph_c0	2.00E-26	2.32170444	up	Retrovirus-related Pol polyprotein from transposon TNT 1-94 [Cajanus cajan]
c83026.graph_	1.76E-06	-1.16696	down	unnamed protein product [Fusarium graminearum]
c89413.graph_c0	1.54E-24	1.02015691	up	G-type lectin S-receptor-like serine/threonine-protein kinase [Glycine soja]
c34524.graph_	6.68E-09	-1.4626795	down	carboxypeptidase Y precursor [Diaporthe helianthi]
c92624.graph_c1	5.23E-22	-2.1075047	down	S-adenosylmethionine synthase [Fusarium oxysporum f. sp. lycopersici 4287]
c96109.graph_c0	9.52E-10	-1.5796092	down	heat shock protein, HSP70, hsc70 [Thalassiosira pseudonana CCMP1335]
c82693.graph_	1.73E-11	1.49182426	up	hypothetical protein KK1_046207 [Cajanus cajan]
c75208.graph_	4.90E-11	-1.6760593	down	hypothetical protein BKCO1_5600048 [Diplodia corticola]
c69219.graph_	1.64E-50	-3.5076016	down	metacaspase [Diaporthe helianthi]
c90753.graph_c0	6.14E-42	1.32765108	up	PREDICTED: protein STRICTOSIDINE SYNTHASE-LIKE 5-like [Cicer arietinum]
c68005.graph_	5.12E-26	-2.6062867	down	siderochrome-iron transporter [Diplodia corticola]
c80232.graph_	0.0001183	-1.0363668	down	unnamed protein product [Fusarium venenatum]
c83761.graph_c0	2.53E-11	-1.2004735	down	PREDICTED: uncharacterized protein LOC108323995 [Vigna angularis]
c80331.graph_	1.49E-21	-2.3073482	down	putative c2h2 transcription factor [Diplodia seriata]
c92606.graph_	9.58E-19	-2.0533014	down	hypothetical protein FNYG_03084 [Fusarium nygamai]
c86616.graph_	1.67E-29	-2.7227147	down	mfs peptide transporter [Diplodia corticola]
c88542.graph_c0	1.73E-15	-1.0891672	down	nucleobase-ascorbate transporter-like protein [Medicago truncatula]
c89911.graph_	0.0001183	1.02927082	up	probable N-acetyltransferase HLS1-like [Cajanus cajan]
c79583.graph_c0	9.05E-76	1.34123578	up	protein SULFUR DEFICIENCY-INDUCED 1-like isoform X1 [Cajanus cajan]
c95166.graph_	7.82E-68	3.28340953	up	hypothetical protein L195_g016306, partial [Trifolium
c38838.graph_	1.64E-05	1.12693487	up	unnamed protein product [Fusarium venenatum]
c81186.graph_c0	1.08E-23	-2.1235645	down	PREDICTED: spermidine hydroxycinnamoyl transferase-like [Lupinus angustifolius]
c87783.graph_	2.87E-50	1.28990463	up	Gamma-glutamyltranspeptidase 1 [Glycine soja]
c79288.graph_c0	3.13E-05	-1.0273021	down	putative polygalacturonase-inhibiting protein precursor [Phaseolus acutifolius]

c83796.graph_	1.63E-23	1.77001827	up	2-methylene-furan-3-one reductase-like [Cajanus cajan]
c87958.graph_	1.45E-25	-1.8598686	down	putative endopolygalacturonase [Diplodia seriata]
c82558.graph_	3.33E-06	-1.1985524	down	putative period circadian [Diplodia seriata]
c38362.graph_	2.49E-12	-1.7798406	down	casein kinase I isoform delta [Diaporthe helianthi]
c79864.graph_ c0	8.85E-16	1.21709962	up	PREDICTED: xyloglucan galactosyltransferase KATAMARI1 homolog [Glycine max]
c70100.graph_	1.48E-08	-1.412402	down	hypothetical protein GLYMA_02G055000 [Glycine max]
c79634.graph_	2.14E-08	-1.4488449	down	60s ribosomal protein l20 [Fusarium langsethiae]
c68963.graph_	2.14E-18	-2.1728248	down	putative d-galacturonic acid reductase [Diplodia seriata]
c73153.graph_	1.07E-09	-1.5752623	down	hypothetical protein BKCO1_2000077 [Diplodia corticola]
c71888.graph_	2.18E-12	-1.7848748	down	ammonium transporter MEP1 [Diaporthe helianthi]
c95541.graph_	1.89E-21	2.38813499	up	unknown [Lotus japonicus]
c90289.graph_	3.01E-08	-1.4368184	down	unnamed protein product [Fusarium sp. FIESC_5 CS3069]
c36933.graph_	2.19E-29	-2.7734591	down	hypothetical protein DHEL01_v212385 [Diaporthe
c81186.graph_ c1	9.93E-06	-1.1705993	down	PREDICTED: spermidine hydroxycinnamoyl transferase-like [Glycine max]
c82684.graph_ c0	9.73E-13	1.7761372	up	Pathogenesis-related protein PR-1 type [Colletotrichum chlorophyti]
c90329.graph_	1.27E-05	1.05852122	up	uncharacterized protein LOC109790137 [Cajanus cajan]
c38072.graph_	1.40E-23	-2.5019445	down	hypothetical protein DHEL01_v210153 [Diaporthe
c81672.graph_ c0	4.39E-13	-1.1140411	down	PREDICTED: uncharacterized protein LOC109328928 isoform X1 [Lupinus angustifolius]
c93338.graph_	2.19E-08	1.11528547	up	putative protein tag-278 [Cajanus cajan]
c89039.graph_	3.87E-32	-2.5870553	down	cannabidiolic acid synthase-like 2 [Cajanus cajan]
c95279.graph_	7.57E-71	-3.6868477	down	sugar transporter [Diplodia corticola]
c94102.graph_	1.17E-21	-2.2837047	down	--
c72234.graph_	5.25E-26	-1.1982416	down	uncharacterized protein LOC109788712 [Cajanus cajan]
c94332.graph_ c0	7.63E-36	-1.0714651	down	PREDICTED: kinesin-like protein KIN12B isoform X1 [Glycine max]
c96135.graph_	8.72E-14	-1.8961411	down	6-phosphogluconate dehydrogenase 2 [Diaporthe helianthi]
c70106.graph_	6.60E-11	-1.6687098	down	Malate synthase, glyoxysomal [Diplodia seriata]
c93934.graph_ c0	1.17E-09	1.00051642	up	PREDICTED: galactinol--sucrose galactosyltransferase-like [Glycine max]
c94479.graph_	1.28E-10	-1.5232715	down	unnamed protein product [Fusarium venenatum]
c55325.graph_ c0	3.69E-13	-1.8477892	down	guanine nucleotide-binding protein alpha-2 subunit [Diaporthe helianthi]
c82618.graph_	4.23E-17	-2.1294548	down	hypothetical protein DHEL01_v204878 [Diaporthe
c95486.graph_	8.66E-39	-3.1388097	down	hypothetical protein AOQ84DRAFT_423185 [Glonium
c89411.graph_ c0	2.85E-06	1.07399774	up	uncharacterized protein LOC109799085 isoform X1 [Cajanus cajan]
c96462.graph_	5.99E-11	1.67983936	up	uncharacterized protein LW93_9080 [Fusarium fujikuroi]
c91662.graph_ c0	2.60E-05	-1.120561	down	probable 1,3-beta-D-glucan synthase subunit [Fusarium mangiferae]
c87501.graph_	2.30E-11	-1.6184763	down	Ribosomal protein L19/L19e [Macrophomina phaseolina
c85909.graph_ c0	1.49E-22	1.21123175	up	PREDICTED: protein DETOXIFICATION 9 isoform X1 [Glycine max]
c74687.graph_	4.91E-63	1.04870466	up	hypothetical protein PHAVU_003G078300g [Phaseolus
c78130.graph_	8.56E-20	-2.2950873	down	pisatin demethylase [Diaporthe helianthi]
c56335.graph_ c0	5.34E-09	-1.4719511	down	nuclear localization sequence binding protein [Diaporthe helianthi]

c88547.graph_	2.28E-13	-1.8497832	down	40S ribosomal protein S4 [Cercospora zeina]
c78536.graph_	1.15E-23	-1.0390063	down	G2/mitotic-specific cyclin S13-7 [Cajanus cajan]
c60209.graph_	1.44E-15	-1.5987785	down	beta-galactosidase-like isoform X2 [Cajanus cajan]
c84402.graph_	1.53E-05	-1.1250352	down	related to dnase1 protein [Fusarium mangiferae]
c95465.graph_ c0	0.000177	1.00937306	up	PREDICTED: short-chain dehydrogenase reductase 2a-like [Lupinus angustifolius]
c85262.graph_	7.83E-13	-1.170956	down	hypothetical protein glysoja_020024 [Glycine soja]
c86079.graph_ c0	9.51E-93	-1.3367969	down	PREDICTED: epidermis-specific secreted glycoprotein EP1-like [Glycine max]
c56800.graph_	2.31E-98	1.07847039	up	1-aminocyclopropane-1-carboxylate oxidase 5 [Cajanus
c96480.graph_	9.18E-15	-1.9690938	down	4-hydroxyphenylpyruvate dioxygenase [Diaporthe
c88216.graph_	1.49E-06	-1.2665539	down	hypothetical protein FOXB_05225 [Fusarium oxysporum
c57714.graph_	2.21E-12	-1.7850429	down	putative tyrosinase [Diplodia seriata]
c91793.graph_	1.47E-39	-1.4938001	down	cinnamoyl-CoA reductase-like SNL6 [Cajanus cajan]
c80968.graph_ c0	6.56E-06	-1.1859953	down	putative erythrocyte band 7 integral membrane protein [Diaporthe ampelina]
c95506.graph_	4.53E-09	-1.5050116	down	hypothetical protein UCDDS831_g05251 [Diplodia
c69666.graph_ c0	2.27E-109	-4.774465	down	conidial yellow pigment biosynthesis polyketide synthase [Aspergillus lentulus]
c55529.graph_	1.61E-19	-2.2799765	down	putative histone h1 [Diaporthe ampelina]
c96004.graph_	1.25E-12	-1.8047112	down	hypothetical protein DHEL01_v204164 [Diaporthe
c95430.graph_	2.61E-29	1.51229037	up	nicotianamine synthase-like [Cajanus cajan]
c95706.graph_	2.86E-159	-5.4811552	down	hypothetical protein ANO11243_042010 [fungal sp.
c81654.graph_ c1	5.74E-09	-1.3305838	down	PREDICTED: alpha,alpha-trehalose-phosphate synthase [UDP-forming] 1-like [Glycine max]
c57373.graph_	3.34E-14	-1.9268426	down	vacuolar protease A [Diaporthe helianthi]
c97826.graph_	1.83E-14	-1.9467077	down	hypothetical protein DHEL01_v212084 [Diaporthe
c91745.graph_ c0	1.75E-06	-1.0607765	down	PREDICTED: putative receptor-like protein kinase At4g00960 [Glycine max]
c86420.graph_	1.28E-11	-1.6500258	down	putative 60s ribosomal protein l10 [Diplodia seriata]
c95616.graph_	1.53E-73	-4.0892313	down	hemagglutinin [Diplodia corticola]
c37973.graph_	1.89E-43	-3.2779012	down	putative pectate lyase A [Diplodia seriata]
c54683.graph_ c0	5.01E-38	-3.0692817	down	PREDICTED: uncharacterized protein LOC100813287 [Glycine max]
c38233.graph_	8.29E-21	-2.3525027	down	phosphate transporter [Diaporthe helianthi]
c87595.graph_	7.01E-54	1.16145669	up	UDP-glycosyltransferase 79B30-like [Cajanus cajan]
c88676.graph_	5.38E-54	1.63404729	up	hypothetical protein PHAVU_002G171800g [Phaseolus
c85514.graph_	1.46E-42	1.67868477	up	hypothetical protein VITISV_019223 [Vitis vinifera]
c76615.graph_	1.47E-33	-1.0418021	down	PREDICTED: chalcone synthase 1 [Lupinus angustifolius]
c94439.graph_	3.05E-104	1.10571434	up	PREDICTED: receptor-like protein 12 [Glycine max]
c74108.graph_	1.90E-11	-1.0496485	down	3-ketoacyl-CoA synthase 12 [Vigna radiata var. radiata]
c78104.graph_	7.45E-08	-1.4017381	down	putative protein yop-1 [Diaporthe ampelina]
c69843.graph_	6.15E-123	-2.2598224	down	hypothetical protein KK1_035105 [Cajanus cajan]
c68164.graph_	1.70E-08	-1.4459041	down	Sel1-like protein [Macrophomina phaseolina MS6]
c77425.graph_	2.61E-08	1.44644169	up	Heat shock cognate 70 kDa protein [Glycine soja]
c86678.graph_	2.97E-39	1.5299903	up	hypothetical protein GLYMA_05G247900 [Glycine max]
c57784.graph_	6.82E-10	-1.5623722	down	putative extracellular dioxygenase [Diaporthe ampelina]
c89538.graph_	4.86E-19	-1.8313288	down	hypothetical protein FNYG_13211 [Fusarium nygamai]
c76461.graph_	1.49E-13	1.72260732	up	hypothetical protein PHAVU_003G088700g [Phaseolus

c92839.graph_	2.26E-26	-1.1032638	down	hypothetical protein PHAVU_007G111200g [Phaseolus
c84047.graph_	8.15E-07	-1.2955739	down	PREDICTED: peroxidase P7-like [Glycine max]
c72329.graph_	1.97E-16	-2.0559152	down	hypothetical protein UCDDS831_g07036 [Diplodia
c81279.graph_	2.61E-20	-2.3244508	down	hypothetical protein UCDDA912_g03080 [Diaporthe
c88075.graph_	9.24E-16	-1.8302329	down	acetyl-coenzyme a synthetase [Nannochloropsis gaditana]
c38089.graph_	1.29E-12	-1.802719	down	putative mitochondrial peroxiredoxin prx1 [Diaporthe
c74307.graph_	2.12E-15	-2.0151652	down	uncharacterized protein LW93_4409 [Fusarium fujikuroi]
c95601.graph_	2.28E-241	6.04399366	up	trypsin inhibitor [Apios americana]
c93276.graph_	0	3.74296417	up	late embryogenesis abundant protein [Arachis ipaensis]
c95444.graph_	5.07E-08	-1.3910869	down	hypothetical protein CRG98_043801 [Punica granatum]
c73112.graph_c0	9.56E-10	-1.2451795	down	Homeobox-leucine zipper protein ATHB-40, partial [Cajanus cajan]
c88164.graph_	2.09E-06	1.22841621	up	DUF3148 family protein [Medicago truncatula]
c84849.graph_	8.15E-05	-1.0560399	down	hypothetical protein FPOA_03201 [Fusarium poae]
c86530.graph_	3.04E-06	1.22315777	up	transcription factor PRE3 [Cajanus cajan]
c55238.graph_c0	4.22E-36	-2.8044183	down	putative beta- -glucan-binding protein [Neofusicoccum parvum UCRNP2]
c97742.graph_c0	1.93E-13	-1.869798	down	GPI-anchored cell wall beta-1,3-endoglucanase EglC [Diaporthe helianthi]
c80969.graph_	1.05E-05	1.09589176	up	hypothetical protein PHAVU_005G177000g [Phaseolus
c76602.graph_	5.53E-18	-1.6283857	down	uncharacterized protein LOC109801205 [Cajanus cajan]
c72317.graph_	1.76E-14	-1.9478599	down	hypothetical protein DHEL01_v205835 [Diaporthe
c75906.graph_c0	1.71E-15	-2.0216007	down	hypothetical protein THITE_2048634 [Thielavia terrestris NRRL 8126]
c95202.graph_c0	1.39E-08	-1.0997696	down	Retrovirus-related Pol polyprotein from transposon TNT 1-94 [Cajanus cajan]
c68017.graph_	6.55E-40	-3.1763665	down	hypothetical protein DHEL01_v202969 [Diaporthe
c95543.graph_	1.08E-23	-2.4936877	down	glycoside hydrolase family 5 protein [Diplodia corticola]
c54626.graph_	7.66E-57	-1.4300005	down	--
c83140.graph_	4.96E-15	-1.411226	down	hypothetical protein PHAVU_005G119300g [Phaseolus
c92163.graph_	9.74E-09	-1.4470039	down	related to putative tartrate transporter [Fusarium
c39758.graph_	1.28E-11	-1.7198971	down	sugar transporter [Diplodia corticola]
c56179.graph_	1.75E-09	-1.5548995	down	bzip transcription factor [Diplodia corticola]
c74577.graph_	4.23E-14	-1.9157309	down	hypothetical protein UCDDS831_g04100 [Diplodia
c88169.graph_	8.23E-68	1.00040244	up	acid beta-fructofuranosidase [Cajanus cajan]
c76570.graph_	2.38E-67	-1.3219917	down	Beta-galactosidase [Glycine soja]
c90174.graph_	3.35E-51	1.06023747	up	unknown [Glycine max]
c54119.graph_	1.37E-17	-2.1608933	down	hypothetical protein BK809_0005735 [Diplodia seriata]
c66152.graph_	1.07E-11	-1.7262373	down	hypothetical protein DHEL01_v211074 [Diaporthe
c94095.graph_	5.76E-10	1.45372006	up	Thiosulfate sulfurtransferase, chloroplastic [Glycine soja]
c93118.graph_	2.66E-07	-1.1632306	down	probable elongation factor 2 [Fusarium fujikuroi IMI
c86622.graph_	7.29E-30	1.97705845	up	hypothetical protein glysoja_026788 [Glycine soja]
c87685.graph_	2.29E-07	-1.3335987	down	laccase-15-like [Cajanus cajan]
c92977.graph_c0	4.41E-161	2.84394184	up	PREDICTED: LOW QUALITY PROTEIN: protein TOO MANY MOUTHS [Glycine max]
c38172.graph_	3.34E-12	-1.7697713	down	uncharacterized protein FRV6_00773 [Fusarium
c53869.graph_	5.85E-14	-1.9096972	down	hypothetical protein BK809_0007877 [Diplodia seriata]
c57337.graph_	7.72E-09	-1.4555975	down	extracellular cellulase allergen asp f7 [Fusarium

c90975.graph_ c0	1.49E-11	1.02622402	up	PREDICTED: putative glucose-6-phosphate 1-epimerase [Cicer arietinum]
c67910.graph_	8.57E-23	-2.4608294	down	hypothetical protein DHEL01_v200454 [Diaporthe
c84679.graph_	1.17E-05	-1.1645471	down	probable ribosomal protein L13B [Fusarium fujikuroi IMI
c70220.graph_	2.49E-05	-1.1245787	down	hypothetical protein FPOA_01915 [Fusarium poae]
c90967.graph_	7.60E-06	-1.1698367	down	S-locus lectin kinase family protein [Medicago truncatula]
c96464.graph_	1.78E-14	-1.9511667	down	putative glycoside hydrolase family 3 [Diplodia seriata]
c94460.graph_ c0	7.52E-15	1.52436752	up	PREDICTED: uncharacterized protein LOC101508823 isoform X2 [Cicer arietinum]
c37949.graph_	4.61E-32	-1.3121656	down	PREDICTED: cytochrome P450 CYP73A100-like
c94694.graph_	1.02E-08	1.39018226	up	ribonuclease H, partial [Trifolium pratense]
c87459.graph_	6.02E-05	-1.0738816	down	probable DFG5 protein [Fusarium proliferatum]
c39041.graph_	4.86E-09	-1.488304	down	neutral amino acid [Diplodia corticola]
c84375.graph_	4.10E-07	-1.3194879	down	hypothetical protein UCDDS831_g00380 [Diplodia
c38457.graph_	1.65E-20	-2.3358061	down	proteinase T [Diaporthe helianthi]
c85250.graph_ c0	3.24E-06	-1.1737876	down	fucoxanthin chlorophyll a /c binding protein [Heterosigma akashiwo]
c95527.graph_	4.89E-18	-2.1890767	down	--
c91263.graph_	6.89E-29	-1.7510689	down	PREDICTED: E3 ubiquitin-protein ligase ATL6 [Glycine
c83686.graph_	1.44E-26	-1.712076	down	syntaxin of plants 122 protein [Medicago truncatula]
c89362.graph_	0	5.70425685	up	primary amine oxidase [Vigna radiata var. radiata]
c96082.graph_	4.67E-26	-2.6226484	down	hypothetical protein DHEL01_v212544 [Diaporthe
c61457.graph_	7.06E-12	-1.7426468	down	putative cellobiose dehydrogenase [Diplodia seriata]
c37660.graph_	2.70E-10	-1.6204406	down	hypothetical protein UCDDS831_g01626 [Diplodia
c76646.graph_ c0	6.92E-21	1.7016915	up	lysM domain-containing GPI-anchored protein 1-like [Cajanus cajan]
c80849.graph_ c0	1.68E-26	-2.5676308	down	RNA-binding domain-containing protein [Meliniomyces bicolor E]
c81859.graph_ c0	9.43E-23	-1.0952233	down	probable inactive poly [ADP-ribose] polymerase SRO2 [Vigna radiata var. radiata]
c74683.graph_ c0	1.51E-05	-1.1303606	down	PREDICTED: probable galacturonosyltransferase-like 10 [Glycine max]
c95338.graph_	2.13E-21	-2.3865604	down	scytalone dehydratase [Diplodia corticola]
c75713.graph_	9.22E-23	-1.1199568	down	uncharacterized protein LOC109787963 [Cajanus cajan]
c93050.graph_ c0	3.78E-20	2.14413069	up	PREDICTED: ABC transporter B family member 9 [Cucumis melo]
c38207.graph_	1.31E-31	-2.8449074	down	Laccase-2, partial [Diplodia seriata]
c84225.graph_	1.01E-23	-1.0035075	down	hypothetical protein PHAVU_003G212200g [Phaseolus
c82120.graph_	1.17E-12	-1.6870201	down	hypothetical protein FNYG_05807 [Fusarium nygamai]
c91260.graph_	3.22E-18	-1.7961729	down	hypothetical protein FOXB_08964 [Fusarium oxysporum
c86049.graph_ c0	7.38E-14	-1.901355	down	uncharacterized protein FFUJ_12625 [Fusarium fujikuroi IMI 58289]
c91693.graph_ c0	1.77E-22	-2.1687425	down	nucleosome assembly protein 1-like 1 [Fusarium verticillioides 7600]
c53807.graph_	5.01E-34	-1.9139214	down	Peroxidase 47 [Glycine soja]
c81325.graph_	1.12E-07	-1.3798943	down	adenine phosphoribosyltransferase [Diaporthe helianthi]
c82001.graph_	3.01E-08	-1.4265851	down	Transcription factor Opi1 [Macrophomina phaseolina
c56903.graph_	2.62E-11	-1.6919574	down	hypothetical protein VM1G_08555 [Valsa mali]
c92493.graph_ c0	5.82E-16	-1.157106	down	PREDICTED: uncharacterized membrane protein At3g27390 [Glycine max]

c77237.graph_	1.38E-17	-2.1607095	down	putative beta-glucanosyltransferase [Diplodia seriata]
c95547.graph_	8.85E-05	-1.0464253	down	Hypothetical protein PINS_007590 [Pythium insidiosum]
c97420.graph_	1.04E-10	-1.6384868	down	hypothetical protein DHEL01_v204821 [Diaporthe
c79317.graph_	5.87E-19	-2.2461772	down	putative woronin body major protein [Diaporthe ampelina]
c81896.graph_	0.0001748	-1.0137488	down	mfs agza xanthine uracil permease [Fusarium langsethiae]
c90417.graph_ c0	1.88E-34	1.66413645	up	PREDICTED: 11 kDa late embryogenesis abundant protein-like [Cicer arietinum]
c54836.graph_	4.78E-11	-1.6687106	down	hypothetical protein UCREL1_8267 [Eutypa lata
c81525.graph_	2.07E-05	-1.1339642	down	unnamed protein product [Fusarium venenatum]
c89488.graph_	1.74E-36	1.30943021	up	hypothetical protein PHAVU_007G020800g [Phaseolus
c37517.graph_	3.88E-11	-1.6769237	down	putative had superfamily hydrolase [Diplodia seriata]
c37716.graph_ c0	1.88E-26	-2.6435013	down	putative gpi anchored serine-threonine rich protein [Diaporthe ampelina]
c85437.graph_	2.89E-205	3.28745706	up	hypothetical protein PHAVU_006G184200g [Phaseolus
c78319.graph_	5.78E-19	-1.0344445	down	hypothetical protein glysoja_004502 [Glycine soja]
c93671.graph_ c0	1.88E-14	1.94965454	up	PREDICTED: seed biotin-containing protein SBP65-like [Lupinus angustifolius]
c96418.graph_	8.19E-09	-1.4826851	down	alpha beta-hydrolase [Diplodia corticola]
c76133.graph_ c0	8.40E-20	-1.1496913	down	PREDICTED: crocetin glucosyltransferase, chloroplastic-like [Glycine max]
c85279.graph_	6.54E-06	-1.1941514	down	hypothetical protein FOXB_13379 [Fusarium oxysporum
c83694.graph_ c1	6.94E-06	-1.1863664	down	hypothetical protein FPSE_00057 [Fusarium pseudograminearum CS3096]
c92390.graph_ c0	1.06E-69	1.84020701	up	hypothetical protein VIGAN_01469900, partial [Vigna angularis var. angularis]
c90015.graph_ c0	9.62E-15	-1.9184072	down	hypothetical protein AU210_000846 [Fusarium oxysporum f. sp. radicis-cucumerinum]
c54117.graph_	4.40E-74	-1.0179601	down	aspartyl protease AED3-like [Cajanus cajan]
c87941.graph_	1.40E-06	-1.2698104	down	probable caspase [Fusarium proliferatum ET1]
c76425.graph_	2.71E-06	-1.2383097	down	atp synthase subunit mitochondrial [Fusarium langsethiae]
c72646.graph_	1.02E-17	-1.1165956	down	hypothetical protein LR48_Vigan03g141400 [Vigna
c92328.graph_	1.25E-06	-1.2753287	down	hypothetical protein FOXB_09165 [Fusarium oxysporum
c73914.graph_	3.18E-38	-3.1192485	down	putative alcohol dehydrogenase [Diaporthe ampelina]
c94052.graph_	0	2.97765244	up	PREDICTED: vicilin-like antimicrobial peptides 2-2
c80517.graph_	3.62E-06	-1.1329347	down	unnamed protein product [Fusarium graminearum]
c81862.graph_	1.14E-09	-1.5684015	down	hypothetical protein BK809_0001124 [Diplodia seriata]
c73163.graph_	1.08E-07	-1.2284413	down	Werner Syndrome-like exonuclease [Vigna radiata var.
c53827.graph_	3.82E-22	-2.4266132	down	hypothetical protein DHEL01_v210943 [Diaporthe
c79654.graph_ c0	4.11E-11	1.10298705	up	hypothetical protein VIGAN_08245800 [Vigna angularis var. angularis]
c75877.graph_	8.54E-10	-1.5687385	down	hypothetical protein BKCO1_5000183 [Diplodia corticola]
c73858.graph_	7.25E-10	-1.5899723	down	cofilin [Diplodia corticola]
c86019.graph_	9.72E-11	-1.6008482	down	unnamed protein product [Fusarium sp. FIESC_5 CS3069]
c74390.graph_ c0	6.28E-08	1.03330319	up	PREDICTED: probable xyloglucan endotransglucosylase/hydrolase protein 23 [Cicer
c40098.graph_	1.68E-12	-1.7947191	down	SPFH domain/Band 7 family protein [Diaporthe helianthi]
c81623.graph_	8.29E-12	-1.1790566	down	UDP-glucose 6-dehydrogenase 5-like isoform X1 [Cajanus
c37508.graph_	2.94E-34	-2.9711114	down	transcriptional regulator prz1 [Diaporthe helianthi]
c85755.graph_ c0	5.19E-16	-1.2742769	down	AP2-like ethylene-responsive transcription factor At1g16060 [Vigna radiata var. radiata]

c82663.graph_ c0	1.72E-05	-1.1298454	down	PREDICTED: uncharacterized protein LOC102669378 [Glycine max]
c86157.graph_	1.33E-05	-1.1578858	down	putative snare domain-containing protein [Diplodia seriata]
c76045.graph_	5.11E-06	-1.1727487	down	hypothetical protein PHAVU_009G001800g [Phaseolus
c85596.graph_	8.95E-22	-2.0459395	down	putative non-histone chromosomal protein 6 [Diplodia
c96984.graph_ c0	0.0001484	-1.0084511	down	Transcription factor MADS-box [Macrophomina phaseolina MS6]
c84650.graph_	2.59E-23	-1.8541137	down	protein DMR6-LIKE OXYGENASE 1-like [Cajanus
c60932.graph_ c0	3.75E-10	-1.5866284	down	C2H2 type zinc finger domain-containing protein [Diaporthe helianthi]
c95484.graph_ c0	2.83E-30	-2.8136825	down	hypothetical protein TCE0_044f16403 [Talaromyces cellulolyticus]
c70987.graph_	3.37E-54	-1.0248336	down	hypothetical protein GLYMA_01G004200 [Glycine max]
c95389.graph_	2.91E-18	-2.1951266	down	glycoside hydrolase family 43 protein [Diplodia corticola]
c38514.graph_ c0	1.37E-32	-2.9057414	down	hypothetical protein TRIATDRAFT_225995 [Trichoderma atroviride IMI 206040]
c84041.graph_	1.47E-14	-1.6403603	down	putative WRKY transcription factor 46 [Cajanus cajan]
c55344.graph_ c0	5.11E-06	-1.1899399	down	putative formate dehydrogenase protein [Neofusicoccum parvum UCRNP2]
c95599.graph_	4.55E-17	-2.1256859	down	Glycoside hydrolase family 43 [Macrophomina phaseolina
c70554.graph_	2.47E-29	-2.6443828	down	amino acid transporter [Diplodia corticola]
c91839.graph_ c0	0	4.13761165	up	probable CDP-alcohol phosphatidyltransferase [Fusarium mangiferae]
c75624.graph_	4.25E-22	-1.1733166	down	chitinase [Medicago truncatula]
c81688.graph_ c1	1.30E-06	1.21400653	up	CBL-interacting serine/threonine-protein kinase 14-like [Cajanus cajan]
c81367.graph_	9.29E-180	2.47415776	up	PREDICTED: gibberellin 20-oxidase 1 isoform X1
c80521.graph_	1.14E-85	1.18318858	up	cytochrome P450 78A5-like [Cajanus cajan]
c79707.graph_ c0	3.90E-16	1.02097762	up	PREDICTED: ethylene-responsive transcription factor ERF060-like [Glycine max]
c83666.graph_	2.02E-23	1.79702996	up	uncharacterized protein LOC100809384 [Glycine max]
c89339.graph_	4.72E-17	-1.9871237	down	unnamed protein product [Fusarium venenatum]
c83861.graph_	4.16E-08	-1.4052217	down	putative zinc finger, CCHC-type [Helianthus annuus]
c90202.graph_	2.49E-11	-1.6190617	down	probable 60S ribosomal protein L5 [Fusarium
c38221.graph_	6.04E-05	-1.0757425	down	60S ribosomal protein L10A [Ectocarpus siliculosus]
c91913.graph_ c0	9.62E-13	1.3867381	up	beta-1,4-N-acetylglucosaminyltransferase-like protein [Medicago truncatula]
c88402.graph_	3.82E-22	2.08454264	up	cinnamoyl-CoA reductase 1 [Arachis ipaensis]
c91077.graph_ c0	1.69E-07	-1.3394958	down	probable inactive poly [ADP-ribose] polymerase SRO2 isoform X2 [Vigna radiata var. radiata]
c98510.graph_	2.39E-09	-1.5085623	down	hypothetical protein ALT_5891 [Aspergillus lentulus]
c93354.graph_ c0	2.35E-49	1.37463561	up	PREDICTED: protein CHROMATIN REMODELING 25 [Glycine max]
c77572.graph_	3.29E-06	-1.2051925	down	hypothetical protein BK809_0006981 [Diplodia seriata]
c81168.graph_	1.68E-17	1.03345217	up	hypothetical protein glysoja_013685 [Glycine soja]
c37723.graph_	8.84E-54	-3.4229623	down	putative peptide transporter ptr2 [Diplodia seriata]
c91019.graph_	3.01E-25	2.11194976	up	hypothetical protein glysoja_030230 [Glycine soja]
c74062.graph_	2.60E-08	-1.3990096	down	putative mfs transporter [Diaporthe ampelina]
c85280.graph_ c0	5.86E-05	-1.0623404	down	hypothetical protein ASCRUDRAFT_43254 [Ascoidea rubescens DSM 1968]
c82134.graph_	7.79E-11	-1.0747764	down	hypothetical protein GLYMA_08G289300 [Glycine max]

c36914.graph_c0	1.64E-14	-1.9504164	down	benzoate 4-monooxygenase cytochrome P450 [Diaporthe helianthi]
c81720.graph_	4.06E-50	-1.5683404	down	hypothetical protein LR48_Vigan10g006100 [Vigna
c66667.graph_	4.75E-12	-1.7548591	down	hypothetical protein DHEL01_v205818 [Diaporthe
c87369.graph_c0	1.14E-08	-1.0731819	down	hypothetical protein VIGAN_07044400 [Vigna angularis var. angularis]
c72799.graph_	3.24E-07	-1.3288403	down	hypothetical protein KK1_033457 [Cajanus cajan]
c78692.graph_c0	3.88E-15	-1.9982207	down	hypothetical protein MPH_03925 [Macrophomina phaseolina MS6]
c93887.graph_	3.23E-05	-1.1096929	down	rasp f 7 allergen [Fusarium langsethiae]
c52952.graph_c0	6.63E-07	-1.2999668	down	PREDICTED: uncharacterized protein LOC101505812 [Cicer arietinum]
c37737.graph_	2.01E-26	-1.4057344	down	pathogenesis-related protein STH-2-like [Cajanus cajan]
c78637.graph_c0	5.52E-111	-2.461368	down	tryptophan aminotransferase-related protein 4-like [Cajanus cajan]
c96635.graph_	1.33E-28	-2.7386748	down	nitrate reductase [Diaporthe helianthi]
c38034.graph_	0.0001223	-1.0226951	down	PREDICTED: 40S ribosomal protein S2-3-like [Ipomoea
c39741.graph_	3.46E-06	1.22049903	up	putative invertase inhibitor precursor [Glycine max]
c74831.graph_	1.53E-08	1.44352264	up	PREDICTED: transcription factor bHLH35 [Vitis vinifera]
c54596.graph_	0.0001072	-1.0428603	down	arabinogalactan protein [Medicago truncatula]
c85824.graph_c0	3.88E-07	-1.3222928	down	uncharacterized protein FPRO_12092 [Fusarium proliferatum ET1]
c37908.graph_	3.44E-23	-2.4272022	down	GPR1/FUN34/yaaH [Macrophomina phaseolina MS6]
c92434.graph_	1.68E-19	2.27904636	up	ABA-responsive protein [Medicago truncatula]
c70394.graph_	8.91E-06	-1.171169	down	c6 finger domain-containing protein [Diplodia corticola]
c95193.graph_	3.57E-09	1.10552637	up	retrotransposon-related protein [Trifolium pratense]
c92856.graph_	4.72E-07	1.19186757	up	hypothetical protein GLYMA_04G187900 [Glycine max]
c96132.graph_	3.55E-10	-1.589398	down	NAD(P)-binding protein [Cenococcum geophilum 1.58]
c86184.graph_c0	1.07E-06	-1.2543254	down	hypothetical protein FPSE_11766 [Fusarium pseudograminearum CS3096]
c96266.graph_	2.87E-10	-1.5986258	down	O-methyltransferase [Paraphaeosphaeria sporulosa]
c81647.graph_	6.52E-09	1.24294002	up	protein DOWNY MILDEW RESISTANCE 6-like
c95581.graph_	7.99E-28	-2.7091246	down	protein of unknown function DUF4243 [Penicillium
c81737.graph_c0	2.49E-14	1.32518789	up	PREDICTED: LOW QUALITY PROTEIN: organic cation/carnitine transporter 3-like [Glycine max]
c96129.graph_	3.21E-11	-1.6842997	down	putative coactivator bridging factor 1 [Diaporthe ampelina]
c80664.graph_	2.76E-36	3.04790557	up	retrotransposon-related protein [Trifolium pratense]
c88805.graph_	3.25E-13	-1.1221836	down	kelch repeat-containing protein At3g27220-like [Cajanus
c91679.graph_	6.51E-06	-1.1792218	down	putative 60s ribosomal protein l8 [Diaporthe ampelina]
c76697.graph_	1.40E-06	-1.0200787	down	hypothetical protein glysoja_033121 [Glycine soja]
c55588.graph_	9.90E-22	-2.3932075	down	glycoside hydrolase family 28 protein [Diplodia corticola]
c74832.graph_	2.94E-34	-2.9708358	down	cellodextrin transport-2 [Diaporthe helianthi]
c78362.graph_	4.89E-21	-2.3658437	down	IDI4 [Diaporthe helianthi]
c57179.graph_	2.47E-11	-1.7130291	down	glycoside hydrolase family 20 protein [Diplodia corticola]
c68628.graph_	6.52E-39	-3.0091548	down	putative oligopeptide transporter [Diplodia seriata]
c91080.graph_c0	2.96E-10	-1.5544585	down	retrovirus-related Pol polyprotein from transposon TNT 1-94 [Trifolium pratense]
c86799.graph_c0	1.44E-06	-1.2684209	down	60s ribosomal protein l33 [Colletotrichum orbiculare MAFF 240422]
c89030.graph_	1.82E-05	-1.0077214	down	heat shock protein 83 [Arachis ipaensis]

c89179.graph_	2.01E-10	1.14619345	up	unknown [Glycine max]
c92939.graph_	2.56E-24	-2.22121	down	hypothetical protein DHEL01_v207605 [Diaporthe
c85645.graph_	2.29E-15	-1.9383469	down	plasma membrane h+-atpase pma1 [Diplodia corticola]
c95341.graph_	5.16E-06	-1.0136216	down	myb-related protein 306-like isoform X1 [Cajanus cajan]
c87048.graph_	3.00E-07	-1.3102051	down	putative mitochondrial dna replication protein [Diplodia
c88334.graph_ c0	9.90E-06	-1.0918291	down	probable ammonium transporter MEAA [Fusarium fujikuroi IMI 58289]
c90783.graph_	2.05E-136	4.01556296	up	extensin [Cajanus cajan]
c85390.graph_	4.39E-107	4.12876395	up	hypothetical protein GLYMA_U020300 [Glycine max]
c89102.graph_ c0	3.63E-07	-1.0346794	down	hydroquinone glucosyltransferase-like protein [Trifolium pratense]
c88714.graph_	3.22E-05	1.02985153	up	nodulation-signaling pathway 1 protein [Trifolium
c97760.graph_	5.68E-10	-1.5702973	down	peptidase S41 family protein [Diaporthe helianthi]
c81668.graph_	2.01E-88	-1.1995015	down	salicylic acid-binding protein 2-like [Cajanus cajan]
c95531.graph_	1.92E-07	-1.3417313	down	uncharacterized protein LOC100500462 [Glycine max]
c79868.graph_	5.16E-14	-1.2113248	down	mitotic spindle checkpoint protein BUBR1 [Cajanus cajan]
c86696.graph_	4.80E-73	2.92248809	up	PREDICTED: chitinase 2-like [Cicer arietinum]
c94349.graph_	3.95E-19	-2.0353899	down	chitin synthase [Fusarium oxysporum FOSC 3-a]
c79371.graph_ c0	2.67E-06	-1.180567	down	Nodule Cysteine-Rich (NCR) secreted peptide [Medicago truncatula]
c84821.graph_	4.46E-06	-1.1999599	down	unnamed protein product [Fusarium venenatum]
c38551.graph_	1.17E-12	-1.8068315	down	putative extracellular serine-rich protein [Diplodia seriata]
c81259.graph_	0	3.18301187	up	seed maturation protein [Glycine tomentella]
c71918.graph_	1.59E-10	-1.6222341	down	high-affinity nicotinic acid transporter [Diaporthe
c97704.graph_	2.96E-10	-1.6188033	down	putative copper radical oxidase [Diplodia seriata]
c94857.graph_	7.87E-46	-2.6527633	down	elongation factor 3 [Fusarium langsethiae]
c89035.graph_	3.14E-111	4.1259119	up	LADA_0D06304g1_1 [Lachancea dasiensis CBS 10888]
c79606.graph_ c0	0.0001943	-1.0074406	down	triosephosphate isomerase [Fusarium oxysporum f. sp. lycopersici 4287]
c53735.graph_	8.52E-13	1.37275044	up	hypothetical protein glysoja_029416 [Glycine soja]
c80387.graph_	0	6.93748069	up	Dehydration-responsive protein RD22 [Glycine soja]
c84176.graph_	4.00E-10	-1.3872808	down	hypothetical protein PHAVU_003G192000g [Phaseolus
c97086.graph_	4.33E-19	-2.2534396	down	hypothetical protein UCDDA912_g04436 [Diaporthe
c75630.graph_	1.08E-10	1.11359356	up	hypothetical protein PHAVU_007G127800g [Phaseolus
c57327.graph_	2.29E-22	-2.4380048	down	hypothetical protein DHEL01_v202806 [Diaporthe
c94868.graph_	1.92E-38	-1.2425109	down	putative mitochondrial protein [Dendrobium catenatum]
c86912.graph_	7.13E-13	-1.7745259	down	hypothetical protein TanjilG_10332 [Lupinus
c85802.graph_	3.70E-06	-1.2098794	down	uncharacterized protein LW94_666 [Fusarium fujikuroi]
c95019.graph_	1.52E-145	1.74701919	up	PREDICTED: protein SRG1 [Glycine max]
c81236.graph_ c1	4.35E-08	-1.2129742	down	uncharacterized protein LOC107609204 isoform X1 [Arachis ipaensis]
c74399.graph_	9.02E-19	-1.0074173	down	PREDICTED: MLP-like protein 423 [Lupinus
c91520.graph_	5.82E-08	-1.1615027	down	unnamed protein product [Fusarium sp. FIESC_5 CS3069]
c90932.graph_	2.28E-49	-2.90838	down	formate dehydrogenase [Diaporthe helianthi]
c81495.graph_ c0	1.38E-06	-1.2618689	down	hypothetical protein NECHADRAFT_62851 [[Nectria] haematococca mpVI 77-13-4]
c88099.graph_	3.84E-05	-1.0943013	down	atp-citrate synthase subunit 2 [Fusarium langsethiae]
c71496.graph_	3.00E-09	-1.5268091	down	hypothetical protein BK809_0001811 [Diplodia seriata]

c97451.graph_	4.56E-08	-1.4211132	down	amino acid transporter [Diplodia corticola]
c95660.graph_	3.46E-56	-3.665797	down	pectate lyase [Diplodia corticola]
c81545.graph_	2.39E-12	1.2454881	up	hypothetical protein glysoja_002197 [Glycine soja]
c78238.graph_	2.49E-23	-2.4901087	down	apses transcription factor [Diplodia corticola]
c88207.graph_	9.24E-31	-2.4424031	down	alcohol dehydrogenase 1 [Fusarium verticillioides 7600]
c85444.graph_ c0	3.62E-06	1.09841326	up	PREDICTED: uncharacterized protein LOC100527109 [Glycine max]
c38513.graph_	1.96E-13	-1.8692236	down	glycoside hydrolase family 10 protein [Diplodia corticola]
c38291.graph_	4.22E-21	-2.3486154	down	WSC domain-containing protein 2 [Diplodia seriata]
c36995.graph_	5.27E-10	-1.6021305	down	hypothetical protein UCDDS831_g02885 [Diplodia
c41324.graph_ c0	3.19E-24	-2.5339216	down	hypothetical protein MYCTH_2305635 [Thermothelomyces thermophila ATCC 42464]
c94797.graph_	4.61E-06	-1.1344129	down	hypothetical protein FPOA_02052 [Fusarium poae]
c92230.graph_	2.66E-83	-1.1622944	down	PREDICTED: dirigent protein 1-like [Glycine max]
c82493.graph_	1.35E-129	-3.5303739	down	PREDICTED: laccase-7-like [Glycine max]
c95711.graph_	3.23E-87	-4.3098328	down	glycoside hydrolase family 61 protein [Diplodia corticola]
c71742.graph_	5.42E-110	-1.7187953	down	protein P21-like [Cajanus cajan]
c77829.graph_	5.47E-36	-2.8520967	down	high-affinity glucose transporter RGT2 [Diaporthe
c82007.graph_	1.24E-13	-1.8100294	down	metacaspase a [Diplodia corticola]
c87797.graph_	5.70E-21	1.63722962	up	hypothetical protein CDL15_Pgr027786 [Punica granatum]
c81322.graph_ c0	1.63E-10	1.12949537	up	PREDICTED: uncharacterized protein LOC100790324 [Glycine max]
c98223.graph_	6.16E-11	-1.6597296	down	hypothetical protein DHEL01_v212357 [Diaporthe
c95462.graph_	4.72E-37	-3.0276224	down	acyl-synthetase [Diplodia corticola]
c72901.graph_	1.32E-08	-1.0535451	down	elongation of fatty acids protein 3-like [Arachis duranensis]
c92336.graph_	1.98E-163	-1.3553677	down	hypothetical protein PHAVU_009G232500g [Phaseolus
c95667.graph_	2.10E-22	-2.4413639	down	short-chain dehydrogenase [Talaromyces cellulolyticus]
c94446.graph_	1.02E-191	3.64794881	up	unnamed protein product [Fusarium venenatum]
c74855.graph_	0.0001719	-1.0146078	down	hypothetical protein PIROE2DRAFT_67050 [Piromyces
c95266.graph_	3.21E-38	2.38152644	up	basic 7S globulin precursor [Glycine max]
c91713.graph_	6.92E-21	-2.010875	down	transketolase [Diaporthe helianthi]
c54274.graph_	1.44E-92	4.44066168	up	unknown [Glycine max]
c73943.graph_	1.10E-10	-1.6362805	down	peroxisomal copper amine oxidase [Diaporthe helianthi]
c72364.graph_	0.0001161	-1.0367759	down	conserved unknown protein [Ectocarpus siliculosus]
c76278.graph_	7.42E-39	-1.1320965	down	cytochrome P450 family 81 protein [Medicago truncatula]
c96175.graph_	1.03E-17	-2.1687556	down	hypothetical protein DHEL01_v212941 [Diaporthe
c78050.graph_	3.89E-18	-2.1952146	down	putative salicylate hydroxylase [Diaporthe ampelina]
c94330.graph_	2.40E-17	-1.677768	down	probable heat shock protein 70 [Fusarium proliferatum]
c57677.graph_ c0	7.55E-28	-2.6993128	down	Unsaturated rhamnogalacturonyl hydrolase YteR [Diplodia seriata]
c98116.graph_	8.33E-12	-1.733324	down	quinate permease [Diaporthe helianthi]
c90737.graph_	2.16E-58	1.28834924	up	hypothetical protein TSUD_70490 [Trifolium
c55361.graph_	1.37E-76	4.14898742	up	hypothetical protein LR48_Vigan08g119000 [Vigna
c80388.graph_ c0	7.33E-22	-1.1203931	down	anthocyanidin reductase ((2S)-flavan-3-ol-forming) isoform X1 [Cajanus cajan]
c88297.graph_ c2	2.22E-05	-1.0022271	down	PREDICTED: probably inactive leucine-rich repeat receptor-like protein kinase At5g48380 [Glycine max]
c85346.graph_ c0	1.04E-27	1.27827466	up	malonyl-CoA:isoflavone 7-O-glucoside-6''-O-malonyltransferase [Glycine max]

c89061.graph_	7.29E-14	1.85404161	up	PREDICTED: polygalacturonase-like [Glycine max]
c90709.graph_	3.67E-28	-1.3713896	down	UPF0481 protein At3g47200-like [Arachis ipaensis]
c54061.graph_	1.02E-231	-3.2082528	down	PREDICTED: chitotriosidase-1-like [Lupinus
c91259.graph_	4.23E-05	-1.0174854	down	related to bZIP transcription factor [Fusarium proliferatum]
c88716.graph_	6.57E-06	-1.1841803	down	hypothetical protein TPAR_03732 [Tolypocladium
c75166.graph_	1.52E-09	1.27612113	up	class I heat shock protein-like [Cajanus cajan]
c98211.graph_	6.68E-12	-1.743923	down	hypothetical protein DHEL01_v209314 [Diaporthe
c95476.graph_ c0	1.12E-42	-3.2082986	down	Galactose-binding domain-like protein [Macrophomina phaseolina MS6]
c71186.graph_	4.55E-05	-1.089961	down	unknown [Lotus japonicus]
c69533.graph_ c0	5.52E-11	-1.6704422	down	hypothetical protein MPH_06284 [Macrophomina phaseolina MS6]
c60659.graph_ c2	9.20E-12	-1.7326756	down	putative nadh:flavin oxidoreductase nadh oxidase [Diplodia seriata]
c83977.graph_ c0	7.68E-21	-1.2646815	down	PREDICTED: tetrahydrocannabinolic acid synthase-like [Glycine max]
c95483.graph_	4.78E-26	-1.3618908	down	PREDICTED: scopoletin glucosyltransferase-like [Glycine
c55112.graph_	5.67E-31	1.9252143	up	PREDICTED: L-ascorbate oxidase homolog [Glycine
c91727.graph_	0.0001406	-1.0226648	down	disulfide-isomerase [Fusarium langsethiae]
c95517.graph_	6.38E-16	-2.0452562	down	glycoside hydrolase family 61 protein [Diplodia corticola]
c82933.graph_ c0	7.20E-09	-1.4997883	down	putative glucose-6-phosphate 1-dehydrogenase protein [Neofusicoccum parvum UCRNP2]
c91421.graph_	1.28E-72	-2.4665525	down	hypothetical protein DHEL01_v200924 [Diaporthe
c84981.graph_	1.20E-08	1.21200581	up	putative hydrolase yugF [Cajanus cajan]
c91277.graph_	1.01E-25	1.87315734	up	xyloglucan xylosyltransferase [Medicago truncatula]
c93554.graph_	0	4.9581916	up	sucrose binding protein homolog S-64 [Glycine max]
c65367.graph_	2.17E-11	-1.7175041	down	Transcriptional activator HAP2 [Diplodia seriata]
c95705.graph_	6.26E-26	-2.6166586	down	putative malate dehydrogenase [Diaporthe ampelina]
c75980.graph_	2.52E-24	-1.1451314	down	PREDICTED: GDSL esterase/lipase At2g04570-like
c82970.graph_ c0	6.39E-05	-1.0608931	down	Heat shock 70 kDa protein 12B [Fusarium oxysporum f. sp. cubense race 1]
c87185.graph_ c0	0.0001611	-1.0184095	down	hypothetical protein FPSE_09444 [Fusarium pseudograminearum CS3096]
c88620.graph_ c0	2.36E-07	-1.2752662	down	hypothetical protein FPSE_04201 [Fusarium pseudograminearum CS3096]
c83060.graph_ c1	2.73E-12	-1.0208825	down	LOW QUALITY PROTEIN: berberine bridge enzyme-like 8 [Cajanus cajan]
c95280.graph_	2.84E-92	-4.4721311	down	hypothetical protein BKCO1_610009 [Diplodia corticola]
c86987.graph_	6.16E-150	1.25212271	up	PREDICTED: endonuclease 2 [Glycine max]
c82587.graph_ c0	3.16E-107	1.67834801	up	PREDICTED: protein MOTHER of FT and TFL1 [Lupinus angustifolius]
c96863.graph_	1.93E-13	-1.8699988	down	cytochrome P450 [Meliniomyces bicolor E]
c56385.graph_ c0	0	-2.2632034	down	stearoyl-[acyl-carrier-protein] 9-desaturase 6, chloroplastic-like [Cajanus cajan]
c93465.graph_	6.32E-08	-1.3803739	down	fatty acid synthase alpha subunit [Fusarium equiseti]
c69013.graph_	6.24E-17	-1.3516349	down	PREDICTED: beta-galactosidase-like [Glycine max]
c71677.graph_ c0	6.27E-13	-1.8268452	down	Short-chain dehydrogenase/reductase SDR [Macrophomina phaseolina MS6]
c95534.graph_	4.08E-14	-1.0399209	down	non-specific lipid-transfer protein 1 [Arachis duranensis]
c88637.graph_	1.34E-05	-1.1579118	down	putative ran-specific gtpase-activating protein [Diplodia
c92357.graph_	1.96E-09	-1.5497937	down	hypothetical protein FOXB_13176 [Fusarium oxysporum

c74363.graph_	9.93E-06	-1.1119545	down	conserved unknown protein [Ectocarpus siliculosus]
c76467.graph_ c0	5.92E-05	-1.0765461	down	PREDICTED: elongation of fatty acids protein 3-like [Glycine max]
c71762.graph_	1.07E-35	-3.0257672	down	tall aerial hyphae-3 [Diaporthe helianthi]
c36775.graph_	2.29E-13	-1.8615061	down	putative mfs transporter [Diaporthe ampelina]
c85695.graph_ c0	2.33E-26	1.14903198	up	PREDICTED: chaperone protein ClpC, chloroplastic-like [Glycine max]
c54741.graph_	8.23E-07	-1.0063723	down	hypothetical protein PHAVU_001G050300g [Phaseolus
c84536.graph_	6.56E-13	1.8131985	up	hypothetical protein PHAVU_007G070400g [Phaseolus
c78543.graph_ c1	3.09E-31	1.07269323	up	non-specific lipid-transfer protein-like protein At5g64080 [Cajanus cajan]
c78256.graph_ c0	2.44E-09	1.53874928	up	glyceraldehyde-3-phosphate dehydrogenase A, chloroplastic [Arachis ipaensis]
c54578.graph_	1.39E-61	1.40828729	up	PREDICTED: vestitone reductase-like isoform X2
c40248.graph_	1.68E-14	-1.9499159	down	putative hlh transcription factor [Diaporthe ampelina]
c57117.graph_	3.66E-23	-2.480327	down	mfs aflatoxin efflux [Diplodia corticola]
c83167.graph_ c0	3.29E-14	-1.9321407	down	putative nadp-dependent alcohol dehydrogenase 6 [Diplodia seriata]
c76035.graph_	1.51E-89	3.26368496	up	desiccation protectant protein Lea14 homolog [Cajanus
c57734.graph_	1.87E-24	-2.5451348	down	hypothetical protein DHEL01_v200533 [Diaporthe
c70203.graph_	4.93E-142	4.87821769	up	PREDICTED: P24 oleosin isoform A-like [Cicer
c85349.graph_	6.01E-05	1.07606028	up	hypothetical protein PHAVU_007G181400g [Phaseolus
c77457.graph_	5.49E-15	-1.9856527	down	malic enzyme [Diaporthe helianthi]
c37873.graph_ c0	1.74E-05	-1.1143045	down	component of cytosolic 80S ribosome and 60S large subunit [Volvox carteri f. nagariensis]
c95712.graph_	4.18E-15	-1.9942224	down	TPR domain-containing protein [Diaporthe helianthi]
c86469.graph_	4.79E-31	-2.8440308	down	putative glyoxalase family protein [Diaporthe ampelina]
c64981.graph_ c0	5.68E-10	-1.5947977	down	carbohydrate-binding module family 21 protein [Diplodia corticola]
c69514.graph_	2.95E-58	-3.719794	down	hypothetical protein DHEL01_v201796 [Diaporthe
c78953.graph_ c0	1.22E-06	1.061965	up	PREDICTED: desiccation-related protein PCC13-62-like [Glycine max]
c68684.graph_	1.89E-22	-1.1407589	down	hypothetical protein GLYMA_11G070200 [Glycine max]
c90600.graph_	7.37E-13	1.62010219	up	PREDICTED: transcription factor HBP-1b(c38) [Glycine
c83995.graph_	9.75E-35	-1.0730046	down	2-aminoethanethiol dioxygenase-like protein [Trifolium
c37849.graph_	6.43E-51	-2.9626608	down	hypothetical protein PHAVU_007G222900g [Phaseolus
c91773.graph_ c1	2.48E-33	-1.651886	down	hypothetical protein PHAVU_001G0393001g, partial [Phaseolus vulgaris]
c68419.graph_	2.54E-47	-1.9280808	down	peroxidase N1-like isoform X1 [Durio zibethinus]
c82701.graph_	1.21E-29	2.60638895	up	uncharacterized protein LOC100306653 [Glycine max]
c74544.graph_	3.50E-06	1.10551096	up	unknown [Glycine max]
c90599.graph_	3.75E-20	1.44877753	up	probable protein phosphatase 2C 8 [Cajanus cajan]
c85972.graph_ c0	2.24E-35	-2.468781	down	hypothetical protein MVLG_03475 [Microbotryum lychnidis-dioicae p1A1 Lamole]
c76177.graph_	2.89E-126	-1.370801	down	hypothetical protein GLYMA_11G070500 [Glycine max]
c96325.graph_	2.05E-15	-2.0159557	down	hypothetical protein DHEL01_v202661 [Diaporthe
c89530.graph_ c0	7.16E-285	3.7380568	up	PREDICTED: late embryogenesis abundant protein 2-like [Glycine max]
c79050.graph_	7.49E-17	-2.0872817	down	c2h2 finger domain [Diplodia corticola]
c69003.graph_	3.43E-32	1.00162599	up	trypsin inhibitor [Apios americana]

c37812.graph_c0	1.04E-37	-3.1013112	down	hypothetical protein M434DRAFT_22549 [Hypoxylon sp. CO27-5]
c91625.graph_c0	6.98E-06	1.02815353	up	putative inactive cysteine synthase 2 isoform X2 [Vigna radiata var. radiata]
c82026.graph_c0	1.68E-17	-2.1552878	down	hypothetical protein NECHADRAFT_39694 [[Nectria] haematococca mpVI 77-13-4]
c88859.graph_c0	6.88E-12	1.33482183	up	PREDICTED: U-box domain-containing protein 8-like [Glycine max]
c88102.graph_	8.80E-19	-1.8483087	down	hypothetical protein PHAVU_009G068100g [Phaseolus
c86916.graph_c0	1.10E-07	-1.1104744	down	probable woronin body major protein precursor [Fusarium proliferatum]
c55588.graph_	3.65E-19	-2.244771	down	glycoside hydrolase family 28 protein [Diplodia corticola]
c77248.graph_c3	2.80E-07	1.14990234	up	PREDICTED: uncharacterized protein LOC100806455 [Glycine max]
c95159.graph_c0	1.58E-05	1.00302291	up	disease resistance protein (TIR-NBS-LRR class), putative [Medicago truncatula]
c81550.graph_	3.06E-06	1.22593939	up	NEDD8 ultimate buster 1 [Arachis ipaensis]
c83101.graph_	1.50E-08	-1.4697585	down	putative glucan endo-alpha-glucosidase agn1 [Diplodia
c83664.graph_	4.62E-06	-1.2068558	down	elongation factor 3 [Fusarium langsethiae]
c70014.graph_	1.40E-06	-1.2613839	down	alcohol oxidase [Diplodia corticola]
c85447.graph_	5.36E-15	-1.9858533	down	quinone oxidoreductase [Diaporthe helianthi]
c87610.graph_	2.46E-18	-1.8319471	down	histone h2b [Diplodia corticola]
c89595.graph_	1.21E-06	-1.0404695	down	Protein neuralized [Glycine soja]
c93662.graph_	3.04E-72	-1.6572572	down	hypothetical protein FPOA_04480 [Fusarium poae]
c57861.graph_	3.53E-11	-1.7002353	down	hypothetical protein BKCO1_600094 [Diplodia corticola]
c76025.graph_c0	3.04E-15	1.22568154	up	probable inactive 2-oxoglutarate-dependent dioxygenase AOP2 [Cajanus cajan]
c56186.graph_	2.53E-16	-2.0779685	down	o-methyltransferase protein [Diplodia corticola]
c77660.graph_	2.54E-09	-1.5054738	down	hypothetical protein DHEL01_v208488 [Diaporthe
c91206.graph_c0	2.55E-06	-1.2080926	down	putative ADP-ribosylation factor, partial [Tolypocladium capitatum]
c88097.graph_	2.86E-17	-2.0243013	down	unnamed protein product [Fusarium venenatum]
c82703.graph_	3.53E-07	-1.2885985	down	uncharacterized protein FMAN_07494 [Fusarium
c78272.graph_	6.50E-05	-1.0709423	down	copper fist dna binding domain protein [Diplodia corticola]
c95674.graph_	2.02E-56	-3.6890236	down	linoleate diol synthase [Diaporthe helianthi]
c90591.graph_	2.50E-39	1.8381514	up	stem-specific protein TSJT1-like isoform X2 [Cajanus
c82403.graph_	1.02E-10	-1.661131	down	eukaryotic aspartyl protease [Diaporthe helianthi]
c92060.graph_c0	1.06E-09	-1.479622	down	mitochondrial phosphate carrier protein [Aschersonia aleyrodis RCEF 2490]
c93660.graph_c0	7.91E-07	-1.282592	down	ABC transporter ATP-binding protein ARB1 [Fusarium verticillioides 7600]
c95550.graph_	6.36E-09	-1.0919049	down	hypothetical protein PHAVU_001G019300g [Phaseolus
c38109.graph_	5.74E-15	-1.9838484	down	putative synaptobrevin [Diaporthe ampelina]
c76068.graph_c0	2.93E-08	-1.4064157	down	ketol-acid reductoisomerase, mitochondrial [Fusarium verticillioides 7600]
c79026.graph_c0	4.51E-15	-1.0202647	down	hypothetical protein VIGAN_08009100 [Vigna angularis var. angularis]
c90948.graph_c0	3.49E-21	-1.9654341	down	ATP-dependent RNA helicase eIF4A [Colletotrichum chlorophyti]
c78554.graph_c0	8.82E-19	-2.1090852	down	putative phytanoyl- dioxygenase protein [Neofusicoccum parvum UCRNP2]
c74611.graph_	1.69E-106	-1.1862535	down	Non-symbiotic hemoglobin 1 [Glycine soja]

c85678.graph_	3.08E-05	1.0749559	up	uncharacterized protein LOC109798425 [Cajanus cajan]
c75426.graph_	3.80E-27	-1.9278492	down	U-box domain-containing protein [Glycine max]
c94463.graph_ c3	7.23E-48	-2.7520028	down	related to PHO89-Na+/phosphate co-transporter [Fusarium fujikuroi IMI 58289]
c92537.graph_	5.22E-39	-2.2311518	down	putative subtilisin-like proteinase spm1 [Diaporthe
c37989.graph_	1.11E-06	-1.2683552	down	uncharacterized protein LOC109795393 [Cajanus cajan]
c96966.graph_	1.14E-13	-1.8877687	down	glycogen phosphorylase [Diaporthe helianthi]
c73753.graph_	1.96E-16	1.95502295	up	bZIP transcription factor bZIP132 [Glycine max]
c92323.graph_ c0	4.89E-09	1.48811414	up	PREDICTED: uncharacterized protein LOC100815148 [Glycine max]
c76496.graph_	2.99E-54	-1.2976082	down	caffeic acid O-methyltransferase [Medicago truncatula]
c95238.graph_	2.79E-16	-1.3328133	down	PREDICTED: protein YLS9-like [Vigna angularis]
c78624.graph_	5.17E-18	-2.117921	down	General substrate transporter [Macrophomina phaseolina
c87364.graph_	1.58E-14	-1.8894409	down	Plasma membrane fusion protein prm1 [Diplodia seriata]
c90989.graph_ c0	1.69E-08	-1.1339687	down	hypothetical protein FOMG_06714 [Fusarium oxysporum f. sp. melonis 26406]
c74493.graph_	4.26E-16	-2.0590772	down	putative gpi anchored cell wall protein [Diplodia seriata]
c92014.graph_	6.87E-15	-1.9550473	down	bzip transcription factor [Diplodia corticola]
c36704.graph_	2.68E-12	-1.7739583	down	hypothetical protein DHEL01_v206999 [Diaporthe
c94067.graph_	5.83E-05	1.07749878	up	unknown [Zea mays]
c74635.graph_	4.45E-06	1.20587299	up	uncharacterized protein LOC109799075 [Cajanus cajan]
c90497.graph_	1.33E-47	1.24250188	up	GDSL esterase/lipase At3g26430-like [Cajanus cajan]
c95089.graph_	4.02E-43	2.42610842	up	putative mitochondrial protein [Apostasia shenzhenica]
c82109.graph_	2.35E-23	2.34779361	up	GDSL esterase/lipase 1-like [Cajanus cajan]
c70775.graph_	0	4.06009656	up	unknown [Lotus japonicus]
c86342.graph_	3.66E-48	1.92303183	up	GDSL esterase/lipase [Glycine soja]
c89227.graph_	4.17E-19	1.22599547	up	anoctamin-like protein At1g73020 isoform X1 [Cajanus
c97081.graph_	7.01E-10	-1.5613806	down	hypothetical protein DHEL01_v205311 [Diaporthe
c38026.graph_ c0	6.48E-48	-1.1227179	down	PREDICTED: uncharacterized protein LOC100776765 [Glycine max]
c42611.graph_	7.96E-15	-1.973872	down	hypothetical protein FNYG_13792 [Fusarium nygamai]
c37226.graph_ c0	5.81E-12	-1.7496592	down	conserved hypothetical protein [Talaromyces marneffei ATCC 18224]
c37476.graph_	4.49E-10	-1.6082326	down	hypothetical protein BK809_0004686 [Diplodia seriata]
c85617.graph_ c0	6.55E-29	2.57761737	up	PREDICTED: uncharacterized protein LOC108324663 [Vigna angularis]
c92097.graph_	2.74E-09	-1.3958033	down	hypothetical protein TSUD_07620 [Trifolium
c80136.graph_	1.70E-58	-2.1364879	down	PREDICTED: basic 7S globulin-like [Vigna angularis]
c84651.graph_	1.93E-14	-1.6313648	down	hypothetical protein FG05_09286 [Fusarium graminearum]
c36973.graph_	9.80E-18	-2.1691932	down	cytochrome p450 [Diplodia corticola]
c89818.graph_	2.15E-06	-1.184755	down	putative pyruvate kinase [Fusarium fujikuroi]
c74454.graph_	3.15E-08	-1.412395	down	40S ribosomal protein S20 [Diaporthe helianthi]
c90333.graph_	1.13E-167	-3.2238062	down	putative esdc-like protein [Diaporthe ampelina]
c78163.graph_	1.14E-18	-2.2280466	down	hypothetical protein DHEL01_v204253 [Diaporthe
c74796.graph_	1.55E-06	1.2283721	up	Dehydration-responsive protein RD22 [Glycine soja]
c36746.graph_	1.52E-10	-1.6302752	down	Beta-glucosidase 1B [Diplodia seriata]
c79754.graph_	3.30E-24	-2.4991828	down	unnamed protein product [Fusarium sp. FIESC_5 CS3069]
c89753.graph_ c0	1.61E-86	1.8850619	up	PREDICTED: probable 1-aminocyclopropane-1-carboxylate oxidase [Glycine max]

c80611.graph_c0	1.20E-16	-2.099759	down	putative oxalate decarboxylase family bicupin [Diaporthe ampelina]
c83308.graph_	3.14E-08	-1.3900762	down	putative glutamine synthetase [Diaporthe ampelina]
c92405.graph_	2.41E-07	-1.3025486	down	hypothetical protein FOXB_16126 [Fusarium oxysporum
c89158.graph_c0	5.78E-13	-1.1060953	down	PREDICTED: probable indole-3-acetic acid-amido synthetase GH3.5 isoform X1 [Glycine max]
c38224.graph_c0	1.01E-27	-2.6992004	down	hypothetical protein P174DRAFT_407796 [Aspergillus novofumigatus IBT 16806]
c93743.graph_	1.42E-08	-1.2916289	down	hypothetical protein FOXB_08643 [Fusarium oxysporum
c91424.graph_c0	7.56E-42	1.13603992	up	PREDICTED: zinc finger CCCH domain-containing protein 2-like [Glycine max]
c55560.graph_c0	1.53E-05	-1.1272804	down	predicted protein, partial [Phaeodactylum tricornutum CCAP 1055/1]
c76468.graph_	9.52E-08	-1.0111205	down	PR1a precursor [Glycine max]
c87174.graph_	3.39E-05	-1.1072188	down	unnamed protein product [Fusarium venenatum]
c67934.graph_	3.25E-05	-1.1089614	down	putative oligopeptidase family protein [Diplodia seriata]
c91400.graph_c1	9.05E-26	-1.2037039	down	CBL-interacting serine/threonine-protein kinase 3-like isoform X2 [Cajanus cajan]
c90342.graph_c0	2.80E-42	2.91829667	up	Mitochondrial substrate carrier family protein ancA [Paramicrosporidium saccamoebae]
c86833.graph_c0	1.30E-07	1.24447634	up	PREDICTED: uncharacterized protein LOC108342196 [Vigna angularis]
c55707.graph_	2.55E-86	-4.3486343	down	nitrate transporter [Diaporthe helianthi]
c76619.graph_	4.31E-64	-1.9281862	down	unknown [Lotus japonicus]
c73359.graph_	2.78E-27	-2.6804013	down	POT family protein [Diaporthe helianthi]
c76039.graph_	2.51E-14	-1.6990501	down	hypothetical protein PHAVU_003G256600g [Phaseolus
c95848.graph_	5.15E-23	-2.4716426	down	putative aldehyde dehydrogenase-like protein [Diplodia
c91020.graph_	3.64E-08	-1.2393066	down	hypothetical protein FVEG_04839 [Fusarium
c92541.graph_c0	5.43E-80	1.71704424	up	poly(ADP-ribose) polymerase domain protein [Medicago truncatula]
c86350.graph_	3.75E-06	-1.221519	down	unnamed protein product [Fusarium venenatum]
c89925.graph_c2	2.11E-05	-1.0096708	down	hypothetical protein BSLG_04848 [Batrachochytrium salamandrivorans]
c95205.graph_c0	2.66E-57	1.60573443	up	Retrovirus-related Pol polyprotein from transposon TNT 1-94 [Cajanus cajan]
c83659.graph_	1.07E-34	-2.987726	down	putative amid-like nadh [Diaporthe ampelina]
c38393.graph_	1.71E-24	-2.5510812	down	E3 ubiquitin-protein ligase hulA [Diaporthe helianthi]
c70779.graph_	1.57E-11	-1.0387651	down	probable carboxylesterase 12 [Cajanus cajan]
c85758.graph_c0	1.52E-06	-1.244317	down	40S ribosomal protein S26E [Fusarium oxysporum f. sp. lycopersici 4287]
c79072.graph_	1.97E-18	-2.2137485	down	oligopeptide transporter [Diaporthe helianthi]
c71079.graph_	1.75E-19	-1.2728482	down	dirigent protein 22-like [Vigna radiata var. radiata]
c94173.graph_	7.99E-69	1.07126811	up	uncharacterized protein LOC109813966 [Cajanus cajan]
c87245.graph_	8.68E-12	-1.128811	down	hypothetical protein PHAVU_004G058100g [Phaseolus
c70306.graph_c0	2.62E-09	-1.5216891	down	hypothetical protein MPH_10408 [Macrophomina phaseolina MS6]
c67447.graph_	3.72E-12	-1.764765	down	hypothetical protein FAVG1_08673 [Fusarium
c91979.graph_	9.93E-06	1.15438276	up	hypothetical protein FLAG1_10626 [Fusarium langsethiae]
c83612.graph_	2.09E-20	-1.2166128	down	kinesin-like protein NACK1 [Cajanus cajan]
c76846.graph_	2.09E-27	-2.6772574	down	Exopolygalacturonase X-1 [Diplodia seriata]
c87595.graph_	3.48E-40	1.15995691	up	hypothetical protein PHAVU_011G136400g [Phaseolus

c76229.graph_	1.14E-12	-1.815098	down	superoxide dismutase [Diplodia corticola]
c94568.graph_	2.62E-11	-1.7081288	down	1a protein [Cucumber mosaic virus]
c38374.graph_	5.17E-16	-2.0575327	down	hypothetical protein DHEL01_v210039 [Diaporthe [illegible]
c94987.graph_ c0	7.93E-46	1.19977884	up	LOW QUALITY PROTEIN: amino acid permease 4-like [Arachis ipaensis]
c93944.graph_	9.60E-143	1.49002809	up	probable aquaporin TIP-type alpha [Cajanus cajan]
c85618.graph_	2.15E-06	1.01574851	up	sugar porter (SP) family MFS transporter [Medicago [illegible]
c75460.graph_	4.25E-08	1.27705715	up	Gibberellin 2-beta-dioxygenase 2 [Glycine soja]
c78621.graph_	2.11E-10	-1.6247453	down	microsomal cytochrome b5 [Diaporthe helianthi]
c81350.graph_	2.85E-17	-1.9174131	down	Protein ecm33, partial [Diplodia seriata]
c77068.graph_	2.47E-07	1.23461281	up	hypothetical protein B456_008G175600 [Gossypium [illegible]
c37037.graph_	5.04E-11	-1.6671509	down	putative pci domain-containing protein [Diaporthe [illegible]
c37710.graph_ c0	1.25E-69	-3.9980443	down	Uncharacterized protein T310_8106 [Rasamsonia emersonii CBS 393.64]
c99526.graph_	4.72E-08	-1.3938622	down	hypothetical protein BKCO1_600037 [Diplodia corticola]
c39648.graph_	1.05E-11	-1.725691	down	hypothetical protein DHEL01_v200043 [Diaporthe [illegible]
c53938.graph_	2.05E-56	-3.676405	down	hypothetical protein DHEL01_v209322 [Diaporthe [illegible]
c37839.graph_ c0	4.53E-18	-1.1723336	down	PREDICTED: probably inactive leucine-rich repeat receptor-like protein kinase At5g48380 [Vigna angularis]
c89203.graph_ c0	1.03E-07	1.16860356	up	PREDICTED: calcium uniporter protein 4, mitochondrial [Cicer arietinum]
c89509.graph_ c0	8.83E-152	3.09327915	up	uncharacterized protein LOC109793247 isoform X2 [Cajanus cajan]
c95374.graph_	5.77E-13	-1.0449939	down	PREDICTED: basic 7S globulin 2-like, partial [Glycine [illegible]
c87665.graph_	1.41E-18	1.32272894	up	ABC transporter G family member 15-like [Cajanus cajan]
c85921.graph_	6.74E-05	-1.0632341	down	40S ribosomal protein S0 [Fusarium verticillioides 7600]
c64040.graph_	1.35E-05	-1.1458678	down	PREDICTED: thaumatin-like protein 1b isoform X2 [illegible]
c66282.graph_	8.61E-17	-2.1091528	down	kelch repeat protein [Diaporthe helianthi]
c90872.graph_	9.93E-27	1.58676193	up	cytochrome P450 93A3-like [Cajanus cajan]
c96147.graph_	2.31E-24	-2.5409065	down	catalase/peroxidase HPI [Diaporthe helianthi]
c36752.graph_	8.45E-20	-2.293604	down	putative cytochrome p450 [Diplodia seriata]
c59007.graph_ c0	5.83E-12	-1.7577431	down	putative tripeptidyl-peptidase 1 precursor protein [Neofusicoccum parvum UCRNP2]
c95821.graph_	2.35E-16	-2.0800127	down	hypothetical protein DHEL01_v204504 [Diaporthe [illegible]
c83731.graph_	3.82E-05	-1.0916019	down	similar to SNARE protein Snc2 [Leptosphaeria maculans [illegible]
c88661.graph_ c0	0.0001088	1.03583484	up	PREDICTED: ethylene-responsive transcription factor 1B-like [Glycine max]
c84717.graph_	1.89E-06	-1.2337554	down	uncharacterized protein FPRN_01157 [Fusarium [illegible]
c38503.graph_ c0	7.66E-12	-1.739351	down	S-adenosyl-L-methionine-dependent methyltransferase [Glonium stellatum]
c94105.graph_ c1	6.65E-23	-1.0945932	down	probably inactive leucine-rich repeat receptor-like protein kinase IMK2 [Sesamum indicum]
c83516.graph_ c0	2.73E-21	-2.3724818	down	MFS transporter, SP family, general alpha glucoside:H+ symporter [Diaporthe helianthi]
c38886.graph_	2.39E-06	-1.0675441	down	predicted protein [Hordeum vulgare subsp. vulgare]
c95520.graph_	5.17E-11	-1.6843191	down	aldo keto reductase [Diplodia corticola]
c92413.graph_ c0	3.29E-56	3.19315024	up	PREDICTED: uncharacterized protein LOC100777508 isoform X4 [Glycine max]
c82098.graph_	2.37E-14	-1.0732274	down	PREDICTED: lipase 1-like isoform X1 [Glycine max]
c81718.graph_ c0	4.13E-08	-1.4183666	down	PREDICTED: transcription factor CPC-like isoform X1 [Glycine max]

c78830.graph_	3.47E-23	-2.2903063	down	woronin body major protein [Diplodia corticola]
c74449.graph_	4.97E-84	4.26145701	up	chitinase homologue [Sesbania rostrata]
c37939.graph_	4.88E-55	-3.2771111	down	hypothetical protein BKCO1_5000209 [Diplodia corticola]
c89534.graph_ c0	6.08E-14	-1.6906251	down	PREDICTED: uncharacterized protein LOC100778228 [Glycine max]
c89766.graph_ c1	7.44E-08	-1.3733026	down	putative 60s ribosomal protein l7 protein [Neofusicoccum parvum UCRNP2]
c90982.graph_	7.75E-07	1.28339455	up	hypothetical protein TSUD_312440 [Trifolium
c84987.graph_ c0	1.86E-05	1.10462376	up	organic cation/carnitine transporter-like protein [Medicago truncatula]
c38447.graph_	1.28E-11	-1.7198938	down	cfem domain-containing protein [Diplodia corticola]
c86868.graph_	1.41E-112	-1.1449131	down	chalcone synthase [Trifolium pratense]
c74304.graph_	1.17E-09	-1.5609369	down	putative amino-acid permease inda1 [Diplodia seriata]
c71505.graph_ c0	2.75E-12	1.1527655	up	hypothetical protein VIGAN_04390300 [Vigna angularis var. angularis]
c91465.graph_	3.59E-11	-1.3359688	down	hypothetical protein glysoja_007873 [Glycine soja]
c84028.graph_	4.21E-07	-1.2885512	down	b-zip transcription factor idi-4 [Diplodia corticola]
c68801.graph_	3.79E-11	-1.0391357	down	hypothetical protein GLYMA_10G283700 [Glycine max]
c38333.graph_	3.99E-23	-2.4775154	down	non-anchored cell wall protein-1 [Diaporthe helianthi]
c92343.graph_	1.96E-14	1.40405726	up	PREDICTED: cytochrome P450 94A1 [Glycine max]
c37865.graph_	8.40E-28	2.70514308	up	Ty3/gypsy retrotransposon protein [Trifolium pratense]
c36744.graph_	9.63E-13	-1.8137473	down	hypothetical protein DHEL01_v210839 [Diaporthe
c60438.graph_	7.90E-12	-1.7381016	down	Chitin synthase D [Diplodia seriata]
c40185.graph_	2.34E-11	-1.697352	down	aromatic amino acid aminotransferase [Diaporthe helianthi]
c95895.graph_	8.05E-16	-2.0442927	down	oxalate decarboxylase oxdC [Diaporthe helianthi]
c54619.graph_	3.50E-07	-1.3260826	down	hypothetical protein BKCO1_5200072 [Diplodia corticola]
c88434.graph_ c0	4.13E-08	-1.3633253	down	probable NAD binding Rossmann fold oxidoreductase [Fusarium mangiferae]
c97248.graph_	2.90E-11	-1.6878048	down	hypothetical protein DHEL01_v206822 [Diaporthe

Cluster_ID	FDR	log2FC	regulated	nr_annotation
c40224.graph_c0	3.03E-12	-1.9113	down	putative transmembrane alpha-helix domain-contai
c92264.graph_c0	4.15E-56	-1.6129	down	ABI-3 homolog [Psophocarpus tetragonolobus]
c82188.graph_c0	1.59E-35	-3.2144	down	pathogenesis associated protein Cap20, putative [M
c37669.graph_c0	6.53E-43	-3.485	down	versicolorin reductase [Aspergillus udagawae]
c91337.graph_c2	1.24E-07	1.30186	up	uncharacterized protein LOC109793102 [Cajanus c
c76828.graph_c0	3.20E-13	1.03196	up	U-box domain-containing protein 25-like [Cajanus
c57963.graph_c0	4.14E-17	-2.2637	down	putative multicopper oxidase type 1 [Diplodia seria
c73894.graph_c0	2.79E-41	2.53619	up	glycoside hydrolase family 1 protein [Medicago tru
c82392.graph_c1	1.44E-07	1.01428	up	Phosphatidylinositol-4-phosphate 5-kinase 9 [Glyci
c79418.graph_c0	3.52E-35	1.07078	up	PREDICTED: alanine--glyoxylate aminotransferase
c76417.graph_c0	1.05E-42	-2.0814	down	hypothetical protein KK1_033668 [Cajanus cajan]
c52452.graph_c0	1.76E-26	-2.8265	down	STE like transcription factor [Diaporthe helianthi]
c88231.graph_c0	3.86E-05	-1.1671	down	retrotransposon-related protein, partial [Trifolium p
c38035.graph_c0	3.48E-21	-2.5432	down	hypothetical protein DHEL01_v200180 [Diaporthe
c76247.graph_c0	1.59E-53	-1.4188	down	unknown [Glycine max]
c38123.graph_c0	1.30E-24	-2.7311	down	hypothetical protein UCDDS831_g02644 [Diplodia
c95361.graph_c0	2.49E-09	-1.6372	down	putative polyketide synthase protein [Neofusicoccu
c87615.graph_c0	3.76E-11	-1.8073	down	hypothetical protein BK809_0004349 [Diplodia ser
c98390.graph_c0	2.84E-10	-1.7263	down	hypothetical protein UCDDA912_g00683 [Diaport
c94301.graph_c1	1.39E-12	1.51741	up	predicted protein [Hordeum vulgare subsp. vulgare]
c75339.graph_c0	#######	1.48802	up	PREDICTED: dirigent protein 22-like [Glycine ma
c89930.graph_c0	2.48E-10	1.16221	up	PREDICTED: uncharacterized protein LOC109363
c71104.graph_c0	3.06E-45	1.6169	up	hypothetical protein PHAVU_009G158100g [Phase
c76838.graph_c0	2.78E-10	1.05587	up	PREDICTED: glycine cleavage system H protein, n
c58605.graph_c0	1.98E-18	-2.375	down	hypothetical protein M426DRAFT_13512 [Hypoxy
c37683.graph_c0	4.03E-06	-1.2108	down	glutamine synthetase [Fusarium oxysporum f. sp. ly
c83968.graph_c0	6.66E-15	-1.6089	down	transaldolase [Fusarium langsethiae]
c77657.graph_c0	6.69E-12	-1.8868	down	hypothetical protein BKCO1_1300023 [Diplodia co
c40010.graph_c0	7.28E-41	-3.4137	down	hypothetical protein DHEL01_v202423 [Diaporthe
c53676.graph_c0	7.57E-06	-1.207	down	hypothetical protein FPSE_02544 [Fusarium pseud
c85365.graph_c0	5.28E-08	1.0452	up	uncharacterized protein LOC106761173 [Vigna rad
c87840.graph_c3	2.26E-09	1.30719	up	Putative WRKY transcription factor 42 [Glycine so
c53718.graph_c0	1.44E-20	-1.8523	down	PREDICTED: protein DETOXIFICATION 49-like
c69304.graph_c0	9.82E-15	-1.5462	down	geraniol 8-hydroxylase-like [Cajanus cajan]
c76633.graph_c0	7.99E-25	-1.9364	down	alpha-dioxygenase 1 [Cajanus cajan]
c92428.graph_c0	#######	1.00881	up	prepropulchellin IV [Abrus pulchellus subsp. tenuif
c98462.graph_c0	5.65E-10	-1.7113	down	tpr repeat protein oca3 protein [Diplodia corticola]
c37859.graph_c0	3.23E-06	1.31118	up	14 kDa proline-rich protein DC2.15 [Arachis duran
c76573.graph_c0	1.77E-43	1.36374	up	aquaporin TIP2;1 [Robinia pseudoacacia]
c58193.graph_c0	7.00E-06	-1.2048	down	elongation factor EF-3 [Ectocarpus siliculosus]
c80067.graph_c0	9.50E-12	1.87334	up	laccase, multicopper oxidase, benzenediol:oxygen c
c95641.graph_c0	1.77E-06	1.1219	up	hypothetical protein LR48_Vigan303s004900 [Vig
c86155.graph_c0	0.0003	1.0359	up	hypothetical protein PHAVU_003G293900g [Phase
c78816.graph_c0	5.79E-10	1.20586	up	hypothetical protein PHAVU_006G181100g [Phase
c84130.graph_c0	1.44E-45	1.2096	up	hypothetical protein LR48_Vigan09g210200 [Vign

c94181.graph_c1	1.57E-57	-1.2355	down	hypothetical protein [Lotus japonicus]
c79471.graph_c0	6.95E-09	-1.5172	down	uncharacterized protein LOC109797106 isoform X[illegible]
c76091.graph_c0	1.45E-12	1.03998	up	unknown [Glycine max]
c77869.graph_c0	1.96E-10	-1.7426	down	putative dicarboxylic amino acid permease [Diplod[illegible]
c97072.graph_c0	1.29E-10	-1.7661	down	putative extracellular exo [Diplodia seriata]
c91769.graph_c0	7.60E-10	1.69542	up	Proprotein convertase subtilisin/kexin type 5 [Rhizo[illegible]
c97022.graph_c0	1.10E-09	-1.6862	down	putative lipase 2 [Diplodia seriata]
c69329.graph_c0	3.50E-18	1.84414	up	uncharacterized protein LOC109787824 [Cajanus c[illegible]
c88864.graph_c0	7.82E-08	-1.0691	down	putative pectate lyase 2 [Cajanus cajan]
c82041.graph_c0	1.36E-52	2.43871	up	PREDICTED: caffeic acid 3-O-methyltransferase-li[illegible]
c95475.graph_c0	3.23E-28	-1.275	down	uncharacterized protein LOC109809639 [Cajanus c[illegible]
c91323.graph_c1	2.37E-65	-4.1296	down	hypothetical protein DHEL01_v206890 [Diaporthe [illegible]
c89177.graph_c1	4.12E-17	-1.2404	down	PREDICTED: WD repeat-containing protein 44-lik[illegible]
c79334.graph_c0	4.92E-11	1.80835	up	hypothetical protein FNYG_11322 [Fusarium nyga[illegible]
c84756.graph_c0	1.45E-12	1.93893	up	macrofage activating glycoprotein, putative [Rhizo[illegible]
c94507.graph_c0	7.89E-26	2.17091	up	PREDICTED: probable disease resistance protein [illegible]
c89905.graph_c5	#######	4.57806	up	germin family protein [Medicago truncatula]
c80161.graph_c1	2.66E-25	-2.5853	down	acetate-CoA ligase [Diaporthe helianthi]
c91695.graph_c0	6.51E-12	-1.0671	down	hypothetical protein GLYMA_08G071200 [Glycin[illegible]
c86601.graph_c0	5.28E-09	1.44694	up	PREDICTED: type IV inositol polyphosphate 5-ph[illegible]
c73735.graph_c0	4.22E-29	-2.9468	down	putative riboflavin transporter mch5 [Diaporthe am[illegible]
c68689.graph_c0	2.37E-14	-2.079	down	putative transcriptional regulatory protein C15D4.0[illegible]
c95562.graph_c0	1.85E-15	-2.1703	down	NADPH-dependent FMN reductase [Diaporthe heli[illegible]
c92950.graph_c5	4.34E-26	-2.0778	down	alkaline alpha-galactosidase [Pisum sativum]
c76008.graph_c0	8.22E-26	2.03759	up	PREDICTED: uncharacterized protein LOC108340[illegible]
c94974.graph_c0	3.06E-53	-1.3677	down	Transposon Ty3-G Gag-Pol polyprotein [Cajanus c[illegible]
c92799.graph_c0	5.96E-09	-1.61	down	related to kynureninase [Fusarium mangiferae]
c56489.graph_c1	9.52E-10	-1.6713	down	gnat family [Diplodia corticola]
c73621.graph_c0	2.55E-05	-1.0181	down	PREDICTED: uncharacterized protein LOC108328[illegible]
c54063.graph_c0	1.12E-58	-3.9351	down	putative glycosyl transferase family 2 protein [Neof[illegible]
c76909.graph_c0	1.58E-29	-2.9654	down	hypothetical protein CGGC5_8688 [Colletotrichum[illegible]
c88288.graph_c0	5.91E-05	1.06975	up	predicted protein [Hordeum vulgare subsp. vulgare[illegible]
c37789.graph_c1	1.28E-12	-1.9438	down	plasma membrane ATPase-1 [Diaporthe helianthi]
c73394.graph_c0	3.26E-17	-1.033	down	protein STRICTOSIDINE SYNTHASE-LIKE 2-lik[illegible]
c55527.graph_c1	0.00018	1.00406	up	PREDICTED: transcription factor MYB1R1-like is[illegible]
c88524.graph_c0	2.10E-23	1.17857	up	PREDICTED: transaldolase [Glycine max]
c79549.graph_c0	5.76E-06	-1.2776	down	calcium calmodulin-dependent protein [Diplodia c[illegible]
c57051.graph_c0	2.63E-24	-2.7128	down	putative mfs multidrug transporter [Diaporthe ampe[illegible]
c76181.graph_c0	4.02E-07	-1.0486	down	hypothetical protein TSUD_113300 [Trifolium sub[illegible]
c95931.graph_c0	3.60E-19	-2.422	down	putative 2og-fe oxygenase [Diaporthe ampelina]
c86116.graph_c0	7.57E-05	1.11561	up	hypothetical protein GLYMA_U019600 [Glycine m[illegible]
c73872.graph_c0	5.51E-11	-1.7957	down	cell pattern formation-associated protein stuA [Dia[illegible]
c78580.graph_c0	6.53E-32	2.49289	up	polygalacturonase PG1 precursor [Glycine max]
c56936.graph_c0	7.20E-12	-1.8841	down	Bifunctional protein GAL10 [Diplodia seriata]
c37760.graph_c0	5.32E-13	-1.9761	down	putative secreted protein [Diaporthe ampelina]
c95493.graph_c0	2.49E-43	1.55835	up	uncharacterized protein LOC100527699 [Glycine m[illegible]

c92006.graph_c0	4.47E-21	-1.8832	down	short chain dehydrogenase [Diaporthe helianthi]
c89248.graph_c1	1.24E-18	1.85572	up	PREDICTED: polyphenol oxidase A1, chloroplasti
c37515.graph_c0	3.79E-14	-2.0699	down	hypothetical protein UCDDA912_g05724 [Diaport
c90716.graph_c1	3.86E-19	1.10828	up	hypothetical protein VIGAN_02037600 [Vigna ang
c89352.graph_c0	1.23E-69	-1.3081	down	PREDICTED: transcriptional corepressor LEUNIG
c38016.graph_c0	5.90E-16	-2.1232	down	putative apoptosis-inducing factor 1 [Diplodia seria
c96249.graph_c0	3.55E-15	-2.149	down	putative amidase [Hypoxylon sp. EC38]
c91922.graph_c0	1.35E-10	-1.6148	down	hypothetical protein FPOA_08123 [Fusarium poae]
c93783.graph_c2	7.17E-23	1.14365	up	NADH dehydrogenase subunit 9 [Brassica napus]
c87041.graph_c0	0.0002	1.02783	up	U-box domain-containing protein 19-like [Cajanus
c47840.graph_c0	2.07E-35	-3.2153	down	amino-acid permease inda1 [Diaporthe helianthi]
c83308.graph_c2	2.42E-15	-2.1616	down	glutamine synthetase [Diaporthe helianthi]
c96318.graph_c0	1.84E-14	-2.0946	down	cytochrome p450 [Diplodia corticola]
c86109.graph_c0	1.31E-15	-1.3463	down	histone H3, partial [Colletotrichum tofieldiae]
c80031.graph_c0	0.00011	-1.0288	down	probable glutamate decarboxylase [Fusarium prolif
c97280.graph_c0	4.37E-08	-1.4874	down	hypothetical protein DHEL01_v201055 [Diaporthe
c89986.graph_c0	2.61E-05	1.19435	up	L30e-like protein [Coccomyxa subellipsoidea C-16
c80709.graph_c0	2.99E-39	-1.4063	down	PREDICTED: lupeol synthase [Glycine max]
c89054.graph_c0	3.42E-06	-1.2734	down	heat shock protein 70 [Aureococcus anophagefferer
c54054.graph_c0	6.40E-07	1.26004	up	2,4-D inducible glutathione S-transferase [Glycine
c54982.graph_c0	1.05E-11	-1.8631	down	RNA recognition domain-containing protein [Diapo
c98237.graph_c0	3.49E-11	-1.8144	down	hypothetical protein DHEL01_v200979 [Diaporthe
c54524.graph_c0	3.17E-21	1.5263	up	hypothetical protein KK1_017515 [Cajanus cajan]
c71268.graph_c0	4.84E-24	-2.6999	down	alpha-amylase 1 [Diaporthe helianthi]
c53684.graph_c0	3.61E-15	-2.1371	down	putative rhamnogalacturonan acetylesterase protein
c85609.graph_c0	0.00018	-1.0688	down	putative aspartic endopeptidase pep2 [Diplodia seri
c95812.graph_c0	1.45E-29	-2.9682	down	clock-controlled gene-15 [Diaporthe helianthi]
c54633.graph_c1	3.66E-23	-2.654	down	heavy metal tolerance protein [Diaporthe helianthi]
c97394.graph_c0	1.61E-13	-2.0188	down	Nitrate transporter [Diplodia seriata]
c88767.graph_c1	8.75E-76	-1.601	down	PREDICTED: protein MALE DISCOVERER 2-lik
c95940.graph_c0	2.87E-09	-1.6256	down	putative oxalate protein [Neofusicoccum parvum U
c96769.graph_c0	6.25E-13	-1.9706	down	rhamnogalacturonate lyase a [Diplodia corticola]
c75270.graph_c0	5.29E-49	2.80544	up	BURP domain protein RD22 [Cajanus cajan]
c72814.graph_c0	2.04E-09	1.65564	up	related to beta-mannanase [Fusarium mangiferae]
c89248.graph_c3	7.39E-09	1.50045	up	PREDICTED: polyphenol oxidase A1, chloroplasti
c54203.graph_c0	2.55E-24	-2.7153	down	hypothetical protein DHEL01_v212164 [Diaporthe
c38490.graph_c0	9.86E-12	-1.8524	down	mfs sugar transporter [Diplodia corticola]
c80334.graph_c0	0.00019	-1.0247	down	hypothetical protein FAVG1_09175 [Fusarium ave
c81487.graph_c0	1.14E-78	-1.5877	down	unknown [Lotus japonicus]
c38190.graph_c1	1.05E-12	-1.9515	down	phosphorus acquisition-controlling protein [Diaport
c38524.graph_c0	1.02E-11	-1.8642	down	putative methyltransferase [Aspergillus novofumig
c70096.graph_c0	5.06E-35	2.69329	up	hypothetical protein PHAVU_011G044500g [Phas
c54577.graph_c0	#######	1.68156	up	unknown, partial [Glycine max]
c79333.graph_c0	3.00E-05	-1.1131	down	cystatin-like [Cajanus cajan]
c76820.graph_c0	6.83E-07	-1.3868	down	hypothetical protein DHEL01_v203734 [Diaporthe
c81394.graph_c0	8.01E-06	1.06858	up	PREDICTED: protein DETOXIFICATION 55-like

c76392.graph_c0	2.35E-05	-1.0249	down	PREDICTED: uncharacterized protein LOC109353
c83291.graph_c0	5.71E-11	1.09214	up	AAA-ATPase At2g18193-like [Cajanus cajan]
c95532.graph_c0	#######	-5.0465	down	putative multicopper oxidase [Diaporthe ampelina]
c39619.graph_c1	1.40E-13	-2.0246	down	putative aspartate aminotransferase protein [Phaeoa
c83039.graph_c0	3.66E-14	-2.0631	down	l-xylulose reductase [Diplodia corticola]
c86549.graph_c0	1.77E-05	1.20988	up	hypothetical protein GLYMA_18G066200 [Glycine
c91909.graph_c0	3.36E-85	-1.1001	down	PREDICTED: glycerophosphodiester phosphodiest
c89243.graph_c0	9.53E-07	1.35776	up	ribosomal protein S3 (mitochondrion) [Millettia pir
c39071.graph_c0	1.53E-07	1.27503	up	hypothetical protein PHAVU_009G131000g [Phase
c95519.graph_c0	7.01E-44	-3.0102	down	Sugar/inositol transporter [Macrophomina phaseoli
c82953.graph_c1	9.47E-10	-1.6927	down	transmembrane alpha-helix domain-containing prot
c77520.graph_c0	2.42E-38	1.19961	up	hypothetical protein PHAVU_009G162900g [Phase
c78489.graph_c0	3.28E-33	1.29988	up	PREDICTED: kunitz-type trypsin inhibitor KTI1-li
c68962.graph_c0	2.12E-13	-1.8438	down	sugar transporter [Diplodia corticola]
c76639.graph_c0	2.04E-24	1.11878	up	uncharacterized protein LOC109803911 [Cajanus c
c88639.graph_c0	2.97E-06	-1.2196	down	hypothetical protein FPSE_00124 [Fusarium pseud
c75973.graph_c0	3.87E-62	-2.3845	down	PREDICTED: mannan endo-1,4-beta-mannosidase
c96102.graph_c0	2.99E-11	-1.8201	down	putative isochorismatase hydrolase protein [Neofus
c82042.graph_c0	4.21E-85	-4.5211	down	putative zinc finger containing protein [Diplodia se
c90533.graph_c1	2.87E-05	-1.1596	down	PREDICTED: abscisic acid receptor PYL12-like [C
c86680.graph_c1	5.98E-17	1.05568	up	PREDICTED: boron transporter 1 [Glycine max]
c88218.graph_c0	3.51E-23	1.19473	up	calmodulin-binding receptor-like cytoplasmic kinas
c96030.graph_c0	2.98E-28	-2.9062	down	hypothetical protein DHEL01_v209067 [Diaporthe
c57665.graph_c0	2.64E-37	2.00716	up	putative mannitol dehydrogenase, partial [Cajanus c
c61923.graph_c0	2.38E-08	-1.5189	down	cAMP-independent regulatory protein pac2 [Diapor
c90915.graph_c1	1.58E-16	2.2447	up	hypothetical protein RSOLAG22IIIB_04364 [Rhizo
c95987.graph_c0	5.61E-42	-3.4541	down	nitrite reductase [Diaporthe helianthi]
c39766.graph_c0	1.10E-09	-1.6738	down	hypothetical protein UCDDS831_g08547 [Diplodia
c82226.graph_c1	5.56E-22	-2.5873	down	hypothetical protein DHEL01_v209978 [Diaporthe
c64960.graph_c0	5.86E-10	-1.7049	down	aggrecan core protein [Diplodia corticola]
c88648.graph_c0	6.33E-14	-1.8472	down	putative cmgc mapk protein kinase [Diaporthe amp
c54965.graph_c0	1.09E-35	1.44418	up	PREDICTED: uncharacterized protein LOC100804
c92014.graph_c1	1.70E-20	-1.698	down	pentose phosphate metabolism-2 [Diaporthe heliant
c90468.graph_c1	2.39E-06	-1.1776	down	probable PYC2 Pyruvate carboxylase 2 [Fusarium c
c87690.graph_c0	4.70E-09	-1.5878	down	related to cutinase negative acting protein [Fusariu
c55439.graph_c0	2.20E-16	-2.2369	down	hypothetical protein PFICI_00156 [Pestalotiopsis fi
c96449.graph_c0	6.37E-14	-2.0521	down	hypothetical protein DHEL01_v209474 [Diaporthe
c87163.graph_c0	6.31E-07	1.37084	up	60S ribosomal protein L10 [Sorghum bicolor]
c39866.graph_c1	4.41E-10	-1.7066	down	hypothetical protein DHEL01_v209106 [Diaporthe
c69739.graph_c0	5.60E-37	-3.2711	down	putative tannase subunit [Diaporthe ampelina]
c95400.graph_c0	3.15E-07	1.43024	up	hypothetical protein PHAVU_001G006700g [Phase
c78587.graph_c2	2.38E-16	1.40566	up	PREDICTED: long-chain-alcohol oxidase FAO4A-
c68455.graph_c0	1.42E-12	-1.9403	down	hypothetical protein UCDDS831_g00989 [Diplodia
c37805.graph_c0	1.40E-09	1.10911	up	ABC transporter B family member 15 [Glycine soja
c89959.graph_c0	9.77E-14	-1.1053	down	PREDICTED: probable carboxylesterase 18 [Glycin
c83363.graph_c0	#######	1.32893	up	hypothetical protein PHAVU_001G221200g [Phase

c38011.graph_c0	6.24E-65	-4.1261	down	atpase aaa+ type core protein [Diplodia corticola]
c95727.graph_c0	1.08E-14	-2.1126	down	hypothetical protein DHEL01_v205104 [Diaporthe
c58433.graph_c0	6.00E-08	-1.4591	down	unknown [Lotus japonicus]
c78889.graph_c0	1.81E-44	1.42826	up	flavone synthase II [Glycine max]
c38051.graph_c0	5.05E-07	-1.4048	down	hypothetical protein BK809_0005152 [Diplodia ser
c37306.graph_c0	4.93E-12	-1.8923	down	malate dehydrogenase [Diaporthe helianthi]
c95492.graph_c0	1.16E-11	-1.5617	down	PREDICTED: alpha carbonic anhydrase 7-like [Gly
c85980.graph_c0	3.44E-34	1.44149	up	hypothetical protein GOBAR_AA16233 [Gossypiu
c57632.graph_c2	2.17E-13	-2.0091	down	calcium P-type ATPase-2 [Diaporthe helianthi]
c37951.graph_c0	2.17E-23	1.63873	up	hypothetical protein TSUD_286930 [Trifolium sub
c91297.graph_c0	#######	1.16572	up	sugar transporter ERD6-like 16 [Cajanus cajan]
c68809.graph_c0	7.22E-18	-2.3385	down	hypothetical protein MYCTH_2061639 [Thermoth
c81416.graph_c0	3.24E-30	2.47926	up	PREDICTED: serine carboxypeptidase-like 17 [Gly
c82180.graph_c0	8.12E-47	2.46871	up	oleosin [Medicago truncatula]
c95713.graph_c0	3.90E-37	-3.2773	down	multicopper oxidase [Diplodia corticola]
c86808.graph_c0	1.35E-22	-1.5577	down	aldehyde dehydrogenase [Fusarium verticillioides 7
c89629.graph_c0	1.23E-23	-1.0753	down	xyloglucan endotransglucosylase/hydrolase family
c61452.graph_c1	2.38E-26	-2.8183	down	hypothetical protein DHEL01_v208629 [Diaporthe
c76250.graph_c0	2.82E-15	1.81289	up	2-hydroxyisoflavanone dehydratase [Medicago trun
c71965.graph_c0	4.23E-10	-1.7257	down	Proteinase inhibitor I13 potato inhibitor I [Macroph
c40188.graph_c0	3.33E-12	-1.9081	down	hypothetical protein VFPPC_08845 [Pochonia chla
c58306.graph_c0	5.54E-17	-2.2788	down	NADPH dehydrogenase [Diaporthe helianthi]
c82538.graph_c0	9.47E-16	-2.1914	down	Hexose transporter HXT13 [Valsa mali]
c61524.graph_c1	6.35E-09	-1.5841	down	hypothetical protein BKCO1_8100018 [Diplodia co
c95477.graph_c0	0.00043	1.01455	up	uncharacterized protein LOC106766732 [Vigna rad
c93434.graph_c3	1.26E-08	1.08072	up	Pleiotropic drug resistance protein 1 [Glycine soja]
c79734.graph_c0	8.60E-09	-1.561	down	PREDICTED: miraculin-like [Glycine max]
c75533.graph_c0	7.00E-07	1.39156	up	PREDICTED: LOB domain-containing protein 12 [
c55674.graph_c0	1.43E-13	1.1271	up	serine carboxypeptidase-like 34 [Cajanus cajan]
c86642.graph_c0	7.98E-39	-3.1415	down	beta-1,3-glucanosyltransferase [Diaporthe helianthi
c76132.graph_c0	2.18E-18	1.02453	up	PREDICTED: UDP-glycosyltransferase 73C3-like
c73344.graph_c0	9.35E-05	1.07062	up	late embryogenesis abundant (LEA)-like protein [M
c39775.graph_c0	1.67E-09	-1.647	down	hypothetical protein DHEL01_v207076 [Diaporthe
c81149.graph_c0	9.07E-63	1.65978	up	probable pectinesterase/pectinesterase inhibitor 7 [
c71205.graph_c0	5.53E-07	-1.3853	down	putative Ddr48p [Diplodia seriata]
c79217.graph_c0	#######	-1.303	down	PREDICTED: probable galactinol--sucrose galacto
c76688.graph_c0	9.89E-17	-1.9778	down	hypothetical protein PHAVU_006G075700g [Phas
c39701.graph_c1	1.48E-13	-2.0213	down	putative fatty acid oxygenase [Diplodia seriata]
c81728.graph_c0	2.41E-15	1.79008	up	related to HXT3-Low-affinity hexose facilitator [Fu
c86908.graph_c0	3.01E-29	-2.7839	down	hypothetical protein glysoja_048907, partial [Glyci
c79298.graph_c0	3.68E-49	-3.6306	down	hypothetical protein DHEL01_v206493 [Diaporthe
c70321.graph_c0	5.13E-32	2.03916	up	gibberellin-regulated family protein [Medicago trun
c95057.graph_c0	3.61E-05	1.01896	up	uncharacterized protein LOC109800562 [Cajanus c
c93139.graph_c1	4.84E-06	1.24546	up	hypothetical protein PHAVU_002G075900g [Phas
c37763.graph_c0	6.53E-25	-2.7471	down	hypothetical protein MYCTH_2110682 [Thermoth
c86406.graph_c1	4.27E-07	-1.024	down	kinesin-like protein KIN-12A [Cajanus cajan]

c42824.graph_c0	2.68E-22	2.05606	up	cytokinin dehydrogenase 3-like [Cajanus cajan]
c90242.graph_c0	8.03E-27	2.26853	up	Enzymatic polyprotein [Cajanus cajan]
c91619.graph_c1	3.82E-10	1.09366	up	ABC transporter G family member 22 [Vigna radiat
c68022.graph_c0	4.56E-10	-1.7048	down	hypothetical protein DHEL01_v203398 [Diaporthe
c93897.graph_c0	0.0003	1.03175	up	ABC transporter G family member 36-like protein,
c94232.graph_c1	9.27E-95	-1.4449	down	PREDICTED: mannan endo-1,4-beta-mannosidase
c81508.graph_c0	1.64E-11	1.04488	up	protein LOW PSII ACCUMULATION 1, chloropla
c77215.graph_c1	8.66E-10	-1.6758	down	cAMP-dependent protein kinase regulatory subunit
c88024.graph_c0	7.22E-26	1.44651	up	cytochrome P450 family protein [Medicago truncat
c78012.graph_c0	1.96E-14	2.08751	up	MSF superfamily transporter [Fusarium kyushuense
c75643.graph_c0	9.37E-08	1.16322	up	Peroxidase 25 [Cajanus cajan]
c40212.graph_c0	8.41E-11	-1.7785	down	glucose-methanol-choline oxidoreductase [Arthrode
c95766.graph_c0	9.59E-15	-2.1165	down	glycoside hydrolase family 61 protein [Diplodia cor
c90814.graph_c0	2.01E-20	1.67071	up	glutamate decarboxylase-like [Vigna radiata var. ra
c38465.graph_c0	4.75E-27	-2.8501	down	putative glycoside hydrolase family 16 protein [Dia
c78289.graph_c0	1.22E-20	-2.5105	down	Lactose permease [Valsa mali]
c68646.graph_c1	9.09E-05	1.11399	up	probable glycosyltransferase At3g07620 [Vigna rad
c85518.graph_c0	2.55E-05	1.196	up	unnamed protein product [Fusarium sp. FIESC_5 C
c84426.graph_c0	7.38E-08	1.12368	up	PREDICTED: serine--glyoxylate aminotransferase
c93837.graph_c1	0	-2.4058	down	probable galactinol--sucrose galactosyltransferase 2
c57561.graph_c0	5.95E-09	1.08903	up	Kynurenine formamidase [Cajanus cajan]
c79669.graph_c0	1.96E-21	1.29007	up	F-box protein At2g27310-like [Cajanus cajan]
c67703.graph_c0	2.10E-95	-4.577	down	lactose permease protein [Diplodia corticola]
c37850.graph_c0	#######	1.48215	up	glucan endo-1,3-beta-glucosidase, basic isoform [C
c82809.graph_c0	2.04E-91	-1.0775	down	phosphoribosylamine--glycine ligase [Glycine max]
c86814.graph_c0	7.43E-16	1.168	up	PREDICTED: membrane protein of ER body-like p
c94485.graph_c0	9.17E-30	2.03214	up	hypothetical protein GLYMA_15G254300 [Glycine
c80361.graph_c0	6.91E-19	-1.9325	down	putative glutamine synthetase protein [Neofusicocc
c74990.graph_c0	2.86E-19	-2.4276	down	MFS general substrate transporter [Aspergillus taicl
c38822.graph_c0	4.55E-09	-1.6004	down	transcriptional activator hac1 [Diaporthe helianthi]
c88270.graph_c0	3.31E-45	-3.565	down	putative glycosyl hydrolase family 7 protein [Neofu
c94768.graph_c1	1.27E-09	-1.0719	down	unnamed protein product [Fusarium venenatum]
c82859.graph_c0	4.31E-33	-3.1031	down	putative aspergillopepsin-2 protein [Neofusicoccum
c37920.graph_c0	1.88E-75	4.29318	up	early nodulin-75-like [Cajanus cajan]
c82748.graph_c0	3.22E-12	1.35164	up	uncharacterized protein LOC109811309 [Cajanus c
c93468.graph_c0	1.72E-05	-1.1881	down	related to synaptic vesicle transporter SV2 (major fa
c82622.graph_c0	3.54E-08	-1.1543	down	PREDICTED: uncharacterized protein LOC100810
c97161.graph_c0	4.01E-10	-1.7112	down	putative solute carrier family 35 member e3 [Diapo
c83013.graph_c0	1.42E-06	1.04929	up	PREDICTED: probable protein phosphatase 2C 72
c55680.graph_c0	3.51E-13	-1.9915	down	putative glucose-6-phosphate 1-dehydrogenase [Dia
c76161.graph_c0	7.96E-09	1.10176	up	PREDICTED: two-component response regulator A
c74912.graph_c0	1.34E-27	2.4136	up	14 kDa proline-rich protein DC2.15 [Arachis duran
c78582.graph_c0	#######	-4.2323	down	putative btb poz-like protein [Diplodia seriata]
c96049.graph_c0	9.66E-09	-1.5923	down	c6 zinc finger domain containing protein [Diplodia
c68911.graph_c0	1.18E-12	1.16898	up	germin-like protein subfamily 3 member 1 [Cajanus
c93811.graph_c0	6.03E-25	-1.2769	down	hypothetical protein GLYMA_20G047600 [Glycine

c85642.graph_c0	5.50E-06	-1.2621	down	hypothetical protein FPSE_03661 [Fusarium pseud
c70096.graph_c1	1.34E-14	2.02866	up	pterocarpan reductase [Lotus japonicus]
c86285.graph_c1	2.61E-15	-2.1522	down	sugar transporter [Ophiostoma piceae UAMH 1134
c95402.graph_c0	1.35E-22	-2.6211	down	hypothetical protein PFICI_00156 [Pestalotiopsis fi
c55966.graph_c0	1.45E-24	-2.7265	down	MFS quinate transporter [Diaporthe helianthi]
c96381.graph_c0	1.44E-11	-1.8501	down	NHL repeat-containing protein [Aspergillus steynii
c95650.graph_c0	5.32E-65	1.61011	up	--
c69825.graph_c0	4.76E-23	-2.6266	down	ammonium transporter [Diplodia corticola]
c82640.graph_c1	2.37E-13	-1.8767	down	eukaryotic translation initiation factor eIF-1 [Fusari
c78159.graph_c0	4.55E-33	-3.1163	down	high-affinity methionine permease [Colletotrichum
c96411.graph_c0	2.30E-14	-2.0869	down	putative ral amino-acid permease gap1 [Diaporthe a
c55555.graph_c0	7.90E-64	1.84176	up	uncharacterized oxidoreductase At4g09670 [Arachi
c95623.graph_c0	3.32E-26	-2.7494	down	putative pectinesterase A [Diplodia seriata]
c38126.graph_c0	3.77E-18	-2.3561	down	hypothetical protein DHEL01_v208702 [Diaporthe
c55806.graph_c0	2.26E-08	-1.5545	down	putative calcium calmodulin-dependent protein kin
c37888.graph_c0	1.99E-29	1.09024	up	--
c81458.graph_c0	1.09E-19	-2.3696	down	ubiquitin carboxyl-terminal hydrolase 2 [Diplodia c
c86609.graph_c1	0.00014	1.05528	up	PREDICTED: putative receptor protein kinase ZmI
c65214.graph_c0	2.44E-11	-1.8291	down	hypothetical protein DHEL01_v201142 [Diaporthe
c38729.graph_c0	4.31E-16	-2.2162	down	hypothetical protein DHEL01_v200797 [Diaporthe
c53750.graph_c0	1.60E-09	-1.6493	down	putative short chain dehydrogenase reductase [Diap
c55893.graph_c0	3.27E-21	-2.544	down	hypothetical protein VP1G_00949 [Valsa mali var.
c80931.graph_c0	2.75E-15	1.21158	up	hypothetical protein GLYMA_13G201100 [Glycine
c82654.graph_c0	#######	1.5076	up	embryonic abundant-like protein [Medicago truncat
c84700.graph_c1	1.74E-07	-1.3982	down	cyclosporin-resistant-1 [Diaporthe helianthi]
c37924.graph_c0	3.82E-72	1.74412	up	E3 ubiquitin-protein ligase ATL23-like [Cajanus ca
c84485.graph_c0	1.97E-23	-2.669	down	hypothetical protein DHEL01_v211129 [Diaporthe
c74520.graph_c0	4.94E-06	1.21219	up	PREDICTED: uncharacterized protein LOC100776
c86701.graph_c0	4.07E-08	1.06066	up	uncharacterized protein LOC109805728 [Cajanus c
c84481.graph_c0	1.46E-22	-2.5191	down	pectin lyase [Fusarium langsethiae]
c95684.graph_c0	1.72E-14	-2.0967	down	kinase-like protein [Stagonospora sp. SRC1lsM3a]
c83532.graph_c0	7.87E-39	1.28736	up	GDSL esterase/lipase At5g55050-like [Cajanus caj
c82135.graph_c0	1.16E-20	-2.4131	down	hypothetical protein UCDDS831_g01262 [Diplodia
c95201.graph_c0	2.18E-81	1.15174	up	retrotransposon protein, putative, Ty1-copia sub-cla
c70541.graph_c0	0.00014	-1.0156	down	unknown [Lotus japonicus]
c85037.graph_c0	7.99E-29	1.53092	up	protein E6-like [Cajanus cajan]
c94180.graph_c0	5.90E-11	1.67189	up	predicted protein [Hordeum vulgare subsp. vulgare]
c68228.graph_c0	2.11E-08	-1.5581	down	putative serine threonine protein kinase [Diplodia s
c37889.graph_c0	5.27E-08	-1.4511	down	hypothetical protein PHAVU_003G218900g [Phase
c59651.graph_c0	1.90E-22	-2.6136	down	nucleoside-triphosphatase, RNA helicase [Pseudolc
c94784.graph_c1	3.41E-66	1.16279	up	putative LOV domain-containing protein [Codorioc
c76818.graph_c0	0.00013	1.08752	up	PREDICTED: tropinone reductase-like isoform X2
c83298.graph_c0	1.60E-10	1.75106	up	acetyl-coenzyme A synthetase [Rhizoctonia solani
c54332.graph_c0	1.06E-11	1.54378	up	PREDICTED: 21 kDa protein-like [Glycine max]
c74774.graph_c0	1.05E-62	3.34417	up	PREDICTED: uncharacterized protein LOC109351
c87345.graph_c0	1.97E-42	1.80708	up	conserved hypothetical protein [Ricinus communis]

c89007.graph_c0	3.43E-05	-1.0109	down	probable ribosomal protein L9.e.c14 [Fusarium fuji
c95345.graph_c0	8.91E-13	-1.9566	down	hypothetical protein P174DRAFT_442171 [Asperg
c95827.graph_c0	2.52E-84	-4.586	down	hypothetical protein MPH_13779 [Macrophomina p
c72296.graph_c0	1.01E-07	1.28592	up	PREDICTED: uncharacterized protein LOC100783
c38636.graph_c1	3.52E-10	-1.7175	down	putative cyclin-like protein [Diaporthe ampelina]
c85193.graph_c0	1.10E-12	1.68436	up	PREDICTED: G-type lectin S-receptor-like serine/t
c87844.graph_c1	4.76E-72	1.19854	up	Putative xyloglucan endotransglucosylase/hydrolase
c77397.graph_c0	3.10E-09	1.23533	up	hypothetical protein GLYMA_20G047000 [Glycine
c92156.graph_c1	2.58E-06	1.29745	up	Elongation factor 3 OS=Schizosaccharomyces pom
c92523.graph_c0	2.05E-77	1.28392	up	hypothetical protein PHAVU_008G090600g [Phase
c76130.graph_c0	3.01E-20	2.04498	up	hypothetical protein KK1_017617 [Cajanus cajan]
c79536.graph_c5	1.62E-22	2.55958	up	PREDICTED: beta-amyrin 11-oxidase-like [Cicer a
c85214.graph_c0	0.00016	1.06434	up	60S ribosomal protein L27a-3 [Monoraphidium neg
c95843.graph_c0	6.10E-10	-1.6776	down	putative mfs sugar transporter [Diplodia seriata]
c95261.graph_c0	1.98E-92	2.24901	up	hypothetical protein glysoja_025159 [Glycine soja]
c80702.graph_c0	4.65E-06	-1.2836	down	unnamed protein product [Fusarium venenatum]
c67762.graph_c0	5.32E-09	-1.5924	down	general substrate transporter [Diplodia corticola]
c55260.graph_c0	3.01E-15	1.65173	up	Peroxidase 53, partial [Cajanus cajan]
c80948.graph_c0	2.77E-08	1.54477	up	TMV resistance protein N-like [Cajanus cajan]
c37871.graph_c0	2.81E-09	1.62477	up	hypothetical protein TanjilG_12047 [Lupinus angus
c70155.graph_c0	1.40E-15	-2.1656	down	hypothetical protein UCDDS831_g05530 [Diplodia
c75903.graph_c0	3.52E-10	-1.7025	down	hypothetical protein FPOA_02826 [Fusarium poae]
c95571.graph_c0	7.62E-30	-2.9789	down	putative acid proteinase [Diaporthe ampelina]
c88189.graph_c0	#######	1.23104	up	PREDICTED: oligopeptide transporter 7-like [Lupi
c82486.graph_c0	1.96E-32	2.0244	up	hypothetical protein GLYMA_20G213700 [Glycine
c88148.graph_c0	3.68E-05	-1.1712	down	PREDICTED: tetrahydrocannabinolic acid synthase
c90327.graph_c0	1.76E-19	1.20301	up	protochlorophyllide-dependent translocon compone
c69203.graph_c0	2.53E-16	-1.256	down	glutathione S-transferase U17-like [Cajanus cajan]
c83005.graph_c0	2.51E-08	1.516	up	hypothetical protein RSOLAG22IIIB_07618 [Rhizo
c81192.graph_c0	2.57E-39	1.16685	up	photosystem II 22 kDa protein, chloroplastic [Arach
c89941.graph_c0	1.77E-17	1.07802	up	hypothetical protein GLYMA_09G279000 [Glycine
c86331.graph_c0	5.02E-10	-1.2819	down	polygalacturonase-like [Cajanus cajan]
c81019.graph_c0	9.38E-13	1.38001	up	secoisolariciresinol dehydrogenase-like, partial [Ca
c82102.graph_c0	1.20E-20	2.27715	up	beta-amyrin 11-oxidase [Glycyrrhiza uralensis]
c37692.graph_c0	5.17E-23	-2.6446	down	NAD-dependent epimerase/dehydratase, putative [T
c81702.graph_c1	2.98E-13	-1.4966	down	WAT1-related protein At1g09380-like [Cajanus caj
c58153.graph_c1	3.85E-08	-1.5297	down	h+ nucleoside cotransporter [Diplodia corticola]
c96316.graph_c0	3.07E-12	-1.9113	down	putative endoglucanase d precursor [Diplodia seriat
c81660.graph_c0	3.62E-08	1.10249	up	Retrovirus-related Pol polyprotein from transposon
c77566.graph_c0	4.58E-40	-3.2418	down	c2h2 transcription factor [Diplodia corticola]
c87025.graph_c0	6.23E-05	-1.1367	down	unnamed protein product [Fusarium venenatum]
c95105.graph_c0	1.67E-10	1.02383	up	PREDICTED: receptor-like protein 12 [Glycine ma
c70289.graph_c0	5.88E-16	-2.1562	down	hypothetical protein BK809_0007542 [Diplodia ser
c70134.graph_c0	6.20E-10	1.30468	up	inorganic pyrophosphatase 1-like [Cajanus cajan]
c95653.graph_c0	2.16E-30	-3.0032	down	hypothetical protein DHEL01_v207178 [Diaporthe
c82346.graph_c0	1.12E-26	2.13603	up	germin-like protein subfamily 1 member 17 [Cajanu

c58153.graph_c0	5.03E-11	-1.8101	down	putative h+ nucleoside cotransporter [Diplodia seria
c82628.graph_c0	6.70E-82	1.58003	up	PREDICTED: protein yippee-like At4g27740 [Lup:
c97279.graph_c0	1.86E-06	-1.3225	down	fungal specific transcription factor domain-containi
c85468.graph_c0	#######	-1.5135	down	PREDICTED: universal stress protein A-like protei
c79907.graph_c1	4.87E-33	1.59335	up	L-ascorbate oxidase homolog [Cajanus cajan]
c91644.graph_c0	0.00032	1.03272	up	60S ribosomal protein L5 [Achlya hypogyna]
c78868.graph_c0	1.03E-13	1.43281	up	cytochrome P450 monooxygenase [Glycyrrhiza ura
c89180.graph_c0	2.74E-57	1.162	up	hypothetical protein TSUD_19710 [Trifolium subte
c73296.graph_c0	8.40E-10	-1.1339	down	putative lipid-transfer protein DIR1 [Cajanus cajan]
c77233.graph_c0	#######	2.12943	up	double-headed trypsin inhibitor [Phaseolus lunatus]
c74422.graph_c0	8.29E-82	-4.5078	down	polyketide synthase [Diplodia corticola]
c78416.graph_c0	7.14E-09	1.59882	up	related to tyrosinase precursor (monophenol monoo
c76694.graph_c2	6.84E-09	-1.2829	down	PREDICTED: putative nuclease HARBI1 [Vigna aı
c55401.graph_c0	2.84E-09	1.15096	up	uclacyanin 1-like [Cajanus cajan]
c91803.graph_c2	7.83E-52	1.67009	up	Secologanin synthase [Glycine soja]
c95214.graph_c0	7.31E-17	1.48212	up	PREDICTED: disease resistance protein At4g2719(
c94240.graph_c0	5.91E-08	-1.192	down	probable aconitase [Fusarium proliferatum]
c95819.graph_c0	1.73E-34	-3.1749	down	sulfate permease [Diaporthe helianthi]
c85449.graph_c0	2.78E-10	1.73816	up	60S acidic ribosomal protein P1 [Eutrema salsugine
c78666.graph_c0	4.04E-26	1.06519	up	hypothetical protein PHAVU_011G068300g [Phase
c96306.graph_c0	2.65E-07	-1.4369	down	glycosyltransferase family 2 protein [Diplodia corti
c74367.graph_c1	1.99E-06	-1.3375	down	putative pre-mrna splicing factor protein [Neofusic
c74291.graph_c0	2.95E-45	-3.5619	down	2-keto-4-pentenoate hydratase [Diaporthe helianthi]
c80241.graph_c0	0.0002	-1.0134	down	PREDICTED: amino acid permease 8-like isoform
c74117.graph_c0	4.17E-13	1.64535	up	uncharacterized protein LOC109801796 [Cajanus c
c58116.graph_c0	1.10E-14	-2.1118	down	Phosphoesterase [Macrophomina phaseolina MS6]
c87454.graph_c0	1.09E-97	1.67295	up	PREDICTED: uncharacterized protein LOC109134
c76051.graph_c0	2.92E-30	-2.9986	down	H+/nucleoside cotransporter [Diaporthe helianthi]
c88937.graph_c1	3.82E-08	-1.5198	down	hypothetical protein FPOA_10356 [Fusarium poae]
c88362.graph_c0	1.09E-08	-1.4058	down	unnamed protein product [Fusarium sp. FIESC_5 C
c84114.graph_c0	6.33E-19	-1.6281	down	PREDICTED: 18.2 kDa class I heat shock protein-l
c96073.graph_c0	2.96E-14	-2.0783	down	putative cdc50 family protein [Diaporthe ampelina]
c83720.graph_c0	#######	1.26839	up	peroxidase P7-like [Cajanus cajan]
c90919.graph_c2	1.42E-26	1.44255	up	Transcription factor UNE10 [Glycine soja]
c54997.graph_c0	2.59E-15	1.20149	up	putative lipid-transfer protein DIR1 [Vigna radiata
c82570.graph_c2	4.92E-15	-2.1374	down	elongation factor 3 [Diplodia corticola]
c95167.graph_c0	8.23E-23	1.85536	up	Retrovirus-related Pol polyprotein from transposon
c76925.graph_c0	4.01E-34	-3.1596	down	hypothetical protein UCDDA912_g05228 [Diaport
c82808.graph_c0	#######	-1.2134	down	uncharacterized protein LOC109817948 [Cajanus c
c81124.graph_c0	7.45E-05	-1.0936	down	protein RTF1 homolog [Vigna radiata var. radiata]
c54296.graph_c0	3.28E-08	-1.5383	down	Histone H1/H5 [Macrophomina phaseolina MS6]
c80175.graph_c0	5.42E-17	1.31036	up	PREDICTED: photosynthetic NDH subunit of subc
c93872.graph_c0	3.42E-11	1.34788	up	Serine carboxypeptidase-like 49 [Cajanus cajan]
c88899.graph_c0	2.02E-26	1.21259	up	PREDICTED: potassium transporter 5 [Glycine ma
c96125.graph_c0	3.95E-14	-2.0681	down	hypothetical protein ALT_4866 [Aspergillus lentulı
c95540.graph_c0	9.94E-19	1.47953	up	PREDICTED: 1-aminocyclopropane-1-carboxylate

c73301.graph_c0	#######	-1.3963	down	desiccation protective protein LEA5 [Glycine max]
c56179.graph_c1	7.89E-13	-1.9631	down	hypothetical protein BK809_0007407 [Diplodia ser
c86759.graph_c0	1.73E-09	-1.1725	down	hypothetical protein CDD82_6266 [Ophiocordycep
c82444.graph_c0	6.87E-16	-2.2019	down	hypothetical protein DHEL01_v208490 [Diaporthe
c92020.graph_c6	1.98E-09	-1.6391	down	putative flavoprotein involved in k+ transport [Diap
c73020.graph_c0	1.57E-77	1.10595	up	PR-5b protein precursor [Glycine max]
c92052.graph_c1	1.00E-18	1.39906	up	ubiquinol oxidase 1 mitochondrial-like [Trifolium p
c90461.graph_c0	6.26E-46	1.32073	up	basic 7S globulin-like [Arachis duranensis]
c87422.graph_c0	2.03E-17	-1.173	down	uncharacterized protein LOC100808567 [Glycine m
c80072.graph_c0	1.80E-05	1.11523	up	Zinc finger protein CONSTANS-LIKE 6 [Cajanus c
c89765.graph_c0	2.25E-07	-1.0224	down	glyceraldehyde-3-phosphate dehydrogenase [Fusari
c64715.graph_c1	4.87E-15	-2.1388	down	hypothetical protein DHEL01_v202533 [Diaporthe
c36772.graph_c0	4.37E-12	-1.8972	down	udp-glucose 4-epimerase [Diaporthe helianthi]
c84724.graph_c0	5.62E-08	1.15301	up	laccase-3-like [Cajanus cajan]
c67694.graph_c0	4.04E-69	-4.2454	down	major facilitator superfamily domain-containing pro
c67561.graph_c0	1.08E-31	-1.7694	down	hypothetical protein glysoja_008433 [Glycine soja]
c37855.graph_c0	2.72E-37	-1.7804	down	PREDICTED: bidirectional sugar transporter N3-lil
c67487.graph_c0	7.57E-13	-1.9579	down	hypothetical protein MPH_10939 [Macrophomina p
c84662.graph_c0	2.33E-13	-2.0061	down	putative endoglucanase-4 precursor [Diplodia seriat
c54950.graph_c0	3.19E-80	1.2127	up	Snakin-2 [Glycine soja]
c82840.graph_c0	2.39E-06	-1.3168	down	guanyl-specific ribonuclease f1 [Diplodia corticola]
c55590.graph_c0	6.99E-17	-2.2716	down	putative MFS-type transporter PB1E7.08c [Valsa m
c91797.graph_c1	9.84E-18	-1.6555	down	probable nitrate transport protein crnA [Fusarium m
c80075.graph_c0	1.08E-13	-2.0336	down	phthalate transporter [Diaporthe helianthi]
c89167.graph_c1	7.75E-16	-2.0228	down	phosphoenolpyruvate carboxykinase (ATP) [Diapor
c76323.graph_c0	5.07E-13	1.12164	up	PREDICTED: 1-aminocyclopropane-1-carboxylate
c84113.graph_c0	7.26E-11	1.24626	up	PREDICTED: bidirectional sugar transporter SWEI
c79771.graph_c0	1.31E-05	-1.1267	down	PREDICTED: solute carrier family 25 member 44-l
c80224.graph_c0	3.32E-11	1.04818	up	probable 2-oxoglutarate-dependent dioxygenase AC
c83505.graph_c0	2.12E-16	-1.195	down	PREDICTED: transcription factor bHLH61-like [G
c88850.graph_c0	1.33E-24	2.73097	up	FliC domain-containing protein [Rhizoctonia solani
c57159.graph_c0	1.10E-11	-1.8601	down	hypothetical protein DHEL01_v201049 [Diaporthe
c55844.graph_c0	3.70E-33	-3.1205	down	hypothetical protein DHEL01_v207595 [Diaporthe
c80213.graph_c0	3.31E-42	-1.2905	down	putative glutathione S-transferase parA, partial [Caj
c37574.graph_c0	0.00032	-1.0362	down	hypothetical protein UCDDS831_g03274 [Diplodia
c93162.graph_c1	1.29E-07	1.4714	up	PREDICTED: pleiotropic drug resistance protein 3
c53961.graph_c0	7.57E-28	2.8878	up	hypothetical protein BN14_10313 [Rhizoctonia sola
c80854.graph_c0	2.85E-18	1.63849	up	leucine-rich repeat extensin-like protein 6 [Cajanus
c37976.graph_c0	4.67E-38	-3.2355	down	hypothetical protein BK809_0007139 [Diplodia ser
c84032.graph_c0	6.53E-84	1.49739	up	RabGAP/TBC domain protein [Medicago truncatul
c89413.graph_c0	4.16E-29	1.1336	up	G-type lectin S-receptor-like serine/threonine-prote
c34524.graph_c0	4.95E-09	-1.5962	down	carboxypeptidase Y precursor [Diaporthe helianthi]
c92624.graph_c1	8.86E-17	-1.7458	down	S-adenosylmethionine synthase [Fusarium oxyspor
c96109.graph_c0	1.28E-06	-1.3478	down	heat shock protein, HSP70, hsc70 [Thalassiosira ps
c75208.graph_c0	9.63E-12	-1.8667	down	hypothetical protein BKCO1_5600048 [Diplodia co
c69219.graph_c0	3.84E-49	-3.6839	down	metacaspase [Diaporthe helianthi]

c88885.graph_c0	1.41E-13	1.07719	up	PREDICTED: oxalate--CoA ligase-like [Vigna ang
c68005.graph_c0	1.46E-26	-2.8156	down	siderochrome-iron transporter [Diplodia corticola]
c37840.graph_c0	7.53E-06	1.23862	up	Zinc finger protein ZAT8 [Glycine soja]
c88342.graph_c0	2.08E-44	1.7249	up	hypothetical protein KK1_027765 [Cajanus cajan]
c80331.graph_c0	9.50E-24	-2.5702	down	putative c2h2 transcription factor [Diplodia seriata]
c81344.graph_c0	2.35E-36	1.08525	up	PREDICTED: cytochrome P450 71A26-like [Glyci
c92606.graph_c1	1.86E-18	-2.1211	down	hypothetical protein FNYG_03084 [Fusarium nyga
c86616.graph_c0	4.99E-31	-2.9927	down	mfs peptide transporter [Diplodia corticola]
c76212.graph_c0	6.54E-18	2.29713	up	hypothetical protein PHAVU_006G196900g [Phase
c38182.graph_c0	2.07E-28	-2.8488	down	Histidine phosphatase superfamily clade-2 [Macrop
c75670.graph_c0	1.07E-16	2.20552	up	related to lactose permease [Fusarium mangiferae]
c76533.graph_c1	0.00025	1.04139	up	PREDICTED: serine/threonine-protein kinase At5g
c80415.graph_c0	5.51E-73	2.13654	up	peroxidase 3-like [Cajanus cajan]
c83796.graph_c1	6.73E-11	1.29056	up	2-methylene-furan-3-one reductase-like [Cajanus ca
c82697.graph_c0	1.75E-12	1.93601	up	cytokinin dehydrogenase 2-like [Cajanus cajan]
c75135.graph_c0	1.35E-52	2.03257	up	Non-specific lipid-transfer protein-like protein [Gly
c92950.graph_c0	0	-2.0797	down	PREDICTED: pentatricopeptide repeat-containing
c82558.graph_c0	1.35E-08	-1.5435	down	putative period circadian [Diplodia seriata]
c74662.graph_c0	1.09E-17	2.30407	up	hypothetical protein GOBAR_DD29327 [Gossypiu
c83934.graph_c0	5.21E-06	1.05666	up	UDP-glycosyltransferase 23 [Pueraria montana var.
c38362.graph_c0	1.89E-12	-1.929	down	casein kinase I isoform delta [Diaporthe helianthi]
c88322.graph_c1	8.13E-21	-1.5659	down	beta-amylase 3, chloroplastic [Cajanus cajan]
c79864.graph_c0	1.77E-15	1.2231	up	PREDICTED: xyloglucan galactosyltransferase KA
c79634.graph_c0	0.00022	-1.0549	down	60s ribosomal protein l20 [Fusarium langsethiae]
c76826.graph_c0	7.24E-09	1.15671	up	transcriptional factor NAC35 [Glycine max]
c37820.graph_c0	6.35E-10	1.47419	up	PREDICTED: dehydration-responsive element-bin
c68963.graph_c0	3.89E-15	-2.0656	down	putative d-galacturonic acid reductase [Diplodia se
c73153.graph_c1	8.42E-14	-2.0424	down	hypothetical protein BKCO1_2000077 [Diplodia co
c71888.graph_c1	1.67E-12	-1.9339	down	ammonium transporter MEP1 [Diaporthe helianthi]
c72419.graph_c0	3.12E-60	-1.5359	down	hypothetical protein GLYMA_16G107200 [Glycine
c90289.graph_c0	6.71E-09	-1.1524	down	unnamed protein product [Fusarium sp. FIESC_5 C
c36933.graph_c1	4.42E-29	-2.9453	down	hypothetical protein DHEL01_v212385 [Diaporthe
c80717.graph_c1	3.43E-14	1.57334	up	calmodulin-binding protein 60 D-like isoform X1 [
c38072.graph_c0	1.77E-23	-2.6707	down	hypothetical protein DHEL01_v210153 [Diaporthe
c37884.graph_c0	1.37E-10	1.13197	up	extensin-2 isoform X2 [Vigna radiata var. radiata]
c94490.graph_c1	3.49E-08	-1.5349	down	uncharacterized protein LOC107625253 [Arachis i
c89039.graph_c0	1.53E-11	-1.3498	down	cannabidiolic acid synthase-like 2 [Cajanus cajan]
c95279.graph_c0	3.59E-52	-3.0108	down	sugar transporter [Diplodia corticola]
c55318.graph_c0	6.88E-78	3.14378	up	non-specific lipid-transfer protein 2-like [Cajanus c
c82019.graph_c0	5.00E-34	1.26256	up	Peroxidase 5 [Glycine soja]
c96135.graph_c0	7.09E-14	-2.0484	down	6-phosphogluconate dehydrogenase 2 [Diaporthe h
c74044.graph_c0	1.68E-10	1.44257	up	PREDICTED: auxin-responsive protein SAUR32-l
c70106.graph_c0	3.36E-08	-1.5372	down	Malate synthase, glyoxysomal [Diplodia seriata]
c55325.graph_c0	2.94E-13	-1.9983	down	guanine nucleotide-binding protein alpha-2 subunit
c82618.graph_c1	3.53E-17	-2.2927	down	hypothetical protein DHEL01_v204878 [Diaporthe
c95486.graph_c0	4.20E-38	-3.3146	down	hypothetical protein AOQ84DRAFT_423185 [Glo

c69635.graph_c0	1.24E-06	1.2295	up	PREDICTED: short-chain dehydrogenase reductase
c79581.graph_c0	5.23E-09	1.39894	up	PREDICTED: calcium-binding protein KIC-like [G
c78521.graph_c1	1.46E-05	1.09862	up	transcription factor MYB108-like [Cajanus cajan]
c78130.graph_c0	9.00E-20	-2.4588	down	pisatin demethylase [Diaporthe helianthi]
c56335.graph_c0	3.91E-09	-1.6068	down	nuclear localization sequence binding protein [Diap
c84147.graph_c0	7.22E-13	1.26352	up	PREDICTED: uncharacterized protein LOC101496
c72596.graph_c0	1.41E-19	1.43536	up	uncharacterized protein LOC100787073 precursor [
c57904.graph_c0	5.23E-09	1.59226	up	NAD(P)H-dependent 6'-deoxychalcone synth
c95465.graph_c0	4.17E-08	1.46356	up	PREDICTED: short-chain dehydrogenase reductase
c78720.graph_c0	2.27E-27	1.79936	up	probable mannitol dehydrogenase [Herrania umbrat
c81357.graph_c0	7.84E-47	1.88998	up	laccase-5 [Vigna radiata var. radiata]
c90832.graph_c0	#######	1.06964	up	In2-1 protein [Glycine max]
c95360.graph_c0	2.09E-28	-1.732	down	PREDICTED: late embryogenesis abundant protein
c96480.graph_c0	7.64E-15	-2.1239	down	4-hydroxyphenylpyruvate dioxygenase [Diaporthe h
c81899.graph_c0	2.38E-25	1.36983	up	sedoheptulose-1,7-bisphosphatase, chloroplastic [V
c78799.graph_c0	#######	1.7753	up	PREDICTED: 2-oxoglutarate-dependent dioxygena
c80192.graph_c1	3.48E-15	1.04866	up	unknown [Lotus japonicus]
c77790.graph_c0	1.33E-06	1.12708	up	PREDICTED: uncharacterized protein LOC108325
c84668.graph_c0	5.44E-07	-1.0403	down	unknown [Glycine max]
c57714.graph_c1	1.74E-12	-1.9328	down	putative tyrosinase [Diplodia seriata]
c37926.graph_c0	9.55E-93	1.50987	up	unknown [Glycine max]
c53988.graph_c0	3.57E-09	-1.6316	down	putative npp1 domain protein [Diplodia seriata]
c93715.graph_c0	1.81E-60	1.376	up	alpha-glucosidase-like [Cajanus cajan]
c80479.graph_c0	#######	1.02602	up	PREDICTED: endochitinase-like [Cicer arietinum]
c91951.graph_c2	1.09E-50	-1.3242	down	PREDICTED: transcription factor GAMYB-like isc
c80968.graph_c0	1.66E-07	-1.4607	down	putative erythrocyte band 7 integral membrane prot
c95506.graph_c0	8.52E-08	-1.4893	down	hypothetical protein UCDDS831_g05251 [Diplodia
c69666.graph_c0	#######	-4.9566	down	conidial yellow pigment biosynthesis polyketide syı
c55529.graph_c0	1.45E-19	-2.4483	down	putative histone h1 [Diaporthe ampelina]
c96004.graph_c0	9.72E-13	-1.9543	down	hypothetical protein DHEL01_v204164 [Diaporthe
c95706.graph_c0	#######	-5.6585	down	hypothetical protein ANO11243_042010 [fungal sp
c94161.graph_c0	3.43E-07	-1.0667	down	PREDICTED: uncharacterized protein LOC100788
c57373.graph_c0	2.57E-14	-2.083	down	vacuolar protease A [Diaporthe helianthi]
c73003.graph_c0	2.14E-08	-1.1795	down	hypothetical protein glysoja_026062 [Glycine soja]
c97826.graph_c0	1.46E-14	-2.1022	down	hypothetical protein DHEL01_v212084 [Diaporthe
c78587.graph_c1	4.88E-26	1.76919	up	PREDICTED: long-chain-alcohol oxidase FAO4A-
c53712.graph_c0	8.98E-27	1.83593	up	hypothetical protein PHAVU_010G063600g [Phase
c95616.graph_c0	7.63E-71	-4.2691	down	hemagglutinin [Diplodia corticola]
c76395.graph_c0	0.00021	-1.0628	down	unnamed protein product [Fusarium venenatum]
c93768.graph_c1	6.51E-39	1.87241	up	PREDICTED: geraniol 8-hydroxylase-like [Vigna a
c37973.graph_c0	6.87E-41	-3.3559	down	putative pectate lyase A [Diplodia seriata]
c79907.graph_c2	1.06E-14	1.76599	up	L-ascorbate oxidase, partial [Trifolium pratense]
c54683.graph_c0	1.86E-20	-2.1387	down	PREDICTED: uncharacterized protein LOC100813
c38233.graph_c1	9.45E-21	-2.5165	down	phosphate transporter [Diaporthe helianthi]
c96159.graph_c0	5.76E-09	1.60556	up	putative myo-inositol 1-phosphate synthase [Arabid
c38104.graph_c0	7.78E-29	2.90783	up	aldehyde dehydrogenase (NAD+) [Rhizoctonia sola

c88676.graph_c1	4.93E-15	-1.2327	down	hypothetical protein PHAVU_002G171800g [Phase
c78104.graph_c0	1.22E-08	-1.5831	down	putative protein yop-1 [Diaporthe ampelina]
c69843.graph_c0	#######	-2.7216	down	hypothetical protein KK1_035105 [Cajanus cajan]
c68164.graph_c1	4.21E-09	-1.6189	down	Sel1-like protein [Macrophomina phaseolina MS6]
c95472.graph_c0	8.95E-18	2.11209	up	PREDICTED: uncharacterized protein LOC100306
c57784.graph_c0	5.05E-10	-1.7011	down	putative extracellular dioxygenase [Diaporthe ampe
c88397.graph_c0	#######	1.12901	up	peroxidase 4-like protein, partial [Trifolium pratens
c82325.graph_c0	5.45E-05	1.13202	up	uncharacterized protein FFUJ_02531 [Fusarium fuj
c86609.graph_c0	0.00015	1.05953	up	PREDICTED: putative receptor protein kinase ZmI
c54427.graph_c0	1.46E-94	2.25429	up	unknown [Glycine max]
c84200.graph_c0	4.67E-13	1.31786	up	peroxidase 64 [Cajanus cajan]
c72329.graph_c0	7.88E-23	-2.6351	down	hypothetical protein UCDDS831_g07036 [Diplodia
c81279.graph_c0	2.87E-20	-2.488	down	hypothetical protein UCDDA912_g03080 [Diaportl
c91473.graph_c1	#######	1.63949	up	PREDICTED: probably inactive leucine-rich repeat
c38089.graph_c0	9.69E-13	-1.9539	down	putative mitochondrial peroxiredoxin prx1 [Diaport
c74307.graph_c0	8.67E-05	-1.1079	down	uncharacterized protein LW93_4409 [Fusarium fuji
c86530.graph_c0	1.59E-13	1.91157	up	transcription factor PRE3 [Cajanus cajan]
c83964.graph_c0	2.55E-08	1.15084	up	protein GAMETE EXPRESSED 1 [Cajanus cajan]
c55238.graph_c0	2.22E-42	-3.3405	down	putative beta- -glucan-binding protein [Neofusicocc
c97742.graph_c0	1.53E-13	-2.0214	down	GPI-anchored cell wall beta-1,3-endoglucanase Egl
c80969.graph_c0	6.50E-14	1.97169	up	hypothetical protein PHAVU_005G177000g [Phase
c78422.graph_c0	3.01E-05	1.04424	up	10-deacetylbaccatin III 10-O-acetyltransferase [Caja
c53931.graph_c0	3.80E-05	1.17065	up	UPF0481 protein At3g47200-like [Cajanus cajan]
c83882.graph_c0	1.53E-11	-1.6516	down	hypothetical protein CSIM01_04358 [Colletotrichu
c90523.graph_c0	1.34E-27	2.82576	up	hypothetical protein RSOLAG22IIIB_07711 [Rhizo
c72317.graph_c1	1.38E-14	-2.1041	down	hypothetical protein DHEL01_v205835 [Diaporthe
c75906.graph_c0	1.41E-15	-2.179	down	hypothetical protein THITE_2048634 [Thielavia te
c55604.graph_c0	1.33E-99	2.21179	up	hypothetical protein PHAVU_008G112100g [Phase
c38192.graph_c0	3.57E-16	2.22137	up	PREDICTED: cytochrome P450 78A7 [Vigna angu
c68017.graph_c0	3.83E-39	-3.3513	down	hypothetical protein DHEL01_v202969 [Diaporthe
c95543.graph_c0	2.20E-23	-2.6444	down	glycoside hydrolase family 5 protein [Diplodia corti
c84603.graph_c0	6.20E-05	-1.1153	down	36.4 kDa proline-rich protein-like [Cajanus cajan]
c95411.graph_c0	5.07E-05	1.02139	up	hypothetical protein PHAVU_009G032900g [Phase
c92163.graph_c0	1.87E-06	-1.2597	down	related to putative tartrate transporter [Fusarium ma
c39758.graph_c0	9.67E-12	-1.8661	down	sugar transporter [Diplodia corticola]
c56179.graph_c0	6.48E-08	-1.5046	down	bzip transcription factor [Diplodia corticola]
c74577.graph_c0	3.21E-12	-1.8769	down	hypothetical protein UCDDS831_g04100 [Diplodia
c90174.graph_c0	9.19E-64	1.10651	up	unknown [Glycine max]
c66152.graph_c0	8.11E-12	-1.8729	down	hypothetical protein DHEL01_v211074 [Diaporthe
c86622.graph_c0	3.76E-07	-1.3844	down	hypothetical protein glysoja_026788 [Glycine soja]
c38172.graph_c0	2.60E-12	-1.9175	down	uncharacterized protein FRV6_00773 [Fusarium ox
c53869.graph_c0	4.81E-14	-2.0618	down	hypothetical protein BK809_0007877 [Diplodia ser
c77801.graph_c0	2.80E-12	-1.8836	down	zinc finger transcription factor ace1 [Diplodia cortic
c57337.graph_c0	5.71E-09	-1.589	down	extracellular cellulase allergen asp f7 [Fusarium lan
c90975.graph_c0	5.31E-43	1.84295	up	PREDICTED: putative glucose-6-phosphate 1-epim
c67910.graph_c0	1.04E-22	-2.6281	down	hypothetical protein DHEL01_v200454 [Diaporthe

c92230.graph_c3	2.58E-44	-1.1029	down	ethylene-responsive transcription factor RAP2-1-lik
c89011.graph_c1	1.49E-06	1.0305	up	ribulose 1,5-bisphosphate carboxylase/oxygenase la
c70220.graph_c0	0.00012	-1.0944	down	hypothetical protein FPOA_01915 [Fusarium poae]
c81127.graph_c1	5.30E-06	1.11761	up	Agmatine coumaroyltransferase [Glycine soja]
c96464.graph_c0	7.69E-05	-1.0862	down	putative glycoside hydrolase family 3 [Diplodia seri
c54640.graph_c0	8.08E-08	1.40691	up	hypothetical protein glysoja_004713 [Glycine soja]
c39041.graph_c0	1.01E-09	-1.6704	down	neutral amino acid [Diplodia corticola]
c79379.graph_c0	7.66E-06	1.12261	up	phosphoenolpyruvate carboxylase kinase 1-like [Ca
c84375.graph_c0	0.00025	-1.0314	down	hypothetical protein UCDDS831_g00380 [Diplodia
c38457.graph_c0	1.84E-20	-2.4995	down	proteinase T [Diaporthe helianthi]
c85250.graph_c0	8.92E-06	-1.1441	down	fucoxanthin chlorophyll a /c binding protein [Hetero
c85264.graph_c0	0.00031	1.03953	up	uncharacterized protein FMAN_10507 [Fusarium n
c92950.graph_c3	5.85E-67	-2.192	down	hypothetical protein POPTR_0016s05500g [Populu
c92387.graph_c0	5.21E-27	1.6954	up	BAG family molecular chaperone regulator 6 [Caja
c95527.graph_c0	4.49E-18	-2.3519	down	--
c89171.graph_c0	8.51E-06	1.19637	up	60S ribosomal protein L7a [Symbiodinium microad
c54364.graph_c0	3.84E-15	1.28164	up	PREDICTED: WAT1-related protein At5g07050-li
c76585.graph_c0	2.62E-70	1.61807	up	probable inorganic phosphate transporter 1-5 [Cajan
c89362.graph_c0	2.03E-12	1.66936	up	primary amine oxidase [Vigna radiata var. radiata]
c92812.graph_c0	5.51E-05	1.1497	up	uncharacterized protein LOC100810937 [Glycine m
c78770.graph_c0	7.36E-41	-1.2005	down	hypothetical protein TSUD_367760 [Trifolium subt
c93835.graph_c0	5.58E-15	1.28115	up	PREDICTED: probable rhamnogalacturonate lyase
c96082.graph_c0	7.34E-26	-2.792	down	hypothetical protein DHEL01_v212544 [Diaporthe
c61457.graph_c0	5.50E-12	-1.8887	down	putative cellobiose dehydrogenase [Diplodia seriata
c37660.graph_c0	3.64E-11	-1.8213	down	hypothetical protein UCDDS831_g01626 [Diplodia
c80849.graph_c0	9.44E-26	-2.6687	down	RNA-binding domain-containing protein [Melinion
c81859.graph_c0	1.90E-87	-2.9203	down	probable inactive poly [ADP-ribose] polymerase SF
c54566.graph_c0	2.39E-07	1.40245	up	PREDICTED: protein BEARSKIN2-like [Vigna an
c95338.graph_c0	5.86E-20	-2.4588	down	scytalone dehydratase [Diplodia corticola]
c74527.graph_c1	4.45E-08	-1.2863	down	PREDICTED: probable receptor-like protein kinase
c81665.graph_c0	7.49E-12	1.049	up	PREDICTED: probable pectate lyase 12 [Glycine m
c38207.graph_c0	1.80E-28	-2.8538	down	Laccase-2, partial [Diplodia seriata]
c75180.graph_c1	1.99E-14	1.29992	up	photosystem I chlorophyll a/b-binding protein 6, ch
c81194.graph_c0	3.50E-14	1.11043	up	macrophage migration inhibitory factor homolog [C
c82269.graph_c0	2.97E-06	1.30816	up	putative serine/threonine-protein kinase NAK [Caja
c91260.graph_c0	5.10E-09	-1.18	down	hypothetical protein FOXB_08964 [Fusarium oxysp
c83885.graph_c0	4.12E-06	-1.297	down	transcription factor [Fusarium langsethiae]
c86049.graph_c0	1.37E-08	-1.5687	down	uncharacterized protein FFUJ_12625 [Fusarium fuj
c91693.graph_c0	1.62E-10	-1.4137	down	nucleosome assembly protein 1-like 1 [Fusarium ve
c95302.graph_c0	1.49E-10	1.50866	up	leguminosin group486 secreted peptide [Medicago
c82001.graph_c0	1.01E-10	-1.7706	down	Transcription factor Opi1 [Macrophomina phaseolin
c56903.graph_c0	1.94E-11	-1.8379	down	hypothetical protein VM1G_08555 [Valsa mali]
c55234.graph_c0	2.02E-08	1.15981	up	amino acid permease 2-like protein [Trifolium prate
c77237.graph_c0	6.71E-17	-2.2699	down	putative beta-glucanosyltransferase [Diplodia seriat
c97420.graph_c0	7.75E-11	-1.7814	down	hypothetical protein DHEL01_v204821 [Diaporthe
c79317.graph_c0	5.05E-19	-2.4146	down	putative woronin body major protein [Diaporthe am

c37517.graph_c0	1.20E-10	-1.7695	down	putative had superfamily hydrolase [Diplodia seriat
c37716.graph_c0	2.72E-26	-2.8159	down	putative gpi anchored serine-threonine rich protein
c96418.graph_c0	4.94E-11	-1.7991	down	alpha beta-hydrolase [Diplodia corticola]
c78591.graph_c0	1.01E-15	1.62042	up	PREDICTED: CASP-like protein 6 [Lupinus angus
c80922.graph_c0	2.38E-05	1.1951	up	probable S-adenosylmethionine-dependent methylt
c74731.graph_c0	1.13E-43	1.63745	up	PREDICTED: triacylglycerol lipase 2-like [Glycine
c90015.graph_c0	2.55E-06	-1.1933	down	hypothetical protein AU210_000846 [Fusarium ox
c89403.graph_c0	2.17E-22	-2.4432	down	PREDICTED: serine/threonine-protein phosphatase
c87941.graph_c1	2.80E-10	-1.7422	down	probable caspase [Fusarium proliferatum ET1]
c85455.graph_c0	1.96E-59	1.02583	up	hypothetical protein PHAVU_011G125300g [Phas
c73914.graph_c0	1.45E-37	-3.295	down	putative alcohol dehydrogenase [Diaporthe ampelin
c54211.graph_c0	2.77E-23	1.69025	up	acetyltransferase (GNAT) domain-containing prote
c81862.graph_c0	8.78E-16	-2.1937	down	hypothetical protein BK809_0001124 [Diplodia ser
c90714.graph_c0	3.47E-08	1.49954	up	hypothetical protein CVT26_012180 [Gymnopilus
c53827.graph_c0	4.34E-22	-2.5938	down	hypothetical protein DHEL01_v210943 [Diaporthe
c73858.graph_c0	1.19E-12	-1.9502	down	cofilin [Diplodia corticola]
c86019.graph_c0	2.47E-12	-1.8713	down	unnamed protein product [Fusarium sp. FIESC_5 C
c84907.graph_c0	2.79E-14	1.44164	up	cinnamyl alcohol dehydrogenase-like protein [Medi
c74390.graph_c0	9.26E-08	1.06416	up	PREDICTED: probable xyloglucan endotransgluco
c40098.graph_c0	1.32E-12	-1.943	down	SPFH domain/Band 7 family protein [Diaporthe he
c37508.graph_c0	9.47E-34	-3.1446	down	transcriptional regulator prz1 [Diaporthe helianthi]
c82542.graph_c0	8.09E-62	1.06963	up	PREDICTED: vicianin hydrolase [Glycine max]
c70363.graph_c0	#######	-2.136	down	1-aminocyclopropane-1-carboxylate oxidase 1-like
c85596.graph_c0	8.07E-18	-1.8082	down	putative non-histone chromosomal protein 6 [Diplo
c96984.graph_c0	1.08E-05	-1.2277	down	Transcription factor MADS-box [Macrophomina p
c60932.graph_c0	2.73E-10	-1.7274	down	C2H2 type zinc finger domain-containing protein [
c95484.graph_c0	5.55E-30	-2.9885	down	hypothetical protein TCE0_044f16403 [Talaromyc
c95389.graph_c0	0.00014	-1.0326	down	glycoside hydrolase family 43 protein [Diplodia co
c86027.graph_c0	1.44E-88	-1.1873	down	Histone H1 [Cajanus cajan]
c38514.graph_c0	3.79E-32	-3.0786	down	hypothetical protein TRIATDRAFT_225995 [Trich
c64640.graph_c0	1.24E-22	1.61217	up	PREDICTED: uncharacterized protein LOC100811
c90101.graph_c0	2.12E-08	1.55823	up	40S ribosomal protein S3-3 [Achlya hypogyna]
c92608.graph_c0	1.98E-05	-1.039	down	outward rectifying K+ channel [Ammopiptanthus n
c82951.graph_c1	5.44E-07	1.07187	up	PREDICTED: protein EXORDIUM-like 2 [Glycine
c38008.graph_c0	1.64E-22	1.18399	up	caffeic acid 3-O-methyltransferase 1-like [Cajanus
c95599.graph_c0	2.51E-11	-1.8095	down	Glycoside hydrolase family 43 [Macrophomina pha
c70554.graph_c0	0.0001	-1.0181	down	amino acid transporter [Diplodia corticola]
c75624.graph_c0	1.47E-56	1.3152	up	chitinase [Medicago truncatula]
c81152.graph_c0	#######	1.50956	up	ribulose bisphosphate carboxylase/oxygenase activ
c81367.graph_c0	6.52E-20	-1.3491	down	PREDICTED: gibberellin 20-oxidase 1 isoform X1
c93477.graph_c2	5.62E-05	-1.106	down	UDP-glucosyltransferase family protein [Medicago
c74597.graph_c0	1.27E-07	1.08573	up	hypothetical protein LR48_Vigan08g173600 [Vign
c74059.graph_c0	2.11E-35	1.40537	up	PREDICTED: uncharacterized protein At4g22758 [
c89339.graph_c0	5.53E-08	-1.2734	down	unnamed protein product [Fusarium venenatum]
c75084.graph_c0	1.89E-10	1.47763	up	Derlin-1 [Glycine soja]
c84760.graph_c0	0.00046	-1.0096	down	unnamed protein product [Fusarium sp. FIESC_5 C

c67612.graph_c0	2.63E-23	-1.3077	down	PREDICTED: F-box protein GID2-like [Glycine m
c80805.graph_c0	2.25E-09	1.64908	up	probable PGU1-Endo-polygalacturonase [Fusarium
c83426.graph_c0	2.85E-06	-1.3185	down	citrate-binding protein [Arachis ipaensis]
c75405.graph_c0	#######	1.04548	up	RecName: Full=UDP-glucose 4-epimerase; AltNan
c98510.graph_c0	1.79E-09	-1.6443	down	hypothetical protein ALT_5891 [Aspergillus lentulu
c77572.graph_c0	4.45E-05	-1.1611	down	hypothetical protein BK809_0006981 [Diplodia ser
c74062.graph_c0	1.94E-08	-1.5292	down	putative mfs transporter [Diaporthe ampelina]
c85280.graph_c0	7.95E-06	-1.2367	down	hypothetical protein ASCRUDRAFT_43254 [Asco
c82532.graph_c0	1.93E-07	1.45452	up	predicted protein [Hordeum vulgare subsp. vulgare]
c48895.graph_c0	0.00045	1.00728	up	PREDICTED: protein YLS9 [Glycine max]
c36914.graph_c0	1.31E-14	-2.1059	down	benzoate 4-monooxygenase cytochrome P450 [Diap
c81720.graph_c0	2.32E-25	-1.0319	down	hypothetical protein LR48_Vigan10g006100 [Vigna
c66667.graph_c0	3.47E-12	-1.9051	down	hypothetical protein DHEL01_v205818 [Diaporthe
c75832.graph_c0	9.81E-05	1.01496	up	beta-1,3-galactosyltransferase 6 [Cajanus cajan]
c80347.graph_c1	5.42E-10	1.10296	up	putative anaphase-promoting complex subunit 11 ri
c81248.graph_c0	9.10E-21	1.09229	up	PREDICTED: 1-aminocyclopropane-1-carboxylate
c93926.graph_c0	1.20E-06	1.15508	up	SCOF-1, partial [Glycine max]
c78692.graph_c0	5.51E-05	-1.1242	down	hypothetical protein MPH_03925 [Macrophomina p
c76248.graph_c0	4.07E-08	1.21585	up	uncharacterized protein LOC109816422 [Cajanus c
c96635.graph_c0	2.60E-28	-2.9094	down	nitrate reductase [Diaporthe helianthi]
c67597.graph_c0	3.84E-13	1.86336	up	PREDICTED: NAD(P)H-quinone oxidoreductase s
c83414.graph_c0	8.73E-08	1.43192	up	endo-1,3-1,4-beta-D-glucanase-like protein, partial
c55708.graph_c0	1.19E-09	1.57587	up	hypothetical protein glysoja_022994 [Glycine soja]
c87363.graph_c0	6.33E-41	1.61131	up	PREDICTED: hevamine-A-like [Glycine max]
c85824.graph_c0	1.39E-08	-1.5734	down	uncharacterized protein FPRO_12092 [Fusarium pr
c95211.graph_c0	8.77E-38	-1.2591	down	hypothetical protein [Lotus japonicus]
c37908.graph_c0	2.29E-24	-2.6689	down	GPR1/FUN34/yaaH [Macrophomina phaseolina MS
c70394.graph_c0	1.87E-08	-1.5499	down	c6 finger domain-containing protein [Diplodia corti
c76476.graph_c0	2.83E-12	1.40462	up	hypothetical protein TanjilG_10357 [Lupinus angus
c96132.graph_c0	2.62E-10	-1.7297	down	NAD(P)-binding protein [Cenococcum geophilum
c96266.graph_c0	2.14E-10	-1.7387	down	O-methyltransferase [Paraphaeosphaeria sporulosa]
c81647.graph_c0	3.73E-10	1.38425	up	protein DOWNY MILDEW RESISTANCE 6-like [
c95581.graph_c0	1.13E-27	-2.8861	down	protein of unknown function DUF4243 [Penicillium
c81737.graph_c0	5.99E-44	2.13924	up	PREDICTED: LOW QUALITY PROTEIN: organic
c96129.graph_c0	2.42E-11	-1.8292	down	putative coactivator bridging factor 1 [Diaporthe an
c70872.graph_c0	9.47E-82	-1.7598	down	PREDICTED: uncharacterized protein LOC100778
c88805.graph_c1	1.50E-19	-1.4382	down	kelch repeat-containing protein At3g27220-like [Ca
c55588.graph_c0	1.11E-14	-2.0293	down	glycoside hydrolase family 28 protein [Diplodia cor
c74832.graph_c0	9.59E-34	-3.144	down	cellodextrin transport-2 [Diaporthe helianthi]
c78362.graph_c0	5.36E-21	-2.5315	down	IDI4 [Diaporthe helianthi]
c57179.graph_c0	2.37E-14	-2.0863	down	glycoside hydrolase family 20 protein [Diplodia cor
c68628.graph_c0	8.00E-41	-3.3009	down	putative oligopeptide transporter [Diplodia seriata]
c76158.graph_c0	1.90E-96	1.4583	up	PREDICTED: disease resistance response protein 2
c89179.graph_c0	4.86E-15	1.37774	up	unknown [Glycine max]
c90062.graph_c1	2.40E-06	-1.3263	down	polyprotein [Cajanus cajan]
c85645.graph_c2	2.40E-12	-1.7821	down	plasma membrane h+-atpase pma1 [Diplodia cortic

c87048.graph_c0	1.80E-09	-1.6099	down	putative mitochondrial dna replication protein [Dipl
c87126.graph_c0	1.60E-05	1.18514	up	NB-ARC domain protein [Medicago truncatula]
c75641.graph_c0	4.30E-05	-1.0542	down	PREDICTED: uncharacterized protein LOC102662
c81591.graph_c1	5.26E-06	1.21317	up	hypothetical protein L195_g014621, partial [Trifoli
c89102.graph_c0	7.34E-18	1.19335	up	hydroquinone glucosyltransferase-like protein [Trif
c97760.graph_c0	4.20E-10	-1.7093	down	peptidase S41 family protein [Diaporthe helianthi]
c79664.graph_c0	1.10E-30	1.74206	up	PREDICTED: probable NAD(P)H dehydrogenase (
c95531.graph_c0	8.29E-10	1.24454	up	uncharacterized protein LOC100500462 [Glycine m
c75993.graph_c0	1.15E-17	1.37712	up	Protein TIFY 3B [Cajanus cajan]
c82152.graph_c0	4.10E-12	1.87454	up	hypothetical protein FOC4_g10015249 [Fusarium c
c86696.graph_c0	3.92E-08	1.17585	up	PREDICTED: chitinase 2-like [Cicer arietinum]
c94349.graph_c1	3.63E-21	-2.1711	down	chitin synthase [Fusarium oxysporum FOSC 3-a]
c79371.graph_c0	2.52E-14	1.38753	up	Nodule Cysteine-Rich (NCR) secreted peptide [Me
c38551.graph_c0	8.92E-11	-1.7871	down	putative extracellular serine-rich protein [Diplodia s
c38106.graph_c0	5.39E-06	1.00691	up	PREDICTED: uncharacterized protein LOC100786
c77617.graph_c0	#######	1.24044	up	hypothetical protein GLYMA_03G109900 [Glycine
c71918.graph_c0	1.20E-10	-1.7636	down	high-affinity nicotinic acid transporter [Diaporthe h
c97704.graph_c0	3.08E-12	-1.9142	down	putative copper radical oxidase [Diplodia seriata]
c83336.graph_c0	1.93E-13	1.1445	up	zinc finger AN1 domain-containing stress-associate
c94857.graph_c0	1.42E-37	-2.3203	down	elongation factor 3 [Fusarium langsethiae]
c82244.graph_c0	1.59E-41	1.01729	up	uncharacterized protein LOC106761101 [Vigna rad
c42891.graph_c0	1.54E-06	1.09151	up	PREDICTED: probable calcium-binding protein CM
c80387.graph_c0	0.00014	1.08738	up	Dehydration-responsive protein RD22 [Glycine soja
c97086.graph_c0	4.42E-19	-2.4159	down	hypothetical protein UCDDA912_g04436 [Diaporth
c57327.graph_c0	2.74E-22	-2.6045	down	hypothetical protein DHEL01_v202806 [Diaporthe
c80722.graph_c1	5.75E-68	1.02742	up	hypothetical protein LR48_Vigan635s004000 [Vign
c85159.graph_c0	3.95E-09	1.0131	up	uncharacterized protein LOC100816003 [Glycine m
c86912.graph_c0	2.89E-14	-1.9924	down	hypothetical protein TanjilG_10332 [Lupinus angus
c88218.graph_c4	4.78E-17	1.0096	up	G-type lectin S-receptor-like serine/threonine-prote
c90932.graph_c0	4.48E-24	-1.8442	down	formate dehydrogenase [Diaporthe helianthi]
c94712.graph_c4	#######	-1.0681	down	cytochrome P450 71A1 isoform X1 [Vigna radiata
c71496.graph_c0	2.88E-11	-1.8211	down	hypothetical protein BK809_0001811 [Diplodia ser
c97451.graph_c0	1.30E-11	-1.8585	down	amino acid transporter [Diplodia corticola]
c95660.graph_c0	1.66E-54	-3.8431	down	pectate lyase [Diplodia corticola]
c91276.graph_c0	3.55E-09	1.41103	up	sucrose transport protein SUC8-like [Cajanus cajan
c72479.graph_c0	9.13E-07	1.37777	up	Arabinogalactan peptide 16 [Glycine soja]
c81545.graph_c1	7.53E-14	1.28538	up	hypothetical protein glysoja_002197 [Glycine soja]
c78238.graph_c1	7.04E-19	-2.3562	down	apses transcription factor [Diplodia corticola]
c79332.graph_c0	6.75E-16	1.25814	up	PREDICTED: cysteine-rich repeat secretory protein
c88207.graph_c0	5.46E-10	-1.1533	down	alcohol dehydrogenase 1 [Fusarium verticillioides 7
c73174.graph_c0	2.57E-08	1.16808	up	Kunitz type trypsin inhibitor / Alpha-fucosidase [M
c95206.graph_c0	2.39E-09	1.29865	up	Retrovirus-related Pol polyprotein from transposon
c38513.graph_c0	1.56E-13	-2.0207	down	glycoside hydrolase family 10 protein [Diplodia cor
c38291.graph_c0	4.00E-22	-2.5785	down	WSC domain-containing protein 2 [Diplodia seriata
c36995.graph_c0	2.09E-08	-1.5423	down	hypothetical protein UCDDS831_g02885 [Diplodia
c41324.graph_c0	4.31E-24	-2.7024	down	hypothetical protein MYCTH_2305635 [Thermothe

c82247.graph_c0	4.70E-16	2.10798	up	uncharacterized protein FMAN_08196 [Fusarium n
c95711.graph_c0	9.67E-84	-4.4786	down	glycoside hydrolase family 61 protein [Diplodia cor
c77829.graph_c0	3.15E-43	-3.4236	down	high-affinity glucose transporter RGT2 [Diaporthe l
c77653.graph_c0	4.16E-09	1.05834	up	ethylene-responsive transcription factor 5-like prote
c82007.graph_c0	8.45E-19	-2.2969	down	metacaspase a [Diplodia corticola]
c98223.graph_c0	4.74E-11	-1.8024	down	hypothetical protein DHEL01_v212357 [Diaporthe
c95462.graph_c0	1.60E-38	-3.3151	down	acyl-synthetase [Diplodia corticola]
c73196.graph_c0	1.55E-11	1.70598	up	hypothetical protein glysoja_042914 [Glycine soja]
c73075.graph_c0	4.06E-45	1.86065	up	PREDICTED: myb-related protein 308-like [Glycin
c95667.graph_c0	2.23E-22	-2.6116	down	short-chain dehydrogenase [Talaromyces cellulolyti
c95266.graph_c0	4.46E-65	3.25245	up	basic 7S globulin precursor [Glycine max]
c91713.graph_c0	4.02E-17	-1.7841	down	transketolase [Diaporthe helianthi]
c73943.graph_c2	8.26E-11	-1.7788	down	peroxisomal copper amine oxidase [Diaporthe helia
c84635.graph_c0	1.72E-85	-1.1335	down	PREDICTED: putative receptor protein kinase ZmP
c96175.graph_c0	9.72E-18	-2.3295	down	hypothetical protein DHEL01_v212941 [Diaporthe
c78050.graph_c0	3.81E-18	-2.3559	down	putative salicylate hydroxylase [Diaporthe ampelina
c57677.graph_c0	7.38E-27	-2.8199	down	Unsaturated rhamnogalacturonyl hydrolase YteR [D
c98116.graph_c0	5.74E-12	-1.8848	down	quinate permease [Diaporthe helianthi]
c88157.graph_c0	4.82E-07	1.05334	up	PREDICTED: uncharacterized protein LOC108347
c81388.graph_c1	#######	1.1572	up	uncharacterized protein LOC106780414 [Vigna rad
c80388.graph_c0	2.06E-35	1.06198	up	anthocyanidin reductase ((2S)-flavan-3-ol-forming)
c83059.graph_c1	#######	1.38686	up	Putative glutathione S-transferase [Glycine soja]
c88986.graph_c0	1.22E-06	1.34004	up	hypothetical protein PHAVU_004G101100g [Phase
c80803.graph_c0	7.48E-31	1.14901	up	PREDICTED: peroxidase 72-like [Glycine max]
c54638.graph_c0	9.72E-92	1.9073	up	hypothetical protein GLYMA_04G010900 [Glycine
c54061.graph_c0	#######	-2.2735	down	PREDICTED: chitotriosidase-1-like [Lupinus angu
c98211.graph_c0	5.08E-12	-1.8914	down	hypothetical protein DHEL01_v209314 [Diaporthe
c95476.graph_c0	6.33E-41	-3.3212	down	Galactose-binding domain-like protein [Macrophom
c79479.graph_c0	3.14E-41	2.28	up	hypothetical protein glysoja_042282, partial [Glycin
c83456.graph_c0	1.86E-07	1.32709	up	organic cation/carnitine transporter 3-like [Cajanus
c90199.graph_c1	1.14E-24	1.10574	up	Hypothetical protein glysoja_004362 [Glycine soja]
c77663.graph_c1	4.90E-09	1.29769	up	PREDICTED: ethylene-responsive transcription fac
c69533.graph_c0	2.46E-13	-1.9863	down	hypothetical protein MPH_06284 [Macrophomina p
c60659.graph_c2	7.07E-12	-1.8787	down	putative nadh:flavin oxidoreductase nadh oxidase [
c39131.graph_c0	8.89E-06	1.11019	up	ultraviolet-B-repressible protein [Medicago truncatu
c83977.graph_c0	1.83E-24	1.07103	up	PREDICTED: tetrahydrocannabinolic acid synthase
c78345.graph_c0	1.66E-38	2.39062	up	probable serine/threonine-protein kinase WNK5 iso
c55112.graph_c0	#######	3.47623	up	PREDICTED: L-ascorbate oxidase homolog [Glyci
c95517.graph_c0	4.37E-19	-2.4109	down	glycoside hydrolase family 61 protein [Diplodia cor
c82933.graph_c0	2.03E-09	-1.6552	down	putative glucose-6-phosphate 1-dehydrogenase prot
c91421.graph_c2	1.61E-80	-2.7276	down	hypothetical protein DHEL01_v200924 [Diaporthe
c65367.graph_c0	1.48E-11	-1.8553	down	Transcriptional activator HAP2 [Diplodia seriata]
c95705.graph_c0	9.70E-26	-2.7861	down	putative malate dehydrogenase [Diaporthe ampelina
c72404.graph_c0	1.58E-50	1.1633	up	UGT1 [Pueraria montana var. lobata]
c95280.graph_c0	2.71E-88	-4.6538	down	hypothetical protein BKCO1_610009 [Diplodia cor
c82587.graph_c0	4.95E-72	1.51655	up	PREDICTED: protein MOTHER of FT and TFL1 [

c96863.graph_c0	1.56E-13	-2.021	down	cytochrome P450 [Meliniomyces bicolor E]
c58152.graph_c1	2.23E-07	1.30411	up	uncharacterized protein LOC100808294 precursor [
c93465.graph_c0	3.66E-07	-1.3698	down	fatty acid synthase alpha subunit [Fusarium equiseti
c86211.graph_c0	5.49E-35	1.02986	up	putative LOV domain-containing protein [Codorioc
c71677.graph_c0	1.47E-17	-2.3082	down	Short-chain dehydrogenase/reductase SDR [Macrop
c95289.graph_c0	5.80E-35	2.62499	up	hypothetical protein PHAVU_005G052500g [Phase
c68740.graph_c0	1.09E-11	1.6595	up	PREDICTED: O-glucosyltransferase rumi homolog
c71762.graph_c0	3.89E-35	-3.1998	down	tall aerial hyphae-3 [Diaporthe helianthi]
c36775.graph_c0	1.61E-13	-2.0183	down	putative mfs transporter [Diaporthe ampelina]
c86865.graph_c0	1.55E-16	1.18424	up	PREDICTED: protein trichome birefringence-like 4
c84536.graph_c0	1.57E-41	3.18995	up	hypothetical protein PHAVU_007G070400g [Phase
c54578.graph_c0	#######	-4.8091	down	PREDICTED: vestitone reductase-like isoform X2
c40248.graph_c1	1.40E-14	-2.1037	down	putative hlh transcription factor [Diaporthe ampelin
c57117.graph_c0	4.50E-23	-2.6486	down	mfs aflatoxin efflux [Diplodia corticola]
c83167.graph_c0	2.51E-11	-1.8312	down	putative nadp-dependent alcohol dehydrogenase 6 [
c57734.graph_c0	2.62E-24	-2.7128	down	hypothetical protein DHEL01_v200533 [Diaporthe
c85349.graph_c0	4.56E-06	1.28495	up	hypothetical protein PHAVU_007G181400g [Phase
c77457.graph_c0	1.20E-11	-1.8634	down	malic enzyme [Diaporthe helianthi]
c95712.graph_c0	3.55E-15	-2.149	down	TPR domain-containing protein [Diaporthe helianth
c80914.graph_c0	9.72E-18	1.53798	up	protein DETOXIFICATION 43 [Cajanus cajan]
c86469.graph_c0	1.10E-30	-3.0172	down	putative glyoxalase family protein [Diaporthe ampe
c64981.graph_c0	7.21E-10	-1.7032	down	carbohydrate-binding module family 21 protein [Di
c69514.graph_c0	1.91E-56	-3.897	down	hypothetical protein DHEL01_v201796 [Diaporthe
c68684.graph_c0	2.85E-39	1.89969	up	hypothetical protein GLYMA_11G070200 [Glycine
c37938.graph_c0	7.00E-06	1.02153	up	ZCF37, putative [Medicago truncatula]
c83995.graph_c0	2.34E-37	-1.0931	down	2-aminoethanethiol dioxygenase-like protein [Trifo
c90275.graph_c2	6.51E-10	1.17692	up	hypothetical protein glysoja_041039 [Glycine soja]
c76429.graph_c0	1.32E-53	1.0392	up	hypothetical protein GLYMA_07G112000 [Glycine
c84420.graph_c0	2.59E-34	2.97927	up	hypothetical protein PHAVU_001G126400g [Phase
c68419.graph_c0	3.74E-36	1.11497	up	peroxidase N1-like isoform X1 [Durio zibethinus]
c92701.graph_c1	2.12E-22	1.67812	up	ABC transporter G family member 11 [Glycine soja
c89304.graph_c2	3.12E-16	-1.1533	down	PREDICTED: subtilisin-like protease SBT1.7 [Gly
c93515.graph_c0	0.00023	1.05812	up	fructose-bisphosphate aldolase, cytoplasmic isozym
c54131.graph_c0	9.40E-31	1.17082	up	unknown [Lotus japonicus]
c85972.graph_c0	2.94E-27	-2.037	down	hypothetical protein MVLG_03475 [Microbotryum
c70337.graph_c0	8.72E-18	1.94686	up	PREDICTED: dirigent protein 23-like [Glycine ma
c96325.graph_c0	1.68E-15	-2.1733	down	hypothetical protein DHEL01_v202661 [Diaporthe
c87747.graph_c0	1.44E-19	1.26775	up	PREDICTED: probable sarcosine oxidase [Vigna a
c79050.graph_c0	1.87E-19	-2.4184	down	c2h2 finger domain [Diplodia corticola]
c88873.graph_c2	3.08E-27	2.65307	up	PREDICTED: serpin-ZX-like [Ziziphus jujuba]
c69003.graph_c0	#######	1.47094	up	trypsin inhibitor [Apios americana]
c37812.graph_c0	4.30E-37	-3.2774	down	hypothetical protein M434DRAFT_22549 [Hypoxy
c76268.graph_c0	5.36E-28	1.02304	up	hypothetical protein PHAVU_008G0876001g, parti
c82026.graph_c0	1.56E-17	-2.3157	down	hypothetical protein NECHADRAFT_39694 [[Nec
c79424.graph_c0	4.39E-08	1.09404	up	hypothetical protein GLYMA_20G241800 [Glycine
c55588.graph_c1	6.35E-15	-2.0633	down	glycoside hydrolase family 28 protein [Diplodia cor

c89823.graph_c0	2.14E-05	1.01523	up	high affinity sulfate transporter 2-like [Cajanus caja
c81550.graph_c1	1.95E-09	1.60835	up	NEDD8 ultimate buster 1 [Arachis ipaensis]
c83101.graph_c0	4.84E-06	-1.2867	down	putative glucan endo-alpha-glucosidase agn1 [Diplo
c83664.graph_c0	7.59E-06	-1.2433	down	elongation factor 3 [Fusarium langsethiae]
c97542.graph_c0	5.35E-14	2.05798	up	hypothetical protein RSOLAG1IB_01585 [Rhizocto
c66307.graph_c0	5.20E-15	1.80744	up	unknown [Lotus japonicus]
c73256.graph_c0	5.14E-91	1.16967	up	hypothetical protein KK1_003131 [Cajanus cajan]
c70014.graph_c0	1.54E-07	-1.4552	down	alcohol oxidase [Diplodia corticola]
c90305.graph_c0	0.00034	1.01525	up	peroxidase [Trifolium pratense]
c89823.graph_c1	3.14E-16	1.33937	up	PREDICTED: high affinity sulfate transporter 2-lik
c53965.graph_c0	1.25E-08	1.42324	up	PREDICTED: cysteine-rich receptor-like protein ki
c85447.graph_c0	4.30E-15	-2.1428	down	quinone oxidoreductase [Diaporthe helianthi]
c81919.graph_c0	2.94E-14	2.0772	up	--
c57861.graph_c0	4.90E-12	-1.8985	down	hypothetical protein BKCO1_600094 [Diplodia cor
c78410.graph_c0	1.24E-06	1.3556	up	uncharacterized protein LOC109806873 [Cajanus c
c89978.graph_c0	3.49E-88	-1.6021	down	bZIP transcription factor TGA10 isoform X1 [Vign
c56186.graph_c0	1.82E-14	-2.0917	down	o-methyltransferase protein [Diplodia corticola]
c77660.graph_c0	1.89E-09	-1.6415	down	hypothetical protein DHEL01_v208488 [Diaporthe
c88097.graph_c0	1.13E-08	-1.3446	down	unnamed protein product [Fusarium venenatum]
c78272.graph_c0	2.77E-10	-1.7388	down	copper fist dna binding domain protein [Diplodia co
c95674.graph_c0	5.47E-55	-3.8748	down	linoleate diol synthase [Diaporthe helianthi]
c93237.graph_c0	3.82E-29	1.00232	up	PREDICTED: glycerate dehydrogenase [Cicer ariet
c82403.graph_c0	3.23E-07	-1.4157	down	eukaryotic aspartyl protease [Diaporthe helianthi]
c92060.graph_c0	8.25E-06	-1.1001	down	mitochondrial phosphate carrier protein [Ascherson
c95533.graph_c0	1.09E-12	1.04394	up	PREDICTED: UDP-arabinopyranose mutase 3-like
c77300.graph_c0	1.23E-43	1.1518	up	asparagine synthetase, partial [Lupinus luteus]
c95413.graph_c0	1.14E-15	2.0829	up	peamaclein-like [Cajanus cajan]
c93660.graph_c0	3.25E-06	-1.2987	down	ABC transporter ATP-binding protein ARB1 [Fusa
c82753.graph_c1	4.31E-30	1.88608	up	hypothetical protein PHAVU_011G005000g [Phase
c38109.graph_c0	4.69E-15	-2.1401	down	putative synaptobrevin [Diaporthe ampelina]
c79026.graph_c0	1.34E-43	1.15824	up	hypothetical protein VIGAN_08009100 [Vigna ang
c78737.graph_c0	9.45E-06	1.04502	up	unknown [Lotus japonicus]
c90948.graph_c0	1.52E-14	-1.6202	down	ATP-dependent RNA helicase eIF4A [Colletotrichu
c78554.graph_c0	3.37E-22	-2.4763	down	putative phytanoyl- dioxygenase protein [Neofusicc
c74611.graph_c0	#######	-1.3782	down	Non-symbiotic hemoglobin 1 [Glycine soja]
c87908.graph_c1	1.30E-17	1.06142	up	TPR repeat-containing thioredoxin TTL1-like prote
c85678.graph_c0	4.42E-22	2.21987	up	uncharacterized protein LOC109798425 [Cajanus c
c68077.graph_c0	1.21E-10	1.58016	up	hypothetical protein glysoja_040012 [Glycine soja]
c94463.graph_c3	2.01E-35	-2.2585	down	related to PHO89-Na+/phosphate co-transporter [Fu
c92537.graph_c0	3.34E-14	-1.2704	down	putative subtilisin-like proteinase spm1 [Diaporthe
c37989.graph_c0	1.08E-07	1.13205	up	uncharacterized protein LOC109795393 [Cajanus c
c82838.graph_c0	6.09E-12	1.88405	up	mannitol-1-phosphate dehydrogenase [Rhizoctonia
c96966.graph_c0	9.31E-14	-2.0391	down	glycogen phosphorylase [Diaporthe helianthi]
c74132.graph_c0	1.28E-32	1.05371	up	disease resistance-responsive (dirigent-like) family
c81847.graph_c0	2.03E-68	1.00819	up	hypothetical protein LR48_Vigan04g017600 [Vign
c81111.graph_c0	#######	1.43166	up	hypothetical protein GLYMA_06G145300 [Glycine

c93664.graph_c0	7.15E-10	1.54133	up	related to beta-mannosidase [Fusarium fujikuroi]
c86762.graph_c0	1.57E-44	-1.6683	down	PREDICTED: ethylene-responsive transcription fac
c78624.graph_c0	5.60E-22	-2.5297	down	General substrate transporter [Macrophomina phase
c95222.graph_c0	4.99E-16	-1.026	down	Retrovirus-related Pol polyprotein from transposon
c85505.graph_c0	6.49E-07	1.20355	up	PREDICTED: uncharacterized protein LOC109338
c87364.graph_c0	4.72E-20	-2.411	down	Plasma membrane fusion protein prm1 [Diplodia se
c74493.graph_c0	1.85E-16	-2.2311	down	putative gpi anchored cell wall protein [Diplodia se
c86231.graph_c1	1.09E-10	1.64418	up	hypothetical protein MANES_13G012700 [Maniho
c92014.graph_c0	1.49E-18	-2.358	down	bzip transcription factor [Diplodia corticola]
c36704.graph_c0	1.76E-12	-1.9296	down	hypothetical protein DHEL01_v206999 [Diaporthe
c87327.graph_c0	2.14E-07	-1.3289	down	Protein WAX2 [Glycine soja]
c84414.graph_c0	2.65E-07	-1.4399	down	ethylene-responsive transcription factor ERF017-lik
c97081.graph_c0	5.21E-10	-1.6999	down	hypothetical protein DHEL01_v205311 [Diaporthe
c42611.graph_c0	6.67E-15	-2.1283	down	hypothetical protein FNYG_13792 [Fusarium nyga
c37226.graph_c0	4.54E-12	-1.8962	down	conserved hypothetical protein [Talaromyces marne
c69337.graph_c0	5.42E-23	2.16359	up	uncharacterized protein FFB14_13255 [Fusarium fu
c91949.graph_c0	3.46E-21	2.23499	up	related to lactose permease [Fusarium proliferatum
c37476.graph_c0	1.01E-11	-1.8709	down	hypothetical protein BK809_0004686 [Diplodia ser
c70166.graph_c0	5.13E-17	2.12823	up	L-ascorbate oxidase-like [Cajanus cajan]
c36973.graph_c0	3.67E-16	-2.2117	down	cytochrome p450 [Diplodia corticola]
c89818.graph_c0	5.19E-07	-1.3097	down	putative pyruvate kinase [Fusarium fujikuroi]
c94075.graph_c0	1.26E-16	1.47535	up	flavonoid 3'-hydroxylase [Trifolium pratense
c74454.graph_c0	1.83E-07	-1.4485	down	40S ribosomal protein S20 [Diaporthe helianthi]
c90333.graph_c0	#######	-2.4974	down	putative esdc-like protein [Diaporthe ampelina]
c78163.graph_c0	1.10E-18	-2.3915	down	hypothetical protein DHEL01_v204253 [Diaporthe
c86901.graph_c2	2.77E-06	1.26093	up	Rhomboid protease gluP [Glycine soja]
c74796.graph_c0	4.23E-13	1.81391	up	Dehydration-responsive protein RD22 [Glycine soja
c36746.graph_c0	4.28E-09	-1.626	down	Beta-glucosidase 1B [Diplodia seriata]
c89732.graph_c1	2.54E-07	1.22876	up	MDIS1-interacting receptor like kinase 2-like [Caja
c54379.graph_c0	2.55E-14	1.66153	up	PREDICTED: auxin-responsive protein SAUR41-li
c79754.graph_c0	5.07E-13	-1.8062	down	unnamed protein product [Fusarium sp. FIESC_5 C
c89753.graph_c0	1.20E-11	-1.0124	down	PREDICTED: probable 1-aminocyclopropane-1-ca
c80611.graph_c0	1.10E-16	-2.2582	down	putative oxalate decarboxylase family bicupin [Dia
c80269.graph_c0	4.61E-06	1.24587	up	putative LRR receptor-like serine/threonine-protein
c83308.graph_c0	2.34E-08	-1.5198	down	putative glutamine synthetase [Diaporthe ampelina]
c83288.graph_c0	0.00022	1.06257	up	related to monocarboxylate transporter 2 [Fusarium
c70870.graph_c0	4.60E-08	1.11028	up	PREDICTED: probable acyl-activating enzyme 1, p
c38224.graph_c0	1.84E-27	-2.8692	down	hypothetical protein P174DRAFT_407796 [Asperg
c86215.graph_c0	1.61E-17	1.25522	up	hypothetical protein GLYMA_10G029600 [Glycine
c93106.graph_c1	#######	1.55244	up	peroxidase 4-like [Cajanus cajan]
c81439.graph_c0	#######	1.19185	up	hypothetical protein PHAVU_002G144800g [Phase
c84216.graph_c0	3.63E-07	1.32118	up	PREDICTED: uncharacterized protein LOC109339
c67934.graph_c0	5.82E-08	-1.5054	down	putative oligopeptidase family protein [Diplodia ser
c71320.graph_c0	0.00032	-1.0343	down	hypothetical protein FLAG1_02625 [Fusarium lang
c55707.graph_c0	1.24E-82	-4.5269	down	nitrate transporter [Diaporthe helianthi]
c76619.graph_c0	2.30E-82	-2.3523	down	unknown [Lotus japonicus]

c73359.graph_c0	4.63E-27	-2.8514	down	POT family protein [Diaporthe helianthi]
c76039.graph_c0	1.39E-23	1.48402	up	hypothetical protein PHAVU_003G256600g [Phase
c95848.graph_c0	1.79E-19	-2.4017	down	putative aldehyde dehydrogenase-like protein [Dipl
c91020.graph_c0	3.05E-06	-1.024	down	hypothetical protein FVEG_04839 [Fusarium vertic
c93971.graph_c0	3.03E-47	3.63712	up	laccase, multicopper oxidase, benzenediol:oxygen o
c67864.graph_c0	6.57E-08	1.50564	up	PREDICTED: uncharacterized protein LOC100819
c83659.graph_c0	3.61E-34	-3.1612	down	putative amid-like nadh [Diaporthe ampelina]
c38393.graph_c0	1.87E-24	-2.7262	down	E3 ubiquitin-protein ligase hulA [Diaporthe heliant
c87839.graph_c0	3.94E-16	2.21924	up	Benzoate 4-monooxygenase [Rhizoctonia solani]
c79072.graph_c0	1.81E-18	-2.3779	down	oligopeptide transporter [Diaporthe helianthi]
c71079.graph_c0	4.91E-45	1.2848	up	dirigent protein 22-like [Vigna radiata var. radiata]
c70306.graph_c0	5.47E-10	-1.7048	down	hypothetical protein MPH_10408 [Macrophomina p
c67447.graph_c0	2.79E-12	-1.9142	down	hypothetical protein FAVG1_08673 [Fusarium aver
c76846.graph_c0	1.11E-18	-2.2556	down	Exopolygalacturonase X-1 [Diplodia seriata]
c89065.graph_c0	3.07E-41	-1.0595	down	uncharacterized LOC100527907 precursor [Glycine
c84210.graph_c0	4.23E-10	1.23977	up	hypothetical protein glysoja_008380 [Glycine soja]
c88755.graph_c0	1.15E-17	-2.1153	down	probable indole-3-pyruvate monooxygenase YUCC
c76229.graph_c0	1.36E-11	-1.839	down	superoxide dismutase [Diplodia corticola]
c88177.graph_c0	1.40E-31	1.07673	up	PREDICTED: 2-oxoglutarate-dependent dioxygena
c38374.graph_c0	4.59E-16	-2.2142	down	hypothetical protein DHEL01_v210039 [Diaporthe
c79486.graph_c0	2.66E-07	-1.1487	down	wound-responsive family protein [Medicago trunca
c75460.graph_c0	5.55E-09	1.40826	up	Gibberellin 2-beta-dioxygenase 2 [Glycine soja]
c78621.graph_c1	1.76E-10	-1.7575	down	microsomal cytochrome b5 [Diaporthe helianthi]
c81350.graph_c0	3.48E-16	-1.9309	down	Protein ecm33, partial [Diplodia seriata]
c74296.graph_c0	2.05E-06	1.27899	up	uncharacterized protein LOC107489980 [Arachis d
c37037.graph_c0	3.85E-11	-1.8106	down	putative pci domain-containing protein [Diaporthe a
c69711.graph_c0	1.60E-31	1.31006	up	PREDICTED: isoliquiritigenin 2'-O-methyltr
c37710.graph_c0	3.91E-67	-4.1766	down	Uncharacterized protein T310_8106 [Rasamsonia e
c81180.graph_c1	2.36E-49	1.23929	up	unknown [Glycine max]
c99526.graph_c0	4.48E-07	-1.4029	down	hypothetical protein BKCO1_600037 [Diplodia cor
c39648.graph_c0	7.59E-12	-1.8746	down	hypothetical protein DHEL01_v200043 [Diaporthe
c53938.graph_c0	8.62E-55	-3.8559	down	hypothetical protein DHEL01_v209322 [Diaporthe
c83392.graph_c0	7.00E-53	1.88924	up	Omega-hydroxypalmitate O-feruloyl transferase [G
c89641.graph_c0	2.49E-10	1.30896	up	hypothetical protein GLYMA_15G015100 [Glycine
c76626.graph_c0	1.14E-84	1.09084	up	caffeic acid 3-O-methyltransferase-like [Glycine ma
c82672.graph_c0	#######	1.24362	up	PREDICTED: xyloglucan endotransglucosylase/hy
c89509.graph_c0	5.48E-18	-2.157	down	uncharacterized protein LOC109793247 isoform X
c69481.graph_c0	2.60E-14	-1.5354	down	cellulase [Phaseolus vulgaris]
c66282.graph_c1	7.22E-17	-2.2713	down	kelch repeat protein [Diaporthe helianthi]
c96147.graph_c0	3.14E-24	-2.7094	down	catalase/peroxidase HPI [Diaporthe helianthi]
c36752.graph_c0	3.25E-18	-2.3474	down	putative cytochrome p450 [Diplodia seriata]
c59007.graph_c0	3.37E-13	-1.9925	down	putative tripeptidyl-peptidase 1 precursor protein [N
c37946.graph_c0	3.27E-11	1.82463	up	GDSL esterase/lipase At1g74460-like [Cajanus caja
c95821.graph_c0	1.84E-16	-2.2432	down	hypothetical protein DHEL01_v204504 [Diaporthe
c88372.graph_c0	5.50E-12	1.35645	up	PREDICTED: uncharacterized protein LOC100798
c38503.graph_c0	5.86E-12	-1.886	down	S-adenosyl-L-methionine-dependent methyltransfer

c86752.graph_c0	5.17E-09	1.02813	up	protein TIC 62, chloroplastic [Cajanus cajan]
c83516.graph_c0	2.50E-13	-1.9283	down	MFS transporter, SP family, general alpha glucosid
c95520.graph_c0	4.73E-06	-1.2718	down	aldo keto reductase [Diplodia corticola]
c78830.graph_c0	3.79E-24	-2.4832	down	woronin body major protein [Diplodia corticola]
c80327.graph_c0	3.55E-33	1.33326	up	PREDICTED: uncharacterized protein LOC100793
c79131.graph_c0	3.37E-08	1.39655	up	PREDICTED: 3,9-dihydroxypterocarpan 6A-mono
c37939.graph_c0	2.63E-56	-3.5469	down	hypothetical protein BKCO1_5000209 [Diplodia co
c89534.graph_c0	5.75E-11	-1.442	down	PREDICTED: uncharacterized protein LOC100778
c76756.graph_c0	#######	1.01308	up	RecName: Full=Chalcone--flavonone isomerase 1;
c81764.graph_c0	1.01E-05	1.12455	up	hypothetical protein GLYMA_01G151100 [Glycine
c38447.graph_c0	9.63E-12	-1.8662	down	cfem domain-containing protein [Diplodia corticola
c92580.graph_c0	1.53E-46	1.46989	up	AP2 domain class transcription factor [Medicago tr
c85483.graph_c0	1.35E-40	-1.115	down	Receptor-like cytosolic serine/threonine-protein kin
c81298.graph_c0	#######	-1.2305	down	hypothetical protein PHAVU_002G270700g [Phase
c73144.graph_c0	3.35E-06	1.25896	up	RING-H2 finger protein ATL70-like [Cajanus cajar
c91465.graph_c1	3.52E-15	-1.7109	down	hypothetical protein glysoja_007873 [Glycine soja]
c73982.graph_c0	0.00016	1.06835	up	hypothetical protein FVEG_12410 [Fusarium vertic
c54822.graph_c0	4.14E-06	1.03268	up	organ-specific protein S2-like isoform X1 [Cajanus
c70349.graph_c0	7.93E-06	-1.2547	down	cation/H(+) antiporter 8-like [Cajanus cajan]
c84028.graph_c0	2.22E-14	-2.0642	down	b-zip transcription factor idi-4 [Diplodia corticola]
c86534.graph_c0	2.03E-17	1.3761	up	Protein CbbY, chromosomal [Glycine soja]
c38333.graph_c0	5.15E-23	-2.6441	down	non-anchored cell wall protein-1 [Diaporthe heliant
c73983.graph_c0	5.41E-10	1.22886	up	PREDICTED: uncharacterized protein LOC102670
c71051.graph_c0	2.13E-10	1.23873	up	hypothetical protein PHAVU_001G002100g [Phase
c85571.graph_c0	9.98E-08	1.22472	up	PREDICTED: NAC domain-containing protein 86-
c36744.graph_c0	7.44E-13	-1.9641	down	hypothetical protein DHEL01_v210839 [Diaporthe
c60438.graph_c0	6.03E-12	-1.8847	down	Chitin synthase D [Diplodia seriata]
c88722.graph_c0	5.71E-09	-1.1694	down	PREDICTED: inositol transporter 4 [Glycine max]
c40185.graph_c1	1.79E-11	-1.8418	down	aromatic amino acid aminotransferase [Diaporthe h
c95895.graph_c0	6.86E-16	-2.2016	down	oxalate decarboxylase oxdC [Diaporthe helianthi]
c54619.graph_c0	2.55E-07	-1.4405	down	hypothetical protein BKCO1_5200072 [Diplodia co
c37980.graph_c0	9.24E-12	1.80149	up	stigma-specific STIG1-like protein precursor [Glyci
c97248.graph_c0	2.16E-11	-1.8335	down	hypothetical protein DHEL01_v206822 [Diaporthe
c83328.graph_c0	2.65E-07	-1.3993	down	putative glycoside hydrolase subgroup catalytic cor

ıing protein [Diaporthe ampelina]

:acrophomina phaseolina MS6]

ajan]
cajan]
ta]
ncatula]
ne soja]
: 2 homolog 1, mitochondrial [Glycine max]

ratense]
helianthi]

ı seriata]
m parvum UCRNP2]
iata]
he ampelina]
]
x]
303 [Lupinus angustifolius]
eolus vulgaris]
nitochondrial [Glycine max]
lon sp. CI-4A]
copersici MN25]

orticola]
helianthi]
ograminearum CS3096]
liata var. radiata]
ja]
[Vigna angularis]

lorus]

ensis]

oxidorectuctase [Rhizoctonia solani]
ıa angularis]
eolus vulgaris]
eolus vulgaris]
a angularis]

2 [Cajanus cajan]

ia seriata]

ctonia solani]

ajan]

ike [Ziziphus jujuba]
ajan]
helianthi]
e [Lupinus angustifolius]
mai]
ctonia solani AG-3 Rhs1AP]
At5g47250 isoform X2 [Glycine max]

e max]
osphatase 9-like isoform X1 [Vigna angularis]
pelina]
2 [Diplodia seriata]
anthi]

050 [Vigna angularis]
ajan]

518 [Vigna angularis]
usicoccum parvum UCRNP2]
gloeosporioides Nara gc5]
]

e isoform X2 [Cajanus cajan]
oform X1 [Vigna angularis]

rticola]
lina]
terraneum]

nax]
orthe helianthi]

nax]

c-like [Glycine max]
he ampelina]
ularis var. angularis]
-like isoform X1 [Glycine max]
ta]

cajan]

eratum]
helianthi]
9]

ns]
max]
orthe helianthi]
helianthi]

[Neofusicoccum parvum UCRNP2]
ata]

e isoform X2 [Lupinus angustifolius]
CRNP2]

c-like [Vigna angularis]
helianthi]

naceum]

the helianthi]
atus IBT 16806]
eolus vulgaris]

helianthi]
[Glycine max]

585 [Lupinus angustifolius]

cremonium minimum UCRPA7]

e max]
erase GDPD1, chloroplastic-like [Vigna angularis]
nata]
eolus vulgaris]
na MS6]
ein [Diplodia corticola]
eolus vulgaris]
ke [Glycine max]

ajan]
ograminearum CS3096]
5-like [Glycine max]
icoccum parvum UCRNP2]
riata]
Glycine max]

e 2 [Cajanus cajan]
helianthi]
cajan]
the helianthi]
ctonia solani]

seriata]
helianthi]

elina]
606 [Glycine max]
hi]
oxysporum]
n proliferatum]
ci W106-1]
helianthi]

helianthi]

eolus vulgaris]
like [Glycine max]
seriata]
]
ne max]
eolus vulgaris]

helianthi]

iata]

/cine max]
m barbadense]

terraneum]

elomyces thermophila ATCC 42464]
/cine max]

600]
protein [Medicago truncatula]
helianthi]
catula]
omina phaseolina MS6]
mydosporia 170]

rticola]
liata var. radiata]

Vigna angularis]

]
isoform X1 [Glycine max]
ledicago truncatula]
helianthi]
Vigna radiata var. radiata]

syltransferase 1 [Glycine max]
eolus vulgaris]

isarium fujikuroi IMI 58289]
ne soja]
helianthi]
icatula]
ajan]
eolus vulgaris]
elomyces thermophila ATCC 42464]

ta var. radiata]
helianthi]
partial [Trifolium pratense]
4-like [Glycine max]
ıstic [Cajanus cajan]
[Diaporthe helianthi]
ula]
ɛ]

ɛrma otae CBS 113480]
ticola]
diata]
porthe ampelina]

liata var. radiata]
S3069]
[Vigna angularis]
[Carica papaya]

ajanus cajan]
]
ɪrotein [Cicer arietinum]
ɛ max]
um parvum UCRNP2]
hungensis]

.sicoccum parvum UCRNP2]

ı parvum UCRNP2]

ajan]
ıcilitator superfamily) [Fusarium proliferatum]
697 [Glycine max]
rthe ampelina]
[Glycine max]
ıporthe ampelina]
ıRR18-like [Glycine max]
ensis]

corticola]
s cajan]
ɛ max]

ograminearum CS3096]

6]
ci W106-1]

IBT 23096]

um oxysporum f. sp. cubense tropical race 4 54006]
orchidophilum]
ımpelina]
ıs ipaensis]

helianthi]
ase [Diplodia seriata]

orticola]
ᴾK1 [Glycine max]
helianthi]
helianthi]
orthe ampelina]
pyri]
e max]
ula]

jan]
helianthi]
616 [Glycine max]
ajan]

an]
ı seriata]
ass [Oryza sativa Japonica Group]

]
eriata]
eolus vulgaris]
oma neurophilia]
alyx motorius]
[Vigna angularis]
AG-3 Rhs1AP]

231 [Lupinus angustifolius]
]

kuroi IMI 58289]
illus novofumigatus IBT 16806]
phaseolina MS6]
048 [Glycine max]

threonine-protein kinase At5g24080 [Glycine max]
e protein 33 [Glycine soja]
e max]
be (strain 972 / ATCC 24843) GN=tef3 PE=1 SV=1 [Rhizoctonia solani AG-1 IB]
eolus vulgaris]

arietinum]
glectum]

stifolius]
a seriata]

nus angustifolius]
e max]
e-like [Glycine max]
ent 52, chloroplastic [Cajanus cajan]

octonia solani]
his ipaensis]
e max]

janus cajan]

Talaromyces marneffei ATCC 18224]
jan]

a]
297 family [Cajanus cajan]

x]
riata]

helianthi]
us cajan]

ıta]
inus angustifolius]
ng protein [Diplodia corticola]
n [Lupinus angustifolius]

lensis]
:rraneum]
]

›xygenase) [Fusarium mangiferae]
ngularis]

)-like isoform X3 [Glycine max]

:um]
:olus vulgaris]
cola]
›ccum parvum UCRNP2]
]
X2 [Glycine max]
:ajan]

·869 [Beta vulgaris subsp. vulgaris]

:S3069]
ike [Nicotiana attenuata]

var. radiata]

TNT 1-94, partial [Cajanus cajan]
he ampelina]
:ajan]

:omplex B 2, chloroplastic [Lupinus angustifolius]

x]
ıs]
oxidase [Glycine max]

iata]
s australis]
helianthi]
orthe ampelina]

ratense]

ıax]
cajan]
um verticillioides 7600]
helianthi]

otein [Pseudomassariella vexata]

ke [Glycine max]
phaseolina MS6]
a]

ıali]
ıangiferae]

the helianthi]
synthase 7-like [Glycine max]
ET3-like [Glycine max]
like isoform X2 [Glycine max]
P1 [Cajanus cajan]
lycine max]
AG-1 IA]
helianthi]
helianthi]
janus cajan]
seriata]
isoform X2 [Glycine max]
ani AG-1 IB]
cajan]
iata]
a]
in kinase [Glycine soja]

um f. sp. lycopersici 4287]
eudonana CCMP1335]
rticola]

ularis]

ne max]
mai]

eolus vulgaris]
homina phaseolina MS6]

;01020-like isoform X2 [Glycine max]

ajan]

cine soja]
protein At1g11290, chloroplastic-like isoform X2 [Glycine max]

m barbadense]
lobata]

TAMARI1 homolog [Glycine max]

ding protein 3-like [Vigna angularis]
iata]
orticola]

e max]
S3069]
helianthi]
Cajanus cajan]
helianthi]

baensis]

ajan]

elianthi]
ike [Glycine max]

[Diaporthe helianthi]
helianthi]
nium stellatum]

: 3b-like [Glycine max]
ilycine max]

ıorthe helianthi]
002 [Cicer arietinum]
[Glycine max]
ıase-like [Cajanus cajan]
: 2a-like [Lupinus angustifolius]
:ica]

. D-7 [Cicer arietinum]
ıelianthi]
igna radiata var. radiata]
.se DAO-like [Glycine max]

753 [Vigna angularis]

ɔform X1 [Glycine max]
ein [Diaporthe ampelina]
ı seriata]
ıthase [Aspergillus lentulus]

helianthi]
). No.11243]
606 isoform X2 [Glycine max]

helianthi]
·like [Glycine max]
ɔolus vulgaris]

ıngularis]

287 [Glycine max]

lopsis thaliana]
ıni]

ɔolus vulgaris]

ʼ044 isoform X1 [Glycine max]
ːlina]
ːe]
ikuroi IMI 58289]
ʼK1 [Cicer arietinum]

ı seriata]
he ampelina]
: receptor-like protein kinase At2g25790 [Glycine max]
:he ampelina]
ikuroi]

:um parvum UCRNP2]
C [Diaporthe helianthi]
ɔolus vulgaris]
ınus cajan]

m simmondsii]
ɔctonia solani]
helianthi]
rrestris NRRL 8126]
ɔolus vulgaris]
ılaris]
helianthi]
icola]

ɔolus vulgaris]
ıngiferae]

ı seriata]

helianthi]

:ysporum]
'iata]
ːola]
ıgsethiae]
ıerase [Cicer arietinum]
helianthi]

ιe [Cajanus cajan]
ırge subunit (chloroplast) [Ammopiptanthus mongolicus]

iata]

ıjanus cajan]
ı seriata]

osigma akashiwo]
ıangiferae]
ıs trichocarpa]
nus cajan]

lriaticum]
ke [Glycine max]
ıus cajan]

ıax]
terraneum]
B [Glycine max]
helianthi]
ı]
ı seriata]
ıyces bicolor E]
ƦO2 [Vigna radiata var. radiata]
gularis]

: At5g18500 [Glycine max]
ıax]

loroplastic [Cajanus cajan]
Cajanus cajan]
ınus cajan]
oorum Fo5176]

ikuroi IMI 58289]
:rticillioides 7600]
truncatula]
na MS6]

ense]
a]
helianthi]
ıpelina]

a]
[Diaporthe ampelina]

tifolius]
:ansferase At5g37990 isoform X1 [Arachis ipaensis]
: max]
/sporum f. sp. radicis-cucumerinum]
: 7 long form homolog [Glycine max]

:olus vulgaris]
ıa]
in [Glycine max]
iata]
dilepis]
helianthi]

:S3069]
icago truncatula]
sylase/hydrolase protein 23 [Cicer arietinum]
lianthi]

[Cajanus cajan]
dia seriata]
ıaseolina MS6]
Diaporthe helianthi]
:s cellulolyticus]
ticola]

ıoderma atroviride IMI 206040]
485 [Glycine max]

ıongolicus]
: max]
cajan]
seolina MS6]

ıse, chloroplastic-like [Cajanus cajan]
[Glycine max]
truncatula]
a angularis]
[Vigna angularis]

:S3069]

ax]
mangiferae]

ıe: Full=Galactowaldenase; AltName: Full=UDP-galactose 4-epimerase
ıs]
iata]

idea rubescens DSM 1968]
]

orthe helianthi]
a angularis]
helianthi]

ng-h2 finger protein [Lupinus angustifolius]
synthase [Vigna angularis]

haseolina MS6]
ajan]

ubunit N, chloroplastic-like [Glycine max]
[Trifolium pratense]

oliferatum ET1]

S6]
cola]
stifolius]
1.58]

Cajanus cajan]
ı occitanis]
cation/carnitine transporter 3-like [Glycine max]
ıpelina]
894 isoform X1 [Glycine max]
ıjanus cajan]
ticola]

ticola]

06-like [Cicer arietinum]

ola]

lodia seriata]

.648 [Glycine max]
um pratense]
olium pratense]

quinone) FQR1-like 2 [Glycine max]
nax]

oxysporum f. sp. cubense race 4]

dicago truncatula]
seriata]
184 [Glycine max]
e max]
elianthi]

ed protein 12 [Cajanus cajan]

liata var. radiata]
ML45 [Vigna angularis]
a]
he ampelina]
helianthi]
na angularis]
nax]
stifolius]
in kinase At4g27290 [Cajanus cajan]

var. radiata]
iata]

]

n 55 [Vigna angularis]
7600]
edicago truncatula]
TNT 1-94 [Cajanus cajan]
ticola]
a]
a seriata]
elomyces thermophila ATCC 42464]

ıangiferae]
:ticola]
helianthi]
:in [Glycine max]

helianthi]

ıe max]
ıcus]

ınthi]
ʾK1 [Glycine max]
helianthi]
ı]
)iplodia seriata]

117 [Vigna angularis]
liata var. radiata]
isoform X1 [Cajanus cajan]

:olus vulgaris]

: max]
stifolius]
helianthi]
nina phaseolina MS6]
ne soja]
cajan]
]
:tor 1B-like [Glycine max]
ɔhaseolina MS6]
Diplodia seriata]
ula]
:-like [Glycine max]
ɔform X2 [Cajanus cajan]
ne max]
:ticola]
ein [Neofusicoccum parvum UCRNP2]
helianthi]

ı]

:ticola]
Lupinus angustifolius]

[Glycine max]
i]
:alyx motorius]
›homina phaseolina MS6]
ɛolus vulgaris]
; isoform X1 [Glycine max]

43 [Glycine max]
ɛolus vulgaris]
[Glycine max]
ia]

Diplodia seriata]
helianthi]
ɛolus vulgaris]

ıi]

:lina]
plodia corticola]
helianthi]
ɛ max]

lium pratense]

ɛ max]
ɛolus vulgaris]

ı]
cine max]
ıe-like [Chenopodium quinoa]

lychnidis-dioicae p1A1 Lamole]
x]
helianthi]
ngularis]

'lon sp. CO27-5]
ial [Phaseolus vulgaris]
tria] haematococca mpVI 77-13-4]
ɛ max]
:ticola]

ın]

ɔdia seriata]

ɔnia solani AG-1 IB]

e [Glycine max]
nase 1 [Glycine max]

ticola]
ajan]
a radiata var. radiata]

helianthi]

ɔrticola]

inum]

ia aleyrodis RCEF 2490]
[Cicer arietinum]

rium verticillioides 7600]
eolus vulgaris]

ularis var. angularis]

ım chlorophyti]
ɔccum parvum UCRNP2]

in [Glycine soja]
ajan]

ısarium fujikuroi IMI 58289]
ampelina]
ajan]
solani AG-3 Rhs1AP]

protein precursor [Glycine max]
a angularis]
e max]

:tor ABI4-like [Lupinus angustifolius]
:olina MS6]
TNT 1-94 [Cajanus cajan]
543 [Lupinus angustifolius]
:riata]
riata]
ıt esculenta]

helianthi]

:e protein [Medicago truncatula]
helianthi]
mai]
:ffei ATCC 18224]
ıjikuroi]
ET1]
:iata]

:]

helianthi]

a]

ınus cajan]
ike [Glycine max]
:S3069]
rboxylate oxidase [Glycine max]
porthe ampelina]
kinase At1g06840 family, partial [Cajanus cajan]
|
mangiferae]
peroxisomal [Glycine max]
illus novofumigatus IBT 16806]
e max]

eolus vulgaris]
364 [Lupinus angustifolius]
:iata]
;sethiae]

ɘolus vulgaris]
odia seriata]
:illioides 7600]
ɔxidorectuctase [Rhizoctonia solani AG-1 IB]
'515 [Glycine max]

hi]

ɔhaseolina MS6]
ıaceum]

ɘ max]

A10 isoform X1 [Cajanus cajan]

ıse AOP3-like [Glycine max]
helianthi]
tula]

uranensis]
ımpelina]
ransferase-like [Glycine max]
mersonii CBS 393.64]

ticola]
helianthi]
helianthi]
lycine soja]
ɘ max]
ıx]
drolase 2 isoform X1 [Glycine max]
2 [Cajanus cajan]

Neofusicoccum parvum UCRNP2]
ın]
helianthi]
639 [Glycine max]
ːase [Glonium stellatum]

e:H+ symporter [Diaporthe helianthi]

250 isoform X1 [Glycine max]
oxygenase-like [Lupinus angustifolius]
orticola]
228 [Glycine max]
Short=Chalcone isomerase 1
e max]
a]
uncatula]
ase RBK1 [Glycine soja]
eolus vulgaris]
a]

cillioides 7600]
cajan]

hi]
552 [Glycine max]
eolus vulgaris]
like isoform X1 [Cicer arietinum]
helianthi]

elianthi]

orticola]
ine max]
helianthi]
e [Diplodia seriata]

Cluster_ID	FDR	log2FC	regulated	nr_annotation
c40224.graph_c0	7.18E-10	-1.1523	down	putative transmembrane alpha-helix domain-contai
c79802.graph_c0	#######	1.04881	up	hypothetical protein PHAVU_010G0470000g, parti
c82188.graph_c0	3.14E-24	-1.9526	down	pathogenesis associated protein Cap20, putative [M
c80757.graph_c0	2.01E-07	1.02902	up	glutathione S-transferase L3-like [Cajanus cajan]
c37669.graph_c0	9.88E-40	-2.5111	down	versicolorin reductase [Aspergillus udagawae]
c83015.graph_c0	7.95E-08	1.07726	up	hypothetical protein AU210_007785 [Fusarium ox
c75691.graph_c0	2.12E-07	-1.0082	down	uncharacterized protein LOC100798341 [Glycine m
c57963.graph_c0	5.89E-16	-1.5671	down	putative multicopper oxidase type 1 [Diplodia seria
c37927.graph_c1	1.29E-79	2.95746	up	2-alkenal reductase (NADP(+)-dependent)-like [Ca
c93561.graph_c1	2.81E-15	1.52478	up	apocytochrome b (mitochondrion) [Microspora stag
c76417.graph_c0	3.26E-25	-1.4085	down	hypothetical protein KK1_033668 [Cajanus cajan]
c95421.graph_c0	1.23E-20	1.80903	up	hypothetical protein PGTG_10268 [Puccinia grami
c52452.graph_c0	1.27E-22	-1.8931	down	STE like transcription factor [Diaporthe helianthi]
c53972.graph_c0	2.74E-19	1.70673	up	putative secreted aspartic proteinase precursor [Dip
c72643.graph_c0	4.92E-07	1.02178	up	PREDICTED: E3 ubiquitin-protein ligase PUB22 [
c38035.graph_c0	9.77E-18	-1.6549	down	hypothetical protein DHEL01_v200180 [Diaporthe
c38123.graph_c0	1.90E-17	-1.6578	down	hypothetical protein UCDDS831_g02644 [Diplodia
c98390.graph_c0	3.01E-08	-1.0187	down	hypothetical protein UCDDA912_g00683 [Diaport
c94301.graph_c1	4.57E-24	1.73294	up	predicted protein [Hordeum vulgare subsp. vulgare
c71104.graph_c0	1.76E-18	1.06778	up	hypothetical protein PHAVU_009G158100g [Phas
c76838.graph_c0	2.34E-15	1.22042	up	PREDICTED: glycine cleavage system H protein, n
c58605.graph_c0	2.94E-15	-1.5163	down	hypothetical protein M426DRAFT_13512 [Hypoxy
c37683.graph_c0	7.54E-07	-1.0081	down	glutamine synthetase [Fusarium oxysporum f. sp. ly
c77657.graph_c0	1.56E-07	-1.0245	down	hypothetical protein BKCO1_1300023 [Diplodia co
c40010.graph_c0	1.44E-37	-2.4452	down	hypothetical protein DHEL01_v202423 [Diaporthe
c72054.graph_c0	1.48E-10	1.10744	up	PREDICTED: uncharacterized protein LOC102669
c92286.graph_c0	#######	1.14693	up	beta-galactosidase 1-like isoform X1 [Cajanus caja
c86263.graph_c2	1.84E-15	1.56118	up	cytochrome c oxidase subunit I, partial (mitochond
c76633.graph_c0	#######	2.22707	up	alpha-dioxygenase 1 [Cajanus cajan]
c95641.graph_c0	8.83E-18	1.51286	up	hypothetical protein LR48_Vigan303s004900 [Vig
c97072.graph_c0	5.25E-09	-1.082	down	putative extracellular exo [Diplodia seriata]
c79245.graph_c0	8.77E-58	1.55255	up	PREDICTED: uncharacterized protein LOC100780
c97022.graph_c0	2.28E-10	-1.1922	down	putative lipase 2 [Diplodia seriata]
c80959.graph_c0	1.97E-58	1.23425	up	PREDICTED: uncharacterized protein LOC108342
c37729.graph_c0	6.46E-27	1.38718	up	PREDICTED: protein YLS9 [Glycine max]
c88864.graph_c0	2.66E-20	-1.6544	down	putative pectate lyase 2 [Cajanus cajan]
c57918.graph_c0	1.27E-25	-2.0178	down	putative allantoate permease [Diplodia seriata]
c89195.graph_c0	3.16E-12	-1.3689	down	nitrate reductase [Fusarium langsethiae]
c91933.graph_c1	1.95E-75	1.01885	up	hypothetical protein GLYMA_20G183700 [Glycin
c91323.graph_c1	1.56E-65	-3.1428	down	hypothetical protein DHEL01_v206890 [Diaporthe
c85258.graph_c1	1.69E-36	-1.117	down	NBS-LRR disease resistance protein [Medicago tru
c77436.graph_c0	2.59E-18	1.68522	up	hypothetical protein KFL_003940020 [Klebsormidi
c95123.graph_c0	1.05E-09	-1.1739	down	uncharacterized protein LOC106770264 [Vigna rad
c75292.graph_c0	2.07E-69	1.22691	up	unknown [Glycine max]
c86893.graph_c0	1.22E-20	1.79138	up	PREDICTED: gibberellin 20 oxidase 2-like [Glycin

c73735.graph_c0	2.30E-25	-2.0117	down	putative riboflavin transporter mch5 [Diaporthe am
c68689.graph_c0	3.39E-11	-1.294	down	putative transcriptional regulatory protein C15D4.0
c95562.graph_c0	1.23E-12	-1.3527	down	NADPH-dependent FMN reductase [Diaporthe heli
c75768.graph_c0	2.94E-54	2.20119	up	12-oxophytodienoate reductase 1 [Cajanus cajan]
c95417.graph_c0	3.82E-09	1.08564	up	Cytochrome P450 [Macrophomina phaseolina MS6
c38187.graph_c0	5.86E-09	-1.0786	down	hypothetical protein BKCO1_2200012 [Diplodia co
c74299.graph_c0	1.10E-08	1.12504	up	nitroreductase [Diplodia corticola]
c87280.graph_c0	7.73E-81	1.098	up	cell division control protein 48 homolog E isoform
c92701.graph_c0	#######	1.41167	up	putative nitronate monooxygenase-like protein [Tri
c54063.graph_c0	3.24E-48	-2.6567	down	putative glycosyl transferase family 2 protein [Neof
c89817.graph_c0	4.28E-09	1.18037	up	ribosomal protein L19 [Ostreococcus lucimarinus C
c76909.graph_c0	8.17E-26	-2.0303	down	hypothetical protein CGGC5_8688 [Colletotrichum
c88288.graph_c0	2.92E-09	1.17997	up	predicted protein [Hordeum vulgare subsp. vulgare]
c37789.graph_c1	3.38E-10	-1.1789	down	plasma membrane ATPase-1 [Diaporthe helianthi]
c88524.graph_c0	8.15E-32	1.30919	up	PREDICTED: transaldolase [Glycine max]
c57051.graph_c0	9.14E-21	-1.8088	down	putative mfs multidrug transporter [Diaporthe ampe
c97289.graph_c0	5.47E-09	1.11888	up	putative indoleaminedioxygenase family protein [D
c95931.graph_c0	6.03E-16	-1.5569	down	putative 2og-fe oxygenase [Diaporthe ampelina]
c73872.graph_c0	7.99E-09	-1.0677	down	cell pattern formation-associated protein stuA [Dia
c78580.graph_c0	1.52E-09	1.19056	up	polygalacturonase PG1 precursor [Glycine max]
c37760.graph_c0	1.76E-10	-1.1987	down	putative secreted protein [Diaporthe ampelina]
c92006.graph_c0	6.14E-35	-2.2526	down	short chain dehydrogenase [Diaporthe helianthi]
c37916.graph_c0	1.01E-51	1.9943	up	PREDICTED: L-lactate dehydrogenase B-like [Gly
c37515.graph_c0	1.60E-11	-1.2771	down	hypothetical protein UCDDA912_g05724 [Diaport
c38016.graph_c0	1.81E-13	-1.4533	down	putative apoptosis-inducing factor 1 [Diplodia seria
c96249.graph_c0	1.96E-11	-1.2866	down	putative amidase [Hypoxylon sp. EC38]
c91922.graph_c0	6.36E-07	-1.0031	down	hypothetical protein FPOA_08123 [Fusarium poae]
c88923.graph_c0	4.84E-14	-1.4818	down	hypothetical protein FPOA_04072 [Fusarium poae]
c89524.graph_c0	1.64E-83	1.11375	up	6-phosphofructokinase 3 [Glycine soja]
c95336.graph_c0	1.15E-29	2.17828	up	hypothetical protein UCDDS831_g05006 [Diplodia
c67742.graph_c0	5.17E-12	1.30296	up	putative major allergen alt protein [Neofusicoccum
c47840.graph_c0	1.48E-31	-2.2479	down	amino-acid permease inda1 [Diaporthe helianthi]
c83308.graph_c2	1.44E-12	-1.3493	down	glutamine synthetase [Diaporthe helianthi]
c96318.graph_c0	9.89E-11	-1.2365	down	cytochrome p450 [Diplodia corticola]
c80031.graph_c0	2.61E-08	-1.1209	down	probable glutamate decarboxylase [Fusarium prolif
c73278.graph_c0	4.88E-96	-1.176	down	gibberellin-regulated protein 1 [Cajanus cajan]
c82911.graph_c0	6.63E-15	-1.3532	down	Endoglucanase 5 [Glycine soja]
c37958.graph_c0	1.05E-08	1.11947	up	cytochrome c oxidase subunit 3 (mitochondrion) [N
c54982.graph_c0	1.90E-09	-1.1199	down	RNA recognition domain-containing protein [Diapo
c98237.graph_c0	5.43E-09	-1.0817	down	hypothetical protein DHEL01_v200979 [Diaporthe
c71268.graph_c0	1.82E-20	-1.7939	down	alpha-amylase 1 [Diaporthe helianthi]
c95812.graph_c0	8.43E-26	-2.0296	down	clock-controlled gene-15 [Diaporthe helianthi]
c54633.graph_c1	1.41E-19	-1.7498	down	heavy metal tolerance protein [Diaporthe helianthi]
c97394.graph_c0	7.21E-11	-1.2259	down	Nitrate transporter [Diplodia seriata]
c95940.graph_c0	4.81E-13	-1.3778	down	putative oxalate protein [Neofusicoccum parvum U
c96769.graph_c0	1.88E-10	-1.1977	down	rhamnogalacturonate lyase a [Diplodia corticola]

c75347.graph_c0	1.77E-09	1.12224	up	serpin-ZXA-like [Dendrobium catenatum]
c54203.graph_c0	1.09E-20	-1.8043	down	hypothetical protein DHEL01_v212164 [Diaporthe
c38490.graph_c0	5.45E-09	-1.1606	down	mfs sugar transporter [Diplodia corticola]
c38190.graph_c1	2.80E-10	-1.1854	down	phosphorus acquisition-controlling protein [Diaport
c93711.graph_c1	4.37E-32	1.11066	up	PREDICTED: ACT domain-containing protein AC
c38524.graph_c0	5.30E-09	-1.0949	down	putative methyltransferase [Aspergillus novofumiga
c78961.graph_c0	#######	-1.0649	down	hypothetical protein KK1_007678, partial [Cajanus
c95532.graph_c0	#######	-4.0219	down	putative multicopper oxidase [Diaporthe ampelina]
c39619.graph_c1	5.23E-11	-1.2392	down	putative aspartate aminotransferase protein [Phaeoa
c57713.graph_c0	1.18E-39	2.476	up	putative epl1 protein [Neofusicoccum parvum UCR
c39071.graph_c0	9.19E-08	1.06431	up	hypothetical protein PHAVU_009G131000g [Phase
c95519.graph_c0	3.30E-51	-2.7278	down	Sugar/inositol transporter [Macrophomina phaseoli
c88639.graph_c0	3.47E-07	-1.0371	down	hypothetical protein FPSE_00124 [Fusarium pseud
c75973.graph_c0	1.94E-17	-1.0476	down	PREDICTED: mannan endo-1,4-beta-mannosidase
c96102.graph_c0	5.17E-09	-1.082	down	putative isochorismatase hydrolase protein [Neofus
c82042.graph_c0	9.72E-76	-3.1729	down	putative zinc finger containing protein [Diplodia se
c96030.graph_c0	1.29E-24	-1.9809	down	hypothetical protein DHEL01_v209067 [Diaporthe
c57665.graph_c0	1.69E-13	1.20553	up	putative mannitol dehydrogenase, partial [Cajanus c
c95987.graph_c0	1.08E-38	-2.4801	down	nitrite reductase [Diaporthe helianthi]
c93614.graph_c1	4.14E-10	1.03416	up	uncharacterized protein LOC109798047 [Cajanus c
c75971.graph_c0	1.26E-30	-2.024	down	probable 2-oxoglutarate-dependent dioxygenase AC
c82226.graph_c1	1.54E-18	-1.6979	down	hypothetical protein DHEL01_v209978 [Diaporthe
c88648.graph_c0	1.48E-10	-1.2691	down	putative cmgc mapk protein kinase [Diaporthe amp
c54965.graph_c0	1.06E-32	1.32279	up	PREDICTED: uncharacterized protein LOC100804
c93555.graph_c0	6.04E-52	1.11081	up	hypothetical protein PHAVU_002G290800g [Phase
c92014.graph_c1	1.57E-18	-1.4542	down	pentose phosphate metabolism-2 [Diaporthe heliant
c55439.graph_c0	1.72E-13	-1.41	down	hypothetical protein PFICI_00156 [Pestalotiopsis fi
c85016.graph_c0	1.56E-07	-1.0541	down	integrator complex subunit 11 [Dendrobium catena
c96449.graph_c0	2.61E-11	-1.2613	down	hypothetical protein DHEL01_v209474 [Diaporthe
c87163.graph_c0	2.09E-16	1.60504	up	60S ribosomal protein L10 [Sorghum bicolor]
c39866.graph_c1	4.50E-08	-1.0027	down	hypothetical protein DHEL01_v209106 [Diaporthe
c69739.graph_c0	1.97E-33	-2.3119	down	putative tannase subunit [Diaporthe ampelina]
c68455.graph_c0	1.21E-08	-1.0847	down	hypothetical protein UCDDS831_g00989 [Diplodia
c38011.graph_c0	1.60E-64	-3.1259	down	atpase aaa+ type core protein [Diplodia corticola]
c95727.graph_c0	5.54E-12	-1.3089	down	hypothetical protein DHEL01_v205104 [Diaporthe
c38051.graph_c0	1.95E-08	-1.069	down	hypothetical protein BK809_0005152 [Diplodia ser
c80941.graph_c0	#######	3.30798	up	hypothetical protein PHAVU_002G305200g [Phase
c57523.graph_c0	1.83E-12	1.33172	up	hypothetical protein PSACC_03723 [Paramicrospo
c37306.graph_c0	1.12E-09	-1.1364	down	malate dehydrogenase [Diaporthe helianthi]
c95492.graph_c0	1.62E-18	-1.6981	down	PREDICTED: alpha carbonic anhydrase 7-like [Gly
c57632.graph_c2	7.16E-11	-1.2302	down	calcium P-type ATPase-2 [Diaporthe helianthi]
c68809.graph_c0	9.58E-15	-1.4859	down	hypothetical protein MYCTH_2061639 [Thermoth
c95713.graph_c0	9.15E-30	-2.173	down	multicopper oxidase [Diplodia corticola]
c89629.graph_c0	3.00E-52	-1.8073	down	xyloglucan endotransglucosylase/hydrolase family
c85062.graph_c0	1.76E-17	1.1654	up	hypothetical protein glysoja_041412 [Glycine soja]
c61452.graph_c1	1.32E-22	-1.8929	down	hypothetical protein DHEL01_v208629 [Diaporthe

c37795.graph_c0	2.99E-14	1.30788	up	hypothetical protein PHAVU_006G103800g [Phase
c71965.graph_c0	5.48E-08	-1.0404	down	Proteinase inhibitor I13 potato inhibitor I [Macroph
c89819.graph_c2	6.16E-12	1.10055	up	PREDICTED: NADP-dependent malic enzyme-like
c40188.graph_c0	7.30E-10	-1.1529	down	hypothetical protein VFPPC_08845 [Pochonia chla
c77537.graph_c0	9.39E-12	1.28339	up	receptor for activated protein kinase C [Chondrus c
c58306.graph_c0	5.72E-14	-1.4388	down	NADPH dehydrogenase [Diaporthe helianthi]
c76630.graph_c0	1.19E-11	1.30832	up	ribosomal protein L28e [Absidia repens]
c68169.graph_c0	1.42E-09	1.11327	up	RecName: Full=Kirola; AltName: Allergen=Act d
c82538.graph_c0	6.50E-13	-1.3719	down	Hexose transporter HXT13 [Valsa mali]
c93434.graph_c3	2.23E-11	1.12179	up	Pleiotropic drug resistance protein 1 [Glycine soja]
c86642.graph_c0	1.90E-23	-1.867	down	beta-1,3-glucanosyltransferase [Diaporthe helianthi
c76132.graph_c0	8.97E-16	1.03192	up	PREDICTED: UDP-glycosyltransferase 73C3-like
c87073.graph_c1	1.30E-07	1.06231	up	PREDICTED: SNF1-related protein kinase regulato
c84401.graph_c0	1.11E-89	2.16391	up	PREDICTED: 3-oxo-Delta(4,5)-steroid 5-beta-redu
c91473.graph_c0	6.57E-73	1.08926	up	MYB/HD-like transcription factor [Glycine max]
c76688.graph_c0	1.22E-07	-1.0333	down	hypothetical protein PHAVU_006G075700g [Phase
c39701.graph_c1	5.90E-10	-1.2078	down	putative fatty acid oxygenase [Diplodia seriata]
c79298.graph_c0	1.48E-42	-2.5607	down	hypothetical protein DHEL01_v206493 [Diaporthe
c37763.graph_c0	3.55E-21	-1.8269	down	hypothetical protein MYCTH_2110682 [Thermothe
c68022.graph_c0	4.68E-08	-1.0006	down	hypothetical protein DHEL01_v203398 [Diaporthe
c68717.graph_c0	4.72E-18	-1.6902	down	putative hexose transporter protein [Diplodia seriata
c90756.graph_c2	#######	1.54631	up	alpha' subunit of beta-conglycinin, partial [G
c88955.graph_c0	2.72E-83	1.15763	up	uncharacterized protein LOC100305676 [Glycine n
c40212.graph_c0	1.09E-08	-1.057	down	glucose-methanol-choline oxidoreductase [Arthrod
c37861.graph_c0	6.91E-55	1.39821	up	seed maturation protein PM35 [Glycine max]
c95766.graph_c0	2.28E-10	-1.2174	down	glycoside hydrolase family 61 protein [Diplodia co
c94328.graph_c1	2.89E-13	1.40881	up	predicted protein [Hordeum vulgare subsp. vulgare
c90925.graph_c0	6.13E-10	-1.2369	down	NADPH-ferrihemoprotein reductase [Fusarium vert
c38465.graph_c0	2.13E-23	-1.9284	down	putative glycoside hydrolase family 16 protein [Dia
c78289.graph_c0	2.69E-17	-1.6322	down	Lactose permease [Valsa mali]
c79653.graph_c0	1.76E-60	2.55743	up	PREDICTED: probable tyrosine-protein phosphata
c79669.graph_c0	2.26E-65	2.04777	up	F-box protein At2g27310-like [Cajanus cajan]
c67703.graph_c0	8.69E-99	-3.6091	down	lactose permease protein [Diplodia corticola]
c80361.graph_c0	7.48E-22	-1.7533	down	putative glutamine synthetase protein [Neofusicocc
c74990.graph_c0	4.29E-16	-1.5664	down	MFS general substrate transporter [Aspergillus taic
c88270.graph_c0	3.10E-23	-1.8506	down	putative glycosyl hydrolase family 7 protein [Neofu
c82859.graph_c0	3.07E-13	-1.4123	down	putative aspergillopepsin-2 protein [Neofusicoccun
c82920.graph_c0	1.72E-31	-1.8211	down	PREDICTED: putative pectinesterase/pectinesteras
c83434.graph_c0	1.02E-35	-1.962	down	PREDICTED: cationic peroxidase 1 [Glycine max]
c97161.graph_c0	4.03E-08	-1.0075	down	putative solute carrier family 35 member e3 [Diapo
c55680.graph_c0	1.18E-10	-1.2127	down	putative glucose-6-phosphate 1-dehydrogenase [Dia
c78200.graph_c0	1.73E-10	1.20193	up	ribosomal L18p/L5e [Helicosporidium sp. ATCC 5
c78582.graph_c0	#######	-3.6731	down	putative btb poz-like protein [Diplodia seriata]
c93811.graph_c0	1.08E-32	-1.4776	down	hypothetical protein GLYMA_20G047600 [Glycine
c85642.graph_c0	2.86E-07	-1.0199	down	hypothetical protein FPSE_03661 [Fusarium pseud
c80420.graph_c0	1.58E-16	1.54866	up	DUF4228 domain protein [Medicago truncatula]

c95402.graph_c0	3.65E-19	-1.7302	down	hypothetical protein PFICI_00156 [Pestalotiopsis fi
c76225.graph_c1	4.27E-24	1.0852	up	PREDICTED: LOB domain-containing protein 41-l
c55966.graph_c0	5.11E-21	-1.8207	down	MFS quinate transporter [Diaporthe helianthi]
c96381.graph_c0	2.63E-09	-1.1072	down	NHL repeat-containing protein [Aspergillus steynii
c69825.graph_c0	1.42E-14	-1.5124	down	ammonium transporter [Diplodia corticola]
c82640.graph_c1	6.51E-10	-1.2293	down	eukaryotic translation initiation factor eIF-1 [Fusari
c78159.graph_c0	1.92E-29	-2.1708	down	high-affinity methionine permease [Colletotrichum
c96411.graph_c0	1.23E-11	-1.2831	down	putative ral amino-acid permease gap1 [Diaporthe a
c79812.graph_c0	7.24E-11	1.04508	up	PREDICTED: uncharacterized protein LOC102665
c87152.graph_c0	1.09E-11	-1.029	down	PREDICTED: cinnamoyl-CoA reductase 2-like [Lu
c95623.graph_c0	5.03E-26	-2.0387	down	putative pectinesterase A [Diplodia seriata]
c38126.graph_c0	4.52E-15	-1.5068	down	hypothetical protein DHEL01_v208702 [Diaporthe
c65214.graph_c0	3.99E-09	-1.093	down	hypothetical protein DHEL01_v201142 [Diaporthe
c38729.graph_c0	3.15E-13	-1.3926	down	hypothetical protein DHEL01_v200797 [Diaporthe
c73316.graph_c0	#######	1.64455	up	PREDICTED: myb-related protein Myb4-like [Gly
c55893.graph_c0	8.07E-18	-1.6601	down	hypothetical protein VP1G_00949 [Valsa mali var.
c95290.graph_c0	1.92E-13	1.42368	up	--
c82654.graph_c0	0	2.22264	up	embryonic abundant-like protein [Medicago truncat
c84485.graph_c0	8.00E-20	-1.7618	down	hypothetical protein DHEL01_v211129 [Diaporthe
c95210.graph_c0	2.73E-12	-1.0767	down	Retrovirus-related Pol polyprotein from transposon
c80545.graph_c0	1.03E-12	1.4098	up	PREDICTED: flavonol synthase/flavanone 3-hydro
c84481.graph_c0	2.08E-18	-1.707	down	pectin lyase [Fusarium langsethiae]
c37705.graph_c0	3.51E-75	2.71858	up	LOW QUALITY PROTEIN: GDSL esterase/lipase
c90978.graph_c0	1.37E-21	1.23782	up	hypothetical protein PHAVU_007G168000g [Phase
c95684.graph_c0	9.41E-12	-1.2913	down	kinase-like protein [Stagonospora sp. SRC1lsM3a]
c53880.graph_c0	5.29E-19	1.71703	up	--
c82135.graph_c0	4.54E-09	-1.164	down	hypothetical protein UCDDS831_g01262 [Diplodia
c94180.graph_c0	2.69E-50	2.61174	up	predicted protein [Hordeum vulgare subsp. vulgare]
c59651.graph_c0	5.70E-19	-1.7198	down	nucleoside-triphosphatase, RNA helicase [Pseudolo
c95345.graph_c0	3.08E-10	-1.1784	down	hypothetical protein P174DRAFT_442171 [Asperg
c95827.graph_c0	5.85E-86	-3.5314	down	hypothetical protein MPH_13779 [Macrophomina p
c38636.graph_c1	3.52E-08	-1.0131	down	putative cyclin-like protein [Diaporthe ampelina]
c80950.graph_c0	3.36E-09	1.08995	up	40S ribosomal protein S5 [Klebsormidium nitens]
c93803.graph_c0	5.56E-41	-1.9048	down	putative alcohol oxidase protein [Neofusicoccum pa
c37078.graph_c1	2.35E-09	1.10324	up	putative cytochrome p450 3a9 protein [Neofusicocc
c86082.graph_c0	1.56E-07	-1.0099	down	Putative respiratory burst oxidase isogeny protein H
c89070.graph_c3	6.88E-60	1.02946	up	defensin-like protein [Cajanus cajan]
c85214.graph_c0	6.89E-08	1.09165	up	60S ribosomal protein L27a-3 [Monoraphidium neg
c95843.graph_c0	2.39E-08	-1.1235	down	putative mfs sugar transporter [Diplodia seriata]
c95261.graph_c0	7.12E-41	1.56913	up	hypothetical protein glysoja_025159 [Glycine soja]
c95552.graph_c0	2.79E-29	1.6913	up	pathogenesis-related protein PR-4-like [Cajanus caj
c55591.graph_c0	8.15E-08	1.01819	up	gpi-anchored cell wall organization protein ecm33 [
c95234.graph_c0	5.59E-25	1.88073	up	calcium-binding EF-hand family protein [Glycine m
c58696.graph_c0	1.14E-08	1.04585	up	cytosine deaminase, partial [Leucosporidium creatin
c87176.graph_c0	3.76E-09	1.1436	up	Ribosomal 60S subunit protein L12 [Rhizophagus i
c92008.graph_c0	2.78E-42	1.07488	up	hypothetical protein LR48_Vigan02g166300 [Vigna

c70155.graph_c0	4.84E-13	-1.4057	down	hypothetical protein UCDDS831_g05530 [Diplodia
c75903.graph_c0	6.36E-13	-1.3924	down	hypothetical protein FPOA_02826 [Fusarium poae]
c94044.graph_c2	7.57E-16	-1.3977	down	seed maturation protein PM34 [Glycine max]
c95571.graph_c0	3.69E-26	-2.0444	down	putative acid proteinase [Diaporthe ampelina]
c85308.graph_c0	4.23E-78	1.20649	up	probable glutathione S-transferase [Morus notabilis
c86957.graph_c8	1.94E-25	1.12633	up	Protein TRANSPARENT TESTA 12 [Glycine soja
c37692.graph_c0	1.63E-19	-1.7475	down	NAD-dependent epimerase/dehydratase, putative [
c58153.graph_c1	2.42E-07	-1.0054	down	h+ nucleoside cotransporter [Diplodia corticola]
c77566.graph_c0	1.69E-39	-2.4895	down	c2h2 transcription factor [Diplodia corticola]
c70289.graph_c0	5.14E-18	-1.6737	down	hypothetical protein BK809_0007542 [Diplodia se
c95653.graph_c0	1.03E-26	-2.0666	down	hypothetical protein DHEL01_v207178 [Diaporthe
c91644.graph_c0	3.47E-08	1.11208	up	60S ribosomal protein L5 [Achlya hypogyna]
c73296.graph_c0	1.14E-20	-1.568	down	putative lipid-transfer protein DIR1 [Cajanus cajan
c74422.graph_c0	1.17E-09	-1.1388	down	polyketide synthase [Diplodia corticola]
c78416.graph_c0	2.13E-14	1.50142	up	related to tyrosinase precursor (monophenol monoo
c54190.graph_c0	4.42E-08	1.01685	up	uncharacterized protein LOC109791007 [Cajanus c
c95819.graph_c0	7.93E-31	-2.2217	down	sulfate permease [Diaporthe helianthi]
c85449.graph_c0	7.59E-11	1.26933	up	60S acidic ribosomal protein P1 [Eutrema salsugin
c96306.graph_c0	1.08E-10	-1.224	down	glycosyltransferase family 2 protein [Diplodia corti
c88201.graph_c0	5.01E-07	1.02355	up	PREDICTED: 60S ribosomal protein L9 [Populus
c74291.graph_c0	2.19E-42	-2.5876	down	2-keto-4-pentenoate hydratase [Diaporthe helianthi
c83035.graph_c0	5.70E-30	1.13927	up	putative methyltransferase DDB_G0268948 [Cajan
c95452.graph_c0	2.88E-10	1.17556	up	lysozyme [Trichophyton violaceum]
c76051.graph_c0	1.64E-26	-2.0585	down	H+/nucleoside cotransporter [Diaporthe helianthi]
c88362.graph_c0	8.55E-15	-1.519	down	unnamed protein product [Fusarium sp. FIESC_5 C
c84114.graph_c0	8.35E-20	-1.4933	down	PREDICTED: 18.2 kDa class I heat shock protein-l
c91048.graph_c0	3.23E-24	1.58215	up	hypothetical protein GLYMA_20G137900 [Glycin
c96073.graph_c0	1.44E-11	-1.2788	down	putative cdc50 family protein [Diaporthe ampelina]
c37734.graph_c0	2.08E-12	-1.3457	down	putative proline-rich protein [Neofusicoccum parvu
c76925.graph_c0	1.74E-30	-2.2092	down	hypothetical protein UCDDA912_g05228 [Diaport
c96125.graph_c0	1.98E-11	-1.2679	down	hypothetical protein ALT_4866 [Aspergillus lentul
c56179.graph_c1	3.32E-07	-1.0181	down	hypothetical protein BK809_0007407 [Diplodia se
c82444.graph_c0	7.02E-13	-1.3653	down	hypothetical protein DHEL01_v208490 [Diaporthe
c71139.graph_c0	1.86E-08	1.0268	up	ribosomal protein S6e [Basidiobolus meristosporus
c92052.graph_c1	4.46E-15	1.1715	up	ubiquinol oxidase 1 mitochondrial-like [Trifolium
c95774.graph_c0	1.86E-13	1.40004	up	putative hit domain containing protein [Neofusicoc
c74311.graph_c0	6.95E-13	1.36206	up	hypothetical protein S7711_01318 [Stachybotrys c
c83758.graph_c0	#######	-1.697	down	molybdopterin biosynthesis protein CNX1 isoform
c64715.graph_c1	2.64E-12	-1.3316	down	hypothetical protein DHEL01_v202533 [Diaporthe
c36772.graph_c0	9.88E-10	-1.1416	down	udp-glucose 4-epimerase [Diaporthe helianthi]
c67694.graph_c0	1.97E-68	-3.2158	down	major facilitator superfamily domain-containing pr
c84897.graph_c0	4.74E-14	1.47676	up	60S ribosomal protein L24, putative [Pythium insid
c84644.graph_c0	4.93E-21	1.41217	up	PREDICTED: peroxygenase 1-like [Lupinus angus
c55590.graph_c0	6.22E-14	-1.4378	down	putative MFS-type transporter PB1E7.08c [Valsa m
c91797.graph_c1	7.38E-22	-1.6708	down	probable nitrate transport protein crnA [Fusarium n
c80075.graph_c0	4.19E-11	-1.2462	down	phthalate transporter [Diaporthe helianthi]

c89167.graph_c1	1.62E-22	-1.8945	down	phosphoenolpyruvate carboxykinase (ATP) [Diapo
c93806.graph_c0	8.77E-12	1.30975	up	HDL538Wp [Eremothecium sinecaudum]
c76644.graph_c0	4.10E-47	-1.8682	down	uncharacterized protein LOC109807675 [Cajanus c
c57159.graph_c0	2.32E-09	-1.1099	down	hypothetical protein DHEL01_v201049 [Diaporthe
c55844.graph_c0	1.65E-29	-2.1733	down	hypothetical protein DHEL01_v207595 [Diaporthe
c88420.graph_c0	3.66E-49	1.11352	up	PREDICTED: pentatricopeptide repeat-containing
c95157.graph_c0	1.04E-23	1.88311	up	Retrovirus-related Pol polyprotein from transposon
c75208.graph_c0	1.60E-08	-1.0661	down	hypothetical protein BKCO1_5600048 [Diplodia co
c69219.graph_c0	9.43E-47	-2.7069	down	metacaspase [Diaporthe helianthi]
c82205.graph_c0	7.80E-20	-1.1923	down	anthocyanidin synthase [Glycine max]
c86107.graph_c0	1.46E-09	-1.11	down	hypothetical protein GLYMA_04G083500 [Glycine
c88885.graph_c0	1.39E-22	1.30109	up	PREDICTED: oxalate--CoA ligase-like [Vigna ang
c68005.graph_c0	4.28E-21	-1.8314	down	siderochrome-iron transporter [Diplodia corticola]
c80331.graph_c0	1.95E-08	-1.113	down	putative c2h2 transcription factor [Diplodia seriata]
c92606.graph_c1	7.01E-11	-1.2608	down	hypothetical protein FNYG_03084 [Fusarium nyga
c86616.graph_c0	4.50E-13	-1.4186	down	mfs peptide transporter [Diplodia corticola]
c38182.graph_c0	1.24E-22	-1.8958	down	Histidine phosphatase superfamily clade-2 [Macrop
c76204.graph_c0	#######	-2.9634	down	Defensin-like protein [Medicago truncatula]
c82638.graph_c0	1.64E-29	1.12064	up	PREDICTED: pyruvate kinase 1, cytosolic-like [M
c79288.graph_c0	#######	4.19475	up	putative polygalacturonase-inhibiting protein precu
c83796.graph_c1	8.70E-09	1.03682	up	2-methylene-furan-3-one reductase-like [Cajanus ca
c90729.graph_c2	3.52E-08	1.06106	up	aliphatic nitrilase [Diplodia corticola]
c38362.graph_c0	4.96E-10	-1.1648	down	casein kinase I isoform delta [Diaporthe helianthi]
c68963.graph_c0	1.56E-13	-1.4557	down	putative d-galacturonic acid reductase [Diplodia ser
c54769.graph_c0	3.14E-47	2.70135	up	--
c71888.graph_c1	4.30E-10	-1.1702	down	ammonium transporter MEP1 [Diaporthe helianthi]
c90289.graph_c0	1.13E-12	-1.2642	down	unnamed protein product [Fusarium sp. FIESC_5 C
c36933.graph_c1	2.27E-25	-2.0121	down	hypothetical protein DHEL01_v212385 [Diaporthe
c81687.graph_c0	2.17E-78	-3.2443	down	photosystem II protein D (chloroplast) [Ficus racem
c38072.graph_c0	6.64E-20	-1.7662	down	hypothetical protein DHEL01_v210153 [Diaporthe
c95279.graph_c0	1.10E-57	-2.7424	down	sugar transporter [Diplodia corticola]
c72234.graph_c0	1.48E-24	-1.1745	down	uncharacterized protein LOC109788712 [Cajanus c
c96135.graph_c0	2.79E-11	-1.2596	down	6-phosphogluconate dehydrogenase 2 [Diaporthe h
c70106.graph_c0	3.60E-08	-1.0526	down	Malate synthase, glyoxysomal [Diplodia seriata]
c55325.graph_c0	9.37E-11	-1.2211	down	guanine nucleotide-binding protein alpha-2 subunit
c82618.graph_c1	4.71E-14	-1.4421	down	hypothetical protein DHEL01_v204878 [Diaporthe
c95486.graph_c0	1.50E-34	-2.3495	down	hypothetical protein AOQ84DRAFT_423185 [Glor
c78521.graph_c1	2.32E-09	1.17408	up	transcription factor MYB108-like [Cajanus cajan]
c78130.graph_c0	1.62E-16	-1.5896	down	pisatin demethylase [Diaporthe helianthi]
c87409.graph_c1	1.03E-42	1.09473	up	PREDICTED: myosin heavy chain kinase B [Lupin
c84402.graph_c0	2.65E-07	-1.0359	down	related to dnase1 protein [Fusarium mangiferae]
c71293.graph_c0	2.68E-10	1.17851	up	protocadherinlike protein putative [Albugo laibachi
c78720.graph_c0	7.93E-11	1.0464	up	probable mannitol dehydrogenase [Herrania umbrat
c58492.graph_c0	2.57E-21	1.83013	up	putative npp1 domain protein [Diplodia seriata]
c96480.graph_c0	4.08E-12	-1.3182	down	4-hydroxyphenylpyruvate dioxygenase [Diaporthe h
c81899.graph_c0	4.09E-25	1.31259	up	sedoheptulose-1,7-bisphosphatase, chloroplastic [V

c78799.graph_c0	5.54E-54	1.06474	up	PREDICTED: 2-oxoglutarate-dependent dioxygena
c84671.graph_c0	#######	1.31219	up	kunitz trypsin inhibitor 2 [Arachis ipaensis]
c57714.graph_c1	4.16E-10	-1.1726	down	putative tyrosinase [Diplodia seriata]
c91951.graph_c2	1.33E-44	-1.4068	down	PREDICTED: transcription factor GAMYB-like isc
c80968.graph_c0	2.82E-08	-1.0216	down	putative erythrocyte band 7 integral membrane prot
c69666.graph_c0	#######	-3.9482	down	conidial yellow pigment biosynthesis polyketide syı
c55529.graph_c0	3.80E-16	-1.5657	down	putative histone h1 [Diaporthe ampelina]
c96004.graph_c0	2.74E-10	-1.1854	down	hypothetical protein DHEL01_v204164 [Diaporthe
c95430.graph_c0	1.34E-14	1.07897	up	nicotianamine synthase-like [Cajanus cajan]
c95706.graph_c0	#######	-4.6795	down	hypothetical protein ANO11243_042010 [fungal sp
c37927.graph_c0	2.00E-26	2.04128	up	putative NADP-dependent oxidoreductase P2 [Caja
c83143.graph_c0	5.74E-08	1.07307	up	uncharacterized protein FMAN_13540 [Fusarium n
c94161.graph_c0	2.65E-08	-1.0342	down	PREDICTED: uncharacterized protein LOC100788
c57373.graph_c0	1.38E-11	-1.2791	down	vacuolar protease A [Diaporthe helianthi]
c97826.graph_c0	7.84E-12	-1.2973	down	hypothetical protein DHEL01_v212084 [Diaporthe
c95616.graph_c0	1.16E-71	-3.2693	down	hemagglutinin [Diplodia corticola]
c93768.graph_c1	8.10E-15	1.15661	up	PREDICTED: geraniol 8-hydroxylase-like [Vigna a
c37973.graph_c0	3.08E-40	-2.5253	down	putative pectate lyase A [Diplodia seriata]
c84856.graph_c0	8.79E-42	1.34434	up	PREDICTED: lysine histidine transporter-like 8 [C
c38233.graph_c1	1.87E-17	-1.6413	down	phosphate transporter [Diaporthe helianthi]
c89944.graph_c0	2.83E-19	1.15786	up	uncharacterized protein LOC109814002 [Cajanus c
c76484.graph_c0	#######	1.13253	up	hypothetical protein GLYMA_16G204600 [Glycine
c86032.graph_c0	1.50E-15	1.15847	up	uncharacterized protein LOC109795746 [Cajanus c
c76615.graph_c2	4.04E-91	1.23221	up	PREDICTED: chalcone synthase 1 [Lupinus angust
c80355.graph_c0	2.68E-25	-1.6501	down	PREDICTED: pectinesterase-like [Glycine max]
c57784.graph_c0	4.84E-08	-1.0006	down	putative extracellular dioxygenase [Diaporthe ampe
c91090.graph_c0	1.60E-09	1.01293	up	PREDICTED: probable protein phosphatase 2C 6 [
c73002.graph_c0	8.23E-16	1.57906	up	PREDICTED: zinc finger protein ZAT11-like [Gly
c95306.graph_c0	2.42E-42	2.09578	up	PREDICTED: uncharacterized protein LOC100819
c72329.graph_c0	9.61E-13	-1.4091	down	hypothetical protein UCDDS831_g07036 [Diplodia
c81279.graph_c0	5.31E-17	-1.6167	down	hypothetical protein UCDDA912_g03080 [Diaportl
c38089.graph_c0	3.03E-10	-1.1803	down	putative mitochondrial peroxiredoxin prx1 [Diaport
c84359.graph_c0	2.73E-09	1.1933	up	hypothetical protein CEUSTIGMA_g7378.t1 [Chla
c74307.graph_c0	1.31E-11	-1.3	down	uncharacterized protein LW93_4409 [Fusarium fuji
c91998.graph_c0	4.69E-10	1.21014	up	PREDICTED: phospholipase D gamma 1 [Glycine
c55238.graph_c0	7.70E-39	-2.4547	down	putative beta- -glucan-binding protein [Neofusicocc
c97742.graph_c0	5.51E-11	-1.2378	down	GPI-anchored cell wall beta-1,3-endoglucanase Egl
c94753.graph_c1	1.45E-10	1.04128	up	hypothetical protein PHAVU_004G092100g [Phase
c83882.graph_c0	3.53E-16	-1.5983	down	hypothetical protein CSIM01_04358 [Colletotrichu
c76615.graph_c3	#######	1.39078	up	chalcone synthase 3, partial [Astragalus mongholicu
c72317.graph_c1	7.76E-12	-1.297	down	hypothetical protein DHEL01_v205835 [Diaporthe
c75906.graph_c0	9.77E-13	-1.3594	down	hypothetical protein THITE_2048634 [Thielavia ter
c83133.graph_c0	3.83E-15	1.03153	up	uncharacterized protein LOC100784877 [Glycine n
c68017.graph_c0	9.33E-36	-2.3883	down	hypothetical protein DHEL01_v202969 [Diaporthe
c93656.graph_c2	2.93E-08	-1.0197	down	unknown [Glycine max]
c95543.graph_c0	1.85E-10	-1.2724	down	glycoside hydrolase family 5 protein [Diplodia corti

c55385.graph_c0	5.52E-32	2.26387	up	hypothetical protein UCDDS831_g02021 [Diplodia
c92163.graph_c0	1.99E-07	-1.0521	down	related to putative tartrate transporter [Fusarium ma
c39758.graph_c0	1.85E-09	-1.12	down	sugar transporter [Diplodia corticola]
c74577.graph_c0	1.07E-07	-1.0764	down	hypothetical protein UCDDS831_g04100 [Diplodia
c54119.graph_c0	3.64E-08	-1.1066	down	hypothetical protein BK809_0005735 [Diplodia ser
c66152.graph_c0	1.63E-09	-1.1241	down	hypothetical protein DHEL01_v211074 [Diaporthe
c38172.graph_c0	6.01E-10	-1.1594	down	uncharacterized protein FRV6_00773 [Fusarium ox
c56037.graph_c0	1.37E-17	1.66859	up	cell wall glucanase [Diplodia corticola]
c53869.graph_c0	1.92E-11	-1.2718	down	hypothetical protein BK809_0007877 [Diplodia ser
c77801.graph_c0	4.47E-07	-1.0255	down	zinc finger transcription factor ace1 [Diplodia corti
c95719.graph_c0	4.42E-11	1.23627	up	putative protein elicitor [Diplodia seriata]
c90975.graph_c0	2.31E-93	2.33249	up	PREDICTED: putative glucose-6-phosphate 1-epim
c67910.graph_c0	3.26E-19	-1.7322	down	hypothetical protein DHEL01_v200454 [Diaporthe
c86573.graph_c0	1.07E-14	1.39522	up	RING-H2 finger protein ATL60 [Vigna radiata var.
c96464.graph_c0	2.09E-07	-1.0384	down	putative glycoside hydrolase family 3 [Diplodia seri
c76615.graph_c0	4.60E-09	1.10639	up	CHS, partial [Lotus sessilifolius]
c37949.graph_c0	2.66E-40	1.15441	up	PREDICTED: cytochrome P450 CYP73A100-like
c38457.graph_c0	3.48E-17	-1.6268	down	proteinase T [Diaporthe helianthi]
c54926.graph_c0	4.62E-07	-1.0199	down	(3S,6E)-nerolidol synthase 1-like isoform X2 [Caja
c92387.graph_c0	5.96E-15	1.22879	up	BAG family molecular chaperone regulator 6 [Caja
c95527.graph_c0	6.31E-15	-1.4967	down	--
c83686.graph_c0	2.14E-47	1.42698	up	syntaxin of plants 122 protein [Medicago truncatula
c89171.graph_c0	1.84E-07	1.0596	up	60S ribosomal protein L7a [Symbiodinium microad
c89362.graph_c0	1.55E-68	2.88115	up	primary amine oxidase [Vigna radiata var. radiata]
c72891.graph_c0	3.81E-14	1.44394	up	60S ribosomal protein L7 [Coccomyxa subellipsoid
c96082.graph_c0	3.00E-22	-1.8775	down	hypothetical protein DHEL01_v212544 [Diaporthe
c76646.graph_c0	5.77E-18	1.43559	up	lysM domain-containing GPI-anchored protein 1-lik
c80849.graph_c0	1.56E-16	-1.6083	down	RNA-binding domain-containing protein [Melinion
c91439.graph_c0	2.76E-08	-1.0349	down	chlorophyll A/B binding protein 1 [Arabidopsis tha
c95338.graph_c0	5.51E-15	-1.5223	down	scytalone dehydratase [Diplodia corticola]
c74527.graph_c1	7.67E-17	-1.6262	down	PREDICTED: probable receptor-like protein kinase
c38207.graph_c0	4.86E-29	-2.1557	down	Laccase-2, partial [Diplodia seriata]
c82120.graph_c0	7.57E-17	-1.6317	down	hypothetical protein FNYG_05807 [Fusarium nygar
c91260.graph_c0	8.91E-10	-1.0778	down	hypothetical protein FOXB_08964 [Fusarium oxysp
c86049.graph_c0	1.51E-09	-1.1633	down	uncharacterized protein FFUJ_12625 [Fusarium fuj
c91693.graph_c0	3.95E-10	-1.1863	down	nucleosome assembly protein 1-like 1 [Fusarium ve
c56903.graph_c0	3.46E-09	-1.0971	down	hypothetical protein VM1G_08555 [Valsa mali]
c77237.graph_c0	5.12E-13	-1.3983	down	putative beta-glucanosyltransferase [Diplodia seriat
c97420.graph_c0	1.07E-08	-1.0569	down	hypothetical protein DHEL01_v204821 [Diaporthe
c79317.graph_c0	1.26E-15	-1.5353	down	putative woronin body major protein [Diaporthe am
c86897.graph_c0	3.62E-21	1.83512	up	--
c54836.graph_c0	5.65E-09	-1.0799	down	hypothetical protein UCREL1_8267 [Eutypa lata U
c37716.graph_c0	1.65E-22	-1.8883	down	putative gpi anchored serine-threonine rich protein
c80279.graph_c0	6.77E-07	1.01386	up	transcription factor MYB14-like [Cajanus cajan]
c89403.graph_c0	5.11E-27	-2.0781	down	PREDICTED: serine/threonine-protein phosphatase
c89805.graph_c4	#######	1.54494	up	hypothetical protein FG05_02761 [Fusarium gramin

c88983.graph_c1	#######	1.02765	up	PREDICTED: NAD(P)H-dependent 6'-deox
c73914.graph_c0	5.83E-34	-2.3298	down	putative alcohol dehydrogenase [Diaporthe ampelin
c81862.graph_c0	2.75E-11	-1.2841	down	hypothetical protein BK809_0001124 [Diplodia ser
c90714.graph_c0	1.07E-13	1.46638	up	hypothetical protein CVT26_012180 [Gymnopilus
c85720.graph_c0	1.07E-07	1.07299	up	hypothetical protein UCRNP2_8349 [Neofusicoccu
c53827.graph_c0	1.31E-18	-1.701	down	hypothetical protein DHEL01_v210943 [Diaporthe
c86019.graph_c0	1.64E-13	-1.4525	down	unnamed protein product [Fusarium sp. FIESC_5 C
c74390.graph_c0	2.36E-09	1.05602	up	PREDICTED: probable xyloglucan endotransgluco
c40098.graph_c0	3.34E-10	-1.1799	down	SPFH domain/Band 7 family protein [Diaporthe hel
c37508.graph_c0	4.18E-30	-2.1954	down	transcriptional regulator prz1 [Diaporthe helianthi]
c85596.graph_c0	4.20E-17	-1.507	down	putative non-histone chromosomal protein 6 [Diplo
c84650.graph_c0	4.76E-43	1.52204	up	protein DMR6-LIKE OXYGENASE 1-like [Cajanu
c82896.graph_c0	2.48E-07	1.04583	up	ribosomal protein l35 [Moniliophthora roreri MCA
c60932.graph_c0	3.07E-08	-1.0169	down	C2H2 type zinc finger domain-containing protein [I
c95484.graph_c0	4.08E-26	-2.0422	down	hypothetical protein TCE0_044f16403 [Talaromyce
c38514.graph_c0	1.71E-28	-2.1351	down	hypothetical protein TRIATDRAFT_225995 [Trich
c96409.graph_c0	1.83E-07	1.01623	up	putative gpi-anchored cell wall organization protein
c89004.graph_c0	6.87E-62	1.47266	up	PREDICTED: 4-coumarate--CoA ligase 2-like [Gly
c95850.graph_c0	1.57E-09	1.11681	up	hypothetical protein UCRNP2_10381 [Neofusicocc
c74527.graph_c0	1.43E-34	-1.9841	down	PREDICTED: PTI1-like tyrosine-protein kinase 2 [
c70554.graph_c0	1.88E-23	-1.9099	down	amino acid transporter [Diplodia corticola]
c37709.graph_c0	2.26E-28	1.70209	up	uncharacterized protein LOC109801766 [Cajanus c
c81152.graph_c0	#######	1.33842	up	ribulose bisphosphate carboxylase/oxygenase activa
c81367.graph_c0	4.22E-69	1.62981	up	PREDICTED: gibberellin 20-oxidase 1 isoform X1
c72643.graph_c1	1.27E-19	1.66198	up	E3 ubiquitin-protein ligase PUB22-like [Cajanus ca
c80521.graph_c0	#######	1.3766	up	cytochrome P450 78A5-like [Cajanus cajan]
c89831.graph_c0	2.33E-11	-1.2733	down	uncharacterized protein LOC100798374 [Glycine n
c83801.graph_c0	7.28E-12	1.20464	up	PREDICTED: F-box protein PP2-B15-like [Glycine
c74059.graph_c0	9.32E-38	1.40925	up	PREDICTED: uncharacterized protein At4g22758 [
c89339.graph_c0	3.81E-16	-1.5852	down	unnamed protein product [Fusarium venenatum]
c88402.graph_c0	8.30E-23	1.57409	up	cinnamoyl-CoA reductase 1 [Arachis ipaensis]
c73429.graph_c0	1.15E-19	1.62138	up	PREDICTED: transcription factor bHLH92 [Glycin
c81168.graph_c0	3.14E-27	1.20179	up	hypothetical protein glysoja_013685 [Glycine soja]
c37723.graph_c0	3.68E-46	-2.5885	down	putative peptide transporter ptr2 [Diplodia seriata]
c82532.graph_c0	4.62E-08	1.07408	up	predicted protein [Hordeum vulgare subsp. vulgare]
c36914.graph_c0	7.20E-12	-1.2999	down	benzoate 4-monooxygenase cytochrome P450 [Dia
c79071.graph_c0	2.65E-08	-1.1044	down	PREDICTED: expansin-A7 [Glycine max]
c66667.graph_c0	9.19E-10	-1.1417	down	hypothetical protein DHEL01_v205818 [Diaporthe
c91070.graph_c1	4.60E-34	2.05154	up	IST1-like protein [Glycine soja]
c79183.graph_c0	6.91E-57	1.60174	up	PREDICTED: uncharacterized protein LOC101508
c78692.graph_c0	5.23E-11	-1.2752	down	hypothetical protein MPH_03925 [Macrophomina p
c52952.graph_c0	4.14E-09	1.0811	up	PREDICTED: uncharacterized protein LOC101505
c37737.graph_c0	3.94E-60	1.4033	up	pathogenesis-related protein STH-2-like [Cajanus c
c95564.graph_c0	2.53E-09	1.10059	up	hypothetical protein MPH_01937 [Macrophomina p
c96635.graph_c0	1.18E-24	-1.9824	down	nitrate reductase [Diaporthe helianthi]
c54596.graph_c0	1.28E-08	1.09481	up	arabinogalactan protein [Medicago truncatula]

c37908.graph_c0	4.65E-18	-1.6906	down	GPR1/FUN34/yaaH [Macrophomina phaseolina MS
c83225.graph_c0	3.98E-18	1.6319	up	copia-type polyprotein [Trifolium pratense]
c96266.graph_c0	2.38E-08	-1.0281	down	O-methyltransferase [Paraphaeosphaeria sporulosa]
c95581.graph_c0	1.11E-23	-1.9393	down	protein of unknown function DUF4243 [Penicilliun
c96129.graph_c0	4.08E-09	-1.0915	down	putative coactivator bridging factor 1 [Diaporthe an
c55588.graph_c0	5.68E-19	-1.7242	down	glycoside hydrolase family 28 protein [Diplodia cor
c85317.graph_c1	5.72E-09	1.07125	up	hypothetical protein PHYSODRAFT_500731 [Phyt
c74832.graph_c0	4.16E-30	-2.1956	down	cellodextrin transport-2 [Diaporthe helianthi]
c78362.graph_c0	1.28E-17	-1.6496	down	IDI4 [Diaporthe helianthi]
c57179.graph_c0	5.15E-10	-1.2024	down	glycoside hydrolase family 20 protein [Diplodia cor
c68628.graph_c0	1.24E-30	-2.1642	down	putative oligopeptide transporter [Diplodia seriata]
c92939.graph_c0	1.25E-18	-1.6358	down	hypothetical protein DHEL01_v207605 [Diaporthe
c56831.graph_c1	3.31E-07	1.03949	up	PREDICTED: expansin-like B1 [Glycine max]
c62879.graph_c0	5.16E-09	1.07534	up	60S ribosomal protein L18-3 [Asparagus officinalis
c86137.graph_c0	2.38E-60	1.02941	up	solute carrier family 25 member 44 isoform X1 [Ar
c97760.graph_c0	4.17E-08	-1.0064	down	peptidase S41 family protein [Diaporthe helianthi]
c95531.graph_c0	1.62E-59	2.39571	up	uncharacterized protein LOC100500462 [Glycine n
c91856.graph_c0	2.21E-12	1.3448	up	lipoxygenase, partial [Porphyra purpurea]
c94349.graph_c1	1.07E-11	-1.2789	down	chitin synthase [Fusarium oxysporum FOSC 3-a]
c38551.graph_c0	1.03E-08	-1.098	down	putative extracellular serine-rich protein [Diplodia s
c71918.graph_c0	1.47E-08	-1.0458	down	high-affinity nicotinic acid transporter [Diaporthe h
c82623.graph_c2	2.40E-20	1.78447	up	elongation factor 3, putative [Phytophthora infestan
c97704.graph_c0	6.00E-10	-1.1695	down	putative copper radical oxidase [Diplodia seriata]
c82768.graph_c0	5.02E-26	-1.4419	down	Peroxidase 7 [Glycine soja]
c94857.graph_c0	3.65E-33	-1.9308	down	elongation factor 3 [Fusarium langsethiae]
c81253.graph_c0	1.62E-45	1.16238	up	UDP-glycosyltransferase 20 [Pueraria montana var.
c86653.graph_c0	1.60E-07	1.04516	up	PREDICTED: scarecrow-like protein 21 [Glycine n
c80387.graph_c0	1.23E-23	1.93809	up	Dehydration-responsive protein RD22 [Glycine soja
c97086.graph_c0	6.72E-16	-1.5547	down	hypothetical protein UCDDA912_g04436 [Diaporth
c57327.graph_c0	7.86E-19	-1.7128	down	hypothetical protein DHEL01_v202806 [Diaporthe
c85159.graph_c0	#######	2.61038	up	uncharacterized protein LOC100816003 [Glycine n
c86912.graph_c0	1.34E-11	-1.344	down	hypothetical protein TanjilG_10332 [Lupinus angus
c78569.graph_c0	1.46E-10	1.22788	up	PREDICTED: lignin-forming anionic peroxidase-li
c95019.graph_c0	#######	1.6763	up	PREDICTED: protein SRG1 [Glycine max]
c90932.graph_c0	2.16E-20	-1.5912	down	formate dehydrogenase [Diaporthe helianthi]
c97451.graph_c0	2.16E-09	-1.125	down	amino acid transporter [Diplodia corticola]
c95660.graph_c0	9.31E-53	-2.8592	down	pectate lyase [Diplodia corticola]
c78238.graph_c1	1.18E-08	-1.1493	down	apses transcription factor [Diplodia corticola]
c86623.graph_c0	7.02E-67	1.16059	up	Wall-associated receptor kinase-like 20 [Glycine so
c38291.graph_c0	1.13E-15	-1.5689	down	WSC domain-containing protein 2 [Diplodia seriata
c41324.graph_c0	1.56E-20	-1.7972	down	hypothetical protein MYCTH_2305635 [Thermothe
c38160.graph_c0	1.32E-09	1.05844	up	expansin-like B1 [Cajanus cajan]
c82247.graph_c0	2.84E-07	1.02942	up	uncharacterized protein FMAN_08196 [Fusarium n
c95711.graph_c0	1.85E-82	-3.4002	down	glycoside hydrolase family 61 protein [Diplodia cor
c82007.graph_c0	6.93E-17	-1.6339	down	metacaspase a [Diplodia corticola]
c98223.graph_c0	6.68E-09	-1.0751	down	hypothetical protein DHEL01_v212357 [Diaporthe

c95462.graph_c0	9.79E-36	-2.3885	down	acyl-synthetase [Diplodia corticola]
c95667.graph_c0	8.97E-19	-1.7083	down	short-chain dehydrogenase [Talaromyces cellulolyti
c95266.graph_c0	#######	3.91766	up	basic 7S globulin precursor [Glycine max]
c91713.graph_c0	7.21E-11	-1.1921	down	transketolase [Diaporthe helianthi]
c73943.graph_c2	9.04E-08	-1	down	peroxisomal copper amine oxidase [Diaporthe helia
c96175.graph_c0	1.14E-14	-1.4823	down	hypothetical protein DHEL01_v212941 [Diaporthe
c78050.graph_c0	4.63E-15	-1.5061	down	putative salicylate hydroxylase [Diaporthe ampelina
c94330.graph_c1	9.99E-10	-1.0793	down	probable heat shock protein 70 [Fusarium proliferat
c57677.graph_c0	1.83E-11	-1.3253	down	Unsaturated rhamnogalacturonyl hydrolase YteR [I
c98116.graph_c0	1.50E-09	-1.1232	down	quinate permease [Diaporthe helianthi]
c80388.graph_c0	1.76E-52	1.25212	up	anthocyanidin reductase ((2S)-flavan-3-ol-forming)
c54638.graph_c0	1.68E-24	1.09085	up	hypothetical protein GLYMA_04G010900 [Glycine
c95250.graph_c0	2.38E-08	1.09761	up	PREDICTED: zinc finger protein ZAT11-like [Gly
c98211.graph_c0	1.10E-09	-1.138	down	hypothetical protein DHEL01_v209314 [Diaporthe
c95476.graph_c0	2.66E-33	-2.265	down	Galactose-binding domain-like protein [Macrophon
c79479.graph_c0	1.75E-13	1.24798	up	hypothetical protein glysoja_042282, partial [Glycin
c77663.graph_c1	7.81E-33	2.01684	up	PREDICTED: ethylene-responsive transcription fac
c95578.graph_c0	7.60E-11	1.29395	up	Survival protein SurE-like phosphatase/nucleotidas
c69533.graph_c0	6.32E-10	-1.2169	down	hypothetical protein MPH_06284 [Macrophomina p
c60659.graph_c2	1.38E-09	-1.1311	down	putative nadh:flavin oxidoreductase nadh oxidase [I
c76232.graph_c0	3.80E-12	1.35177	up	extracellular serine-threonine rich protein [Diplodia
c55112.graph_c0	2.43E-25	1.58089	up	PREDICTED: L-ascorbate oxidase homolog [Glyci
c95517.graph_c0	8.48E-08	-1.0827	down	glycoside hydrolase family 61 protein [Diplodia cor
c91421.graph_c2	2.21E-59	-2.0866	down	hypothetical protein DHEL01_v200924 [Diaporthe
c84941.graph_c1	1.92E-59	1.51444	up	Zinc finger protein ZAT10 [Glycine soja]
c93554.graph_c0	2.53E-20	1.23916	up	sucrose binding protein homolog S-64 [Glycine ma
c65367.graph_c0	9.64E-09	-1.1183	down	Transcriptional activator HAP2 [Diplodia seriata]
c95705.graph_c0	4.06E-22	-1.8714	down	putative malate dehydrogenase [Diaporthe ampelina
c72404.graph_c0	4.56E-50	1.22327	up	UGT1 [Pueraria montana var. lobata]
c95280.graph_c0	7.10E-93	-3.645	down	hypothetical protein BKCO1_610009 [Diplodia cor
c96863.graph_c0	5.36E-11	-1.2394	down	cytochrome P450 [Meliniomyces bicolor E]
c93465.graph_c0	1.85E-09	-1.1971	down	fatty acid synthase alpha subunit [Fusarium equiseti
c71677.graph_c0	1.09E-11	-1.3297	down	Short-chain dehydrogenase/reductase SDR [Macrop
c95289.graph_c0	2.92E-17	1.60598	up	hypothetical protein PHAVU_005G052500g [Phase
c95529.graph_c0	9.63E-41	2.42264	up	polygalacturonase inhibitor-like [Cajanus cajan]
c71762.graph_c0	1.58E-31	-2.2463	down	tall aerial hyphae-3 [Diaporthe helianthi]
c36775.graph_c0	8.16E-11	-1.2199	down	putative mfs transporter [Diaporthe ampelina]
c92911.graph_c0	7.65E-38	1.54346	up	ankyrin repeat-containing protein BDA1-like [Vign
c80578.graph_c0	2.55E-19	1.64581	up	PREDICTED: protein DETOXIFICATION 49-like
c85712.graph_c0	3.29E-12	1.37303	up	putative peptidase m35 deuterolysin [Diplodia seria
c84536.graph_c0	2.11E-18	1.70772	up	hypothetical protein PHAVU_007G070400g [Phase
c40248.graph_c1	6.80E-12	-1.3031	down	putative hlh transcription factor [Diaporthe ampelin
c57117.graph_c0	1.36E-17	-1.659	down	mfs aflatoxin efflux [Diplodia corticola]
c57734.graph_c0	8.94E-21	-1.8094	down	hypothetical protein DHEL01_v200533 [Diaporthe
c77457.graph_c0	2.52E-12	-1.3333	down	malic enzyme [Diaporthe helianthi]
c95712.graph_c0	1.99E-12	-1.3403	down	TPR domain-containing protein [Diaporthe helianth

c86469.graph_c0	6.03E-27	-2.0757	down	putative glyoxalase family protein [Diaporthe ampe
c69514.graph_c0	5.17E-55	-2.9128	down	hypothetical protein DHEL01_v201796 [Diaporthe
c68684.graph_c0	3.75E-34	1.02734	up	hypothetical protein GLYMA_11G070200 [Glycine
c37849.graph_c0	#######	2.67582	up	hypothetical protein PHAVU_007G222900g [Phase
c85927.graph_c0	2.92E-17	-1.5018	down	Peroxidase 7 [Glycine soja]
c68419.graph_c0	4.82E-30	1.06639	up	peroxidase N1-like isoform X1 [Durio zibethinus]
c92701.graph_c1	3.63E-61	2.27443	up	ABC transporter G family member 11 [Glycine soja
c93515.graph_c0	1.22E-08	1.13782	up	fructose-bisphosphate aldolase, cytoplasmic isozym
c54131.graph_c0	4.45E-28	1.11141	up	unknown [Lotus japonicus]
c82264.graph_c0	2.47E-08	1.11847	up	uncharacterized protein LOC109788401 [Cajanus c
c95568.graph_c0	8.20E-36	2.35213	up	small secreted protein [Diplodia corticola]
c85972.graph_c0	5.56E-33	-2.0215	down	hypothetical protein MVLG_03475 [Microbotryum
c96325.graph_c0	1.16E-12	-1.3542	down	hypothetical protein DHEL01_v202661 [Diaporthe
c89530.graph_c0	1.34E-13	1.18366	up	PREDICTED: late embryogenesis abundant protein
c79050.graph_c0	1.28E-10	-1.2794	down	c2h2 finger domain [Diplodia corticola]
c79643.graph_c1	3.86E-07	1.02026	up	glutaredoxin-like family protein [Glycine max]
c37812.graph_c0	1.91E-33	-2.3127	down	hypothetical protein M434DRAFT_22549 [Hypoxy
c86910.graph_c0	8.15E-12	-1.0229	down	UDPGT domain-containing protein [Cephalotus fol
c89791.graph_c0	7.52E-07	1.01039	up	60S acidic ribosomal protein P0 [Achlya hypogyna]
c82026.graph_c0	1.72E-14	-1.4715	down	hypothetical protein NECHADRAFT_39694 [[Nec
c88102.graph_c0	3.91E-19	1.17683	up	hypothetical protein PHAVU_009G068100g [Phase
c55588.graph_c1	9.81E-19	-1.7075	down	glycoside hydrolase family 28 protein [Diplodia cor
c70014.graph_c0	6.09E-10	-1.1867	down	alcohol oxidase [Diplodia corticola]
c89823.graph_c1	6.78E-08	-1.0732	down	PREDICTED: high affinity sulfate transporter 2-lik
c73691.graph_c0	8.38E-25	1.08028	up	PREDICTED: pyruvate kinase 1, cytosolic-like isof
c85447.graph_c0	2.79E-12	-1.3279	down	quinone oxidoreductase [Diaporthe helianthi]
c86590.graph_c0	6.44E-20	1.25872	up	Nuclear transcription factor Y subunit C-4 [Cajanus
c56186.graph_c0	2.63E-12	-1.3435	down	o-methyltransferase protein [Diplodia corticola]
c88097.graph_c0	5.08E-09	-1.149	down	unnamed protein product [Fusarium venenatum]
c94764.graph_c0	2.89E-34	1.92797	up	N-hydroxythioamide S-beta-glucosyltransferase [Ca
c76389.graph_c0	2.96E-14	1.05157	up	PREDICTED: uncharacterized protein LOC108341
c95674.graph_c0	3.26E-52	-2.8539	down	linoleate diol synthase [Diaporthe helianthi]
c96146.graph_c0	3.02E-12	1.31983	up	hypothetical protein AXG93_2779s1090 [Marchant
c82753.graph_c1	1.62E-08	1.03362	up	hypothetical protein PHAVU_011G005000g [Phase
c38109.graph_c0	2.82E-12	-1.3284	down	putative synaptobrevin [Diaporthe ampelina]
c80926.graph_c0	4.98E-13	1.25458	up	E3 ubiquitin-protein ligase PUB23-like [Cajanus ca
c90948.graph_c0	2.58E-09	-1.1293	down	ATP-dependent RNA helicase eIF4A [Colletotrichu
c78554.graph_c0	2.69E-22	-1.885	down	putative phytanoyl- dioxygenase protein [Neofusicc
c75426.graph_c0	1.94E-25	1.16367	up	U-box domain-containing protein [Glycine max]
c94463.graph_c3	2.26E-26	-1.709	down	related to PHO89-Na+/phosphate co-transporter [Fu
c92537.graph_c0	2.48E-24	-1.4855	down	putative subtilisin-like proteinase spm1 [Diaporthe
c37989.graph_c0	1.43E-54	2.34317	up	uncharacterized protein LOC109795393 [Cajanus c
c96966.graph_c0	3.38E-11	-1.2542	down	glycogen phosphorylase [Diaporthe helianthi]
c81875.graph_c0	8.31E-11	1.21487	up	lectin, partial [Allium sativum]
c87364.graph_c0	4.81E-13	-1.4291	down	Plasma membrane fusion protein prm1 [Diplodia se
c74493.graph_c0	1.15E-10	-1.2712	down	putative gpi anchored cell wall protein [Diplodia se

c92014.graph_c0	8.43E-09	-1.1596	down	bzip transcription factor [Diplodia corticola]
c36704.graph_c0	6.36E-10	-1.1506	down	hypothetical protein DHEL01_v206999 [Diaporthe
c85889.graph_c0	#######	1.77207	up	polygalacturonase inhibitor-like [Cajanus cajan]
c42611.graph_c0	3.49E-12	-1.3233	down	hypothetical protein FNYG_13792 [Fusarium nyga
c37226.graph_c0	9.47E-10	-1.1443	down	conserved hypothetical protein [Talaromyces marne
c37476.graph_c0	5.22E-08	-1.0656	down	hypothetical protein BK809_0004686 [Diplodia ser
c80136.graph_c0	1.81E-33	1.00171	up	PREDICTED: basic 7S globulin-like [Vigna angula
c85999.graph_c0	6.98E-37	1.2748	up	hypothetical protein PHAVU_001G088200g [Phase
c36973.graph_c0	2.83E-15	-1.5174	down	cytochrome p450 [Diplodia corticola]
c96051.graph_c0	3.07E-08	1.00836	up	hypothetical protein CHLNCDRAFT_59390 [Chlo
c90333.graph_c0	#######	-2.2398	down	putative esdc-like protein [Diaporthe ampelina]
c78163.graph_c0	1.72E-15	-1.5301	down	hypothetical protein DHEL01_v204253 [Diaporthe
c75465.graph_c1	5.92E-09	-1.0894	down	PREDICTED: putative expansin-A30 [Glycine max
c74796.graph_c0	2.37E-16	1.59011	up	Dehydration-responsive protein RD22 [Glycine soj
c36746.graph_c0	3.31E-08	-1.0359	down	Beta-glucosidase 1B [Diplodia seriata]
c79754.graph_c0	9.92E-23	-1.902	down	unnamed protein product [Fusarium sp. FIESC_5 C
c80611.graph_c0	9.61E-14	-1.4259	down	putative oxalate decarboxylase family bicupin [Dia
c83288.graph_c0	3.51E-11	1.30791	up	related to monocarboxylate transporter 2 [Fusarium
c39561.graph_c0	1.72E-14	1.48766	up	hypothetical protein VIGAN_08134800 [Vigna ang
c91307.graph_c0	3.68E-08	1.06327	up	Na+-ATPase [Pyropia yezoensis]
c38224.graph_c0	7.77E-24	-1.9476	down	hypothetical protein P174DRAFT_407796 [Asperg
c84216.graph_c0	7.32E-17	1.58918	up	PREDICTED: uncharacterized protein LOC109339
c90342.graph_c0	9.24E-08	1.08284	up	Mitochondrial substrate carrier family protein ancA
c68518.graph_c0	5.46E-16	1.58702	up	WRKY25 protein [Glycine max]
c55707.graph_c0	1.42E-86	-3.5343	down	nitrate transporter [Diaporthe helianthi]
c73359.graph_c0	2.28E-23	-1.9269	down	POT family protein [Diaporthe helianthi]
c76039.graph_c0	2.72E-29	1.56089	up	hypothetical protein PHAVU_003G256600g [Phase
c95848.graph_c0	1.01E-17	-1.667	down	putative aldehyde dehydrogenase-like protein [Dipl
c83659.graph_c0	1.51E-30	-2.2114	down	putative amid-like nadh [Diaporthe ampelina]
c38393.graph_c0	1.42E-20	-1.7967	down	E3 ubiquitin-protein ligase hulA [Diaporthe helian
c38059.graph_c0	2.11E-28	1.96676	up	hypothetical protein LR48_Vigan02g190900 [Vign
c86147.graph_c0	2.09E-10	1.2245	up	hypothetical protein BU14_0434s0003 [Porphyra u
c79072.graph_c0	2.93E-15	-1.5157	down	oligopeptide transporter [Diaporthe helianthi]
c70306.graph_c0	4.64E-08	-1.0143	down	hypothetical protein MPH_10408 [Macrophomina
c67447.graph_c0	7.09E-10	-1.1518	down	hypothetical protein FAVG1_08673 [Fusarium ave
c76846.graph_c0	3.20E-20	-1.7925	down	Exopolygalacturonase X-1 [Diplodia seriata]
c89065.graph_c0	3.56E-98	-1.8184	down	uncharacterized LOC100527907 precursor [Glycine
c88755.graph_c0	3.66E-17	-1.6421	down	probable indole-3-pyruvate monooxygenase YUCC
c38374.graph_c0	3.23E-13	-1.3923	down	hypothetical protein DHEL01_v210039 [Diaporthe
c77068.graph_c0	1.16E-08	1.13034	up	hypothetical protein B456_008G175600 [Gossypiu
c37037.graph_c0	5.76E-09	-1.0799	down	putative pci domain-containing protein [Diaporthe
c90380.graph_c0	#######	1.433	up	PREDICTED: 12-oxophytodienoate reductase 2 [G
c37710.graph_c0	1.89E-67	-3.1838	down	Uncharacterized protein T310_8106 [Rasamsonia e
c81180.graph_c1	5.15E-30	1.00053	up	unknown [Glycine max]
c39648.graph_c0	1.74E-09	-1.1198	down	hypothetical protein DHEL01_v200043 [Diaporthe
c80765.graph_c0	3.58E-50	1.37494	up	unknown [Glycine max]

c74710.graph_c1	6.49E-50	1.19411	up	glyceraldehyde-3-phosphate dehydrogenase, cytoso
c53938.graph_c0	8.57E-53	-2.8624	down	hypothetical protein DHEL01_v209322 [Diaporthe
c37875.graph_c0	1.34E-08	1.07951	up	hypothetical protein GOBAR_AA03427 [Gossypiu
c95374.graph_c0	1.41E-30	1.15521	up	PREDICTED: basic 7S globulin 2-like, partial [Gly
c79735.graph_c1	4.91E-12	-1.202	down	PREDICTED: probable pectinesterase/pectinesteras
c66282.graph_c1	8.56E-14	-1.4264	down	kelch repeat protein [Diaporthe helianthi]
c96147.graph_c0	1.14E-20	-1.8039	down	catalase/peroxidase HPI [Diaporthe helianthi]
c36752.graph_c0	5.19E-16	-1.5693	down	putative cytochrome p450 [Diplodia seriata]
c59007.graph_c0	1.18E-09	-1.1527	down	putative tripeptidyl-peptidase 1 precursor protein [
c95821.graph_c0	2.29E-13	-1.3973	down	hypothetical protein DHEL01_v204504 [Diaporthe
c38503.graph_c0	1.19E-09	-1.1357	down	S-adenosyl-L-methionine-dependent methyltransfer
c93208.graph_c0	6.30E-07	1.01663	up	hypothetical protein AXG93_2779s1090 [Marchant
c83516.graph_c0	8.64E-16	-1.5703	down	MFS transporter, SP family, general alpha glucosid
c55434.graph_c0	5.04E-29	1.10041	up	PREDICTED: uncharacterized protein LOC108331
c93561.graph_c0	7.33E-11	1.21953	up	predicted protein [Physcomitrella patens]
c82401.graph_c0	6.33E-20	1.76401	up	trypsin [Stemphylium lycopersici]
c78830.graph_c0	3.23E-11	-1.3055	down	woronin body major protein [Diplodia corticola]
c80327.graph_c0	3.03E-33	1.20766	up	PREDICTED: uncharacterized protein LOC100793
c37939.graph_c0	1.51E-55	-2.8193	down	hypothetical protein BKCO1_5000209 [Diplodia co
c76435.graph_c0	1.50E-09	1.11947	up	Protein component of the small ribosomal subunit [
c38447.graph_c0	1.62E-08	-1.0639	down	cfem domain-containing protein [Diplodia corticola
c37797.graph_c0	2.15E-09	1.12539	up	hypothetical protein UCRNP2_10381 [Neofusicocc
c84028.graph_c0	8.09E-10	-1.2215	down	b-zip transcription factor idi-4 [Diplodia corticola]
c38333.graph_c0	1.49E-19	-1.7498	down	non-anchored cell wall protein-1 [Diaporthe heliant
c36744.graph_c0	2.25E-10	-1.1915	down	hypothetical protein DHEL01_v210839 [Diaporthe
c40185.graph_c1	3.02E-09	-1.1034	down	aromatic amino acid aminotransferase [Diaporthe h
c95895.graph_c0	4.99E-13	-1.3791	down	oxalate decarboxylase oxdC [Diaporthe helianthi]
c97248.graph_c0	3.86E-09	-1.0929	down	hypothetical protein DHEL01_v206822 [Diaporthe
c55012.graph_c0	2.73E-46	2.68386	up	--

ning protein [Diaporthe ampelina]
ial [Phaseolus vulgaris]
acrophomina phaseolina MS6]

/sporum f. sp. radicis-cucumerinum]
nax]
ta]
janus cajan]
gnorum]

nis f. sp. tritici CRL 75-36-700-3]

lodia seriata]
Vigna angularis]
helianthi]
seriata]
he ampelina]
]
eolus vulgaris]
nitochondrial [Glycine max]
lon sp. CI-4A]
copersici MN25]
orticola]
helianthi]
230 [Glycine max]
n]
tion) [Lingulodinium polyedrum]

na angularis]

361 [Glycine max]

185 [Vigna angularis]

e max]
helianthi]
ncatula]
ium nitens]
liata var. radiata]

ne max]

pelina]
2 [Diplodia seriata]
anthi]

]
rticola]

X1 [Sorghum bicolor]
folium pratense]
usicoccum parvum UCRNP2]
CE9901]
gloeosporioides Nara gc5]
]

lina]
iplodia seriata]

orthe helianthi]

cine max]
he ampelina]
ta]

seriata]
parvum UCRNP2]

eratum]

ephroselmis olivacea]
rthe helianthi]
helianthi]

CRNP2]

helianthi]

:he helianthi]
R1-like [Glycine max]
atus IBT 16806]
cajan]

cremonium minimum UCRPA7]
:NP2]
eolus vulgaris]
na MS6]
ograminearum CS3096]
5-like [Glycine max]
icoccum parvum UCRNP2]
riata]
helianthi]
:ajan]

ajan]
)P1 [Cajanus cajan]
helianthi]
elina]
606 [Glycine max]
eolus vulgaris]
:hi]
ci W106-1]
tum]
helianthi]

helianthi]

ı seriata]

helianthi]
iata]
eolus vulgaris]
ridium saccamoebae]

/cine max]

elomyces thermophila ATCC 42464]

protein [Medicago truncatula]

helianthi]

ɛolus vulgaris]
.omina phaseolina MS6]
ɛ [Glycine max]
mydosporia 170]
rispus]

11

]
isoform X1 [Glycine max]
ory subunit beta-3-like [Glycine max]
.ctase-like [Lupinus angustifolius]

ɛolus vulgaris]

helianthi]
ɛlomyces thermophila ATCC 42464]
helianthi]
a]
lycine max]
nax]
ɛrma otae CBS 113480]

:ticola]
]
ticillioides 7600]
porthe ampelina]

se At1g05000 [Glycine max]

um parvum UCRNP2]
hungensis]
.sicoccum parvum UCRNP2]
n parvum UCRNP2]
e inhibitor 24 [Glycine max]

rthe ampelina]
aporthe ampelina]
0920]

ɛ max]
ograminearum CS3096]

ci W106-1]
like [Glycine max]

IBT 23096]

um oxysporum f. sp. cubense tropical race 4 54006]
orchidophilum]
ımpelina]
184 [Glycine max]
ıpinus angustifolius]

helianthi]
helianthi]
helianthi]
:ine max]
pyri]

:ula]
helianthi]
TNT 1-94 [Cajanus cajan]
xylase-like [Glycine max]

1-like [Cajanus cajan]
:olus vulgaris]

ı seriata]
]
ɔma neurophilia]
illus novofumigatus IBT 16806]
ɔhaseolina MS6]

arvum UCRNP2]
:um parvum UCRNP2]
[[Cajanus cajan]

glectum]

an]
[Diplodia corticola]
ıax]
ıivorum]
ırregularis DAOM 181602]
a angularis]

ı seriata]

]
]
[alaromyces marneffei ATCC 18224]

iata]
helianthi]

|

xygenase) [Fusarium mangiferae]
ajan]

eum]
cola]
euphratica]
]
us cajan]

S3069]
ike [Nicotiana attenuata]
e max]

m UCRNP2]
he ampelina]
ıs]
iata]
helianthi]
CBS 931.73]
pratense]
cum parvum UCRNP2]
artarum IBT 7711]
X1 [Cajanus cajan]
helianthi]

tein [Pseudomassariella vexata]
iosum]
tifolius]
ali]
angiferae]

the helianthi]

ajan]
helianthi]
helianthi]
protein At3g09060 [Glycine max]
TNT 1-94 [Cajanus cajan]
orticola]

max]
ularis]

mai]

homina phaseolina MS6]

alus domestica]
rsor [Phaseolus acutifolius]
ajan]

iata]

S3069]
helianthi]
osa]
helianthi]

ajan]
lianthi]

[Diaporthe helianthi]
helianthi]
ium stellatum]

us angustifolius]

i Nc14]
ica]

elianthi]
igna radiata var. radiata]

.se DAO-like [Glycine max]

›form X1 [Glycine max]
ein [Diaporthe ampelina]
ıthase [Aspergillus lentulus]

helianthi]

›. No.11243]
ınus cajan]
ıangiferae]
606 isoform X2 [Glycine max]

helianthi]

ıngularis]

icer arietinum]

ajan]
ɔ max]
ajan]
:ifolius]

:lina]
Vigna angularis]
cine max]
'590 [Glycine max]
ı seriata]
he ampelina]
:he ampelina]
mydomonas eustigma]
ikuroi]
max]
:um parvum UCRNP2]
C [Diaporthe helianthi]
ɔolus vulgaris]
m simmondsii]
ıs]
helianthi]
rrestris NRRL 8126]
ıax]
helianthi]

icola]

ı seriata]
ıngiferae]

ı seriata]
·iata]
helianthi]
:ysporum]

·iata]
:ola]

ıerase [Cicer arietinum]
helianthi]
radiata]
iata]

[Glycine max]

nus cajan]
nus cajan]

ı]
lriaticum]

lea C-169]
helianthi]
‹e [Cajanus cajan]
ıyces bicolor E]
liana]

: At5g18500 [Glycine max]

mai]
ɔorum Fo5176]
ikuroi IMI 58289]
:rticillioides 7600]

a]
helianthi]
ıpelina]

CREL1]
[Diaporthe ampelina]

: 7 long form homolog [Glycine max]
nearum]

ychalcone synthase [Cicer arietinum]
ia]
riata]
dilepis]
um parvum UCRNP2]
helianthi]
S3069]
sylase/hydrolase protein 23 [Cicer arietinum]
lianthi]

dia seriata]
us cajan]
2997]
Diaporthe helianthi]
es cellulolyticus]
noderma atroviride IMI 206040]
ecm33 [Diplodia seriata]
ycine max]
cum parvum UCRNP2]
Glycine max]

cajan]
ase, chloroplastic-like [Cajanus cajan]
[Glycine max]
ajan]

max]
e max]
[Vigna angularis]

ne max]

]
porthe helianthi]

helianthi]

556 [Cicer arietinum]
phaseolina MS6]
812 [Cicer arietinum]
ajan]
phaseolina MS6]

S6]

ı occitanis]
ıpelina]
:ticola]
:ophthora sojae]

:ticola]

helianthi]

;]
achis duranensis]

ıax]

seriata]
elianthi]
s T30-4]

lobata]
nax]
a]
he ampelina]
helianthi]
ıax]
stifolius]
ke [Glycine max]

ja]
ı]
:lomyces thermophila ATCC 42464]

ıangiferae]
:ticola]

helianthi]

icus]

inthi]
helianthi]
a]
um]
Diplodia seriata]

isoform X1 [Cajanus cajan]
e max]
cine max]
helianthi]
mina phaseolina MS6]
ne soja]
ctor 1B-like [Glycine max]
e [Macrophomina phaseolina MS6]
phaseolina MS6]
Diplodia seriata]
a corticola]
ne max]
rticola]
helianthi]

x]

a]

rticola]

i]
phomina phaseolina MS6]
eolus vulgaris]

a radiata var. radiata]
[Glycine max]
ta]
eolus vulgaris]
a]

helianthi]

ni]

:lina]
helianthi]
: max]
:olus vulgaris]

ı]
ıe-like [Chenopodium quinoa]

:ajan]

lychnidis-dioicae p1A1 Lamole]
helianthi]
. 2-like [Glycine max]

'lon sp. CO27-5]
.licularis]
]
tria] haematococca mpVI 77-13-4]
:olus vulgaris]
:ticola]

e [Glycine max]
:orm X4 [Vigna angularis]

; cajan]

ajanus cajan]
698 [Vigna angularis]

:tia polymorpha subsp. ruderalis]
:olus vulgaris]

ıjan]
ım chlorophyti]
ɔccum parvum UCRNP2]

ısarium fujikuroi IMI 58289]
ampelina]
:ajan]

:riata]
riata]

helianthi]

mai]
:ffei ATCC 18224]
iata]
ıris]
eolus vulgaris]

rella variabilis]

helianthi]
ː]
a]

S3069]
porthe ampelina]
mangiferae]
ularis var. angularis]

illus novofumigatus IBT 16806]
364 [Lupinus angustifolius]
[Paramicrosporidium saccamoebae]

eolus vulgaris]
odia seriata]

hi]
a angularis]
mbilicalis]

phaseolina MS6]
naceum]

: max]
A10 isoform X1 [Cajanus cajan]
helianthi]
m raimondii]
ampelina]
lycine max]
mersonii CBS 393.64]

helianthi]

lic [Cajanus cajan]
helianthi]
m barbadense]
'cine max]
se inhibitor 20, partial [Vigna angularis]

Neofusicoccum parvum UCRNP2]
helianthi]
rase [Glonium stellatum]
tia polymorpha subsp. ruderalis]
e:H+ symporter [Diaporthe helianthi]
185 [Vigna angularis]

250 isoform X1 [Glycine max]
orticola]
Blumeria graminis f. sp. tritici 96224]
a]
cum parvum UCRNP2]

hi]
helianthi]
elianthi]

helianthi]
