# Supplementary material for: Comparative transcriptome analysis of the effects of friction and exogenous gibberellin on germination in Abrus cantoniensis
Source: Plant Signal Behav. 2022 Nov 30;17(1):2149113. doi: 10.1080/15592324.2022.2149113 (PMC9721420; doi:10.1080/15592324.2022.2149113)
Supplement: Supplemental Material [file KPSB_A_2149113_SM4039.zip › ╕╜▒φ/Table S2.pdf]

Table S2. GO enrichment of DEGs (G1 vs G2)

#GO_classify1	GO_classify2	DEG Unigene
cellular component	extracellular region	36
cellular component	cell	327
cellular component	nucleoid	5
cellular component	membrane	319
cellular component	virion	1
cellular component	cell junction	8
cellular component	membrane-enclosed lumen	18
cellular component	macromolecular complex	94
cellular component	organelle	227
cellular component	extracellular region part	2
cellular component	organelle part	93
cellular component	virion part	1
cellular component	membrane part	280
cellular component	cell part	327
cellular component	symplast	8
molecular function	transcription factor activity, protein binding	2
molecular function	nucleic acid binding transcription factor activity	35
molecular function	catalytic activity	462
molecular function	signal transducer activity	11
molecular function	structural molecule activity	51
molecular function	transporter activity	86
molecular function	binding	394
molecular function	electron carrier activity	4
molecular function	antioxidant activity	12
molecular function	metallochaperone activity	0
molecular function	protein tag	2
molecular function	translation regulator activity	0
molecular function	nutrient reservoir activity	13
molecular function	molecular transducer activity	11
biological process	reproduction	15
biological process	cell killing	1
biological process	immune system process	0
biological process	metabolic process	535
biological process	cellular process	456
biological process	reproductive process	15
biological process	biological adhesion	0
biological process	signaling	28
biological process	multicellular organismal process	19
biological process	developmental process	22
biological process	growth	9
biological process	locomotion	2
biological process	single-organism process	418
biological process	biological phase	0
biological process	rhythmic process	0

biological process	response to stimulus	94
biological process	localization	141
biological process	multi-organism process	21
biological process	biological regulation	126
biological process	cellular component organization or biogenesis	74

Table S2. GO enrichment of DEGs (G1 vs G3)

#GO_classify1	GO_classify2	DEG Unigene
cellular component	extracellular region	52
cellular component	cell	270
cellular component	nucleoid	2
cellular component	membrane	305
cellular component	virion	1
cellular component	cell junction	11
cellular component	membrane-enclosed lumen	10
cellular component	macromolecular complex	45
cellular component	organelle	155
cellular component	extracellular region part	2
cellular component	organelle part	54
cellular component	virion part	1
cellular component	membrane part	267
cellular component	cell part	269
cellular component	symplast	11
molecular function	transcription factor activity, protein binding	3
molecular function	nucleic acid binding transcription factor activity	33
molecular function	catalytic activity	478
molecular function	signal transducer activity	7
molecular function	structural molecule activity	21
molecular function	transporter activity	86
molecular function	binding	374
molecular function	electron carrier activity	4
molecular function	antioxidant activity	21
molecular function	metallochaperone activity	0
molecular function	protein tag	0
molecular function	translation regulator activity	0
molecular function	nutrient reservoir activity	8
molecular function	molecular transducer activity	7
biological process	reproduction	12
biological process	cell killing	1
biological process	immune system process	3
biological process	metabolic process	518
biological process	cellular process	429
biological process	reproductive process	18
biological process	biological adhesion	1
biological process	signaling	19
biological process	multicellular organismal process	20
biological process	developmental process	18
biological process	growth	6
biological process	locomotion	2
biological process	single-organism process	409
biological process	biological phase	0
biological process	rhythmic process	0
biological process	response to stimulus	83
biological process	localization	126
biological process	multi-organism process	22
biological process	biological regulation	118
biological process	cellular component organization or biogenesis	60

Table S2. GO enrichment of DEGs (G1 vs G4)

#GO_classify1	GO_classify2	DEG Unigene
cellular component	extracellular region	31
cellular component	cell	166
cellular component	nucleoid	0
cellular component	membrane	180
cellular component	virion	0
cellular component	cell junction	4
cellular component	membrane-enclosed lumen	5
cellular component	macromolecular complex	46
cellular component	organelle	97
cellular component	extracellular region part	1
cellular component	organelle part	43
cellular component	virion part	0
cellular component	membrane part	157
cellular component	cell part	166
cellular component	symplast	4
molecular function	transcription factor activity, protein binding	1
molecular function	nucleic acid binding transcription factor activity	26
molecular function	catalytic activity	286
molecular function	signal transducer activity	4
molecular function	structural molecule activity	28
molecular function	transporter activity	47
molecular function	binding	226
molecular function	electron carrier activity	3
molecular function	antioxidant activity	12
molecular function	metallochaperone activity	0
molecular function	protein tag	0
molecular function	translation regulator activity	0
molecular function	nutrient reservoir activity	6
molecular function	molecular transducer activity	4
biological process	reproduction	13
biological process	cell killing	2
biological process	immune system process	1
biological process	metabolic process	322
biological process	cellular process	254
biological process	reproductive process	12
biological process	biological adhesion	0
biological process	signaling	11
biological process	multicellular organismal process	9
biological process	developmental process	12
biological process	growth	6
biological process	locomotion	2
biological process	single-organism process	252
biological process	biological phase	0
biological process	rhythmic process	0
biological process	response to stimulus	49
biological process	localization	79
biological process	multi-organism process	17
biological process	biological regulation	70
biological process	cellular component organization or biogenesis	47
