# Supplementary material for: Comparative transcriptome analysis of the effects of friction and exogenous gibberellin on germination in Abrus cantoniensis
Source: Plant Signal Behav. 2022 Nov 30;17(1):2149113. doi: 10.1080/15592324.2022.2149113 (PMC9721420; doi:10.1080/15592324.2022.2149113)
Supplement: Supplemental Material [file KPSB_A_2149113_SM4039.zip › ╕╜▒φ/Table S3.pdf]

Table S3. KEGG pathway analysis of DEGs (G1 vs G2)					
**#Pathway**	**ko_ID**	**DEG_in_Pathway**	**P-value**	**Gene_id**	**KEGG_Orthology**
Flavonoid biosynth	ko00941	9	4.92E-06	c37949.graph_c0;c70987.graph_c0;c76615.graph_c2;c80388.graph_c0;c80941.graph_c0;c81186.graph_c0;c81186.graph_c1;c86868.graph_c0;c95514.graph_	K00487+K00588+K00660+K08695+K00588+K13065+K13065+K00660+K05278
Nitrogen metabolism	ko00910	13	7.68E-06	c37683.graph_c0;c55707.graph_c0;c80361.graph_c0;c82986.graph_c0;c83281.graph_c1;c83308.graph_c0;c83308.graph_c2;c88217.graph_c0;c89195.graph_c0;c91797.graph_c1;c95492.graph_c0;c96635.graph	K01915+K02575+K01915+K01674+K00264+K01915+K01915+K00262+K10534+K02575+K01674+K10534+K025
Stilbenoid, diarylheptanoid and gingerol biosynth	ko00945	5	0.00015	c37949.graph_c0;c70987.graph_c0;c80941.graph_c0;c81186.graph_c0;c81186.graph_c1	K00487+K00588+K00588+K13065+K13065
Diterpenoid biosynthesis	ko00904	5	0.00018	c75460.graph_c0;c76181.graph_c0;c81367.graph_c0;c86893.graph_c0;c95931.graph_c0	K04125+K04124+K05282+K05282+K04124
Ribosome	ko03010	40	0.00065	c37873.graph_c0;c38034.graph_c0;c38221.graph_c1;c54876.graph_c0;c55560.graph_c0;c72364.graph_c0;c74363.graph_c0;c74454.graph_c0;c74855.graph_c0;c79634.graph_c0;c80223.graph_c0;c80232.graph_c0;c81219.graph_c0;c82890.graph_c0;c83694.graph_c1;c84515.graph_c0;c84679.graph_c0;c84821.graph_c0;c85758.graph_c0;c85921.graph_c0;c86184.graph_c0;c86350.graph_c0;c86420.graph_c0;c86637.graph_c0;c86799.graph_c0;c87185.graph_c0;c87225.graph_c0;c87501.graph_c0;c88279.graph_c1;c88310.graph_c1;c88547.graph_c0;c89007.graph_c0;c89071.graph_c0;c89766.graph_c1;c89817.graph_c0;c902	K02930+K02981+K02865+K02987+K02998+K02975+K02936+K02969+K02903+K02882+K02901+K02974+K02978+K02898+K02958+K02872+K02873+K02912+K02976+K02998+K02964+K02880+K02866+K02973+K02917+K02984+K02877+K02885+K02868+K02934+K02987+K02940+K02938+K02937+K02885+K02932+K02936+K0292
Phenylpropanoid biosynthesis	ko00940	15	0.00082	c36746.graph_c0;c37949.graph_c0;c53807.graph_c0;c68419.graph_c0;c70987.graph_c0;c76496.graph_c0;c76688.graph_c0;c80941.graph_c0;c81186.graph_c0;c81186.graph_c1;c83167.graph_c0;c84047.graph_c0;c93381.graph_c0;c95030.graph_c0;c96464.grap	K01188+K00487+K00430+K00430+K00588+K13066+K00430+K00588+K13065+K13065+K00083+K00430+K01188+K11188+K05349
Pentose and glucuronate interconversions	ko00040	12	0.00147	c37973.graph_c0;c68963.graph_c0;c81623.graph_c0;c81623.graph_c1;c84481.graph_c0;c86808.graph_c0;c87958.graph_c0;c89061.graph_c2;c89943.graph_c0;c94799.graph_c0;c95623.graph_c0;c95848.graph_c0	K01728+K18106+K00012+K00012+K01728+K00128+K01213+K01184+K00963+K00128+K01051+K00128
Phenylalanine metabolism	ko00360	8	0.00433	c37949.graph_c0;c70987.graph_c0;c73943.graph_c2;c76695.graph_c0;c80941.graph_c0;c88676.graph_c1;c89362.graph_c0;c96480.graph_c0	K00487+K00588+K00276+K07253+K00588+K01426+K00276+K00457
Glycolysis / Gluconeogenesis	ko00010	19	0.00463	c37849.graph_c0;c37916.graph_c0;c56936.graph_c0;c76461.graph_c1;c76489.graph_c0;c79606.graph_c0;c80161.graph_c1;c86808.graph_c0;c87174.graph_c0;c88075.graph_c0;c89167.graph_c1;c89765.graph_c0;c89818.graph_c0;c90852.graph_c0;c90975.graph_c0;c91060.graph_c0;c92268.graph_c0;c94799.gra	K01623+K00016+K01785+K00850+K18857+K01803+K01895+K00128+K00161+K01895+K01610+K00134+K00873+K01689+K01792+K01624+K00927+K00128+K00128

Biosynthesis of unsaturated fatty acids	ko01040	8	0.00805	c37669.graph_c0;c37939.graph_c0;c56385.graph_c0;c71677.graph_c0;c80433.graph_c0;c91839.graph_c0;c92958.graph_c0;c94255.graph_c1	K00059+K00059+K03921+K00059+K10256+K10256+K10256+K07513
Linoleic acid metabolism	ko00591	4	0.00846	c88447.graph_c1;c88447.graph_c3;c88447.graph_c4;c92705.graph_c2	K15718+K15718+K15718+K00454
Tyrosine metabolism	ko00350	8	0.01222	c73943.graph_c2;c74291.graph_c0;c76489.graph_c0;c76695.graph_c0;c89248.graph_c1;c89248.graph_c3;c89362.graph_c0;c96480.graph_c0	K00276+K01557+K18857+K07253+K00422+K00422+K00276+K00457
Taurine and hypotaurine metabolism	ko00430	4	0.01354	c80031.graph_c0;c82285.graph_c0;c83995.graph_c0;c87783.graph_c2	K01580+K01580+K10712+K18592
Isoflavonoid biosynthesis	ko00943	2	0.01658	c80054.graph_c0;c85346.graph_c0	K13265+K13264
Pyruvate metabolism	ko00620	14	0.01839	c37916.graph_c0;c70106.graph_c0;c77457.graph_c0;c80161.graph_c1;c86808.graph_c0;c87174.graph_c0;c88075.graph_c0;c89167.graph_c1;c89818.graph_c0;c90468.graph_c1;c92712.graph_c1;c94799.graph	K00016+K01638+K00029+K01895+K00128+K00161+K01895+K01610+K00873+K01958+K00026+K00128+K001
Fatty acid biosynth	ko00061	7	0.01848	c37669.graph_c0;c37939.graph_c0;c56385.graph_c0;c69666.graph_c0;c71677.graph_c0;c74422.graph_c0;c93465.graph_c0	K00059+K00059+K03921+K00665+K00059+K00665+K00667
Starch and sucrose metabolism	ko00500	16	0.02058	c36746.graph_c0;c71268.graph_c0;c81623.graph_c0;c81623.graph_c1;c81654.graph_c1;c84662.graph_c0;c87958.graph_c0;c89061.graph_c2;c89943.graph_c0;c93381.graph_c0;c95517.graph_c0;c95623.graph_c0;c95711.graph_c0;c95766.graph_c0;c96464.grap	K01188+K01176+K00012+K00012+K16055+K19356+K01213+K01184+K00963+K01188+K19356+K01051+K19356+K19356+K05349+K0068
Fatty acid metabolism	ko01212	11	0.02891	c37669.graph_c0;c37939.graph_c0;c56385.graph_c0;c69666.graph_c0;c71677.graph_c0;c74422.graph_c0;c80433.graph_c0;c91839.graph_c0;c92958.graph_c0;c93465.graph_c0;c94255.graph_c1	K00059+K00059+K03921+K00665+K00059+K00665+K10256+K10256+K10256+K00667+K07513
Cutin, suberine and wax biosynthesis	ko00073	3	0.03119	c79385.graph_c0;c79385.graph_c2;c84644.graph_c0	K13356+K13356+K17991
Isoquinoline alkaloid biosynthesis	ko00950	4	0.03428	c73943.graph_c2;c89248.graph_c1;c89248.graph_c3;c89362.graph_c0	K00276+K00422+K00422+K00276
Pentose phosphate pathway	ko00030	8	0.04419	c37849.graph_c0;c55680.graph_c0;c76461.graph_c1;c82933.graph_c0;c83968.graph_c0;c91060.graph_c0;c91713.graph_c0;c92014.graph_c1	K01623+K00036+K00850+K00036+K00616+K01624+K00615+K00033

#Pathway	ko_ID	DEG_in_Path	P-value	Gene_id	KEGG_Orthology
Table S3. KEGG pathway analysis of DEGs (G1 vs G2)					
Phenylpropanoid biosynthesis	ko00940	29	######	c36746.graph_c0;c38008.graph_c0;c55260.graph_c0;c57665.graph_c0;c66307.graph_c0;c68419.graph_c0;c69711.graph_c0;c72596.graph_c0;c73894.graph_c0;c75643.graph_c0;c76626.graph_c0;c76688.graph_c0;c78720.graph_c0;c79424.graph_c0;c80415.graph_c0;c80803.graph_c0;c81111.graph_c0;c81439.graph_c0;c82019.graph_c0;c82041.graph_c0;c82542.graph_c0;c83167.graph_c0;c83720.graph_c0;c84200.graph_c0;c84907.graph_c0;c88397.graph_c0;c90305.graph_	K01188+K13066+K00430+K00083+K13066+K00430+K13066+K00430+K05350+K00430+K13066+K00430+K00083+K00430+K00430+K00430+K00430+K00083+K00430+K13066+K01188+K00083+K00430+K00430+K00083+K00430+K00430+K00430+K05349
Diterpenoid biosynthesi	ko00904	6	######	c75460.graph_c0;c76181.graph_c0;c78770.graph_c0;c79536.graph_c5;c81367.graph_c0;c95931.graph_c0	K04125+K04124+K04125+K04123+K05282+K04124
Pentose and glucuronate interconver	ko00040	12	0.0004	c37973.graph_c0;c38104.graph_c0;c68963.graph_c0;c78580.graph_c0;c81149.graph_c0;c81665.graph_c0;c82753.graph_c1;c84481.graph_c0;c86808.graph_c0;c88864.graph_c0;c95623.graph_c0;c95848.graph_c0	K01728+K00128+K18106+K01184+K01051+K01728+K01051+K01728+K00128+K01728+K01051+K00128
Ascorbate and aldarate metabolism	ko00053	9	0.0006	c38104.graph_c0;c57963.graph_c0;c70166.graph_c0;c83882.graph_c0;c86808.graph_c0;c90174.graph_c0;c90915.graph_c1;c93971.graph_c0;c95848.graph_c0	K00128+K00423+K00423+K00469+K00128+K00469+K00423+K00423+K00128
Isoquinoline alkaloid biosynthesis	ko00950	6	0.0007	c54524.graph_c0;c73943.graph_c2;c78416.graph_c0;c89248.graph_c1;c89248.graph_c3;c89362.graph_c0	K00422+K00276+K00505+K00422+K00422+K00276
Nitrogen metabolism	ko00910	9	0.001	c37683.graph_c0;c55707.graph_c0;c80361.graph_c0;c83308.graph_c0;c83308.graph_c2;c91797.graph_c1;c95492.graph_c0;c96635.graph_c0;c97394.graph_c0	K01915+K02575+K01915+K01915+K01915+K02575+K01674+K10534+K02575
Fatty acid biosynthesis	ko00061	8	0.0024	c37669.graph_c0;c37939.graph_c0;c69666.graph_c0;c71677.graph_c0;c74422.graph_c0;c83328.graph_c0;c85980.graph_c0;c9346	K00059+K00059+K00665+K00059+K00665+K00665+K09458+K00667
Starch and sucrose metabolism	ko00500	17	0.0024	c36746.graph_c0;c71268.graph_c0;c73894.graph_c0;c74597.graph_c0;c78580.graph_c0;c81149.graph_c0;c82542.graph_c0;c82753.graph_c1;c84662.graph_c0;c88322.graph_c1;c93715.graph_c0;c95517.graph_c0;c95623.graph_c0;c95711.graph_c0;c95766.graph_c0;c96464.graph_c0;c96966.graph_c0	K01188+K01176+K05350+K00975+K01184+K01051+K01188+K01051+K19356+K01177+K01187+K19356+K01051+K19356+K19356+K05349+K00688
Tryptophan metabolism	ko00380	9	0.005	c38104.graph_c0;c40185.graph_c1;c42611.graph_c0;c79131.graph_c0;c86808.graph_c0;c88676.graph_c1;c88755.graph_c0;c92799.graph_c0;c95848.graph_c0	K00128+K00825+K11182+K07408+K00128+K01426+K11816+K01556+K00128
Flavonoid biosynthesis	ko00941	5	0.005	c39071.graph_c0;c76756.graph_c0;c80388.graph_c0;c92812.graph_c0;c94075.graph_c0	K00660+K01859+K08695+K05278+K05280
Tyrosine metabolism	ko00350	8	0.0054	c54524.graph_c0;c73943.graph_c2;c74291.graph_c0;c78416.graph_c0;c89248.graph_c1;c89248.graph_c3;c89362.graph_c0;c9648	K00422+K00276+K01557+K00505+K00422+K00422+K00276+K00457

Glyoxylate and dicarboxylate metabolism	ko00630	13	0.0105	c37683.graph_c0;c70106.graph_c0;c75903.graph_c0;c76838.graph_c0;c80361.graph_c0;c80702.graph_c0;c83308.graph_c0;c83308.graph_c2;c84426.graph_c0;c89011.graph_c1;c90932.graph_c0;c93237.graph_c0;c94	K01915+K01638+K01637+K02437+K01915+K01638+K01915+K01915+K00830+K01601+K00122+K15893+K01681
Galactose metabolism	ko00052	8	0.0109	c36772.graph_c0;c56936.graph_c0;c75405.graph_c0;c79217.graph_c0;c92950.graph_c3;c92950.graph_c5;c93715.graph_c0;c9383	K01784+K01785+K01784+K06617+K06617+K06617+K01187+K06617
Biotin metabolism	ko00780	4	0.0112	c37669.graph_c0;c37939.graph_c0;c71677.graph_c0;c85980.graph_c0	K00059+K00059+K00059+K09458
Pyruvate metabolism	ko00620	12	0.0297	c38104.graph_c0;c70106.graph_c0;c77457.graph_c0;c80161.graph_c1;c80702.graph_c0;c83298.graph_c0;c86808.graph_c0;c89167.graph_c1;c89818.graph_c0;c90468.graph_c1;c95848.graph_c0;c98237.graph_c0	K00128+K01638+K00029+K01895+K01638+K01895+K00128+K01610+K00873+K01958+K00128+K00102
beta-Alanine metabolism	ko00410	7	0.0311	c38104.graph_c0;c73943.graph_c2;c80031.graph_c0;c86808.graph_c0;c89362.graph_c0;c90814.graph_c0;c95848.graph_c0	K00128+K00276+K01580+K00128+K00276+K01580+K00128
Taurine and hypotaurin	ko00430	3	0.0458	c80031.graph_c0;c83995.graph_c0;c90814.graph_c0	K01580+K10712+K01580
Alanine, aspartate and glutamate metabolism	ko00250	10	0.0471	c37683.graph_c0;c76268.graph_c0;c77300.graph_c0;c79418.graph_c0;c80031.graph_c0;c80361.graph_c0;c83308.graph_c0;c83308.graph_c2;c84426.graph_c0;c90814.graph_c0	K01915+K00827+K01953+K00827+K01580+K01915+K01915+K01915+K00830+K01580

Table S3. KEGG pathway analysis of DEGs (G1 vs G4)					
#Pathway	ko_ID	DEG_in_Pathw	P-value	Gene_id	KEGG_Orthology
Flavonoid biosynthesis	ko00941	8	######	c37949.graph_c0;c39071.graph_c0;c76615.graph_c0;c76615.graph_c2;c76615.graph_c3;c80388.graph_c0;c80941.graph_c0;c82205.graph_c0	K00487+K00660+K00660+K00660+K00660+K08695+K00588+K05277
Phenylpropanoid biosynthesis	ko00940	13	######	c36746.graph_c0;c37949.graph_c0;c57665.graph_c0;c68419.graph_c0;c76688.graph_c0;c78569.graph_c0;c78720.graph_c0;c80941.graph_c0;c82768.graph_c0;c83434.graph_c0;c85927.graph_c0;c89004.grap	K01188+K00487+K00083+K00430+K00430+K00430+K00083+K00588+K00430+K00430+K00430+K01904+
Nitrogen metabolism	ko00910	9	######	c37683.graph_c0;c55707.graph_c0;c80361.graph_c0;c83308.graph_c2;c89195.graph_c0;c91797.graph_c1;c95492.graph_c0;c96635.graph_c0;c97394.graph	K01915+K02575+K01915+K01915+K10534+K02575+K01674+K10534+K02575
Pentose and glucuronate	ko00040	10	0.0003	c37973.graph_c0;c68963.graph_c0;c78580.graph_c0;c79735.graph_c1;c80355.graph_c0;c82753.graph_c1;c84481.graph_c0;c88864.graph_c0;c95623.graph_c0;c95848.graph_c0	K01728+K18106+K01184+K01051+K01051+K01051+K01728+K01728+K01051+K00128
Ribosome	ko03010	26	0.0014	c57523.graph_c0;c62879.graph_c0;c71139.graph_c0;c72891.graph_c0;c76435.graph_c0;c76630.graph_c0;c78200.graph_c0;c80950.graph_c0;c82532.graph_c0;c82896.graph_c0;c84359.graph_c0;c84897.graph_c0;c85214.graph_c0;c85449.graph_c0;c86147.graph_c0;c87163.graph_c0;c87176.graph_c0;c88201.graph_c0;c89171.graph_c0;c89791.graph_c0;c89817.graph_c0;c90714.graph_c0;c91644.graph_c0;c9320	K02998+K02883+K02991+K02937+K02971+K02903+K02932+K02989+K02912+K02918+K02997+K02896+K02900+K02942+K02984+K02866+K02870+K02940+K02936+K02941+K02885+K02938+K02932+K02930+
Diterpenoid biosynthesi	ko00904	3	0.0038	c81367.graph_c0;c86893.graph_c0;c95931.graph_c0	K05282+K05282+K04124
Phenylalanine metabolism	ko00360	6	0.0042	c37949.graph_c0;c73943.graph_c2;c80941.graph_c0;c89004.graph_c0;c89362.graph_c0;c96480.graph_c0	K00487+K00276+K00588+K01904+K00276+K00457
Fatty acid biosynthesis	ko00061	6	0.0052	c37669.graph_c0;c37939.graph_c0;c69666.graph_c0;c71677.graph_c0;c74422.graph_c0;c93465.graph_c0	K00059+K00059+K00665+K00059+K00665+K00667
Starch and sucrose metabolism	ko00500	12	0.0076	c36746.graph_c0;c71268.graph_c0;c78580.graph_c0;c79735.graph_c1;c80355.graph_c0;c82753.graph_c1;c95517.graph_c0;c95623.graph_c0;c95711.graph_c0;c95766.graph_c0;c96464.graph_c0;c96966.grap	K01188+K01176+K01184+K01051+K01051+K01051+K19356+K01051+K19356+K19356+K05349+K00688
Circadian rhythm - plant	ko04712	4	0.0082	c39071.graph_c0;c76615.graph_c0;c76615.graph_c2;c76615.graph_c3	K00660+K00660+K00660+K00660
Pentose phosphate pathway	ko00030	7	0.0088	c37849.graph_c0;c55680.graph_c0;c88524.graph_c0;c89524.graph_c0;c91713.graph_c0;c92014.graph_c1;c93515.graph_c0	K01623+K00036+K00616+K00850+K00615+K00033+K01623
Tyrosine metabolism	ko00350	6	0.0098	c73943.graph_c2;c74291.graph_c0;c75691.graph_c0;c78416.graph_c0;c89362.graph_c0;c96480.graph_	K00276+K01557+K00121+K00505+K00276+K00457
Glycolysis / Gluconeogenesis	ko00010	12	0.0134	c37849.graph_c0;c37916.graph_c0;c73691.graph_c0;c74710.graph_c1;c75691.graph_c0;c76484.graph_c0;c82638.graph_c0;c89167.graph_c1;c89524.graph_c0;c90975.graph_c0;c93515.graph_c0;c95848.grap	K01623+K00016+K00873+K00134+K00121+K01689+K00873+K01610+K00850+K01792+K01623+K00128
Biotin metabolism	ko00780	3	0.0216	c37669.graph_c0;c37939.graph_c0;c71677.graph_c0	K00059+K00059+K00059
Tryptophan metabolism	ko00380	6	0.0224	c40185.graph_c1;c42611.graph_c0;c88755.graph_c0;c94764.graph_c0;c95848.graph_c0;c97289.graph_	K00825+K11182+K11816+K11820+K00128+K00463

alpha-Linolenic acid metabolism	ko00592	5	0.0237	c54638.graph_c0;c75768.graph_c0;c76633.graph_c0;c85159.graph_c0;c90380.graph_c0	K08241+K05894+K10529+K05894+K05894
Isoquinoline alkaloid biosynthesis	ko00950	3	0.0358	c73943.graph_c2;c78416.graph_c0;c89362.graph_c0	K00276+K00505+K00276
Stilbenoid, diarylheptanoid and gingerol biosynthesis	ko00945	2	0.0376	c37949.graph_c0;c80941.graph_c0	K00487+K00588
Glucosinolate biosynthesis	ko00966	1	0.0486	c94764.graph_c0	K11820
Betalain biosynthesis	ko00965	1	0.0486	c78416.graph_c0	K00505
