# Supplementary material for: Comparative transcriptome analysis of the effects of friction and exogenous gibberellin on germination in Abrus cantoniensis
Source: Plant Signal Behav. 2022 Nov 30;17(1):2149113. doi: 10.1080/15592324.2022.2149113 (PMC9721420; doi:10.1080/15592324.2022.2149113)
Supplement: Supplemental Material [file KPSB_A_2149113_SM4039.zip › ╕╜▒φ/Table S4.pdf]

Table S4. Primers of the ten DEGS used for qRT-PCR

**Gene**	**Forward Primer (5'-3')**	**Reverse Primer (5'-3')**	**PCR products (bp)**	**Annealing temperature (℃)**
Gibberellin 20-oxidase 1	TTGTTGTTCACC ACTGCCCTAT	GCTCAATTACTA CCCTCCTTGTC	246	60
Primary amine oxidase	ATGAATGTCCA CCACACGCT	GCCAGCTGATT CCACCGTAT	117	60
Alpha-glucosidase-like	ACACCAATTGC AAGACCCCT	CAGTGACTGCC CCAGATTGT	127	60
Anthocyanidin synthase	ACGTCAGTTAT GCTCGCCAA	GTATGGAGACG GTGGCAACA	109	60
Caffeic acid O-methyltransferase	ATTGAACATGC ACCACCCCT	ACAGCCTTGAG GATGATGGC	95	60
Chalcone synthase	ACAGTGAGCAC AAGACCGAG	TGACCCCATTCC TTGATGGC	223	60
Flavonol synthase	ACTTCGTGCTCT GGTTGAGG	CACGTCGGGGA TCAAATGGA	131	60
1-Cys peroxiredoxin	ACACCGCTGCC TCTTCACT	ATCAACACGCT GCGTTCC	146	60
Chalcone isomerase 1	ACTTGGAGGAT GAAGCCGTG	AGCTTTTCAAA GGGGCCTGA	110	60
L-ascorbate oxidase homolog	TGTGGAAAGGC CAAACACCT	CACTTTGAGAG CCACACCCT	170	60
GAPDH	AGGTGAGAGGC TTGCTTGAC	TGGGAGGATTG GGAGGAACT	108	60
